# Co-expression network analysis identifies gonad- and embryo-associated protein modules in the sentinel species *Gammarus fossarum*

**DOI:** 10.1038/s41598-019-44203-5

**Published:** 2019-05-27

**Authors:** Davide Degli Esposti, Christine Almunia, Marc-Antoine Guery, Natacha Koenig, Jean Armengaud, Arnaud Chaumot, Olivier Geffard

**Affiliations:** 1 Irstea, UR RiverLy, Ecotoxicology Team. Centre de Lyon-Villeurbanne, 5 rue de la Doua CS 20244, 69625 Villeurbanne, France; 2 Laboratoire Innovations technologiques pour la Détection et le Diagnostic (Li2D), Service de Pharmacologie et Immunoanalyse (SPI), CEA, INRA, F-30207 Bagnols sur Cèze, France

**Keywords:** Ecophysiology, Protein-protein interaction networks

## Abstract

Next generation sequencing and mass spectrometry technologies have recently expanded the availability of whole transcriptomes and proteomes beyond classical model organisms in molecular biology, even in absence of an annotated genome. However, the fragmented nature of transcriptomic and proteomic data reduces the ability to interpret the data, notably in non-model organisms. Network-based approaches may help extracting important biological information from -omics datasets. The reproductive cycle of the freshwater crustacean *Gammarus fossarum*.provides an excellent case study to test the relevance of a network analysis in non-model organisms. Here, we illustrated how the use of a co-expression network analysis (based on Weighted Gene Co-expression Network Analysis algorithm, WGCNA) allowed identifying protein modules whose expression profiles described germ cell maturation and embryonic development in the freshwater crustacean *Gammarus fossarum*. Proteome datasets included testes, ovaries or embryos samples at different maturation or developmental stages, respectively. We identified an embryonic module correlated with mid-developmental stages corresponding to the organogenesis and it was characterized by enrichment in proteins involved in RNA editing and splicing. An ovarian module was enriched in vitellogenin-like proteins and clottable proteins, confirming the diversity of proteins belonging to the large lipid transfer family involved in oocytes maturations in this freshwater amphipod. Moreover, our results found evidence of a fine-tuned regulation between energy production by glycolysis and actin-myosin-dependent events in *G. fossarum* spermatogenesis. This study illustrates the importance of applying systems biology approaches to emergent animal models to improve the understanding of the molecular mechanisms regulating important physiological events with ecological relevance.

## Introduction

Advances in nucleic acid sequencing technologies and mass spectrometry have recently increased the availability of whole transcriptomes and proteomes beyond classical model organisms, even in absence of an annotated genome^1^. The use of proteogenomics approaches, that couple species-specific RNA-sequencing followed by the acquisition of shotgun proteomic data by high-resolution mass spectrometry for a straightforward interpretation of peptide spectra, has opened the way to get insights into the molecular mechanisms involved in the physiology and the response to environmental stress in many species of ecological relevance^1,2^.

However, the fragmented nature of proteomic and transcriptomic data without a reference genome usually prevents extensive interpretation of the data. This is particularly true in the context of the molecular physiology of non-model organisms whose genomes are evolutionarily distant from well-annotated genomes, such as those of the fly *D. melanogaster* or the human genome. Network-based approaches provide an excellent methodological framework to investigate the rich content of information from omics datasets^3,4^. Weighted Gene Co-expression Network Analysis (WGCNA) is a systems biology method originally conceived for describing correlation patterns among genes across microarrays data^4^. One of the main advantages of this statistical model is to be data driven and it represents a great opportunity in non-model organisms for which genome sequences, gene annotations, or functional pathways are not still available. WGCNA has been successfully used also for count-based (i.e. RNA-Seq based) gene expression data^5,6^. However, shotgun proteomics data have been poorly explored using WGCNA to date, despite the possibility to apply this approach to the spectral count metric largely used in this phenotypic methodology^7^. Concurrently, gene co-expression network analyses of transcriptomic data have provided useful insights in understanding fundamental biological processes in many arthropod species. For instance, WGCNA provided evidence of conserved pathways across 16 different species of ants involved in reproductive division of labor^8^. Moreover, a network analysis highlighted the role of metabolic and cell signaling genes in the stress response in the model crustacean *Daphnia magna*, when simple differential expression analysis failed to find meaningful biological answers^9^.

Our group has provided the first proteomic-based description of the reproductive systems of the freshwater amphipod *Gammarus fossarum*^2,10^. *G. fossarum* is an established and ecologically relevant test species and it has been extensively used in ecotoxicology to monitor the quality of freshwater bodies^11–13^.

Morphological and biochemical alterations in the gammarid female or male gonads are currently used as endpoint in ecotoxicology^14,15^. Testes have been reported to be particularly sensitive to genotoxic insult and minimal sperm DNA damage induced an increased abnormality rate in embryos, with potential consequences for population dynamics of this species^16^. Morphological abnormalities in embryonic development were also observed after parental exposure to heavy metals (Cd), or various organic compounds (such as nonylphenol or phenoxycarb)^14,17^. However, we do not have any detailed knowledge on the metabolic and signaling pathways underlying the full reproductive process in *G. fossarum*. The aim of this work is to develop systems biology approaches for identifying fundamental pathways and key hub proteins involved in the reproductive process and that could provide predictive biomarkers of physiological impairment following contaminant exposure. In particular, we aimed to use a co-expression network analysis through a pooled proteomics dataset including testes, ovaries or embryos at different maturation or developmental stages to identify protein pathways regulating germ cell maturation and embryonic development of *G. fossarum*. This provides an excellent case study to test the relevance of a network analysis in non-model organisms, since the structure and the molecular physiology of sperms, oocytes, and embryos are highly differentiated.

## Materials and Methods

### Proteomics datasets

We retrieved protein abundance data measured as spectral counts (SC) from two published shotgun proteomics datasets obtained by high-resolution tandem mass spectrometry using a LTQ-Orbitrap XL instrument. Only proteins validated with at least two different peptides were retained^2,10^. The datasets were merged in a matrix *n* × *m*, with *n* indicating the total number of proteins and *m* the total number of samples. Finally, the matrix consisted in 1,199 protein abundance data expressed as SC for 85 samples (Supplementary Data 1). The samples included *G. fossarum* ovaries (individual whole paired organs) and embryos (6 embryos per female) proteomes^10^ at different stages of oocyte maturation and embryo development^10,14^. Five biological replicates for each stage (AB, C1, C2, D1, D2 for ovaries and S1 to S5 for embryos) were included in this dataset. Testes were individually sampled from male organisms at 6 stages of spermatogenesis, namely at pre-copula stage (mature male in amplexus at the end of the spermatogenesis), and days after copulations (J0, J1, J2, J3, J4, J7)^2,15^. At J7 (7 days after copulation), the spermatogenesis cycle is completed. Five biological replicates for each spermatogenesis stage were included in this dataset. The annotation of the 1,199 proteins was updated compared with the original studies, by using a semi-automatic pipeline using DIAMOND^18^. Briefly, protein sequences identified by peptides that matched an ORF entry of the GFOSS RNA-seq database^2^ were blasted against the NCBInr and Swissprot databases. The closest predicted protein (indicated with NCBInr ID or SwissProt ID) and its E-value were associated with the corresponding GFOSS contig. The Gene Ontology (GO) annotation of proteins homolog found in Swissprot database was performed using data provided by the GO Consortium through the GO website and the web application Amigo (http://amigo.geneontology.org). Two levels (1 and 2) of GO annotation were extracted.

### Data pre-processing and normalization

In order to identify possible outliers, sample clustering was performed using the hierarchical clustering function implemented in the lumi R package. One sample (AB-n1) was identified as an outlier and thus excluded from the final dataset. In particular, this sample was characterized by the lowest number of total measured SC. Principal Component Analysis (PCA) was used to analyze and identify the variables explaining the maximum variance associated to the proteomic data of the different developmental stages of *G. fossarum* gonads and its embryos. In order to avoid the noise and poor reproducibility associated with low abundant proteins, proteins with less than three SC in the most abundant condition were filtered out^19^. Finally, a dataset comprising 84 samples and 375 proteins was retained for further analyses. Before network modeling, counts need to be normalized across samples^4,7^. Normalization was performed using the *calcNormFactors* function from the R package edgeR^20^. While this function has been originally implemented for RNA-Seq count data, it applies equally well to spectral count data^19^. The *calcNormFactors* function automatically adjusts for the total number of spectral counts per sample (also known as original library size in RNA-Seq experiments) and normalizes for protein composition, taking into account the fact that highly abundant proteins tend to contribute more peptide/spectra than those lowly abundant^20^. *calcNormFactors* calculate a normalization factor that was applied to normalize SC values of each protein in each sample.

### Network analysis

The network properties of the proteome of the reproductive system of *G. fossarum* were analyzed using the R package Weighted Gene Coexpression Network Analysis (WGCNA)^3,4^. Shotgun proteomics gives access to a count-based quantification of peptides that can be aggregated to a contig/protein level when a species-specific RNA-Seq database is available, as it is the case for the datasets analyzed in this study. Thus, we used the WGCNA package to investigate the count-based proteomic database of gonadic and embryonal tissues obtained from the freshwater amphipod *G. fossarum*. WGCNA was used to construct a protein co-expression network, identify protein modules in the network and correlate the identified modules to external information, such as tissue and stage. In particular, network dendrogram was built using the *blockwiseModules* function, choosing an “unsigned” network type in order to keep relationships of negatively correlated proteins and a “signed” topological overlap matrix (TOM) in order to subtract connections affected by noise^3^. TOM values are a measure of proximity (i.e. interconnection) between expressed proteins. They range from 0 to 1, with 1 indicating maximal proximity, i.e. very high interconnection. We set 20 the minimum number of proteins forming a module. Proteins outside of any modules (indicating low co-expression) were combined together in a grey module. Different clustering methods (static, dynamic, hybrid and dynamic-hybrid methods) were used to delimit the network into distinct modules^21^. Module eigengenes were calculated using the *moduleEigengenes* function. Module eigengenes are defined as the principal component of each module and they represent the protein expression profile in a module. Module eigengenes allow to explore how related the modules are and the correlation between modules and phenotypic traits, such as tissue type or maturation/developmental stages. Network properties, such as total (*k*_total_) or intramodular (*k*_within_) connectivity or module membership (ME) have been used to identify proteins that show the high degree of connectivity within a module (hub proteins). Due to their central position in the network, hub proteins are expected to play important biological role within their module.

### Reproducibility

An R script combining the whole analysis pipeline is available as Supplementary Document 1. Spectral count data and the associated metadata are available as Supplementary Data 1 and Supplementary Data 2, respectively.

## Results

### Global proteomics profiles distinguish testes, ovaries and embryos in *Gammarus fossarum*

**Figure 1:**
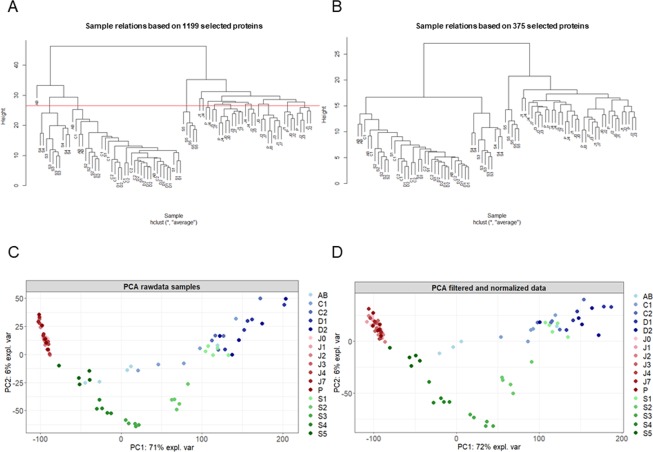
Proteomic profiles clearly distinguish testes, ovaries and embryos of *G. fossarum*. Blue dots indicate the different molt stages in females (AB, C1, C2, D1 and D2). Red dots indicate the testes at different maturation stages: P indicates testes in the pre-copula stage. J0, J1, J2, J3, J4, J7 indicate the days after copulation. Green dots indicate the different embryonic development stages (S1, S2, S3, S4, and S5). (**A**) Hierarchical clustering of raw data. (**B**) Hierarchical clustering after filtering and normalization. (**C**) Principal component analysis of raw data. (**D**) Principal component analysis after filtering and normalization.

In order to have a systemic view of the molecular processes in the reproductive organs and in the embryonic development of the sentinel species *G. fossarum*, we analyzed proteomics data obtained from individually sampled testes and ovaries at different maturation status and from individually sampled embryos obtained at different developmental stages (see Material and Methods section). Sample clustering and Principal Component Analysis (PCA) based on raw data did not change after filtering and normalization (Fig. 1A,C for raw data, Fig. 1B,D for normalized samples). The proteomes of the testes and ovaries were clearly separated and the difference across tissues accounted for up to 72% of the variance in the data (Fig. 1C,D). However, testes appeared to cluster tightly, independently from the maturation stage, while ovaries showed a much higher variation in their proteomic profiles during the maturation process. The protein profiles of embryos showed also a high level of variability, essentially explained by between-stage variability distributed along the embryonic development. Early stage embryo profiles (S1 and S2) clustered close to the vitellogenic ovary profiles (C1-D2 stage) while middle stages (S3 and S4) clustered independently and the last embryonic stage (S5) clustered even further (Fig. 1C,D).

### Distinct modules of co-expressed proteins are identified in the proteome of the reproductive system of *Gammarus fossarum*

**Figure 2:**
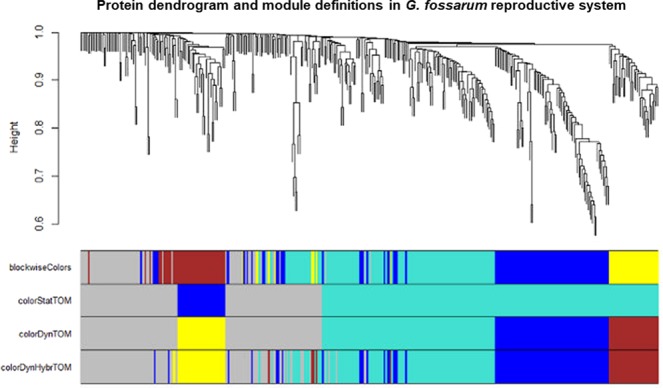
Distinct protein modules can be identified using co-expression network analysis. Protein dendrogram and module delimitation based on different clustering methods (see Materials & Method section). Modules group the proteins that have a high level of co-expression, based on pair-wise correlations between protein abundance. Four protein modules (indicated by colors: yellow, turquoise, blue or brown) were identified by 3 different clustering methods.

**Figure 3:**
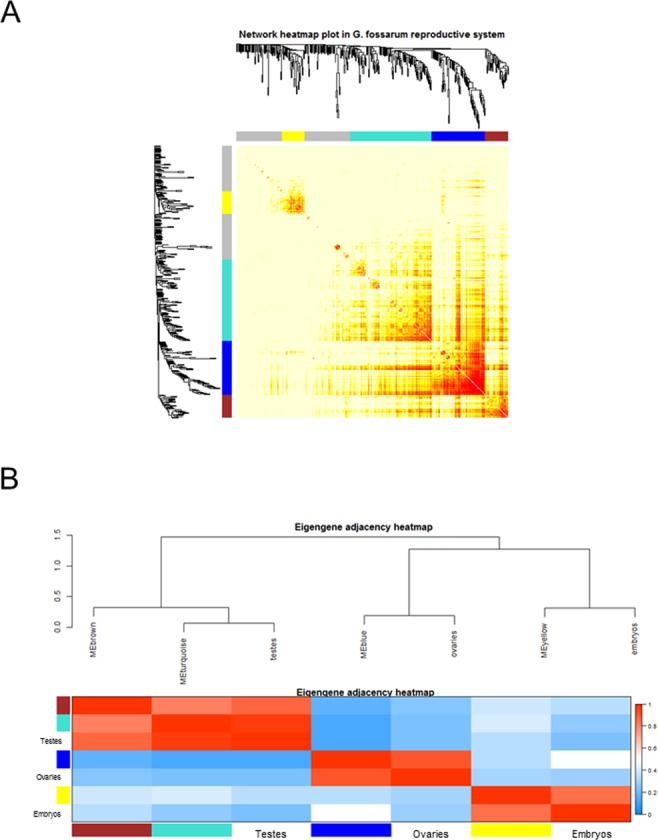
Distinct modules interact differently each other. (**A**) The heatmap shows the Topological Overlap Matrix (TOM) values among the proteins of the network delimited in modules by the dynamic method. Light yellow color represents low overlap (*i.e*. low interconnection), while dark red color represents high overlap (*i.e*. high interconnection). (**B**) The module eigengene adjacency showed by hierarchical clustering and heatmap. A module eigengene summarizes the protein expression profile of each module. Testes cluster together with the turquoise and brown module eigengenes, embryos with the yellow one and ovaries with the blue one.

We used four different clustering methods to explore the protein network of the gonads and embryos. Figure 2 shows the differences in terms of protein clustering between the four methods. The hybrid method (indicated as *blockwise Colors)*, the dynamic method and the mixed dynamic-hybrid method identified four distinct modules with high overlap among them. In order to minimize spurious associations induced by forcing proteins into proper modules but at the same time to maximize the sensitivity in detecting biologically relevant modules, the clustering performed by the dynamic method was retained for further analyses. On this basis, the four distinct modules were identified as a yellow module (31 proteins), a turquoise module (112 proteins), a blue module (74 proteins) and a brown module (32 proteins) (Fig. 2). The values of topological overlap among the proteins of the network showed that the yellow and the blue modules clustered distinctly each other and from the other modules (Fig. 3A). In order to quantify the similarity between modules and to see how the different tissues fit the eigengene network, we used the module eigengenes. Figure 3B shows the yellow module co-clustered exclusively with the embryonic tissues while the blue module clustered only with the ovaries. Interestingly, the two other modules (turquoise and brown) were close each other and co-clustered with testes (Fig. 3A,B). In this context, it was possible to associate each tissue or organ to one or two distinct co-expressed modules, independently from their maturation stage, identifying a previously unappreciated organization of the protein expression profiles in the reproductive tissues and embryonic development of *G. fossarum*^2,10^.

### Co-expressed modules mirrors gonad maturation and embryos developmental stages

**Figure 4:**
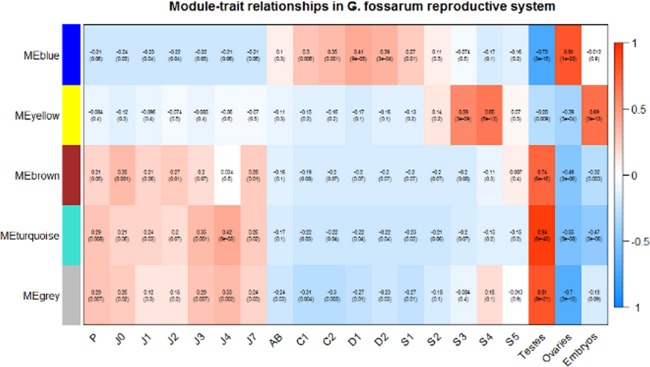
Testes, ovaries and embryos of *G. fossarum* are distinctively associated with different protein modules. Each row corresponds to a module eigengene, each column to a different developmental (for the embryos) and maturity (for the gonads) stages (on the left side) or different organ/tissue (on the right side). In each cell, the correlation value and the p-value (in parenthesis) for each module-stage association are shown.

By correlating the module eigengenes with each stage, we observed that two embryonic mid-developmental stages (S3 and S4) were the main drivers of the association between the yellow module and the embryonic traits, with positive correlation values of 0.59 and 0.66, respectively (p-values = 3*10^−9^ and 6*10^−12^ respectively) (Fig. 4). On the contrary, the earliest stage of embryonic development (S1) showed no correlation with the yellow module (−0.13, p-value = 0.2), while it was weakly but significantly correlated (0.27, p-value = 0.01) with the ovarian-associated module (blue), reflecting its cytoplasmic inheritance from the egg. Concerning the ovaries, all stages were positively correlated with the blue module, except the earliest stage (AB). All stages from C2 to D2 are mainly involved in vitellogenesis, indicating that the blue module contains mainly proteins involved in this physiological pathway. Moreover, the blue module was negatively correlated to (i.e. repressed in) testes (−0.73, p-value = 3*10^−15^), indicating that this pathway is strictly controlled in male gonads. Similarly, the brown and turquoise modules were highly correlated with the testes and significantly repressed in both embryos and ovaries (Fig. 4). Finally, even proteins that are not strongly co-expressed (grey module) were found to have an expression profile similar to the turquoise module, with high positive correlation to the testes and negative correlation to the ovaries. This is likely due to the choice of a conservative clustering in defining the co-expression modules. Indeed, a few proteins showed also a relatively high value of module membership (ME > 0.60) for the turquoise module (Supplementary Document 2) and could be probably involved in the same biological pathway activated in the testes and concomitantly repressed in the ovaries.

### Module protein composition and hub proteins provide new insights in the reproductive biology and embryonic developmental processes in *Gammarus fossarum*

**Figure 5:**
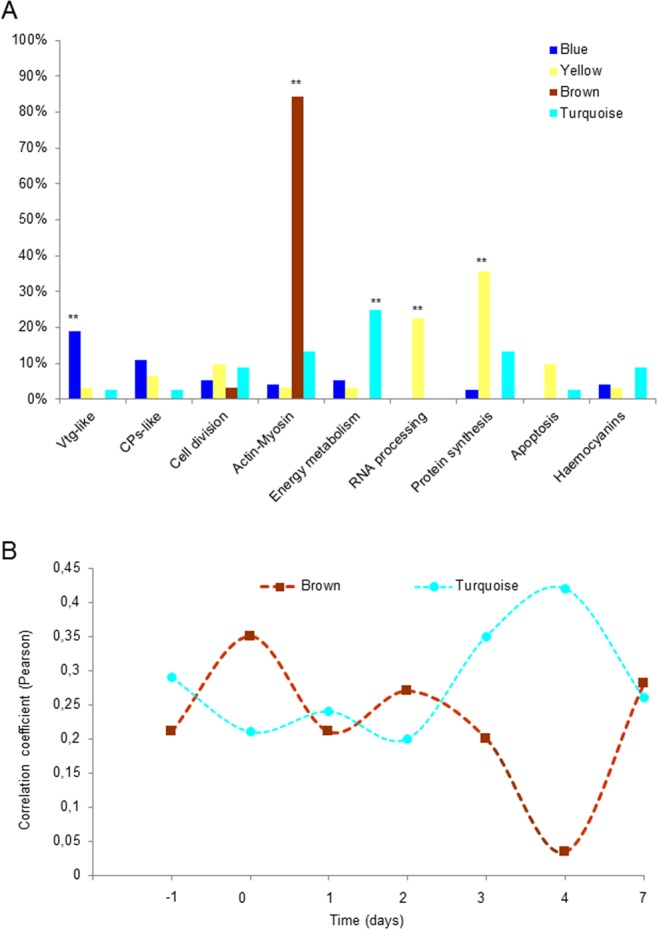
Different protein modules are differentially enriched in distinct molecular functions. (**A**) Percentage of modules’ proteins for each molecular function identified. **p-value < 0.01, Fisher’s exact-test. (**B**) Temporal variation of the correlation coefficient between brown module and turquoise module eigengenes and the phenotypic trait “testes”. The results show that the two modules are highly interconnected and their expression profiles regulated in a tightly coordinate manner.

We looked at the predicted biological role of the different proteins that clustered into the same co-expression module in order to investigate the biological significance of the identified co-expressed modules. We used the output of the NCBInr and Swissprot BLAST results to associate each protein to one category of the following molecular functions or protein families: the Large Lipid Transport Protein superfamily which included both Vitellogenin-like proteins (Vtg-like) and clottable protein-like (CPs-like), cell cycle and division, actin-myosin families, energy metabolism, RNA processing (including proteins regulating the transcriptional process, RNA splicing and mRNA transport to cytoplasm), protein synthesis (including riboproteins and factors involved in protein translation), apoptosis (including both potential activators and inhibitors), and haemocyanin-like proteins. The module correlated with the embryos (yellow) had a significant higher proportion of proteins involved in RNA processing (23%) and protein synthesis (35%) compared with the other modules (Table 1 and Fig. 5A, p-value < 0.01, Fisher’s exact test), suggesting an active cellular remodeling. Moreover, among the top five hub proteins (those showing the highest module membership value), contigs 121249_fr2 and 203833_fr2 were predicted to have functions involved in RNA editing and splicing regulation while 122312_fr4 is predicted to be involved in protein translation (Table 2, Supplementary Document 2). These results suggest the functional importance of RNA processing, gene isoform switch and protein translation in *G. fossarum* embryogenesis, particularly during the organogenesis (S3 and S4 stages).

**Table 1:** Number and percentage (in parentheses) of proteins belonging to the main molecular functions identified in the protein network. Comparison of relative abundance was made among the 4 co-expression modules. P-values were calculated with Fisher exact test.

Modules	Large Lipid Transport Proteins		Cell Division	Actin- Myosin	Energy metabolism	RNA processing^†^	Protein synthesis
	Vitellogenin-like	Clottable protein-like					
Blue	14 (19)**	8 (11)^‡^	4 (5)	3 (4)	4 (5)	0 (0)	2 (3)
Yellow	1 (3)	2 (6)	3 (10)	1 (3)	1 (3)	7 (23)**	11 (35)**
Brown	0 (0)	0 (0)	1 (3)	27 (84)**	0 (0)	0 (0)	0 (0)
Turquoise	3 (3)	3 (3)	10 (9)	15 (13)	28 (25)**	0 (0)	15 (13)

(**p-value < 0.01).

^†^We included in this category proteins involved in transcriptional and splicing regulation or mRNA transport.

^‡^p-value = 0.05025.

**Table 2:** Top hub proteins in the four co-expressed modules of the reproductive organs and embryos of *G. fossarum*.

Contig N	Module	Swissprot ACC	NCBInr ACC	E-value^†^	Organisms^††^	k_total_	k_within_	Module Membership
199971_fr3	Blue	Q94637	XP_018007759.1	1.40E-28	*H. atzeca*	15.340	13.757	0,97
276_fr4	Blue	O94518	XP_018025391.1	1.00E-10	*H. atzeca*	14.672	13.362	0.96
30649_fr2	Blue	Q868N5	AHK05984.1	9.00E-115	*G. marinus*	13.918	12.428	0.95
39606_fr3	Blue	—	XP_018007759.1	2.80E-96	*H. atzeca*	19.291	13.048	0.95
67263_fr2	Blue	Q17RH7	XP_017986259.1	0.049	*E. sinecaudum*	13.666	12.186	0.94
121249_fr2	Yellow	P51400	XP_018009377.1	2.60E-42	*H. atzeca*	2.409	2.259	0.89
203833_fr2	Yellow	P48810	XP_018010441.1	3.70E-97	*H. atzeca*	2.359	2.090	0.88
201834_fr4	Yellow	—	XP_018025391.1	2,00	*H. atzeca*	1.968	1.858	0.87
122312_fr4	Yellow	P49041	XP_018016935.1	5.40E-26	*H. atzeca*	2.079	1.912	0.87
153160_fr6	Yellow	P21895	XP_018020084.1	3.80E-45	*H. atzeca*	2.060	1.671	0.86
20975_fr4	Brown	Q24756	AFP95338.1	2,20E-36	*P. clarkii*	4.582	3.378	0.91
194796_fr2	Brown	P05661	BAK61430.1	1,40E-78	*M. japonicus*	6.384	3.384	0.90
37276_fr6	Brown	P05661	XP_018022402.1	1,70E-70	*H. atzeca*	9.565	3.522	0.90
182086_fr1	Brown	P05661	XP_018022403.1	7,60E-83	*H. atzeca*	3.602	3.019	0.89
48693_fr3	Brown	P30163	XP_020899190.1	1,50E-52	*E. pallida*	10.374	3.416	0.89
191065_fr3	Turquoise	P11979	XP_018006716.1	1,70E-32	*H. atzeca*	11.387	7.798	0.94
141775_fr4	Turquoise	Q5R2J2	CAQ60115.1	4,40E-118	*G. locusta*	10.926	6.974	0.89
34845_fr5	Turquoise	Q8JZW4	XP_018022776.1	4,80E-168	*H. atzeca*	10.154	6.587	0.89
2278_fr6	Turquoise	P07764	XP_018027036.1	5,00E-132	*H. atzeca*	12.427	6.770	0.89
16972_fr2	Turquoise	P35381	XP_018018775.1	3,00E-146	*H. atzeca*	8.411	5.042	0.88

^†^E-value < 0.001 were considered significant. Proteins with E-values > 0.001 should be considered without homologs.

^††^*H.: Hyalella; G.: Gammarus; E. sinecaudum: Eremothecium sinecaudum; P.: Procambarus; M.: Marsupenaeus; E. pallida: Exaiptasia pallida*.

The blue module correlated with the ovaries and in particular with the stages involved in secondary vitellogenesis. It presented a significant higher proportion of proteins annotated as vitellogenins or their precursors (19%) (Table 1, Fig. 5A, p-value < 0.01, Fisher’s exact test). Moreover, clottable protein-like proteins, another group of proteins belonging to the large lipid transport proteins (LLTP), showed a slight enrichment in this module compared with the others (11%, p-value = 0.05025, Fisher’s exact test). The functional importance of lipid transport in the ovaries was also confirmed using network statistics that showed the top four hub proteins belonged to either Vtg-like or clottable protein-like subfamilies (Table 2, Supplementary Document 2). It merits noting that LLTP proteins may arise from an ancient duplication event leading to paralogs of Vtg sequences and that the “clottable-like” annotation derives from neofunctionalization processes of proteins belonging to the group of metazoan Vtg^22^.

Interestingly, we found two taxonomically-restricted proteins in the yellow module and in the blue module (coded by the contigs 201834_fr4 and 67263_fr2) that showed high intramodular connectivity and module membership, characteristics of a hub role in their respective modules. These proteins do not show any significant homology (E-value > 0.001) with proteins currently available in the NCBInr and Swissprot databases. This result suggests that protein with key roles in the organogenesis and oocyte maturation may have evolved divergently in amphipods and it shows the strength of co-expression network analysis to get new molecular insights in non-model organisms.

Finally, the two modules correlated with the amphipod testes showed two distinct functional profiles. The brown module was highly enriched in proteins annotated as members of the actin or myosin families (84%, Table 1 and Fig. 5, Supplementary Document 1, p-value < 0.01, Fisher’s exact test). In particular, among the top five hub proteins, we found the top four to be annotated as myosin light (contig 20975_fr4) or heavy (194796_fr2, 37276_fr6, 182086_fr1) chains and the fifth to be an actin isoform (48693_fr3) (Table 2, Supplementary Document 2). The turquoise module presented instead a significant higher proportion of proteins involved in the energy metabolism, in particular in the glycolysis pathway and ATP synthesis (25%, Table 1 and Fig. 5, Supplementary Document 2, p-value < 0.01, Fisher’s exact test). The contig 191065_fr3 that codes for the rate limiting enzyme pyruvate kinase was the top hub protein in the module. Other two enzymes involved in the glycolytic pathway (a putative GAPDH and a predicted fructose aldolase, coded by the contigs 141775_fr4 and 2278_fr6, respectively) were also found among the top five hub proteins (Table 2, Supplementary Document 2). Finally, we observed that the values of correlation coefficients between the eigengenes of the turquoise and brown modules and the post-copula times showed opposite trends, with the maximum difference at day 4 (Fig. 5B). It is also of interest noting that the peak in the brown module is at day 0 of copulation, suggesting a contribution of the muscle contraction of the gonad during fertilization. These results suggest that the two pathways interact in a coordinated manner during spermatogenesis in *G. fossarum* testes.

## Discussion

In this study, we used for the first time a co-expression network analysis to investigate the protein organization of the reproductive organs and embryos in the freshwater amphipod *G. fossarum* using shotgun proteomics data. We showed that WGCNA was well suited to extract biological information using shotgun proteomics data from a non-model organism. In our case study that considered contrasted biological samples at different maturation or developmental stages, this methodology allowed us to identify different modules of co-expressed proteins correlated with the spermatogenesis cycle, and some specific physiological stage of ovary maturation or embryonic development, shedding new light on the molecular physiology of the reproductive system in *G. fossarum*. We found one module (named as yellow) correlated with embryos, notably with the mid-developmental stages S3 and S4. A second module (named as blue) correlated with the ovaries, notably with stages from C1 to D2; and finally the two other modules (namely the brown and the turquoise) both correlated with the testes, with the trends in the correlation coefficients suggesting these two pathways are mutually controlled during the amphipod spermatogenesis. Compared with our previous studies^2,10^, we were able to identify key proteins involved in physiological pathways such as oocyte maturation, spermatogenesis and embryonic development without making use of *a priori* protein function predicted by standard sequence similarity search, but by using hierarchical clustering to construct modules from protein abundance data obtained by label-free proteomics. We showed for the first time that embryogenesis in a non-model species, namely *G. fossarum*, is characterized by the activation of factors involved in RNA processing, such as RNA editing or RNA splicing control. Interestingly, mechanisms involved in the regulation of alternative splicing have been identified in evolutionarily distant metazoans, such as the fruit fly and the mouse which are well characterized models^23,24^. Our data-driven approach enabled us to identify two proteins with hub properties, 201834_fr4 in the yellow module and 67263_fr2 in the blue module, that had no significant homology among the NCBInr and Swissprot databases by sequence similarity search (E-value < 0.001). This result highlights the potential of co-expression network analyses in identifying taxon-restricted proteins with key roles in molecular physiology processes. Moreover, the observation that one of these proteins is involved in organogenesis is in line with the recent proposal, based on the comparison of the developmental transcriptomes of 10 different species, of a divergent mid-development transition that uses species-specific functions in embryo development for defining the phyletic body plan^25^. This result is coherent with the observation that S1 stage corresponds to a 2 cell-stage embryos and most of their proteomic profiles are inherited by the maternal egg, while stage S3 and S4 are mainly involved in the organogenesis^10,14^.

Our network analysis found evidence of two different pathways involved in testicular processes, namely the glycolytic pathways and the actin-myosin system in *G. fossarum*. While testicular metabolism is scarcely investigated in arthropods, an early biochemical study reported that glycolysis played an important energetic role in both early and late spermatogenesis in *Drosophila hydei*^26^. However, glycolysis has been reported as a key pathway in the spermatogenesis of different vertebrate species. Notably, glycolytic enzymes have been found in the carp seminal plasma using mass spectrometry^27^. A gene expression screening across human, mouse, and rat testes found a functional enrichment in genes involved in glycolysis and pyruvate metabolism^28^. Moreover, a study suggested that mouse spermatogonial stem cells might be primed for conditions that favor glycolytic activity, a bioenergetics state that helps to maintain their functional integrity^29^. Interestingly, we previously reported the contig 40028_fr4 (annotated as a glycogen phosphorylase) among the proteins altered in the testes of male gammarids exposed to pyriproxyfen, suggesting that this pathway might be sensitive to chemical exposures in this amphipod species^30^.

Actins and myosins play also an important role in spermatogenesis^31^. Myosins belong to a large superfamily of molecular motors and they are involved in different processes during spermatogenesis, such as acrosomal formation or spermatid individualization^32^. Actin is responsible for the formation of specific sub-cellular structures in arthropods^31^. In *Drosophila*, actin forms apical bundles required for spermatid individualization. Moreover, a new cytoskeletal structure composed of actin and microtubules, namely the acroframosome, has been recently described in the crustacean *Macrobrachium nipponense*^33^. Together, these studies suggest the importance of actin and myosin interactions and the glycolytic pathway as the energy fuel during spermatogenesis in metazoans, including crustaceans, reinforcing our results that showed that these interactions are distinctly activated in *G. fossarum* testes.

Finally, the co-expression analysis performed in this study confirmed and expanded our previous results that showed a high diversity of proteins belonging to the LLTP superfamily involved in yolk formation^10^. We found strong co-expression of 22 contigs annotated as Vtg-like or clottable protein like proteins in the ovaries, expanding the previous set of 8 contigs. While multiple copies of Vtg genes were reported in many arthropods^34,35^, it might be possible that some of the contigs represent a fragmented reconstruction of the original mRNA, giving an overestimation of the total number of vitellogenins or clottable proteins in *G. fossarum*. Our co-expression correlations among different Vtg-like contigs might help annotation efforts and improve *de novo* transcriptome assemblies, likely providing a better estimation of the number of LLTP proteins in this freshwater amphipod.

In conclusion, in this case study we performed the first co-expression network analysis on the proteome of male, female and embryos of *G. fossarum*, an emergent model species in molecular ecotoxicology. We found evidence of the importance of unappreciated molecular pathways involved in the amphipod embryogenesis, notably RNA splicing, and confirmed the diversity of proteins belonging to the large lipid transfer family. Moreover, we were able to identify proteins with a hub role in embryogenesis and vitellogenesis that do not have any close homologs in sequenced animal genomes. This result shows the strength of co-expression network methods in generating working hypothesis on specific proteins that standard homology comparisons or differential expression analysis would have failed to identify. Finally, our results found evidence of a fine-tuned regulation between energy production and myosin-dependent events in *G. fossarum* spermatogenesis. This study illustrates the relevance of applying systems biology approaches to emergent animal models to improve the understanding of the molecular mechanisms of physiological events with high ecological relevance.

Network analyses are promising but still not fully exploited approaches for the understanding of the molecular physiology of sentinel organisms in ecotoxicology. These tools will help to link adverse outcomes to gene or protein modules, informing the Adverse Outcome Pathway framework with the underlying molecular mechanisms of toxicity.

## Supplementary information

Supplementary Document 1

Supplementary Document 2

Dataset 1

Dataset 2

## Data Availability

Original mass spectrometry data are available via the PRIDE repository with the dataset identifier PXD000576 and PXD001002. Spectral count data are directly available in Supplementary Data 1.
