# Supplementary material for: Co-expression network analysis identifies gonad- and embryo-associated protein modules in the sentinel species Gammarus fossarum
Source: Sci Rep. 2019 May 27;9:7862. doi: 10.1038/s41598-019-44203-5 (PMC6536538; doi:10.1038/s41598-019-44203-5)
Supplement: Supplementary file 1 — Supplementary Document 1 [file 41598_2019_44203_MOESM1_ESM.docx]

Co-expression network analysis identifies gonad- and embryo-associated protein modules in the sentinel species *Gammarus fossarum*.

Davide Degli Esposti^1^, Christine Almunia^2^, Marc-Antoine Guery^1^, Natacha Koenig^1^, Jean Armengaud^2^, Arnaud Chaumot^1^, Olivier Geffard^1^

# 30 January 2019

# Network analysis of label-free LC-MS Proteomics data of reproductive system in G. fossarum

#################################################

# Index. Summary of the pipeline

#################################################

# 1. LOADING

# 2. FILTERING LOW ABUNDANCE PROTEINS AND PRE-PROCESSING

# 3. DATA NORMALIZATION

# 4. EXPLORATORY DATA ANALYSIS

# 5. NETWORK CONSTRUCTION AND MODULE DETECTION

# 6. RELATING MODULES TO TRAITS

# 7. HUB GENES IN THE MODULES

# 8. VISUALIZATION OF THE NETWORK

################################################

# Required packages

#tools

library(plyr)

library(ggplot2)

library(grid)

library(gridExtra)

#PCA and clustering

library(mixOmics)

library(minfi)

library(lumi)

library(stats)

# Networks

library(WGCNA)

#If bug on blockwise function (namespace conflict)

cor <- WGCNA::cor

# The following setting is important, do not omit.

options(stringsAsFactors = FALSE)

allowWGCNAThreads()

# Generics

library(limma)

library(edgeR)

library(made4)

library(RColorBrewer)

###########################################################

setwd("/Users/davide.degli-esposti/Desktop/R/repro")

dir()

getwd()

###################################################################################

# INTRODUCTORY NOTE

####################################################################################

# In this script I will reproduce all the analytical steps used to produce

# the results on the network analysis on the reproductive tssues of Gammarus fossarum

####################################################################################

# 1. LOADING

#################################################

rawdata <- read.delim("repro_testis-oocytes-embryo_specCounts.txt",

check.names=FALSE, stringsAsFactors=FALSE)

# Taking a look to the raw data is always a good idea

names(rawdata)

row.names(rawdata)

View(rawdata)

# read the phenotype data (descriptors, i.e.: exposure, class, batch effects)

pData <- read.delim("repro_stage_pdata.txt")

colnames(pData)

View(pData)

#######################################################

# 3. FILTERING LOW ABUNDANCE PROTEINS OR MISSING VALUES

#######################################################

# PRE-FILTERING STEP

# Put the data into a DGEList object (edgeR):

y <- DGEList(counts=rawdata[1:85], genes=rownames(rawdata))

dim(y) # this object is still keeping proteins wit too many NAs. In the next step we will filter

y$samples

y$genes

save(y, file="y_repro_13-7-18.RData")

#Look at the data: Euclidian clustering and PCA.

# Euclidian clustering

plotSampleRelation(y$counts, subset = 2000, method = "cluster",labels=pData$group, cex=0.7)

# WE HAVE AN OUTLIER: AB-n1

abline(h=26.5, col="red")

#PCA

ty<-as.data.frame(t(y$counts)) #transpose count dataframe

tune.pca(ty, ncomp=10, center=T, scale=F) #explore how many dimensions explain variability #here 2

pca =pca(ty, ncomp=2)

#plotting results

colSeqRepro<-c(colorRampPalette(c("lightblue", "darkblue")) (5),

colorRampPalette(c("pink", "darkred")) (7),

colorRampPalette(c("lightgreen", "darkgreen")) (5))

plotIndiv(pca,

group = pData$group ,

col.per.group = colSeqRepro,

ind.names = F,

legend=T, legend.position="right", legend.title=NULL,

size.xlabel = 15,

size.ylabel = 15,

size.legend = 15,

size.axis = 15,

size.title = 15,

title= paste('PCA rawdata'),

comp=c(1,2),

ellipse=F,

pch=16)

#FILTERING STEP: EXLUDE OUTLIER + FILTER OUT LOWLY EXPRESSED PROTEINS

# (recommended for networking analyses)

# We seek proteins that achieve at least spectral count n>3 in 5 samples)

# Exclude AB-n1

rawdataF<-rawdata[,-36]

dim(rawdata)

dim(rawdataF)

View(rawdataF)

pDataF<-pData[-36,]

dim(pDataF)

# Define a threshold corresponding to a count of 3 spectral counts

sc<-3

th<-cpm(sc, mean(colSums(rawdataF)))[1]

round(th) #2779

nbsamples<-5

keep<-rowSums(cpm(rawdataF) > th) >=nbsamples

rawdataF<-rawdataF[keep,]

dim(rawdataF)

#[1] 375 84

######################################################

# 4.DATA NORMALIZATION

######################################################

#In this analysis, we normalized using the function calcNormfactors of the edgeR package

# based on a TMM (Trimmed mean of M-values) normalization procedure

#(https://genomebiology.biomedcentral.com/articles/10.1186/gb-2010-11-3-r25)

#Put the data into a DGEList object

yF<-DGEList(counts=rawdataF,

genes=row.names(rawdataF),

group= pDataF$group)

dim(yF)

# normalization (required for WGCNA)

yN <- calcNormFactors(yF)

yN$samples

yN

save(yN,file="yN_repro_13-7-18.RData")

dim(yN)

#Create the data with counts multiplied for norm.factors

yN2<-yN

n<-length(colnames(yN$counts))

n

for (i in seq (from=1, to=n)) {

yN2$counts[,i]<-yN$counts[,i]*yN$samples$norm.factors[i]

};

dim(yN2)

head(yN2$genes)

data<-yN2$counts

rownames(data)<-yN2$genes$genes

rownames(data)

head(data)

head(yN$counts)

head(yN2$counts)

save(yN2,data,pDataF, file = "yN2_repro_13-7-18.RData")

#Look at the effect of filtering and normalization on the data: Euclidian clustering and PCA.

# Euclidian clustering

plotSampleRelation(yN2$counts, subset = 500, method = "cluster",labels=pDataF$group, cex=0.7)

# NO MORE OUTLIERS

#PCA

tyN2<-as.data.frame(t(yN2$counts)) #transpose count dataframe

tune.pca(tyN2, ncomp=10, center=T, scale=F) #explore how many dimensions explain variability #here 2

pca =pca(tyN2, ncomp=2)

#plotting results

colSeqRepro<-c(colorRampPalette(c("lightblue", "darkblue")) (5),

colorRampPalette(c("pink", "darkred")) (7),

colorRampPalette(c("lightgreen", "darkgreen")) (5))

plotIndiv(pca,

group = pDataF$group ,

col.per.group = colSeqRepro,

ind.names = F,

legend=T, legend.position="right", legend.title=NULL,

size.xlabel = 15,

size.ylabel = 15,

size.legend = 15,

size.axis = 15,

size.title = 15,

title= paste('PCA filtered and normalized data'),

comp=c(1,2),

ellipse=F,

pch=16)

#################################################

# 5. NETWORK CONSTRUCTION AND MODULE DETECTION

#################################################

source("http://bioconductor.org/biocLite.R")

biocLite(c("AnnotationDbi", "impute", "GO.db", "preprocessCore"))

#Aim: to build the network based on proteines expressed over the previously established threshold

#Create a eset object to use for choosing the n. of genes to study with WGCNA.

library(Biobase)

esetpData<-as.data.frame(pDataF)

esetpData<-AnnotatedDataFrame(esetpData)

sampleNames(esetpData)=row.names(pDataF)

eset<-ExpressionSet(data,esetpData)

eset

save(eset, file = "repro_eset_13-7-18.RData")

#Transpose the table with expression values. Here we use the whole protein dataset after the

# filtering step we performed above

data <- t(exprs(eset))

dim(data)

#Model the data to approach a scale-free topology network for picking a soft-threshold.

# Choose a set of soft-thresholding powers

powers = c(c(1:12))

powers

# Call the network topology analysis function.

# From now, we have to work with the transposed table for expression values.

sft = pickSoftThreshold(data, powerVector = powers, verbose = 6)

# Plot the results:

sizeGrWindow(9, 5)

par(mfrow = c(1,2));

cex1 = 0.9;

# Scale-free topology fit index as a function of the soft-thresholding power

plot(sft$fitIndices[,1], -sign(sft$fitIndices[,3])*sft$fitIndices[,2],

xlab="Soft Threshold (power)",ylab="Scale Free Topology Model Fit,signed R^2",type="n",

main = paste("Scale independence"));

text(sft$fitIndices[,1], -sign(sft$fitIndices[,3])*sft$fitIndices[,2],

labels=powers,cex=cex1,col="red");

# this line corresponds to using an R^2 cut-off of h

abline(h=0.9,col="red")

# Mean connectivity as a function of the soft-thresholding power

plot(sft$fitIndices[,1], sft$fitIndices[,6],

xlab="Soft Threshold (power)",ylab="Mean Connectivity", type="n",

main = paste("Mean connectivity"))

text(sft$fitIndices[,1], sft$fitIndices[,6], labels=powers, cex=cex1,col="red")

# One-step network construction and module detection

# Block-wise using all data

net = blockwiseModules(data, power = 7, maxBlockSize=500,

networkType="unsigned", TOMType = "signed", minModuleSize = 25,

reassignThreshold = 0, mergeCutHeight = 0.25,

numericLabels = TRUE, pamRespectsDendro = FALSE,

saveTOMs = TRUE, deepSplit = 2,

saveTOMFileBase = "repro_net_13-718",

verbose = 3)

#....removing 1 genes from module 1 because their KME is too low.

#....removing 1 genes from module 1 because their KME is too low.

net # take a look at what looks like

save(net,file="repro_net_13-7-18.RData")

table(net$colors)

table(net$unmergedColors)

head(net$MEs)

net$goodSamples

table(net$goodGenes)

net$dendrograms

net$TOMFiles

table(net$blocks)

net$MEsOK

# Convert labels to colors for plotting

moduleColors = labels2colors(net$colors)

table(moduleColors)

#moduleColors

moduleColors

#moduleColors

# blue brown grey turquoise yellow

# 103 44 66 121 41

# Plot the dendrogram and the module colors underneath (1 block)

sizeGrWindow(10,5)

plotDendroAndColors(net$dendrograms[[1]], moduleColors,

"Modules",

main = "Protein dendrogram and module definition in G. fossarum reproductive system",

dendroLabels = FALSE, hang = 0.03,

addGuide = TRUE, guideHang = 0.05)

# save the data

moduleLabels = net$colors

moduleColors = labels2colors(net$colors)

MEs = net$MEs;

geneTree = net$dendrograms[[1]];

save(MEs, moduleLabels, moduleColors, geneTree,data, pData, eset,

file = "repro13-7-18_network-auto.RData")

# Module definition using adjacency measures

# Refine the gene dendrogram, retrieving highly connected intramodular hub genes

# Test different height cut-off threshold to assess how rebust the findings are.

# Test different branch cutting methods

# Calculate and store the adjacencies in an object, using the soft thresholding power 7

softPower = 7

adjacency = adjacency(data, power = softPower)

# transform adjacency into Topologiacal Overlap Matrix and calculate the corresponding dissimilarity

TOM=TOMsimilarity(adjacency)

dissTOM=1-TOM

# 1. Static method

cutreeStaticColor(geneTree, cutHeight = 0.99, minSize=25)

colorStaticADJ=as.character(cutreeStaticColor(geneTree, cutHeight = 0.99, minSize=25))

#2. tree (Dynamic) method

branch.number=cutreeDynamic(geneTree, method="tree")

colorDynamicADJ=labels2colors(branch.number)

#3. Hybrid method (hclust and pam). To perform this method we need both the dendrogram and the dissimilarity matrix

colorDynHybrADJ= labels2colors(cutreeDynamic(geneTree, distM=dissTOM, cutHeight = 0.998, deepSplit = 2, pamRespectsDendro = FALSE))

#Plot the results o all module detection methods together:

plotDendroAndColors(geneTree, colors = data.frame (moduleColors, colorStaticADJ,

colorDynamicADJ, colorDynHybrADJ),

main = "Protein dendrogram & module definitions in G. fossarum reproductive sytem",

dendroLabels = FALSE)

# Module definition using TOM dissimilarity hierarchical clustering

# calculate the dendrogram

hierTOM = hclust(as.dist(dissTOM), method="average")

colorStaticTOM = as.character(cutreeStaticColor(hierTOM, cutHeight=.99, minSize=25))

colorDynamicTOM = labels2colors (cutreeDynamic(hierTOM,method="tree"))

colorDynamicHybridTOM = labels2colors(cutreeDynamic(hierTOM, distM= dissTOM , cutHeight = 0.998,

deepSplit=2, pamRespectsDendro = FALSE))

# Now we plot the results

sizeGrWindow(10,5)

plotDendroAndColors(hierTOM,

colors=data.frame(moduleColors, colorStaticTOM,

colorDynamicTOM, colorDynamicHybridTOM),

dendroLabels = FALSE,

main = "Protein dendrogram & module definitions in G. fossarum reproductive sytem")

#compare different module outcoume

table(moduleColors)

table(colorStaticTOM)

table(colorDynamicTOM)

table(colorDynamicHybridTOM)

table(colorStaticADJ)

table(colorDynamicADJ)

table(colorDynHybrADJ)

#moduleColors

#blue brown grey turquoise yellow

#103 44 66 121 41

#> table(colorStaticTOM)

#colorStaticTOM

#blue grey turquoise

#41 84 250

#> table(colorDynamicTOM)

#colorDynamicTOM

#blue brown grey turquoise

#95 41 84 155

#> table(colorDynamicHybridTOM)

#colorDynamicHybridTOM

##blue brown grey turquoise yellow

#123 53 17 137 45 #

#> table(colorStaticADJ)

#colorStaticADJ

#blue grey turquoise

#31 126 218

#> table(colorDynamicADJ)

#colorDynamicADJ

#blue brown grey turquoise yellow

#74 32 126 112 31

#> table(colorDynHybrADJ)

#colorDynHybrADJ

#blue brown green grey turquoise yellow

#116 52 25 14 124 44

# Save the network showing good sensitivity and specificity, and relatively stringency in detecting genes with high MM

save(MEs, moduleLabels, colorDynamicADJ, geneTree, data, pData, eset,

file = "repro_13-7-18_DynamicADJ.RData")

# Extract the genes from each module of the chosen network

table(colorDynamicADJ)

genes.blue<-colnames(data)[colorDynamicADJ=="blue"] #1

length(genes.blue)

head(genes.blue)

genes.brown<-colnames(data)[colorDynamicADJ=="brown"] #2

length(genes.brown)

genes.turquoise<-colnames(data)[colorDynamicADJ=="turquoise"] #3

length(genes.turquoise)

genes.yellow<-colnames(data)[colorDynamicADJ=="yellow"] #4

length(genes.yellow)

genes.grey<-colnames(data)[colorDynamicADJ=="grey"] #0

length(genes.grey)

# write the tables

write.csv(genes.blue,"genes.blue_repro_13-7-18.csv")

write.csv(genes.brown,"genes.brown_repro_13-7-18.csv")

write.csv(genes.turquoise,"genes.turquoise_repro_13-7-18.csv")

write.csv(genes.yellow,"genes.yellow_repro_13-7-18.csv")

write.csv(genes.grey,"genes.grey_repro_13-7-18.csv")

#################################################

# 6. RELATING MODULES TO TRAITS

#################################################

# Load network data saved previously.

lnames = load(file = "repro_13-7-18_DynamicADJ.RData")

lnames

# Define numbers of genes and samples

nGenes = ncol(data)

nGenes

nSamples = nrow(data)

nSamples

dim(pDataF)

View(pDataF)

pData2<-pDataF[,-c(18,19,20,21)]

dim(pData2)

View(pData2)

# Recalculate MEs with color labels

MEs0 = moduleEigengenes(data, colorDynamicADJ)$eigengenes

MEs0

MEs = orderMEs(MEs0)

moduleTraitCor = cor(MEs, pData2)

moduleTraitPvalue = corPvalueStudent(moduleTraitCor, nSamples);

# graphical representation will help in reading the table.

# We color code each association by the correlation value:

sizeGrWindow(10,6)

# Will display correlations and their p-values

textMatrix = paste(signif(moduleTraitCor, 2), "\n(",

signif(moduleTraitPvalue, 1), ")", sep = "");

dim(textMatrix) = dim(moduleTraitCor)

par(mar = c(6, 8.5, 3, 3));

# Display the correlation values within a heatmap plot

labeledHeatmap(Matrix = moduleTraitCor,

xLabels = names(pData2),

yLabels = names(MEs),

ySymbols = names(MEs),

colorLabels = FALSE,

colors = blueWhiteRed(50),

textMatrix = textMatrix,

setStdMargins = FALSE,

cex.text = 0.5,

zlim = c(-1,1),

main = paste("Module-trait relationships in G. fossarum reproductive system"))

#################################################

# 7. HUB GENES IN THE MODULES

#################################################

#Calculate intramodular connectivity for each gene

ADJ1=abs(cor(data, use ="p"))^7

Alldegrees1=intramodularConnectivity(ADJ1, colorDynamicADJ)

head(Alldegrees1)

write.csv(Alldegrees1, "repro_13-7-18_intramodularConnectivity_DynADJ.csv")

geneModuleMembership=as.data.frame(cor(data, MEs, use = "p"))

geneModuleMembership

write.csv(geneModuleMembership,"repro_13-7-18_geneModuleMembership_DynADJ.csv")

MMPvalue=as.data.frame(corPvalueStudent(as.matrix(geneModuleMembership), nSamples))

MMPvalue

write.csv(MMPvalue,"repro_13-7-18_MMPvalue_DynADJ.csv")

#MDS plots to show proteins in modules with hub genes in the finger tips

cmd1=cmdscale(as.dist(dissTOM),2)

sizeGrWindow(7,6)

par(mfrow=c(1,1))

plot(cmd1, col=as.character(colorDynamicADJ), main="MDS plot of network modules in G fossarum reproductive system",

xlab="Scaling Dimension 1", ylab="Scaling Dimension 2")

#################################################

# 8. VISUALIZATION OF THE NETWORK

#################################################

#Visualizing the gene network

#Load network data saved in the second part.

lnames = load(file = "repro_13-7-18_DynamicADJ.RData");

lnames

#The variable lnames contains the names of loaded variables.

nGenes = ncol(data)

nSamples = nrow(data)

# Load the topological overlap (TOM) for each block

dissTOM = 1-TOMsimilarityFromExpr(data, power = 7);

# Transform dissTOM with a power to make moderately strong connections more visible in the heatmap

plotTOM = dissTOM^12;

# Set diagonal to NA also improves the clarity of the plot

diag(plotTOM) = NA;

# Call the plot function

sizeGrWindow(9,9)

TOMplot(plotTOM, geneTree, colorDynamicADJ,

main = "Network heatmap plot in G. fossarum reproductive system")

#Visualizing Eigengene relationships

# Recalculate module eigengenes

MEs = moduleEigengenes(data, colorDynamicADJ)$eigengenes

MEs

# Plot the relationships among the eigengenes of the different modules (dendrogram+hm)

sizeGrWindow(5,7.5);

par(cex = 0.9)

plotEigengeneNetworks(MEs, "Eigengene adjacency heatmap", marDendro = c(0,4,1,2),

marHeatmap = c(3,4,1,2), cex.lab = 0.8,

xLabelsAngle= 90)

#####################################################

sessionInfo()

######################################################

R version 3.5.2 (2018-12-20)

Platform: x86_64-w64-mingw32/x64 (64-bit)

Running under: Windows >= 8 x64 (build 9200)

Matrix products: default

locale:

[1] LC_COLLATE=French_France.1252 LC_CTYPE=French_France.1252

[3] LC_MONETARY=French_France.1252 LC_NUMERIC=C

[5] LC_TIME=French_France.1252

attached base packages:

[1] grid parallel stats4 stats graphics grDevices utils

[8] datasets methods base

other attached packages:

[1] edgeR_3.24.3 made4_1.56.0

[3] scatterplot3d_0.3-41 gplots_3.0.1.1

[5] ade4_1.7-13 limma_3.38.3

[7] lumi_2.34.0 minfi_1.28.3

[9] bumphunter_1.24.5 locfit_1.5-9.1

[11] iterators_1.0.10 foreach_1.4.4

[13] Biostrings_2.50.2 XVector_0.22.0

[15] SummarizedExperiment_1.12.0 DelayedArray_0.8.0

[17] BiocParallel_1.16.5 matrixStats_0.54.0

[19] GenomicRanges_1.34.0 GenomeInfoDb_1.18.1

[21] gridExtra_2.3 plyr_1.8.4

[23] RColorBrewer_1.1-2 mixOmics_6.6.1

[25] ggplot2_3.1.0 lattice_0.20-38

[27] MASS_7.3-51.1 WGCNA_1.66

[29] GO.db_3.7.0 AnnotationDbi_1.44.0

[31] IRanges_2.16.0 S4Vectors_0.20.1

[33] Biobase_2.42.0 BiocGenerics_0.28.0

[35] fastcluster_1.1.25 dynamicTreeCut_1.63-1
