# Supplementary material for: Co-expression network analysis identifies gonad- and embryo-associated protein modules in the sentinel species Gammarus fossarum
Source: Sci Rep. 2019 May 27;9:7862. doi: 10.1038/s41598-019-44203-5 (PMC6536538; doi:10.1038/s41598-019-44203-5)
Supplement: Supplementary file 3 — Dataset 1 [file 41598_2019_44203_MOESM3_ESM.docx]

Co-expression network analysis identifies gonad- and embryo-associated protein modules in the sentinel species *Gammarus fossarum*.

Davide Degli Esposti^1^, Christine Almunia^2^, Marc-Antoine Guery^1^, Natacha Koenig^1^, Jean Armengaud^2^, Arnaud Chaumot^1^, Olivier Geffard^1^

"X25293947testis.P.n1" "X25293947testis.P.n2" "X25293947testis.P.n3" "X25293947testis.P.n4" "X25293947testis.P.n5" "X25293947testis.J0.n1" "X25293947testis.J0.n2" "X25293947testis.J0.n3" "X25293947testis.J0.n4" "X25293947testis.J0.n5" "X25293947testis.J1.n1" "X25293947testis.J1.n2" "X25293947testis.J1.n3" "X25293947testis.J1.n4" "X25293947testis.J1.n5" "X25293947testis.J2.n1" "X25293947testis.J2.n2" "X25293947testis.J2.n3" "X25293947testis.J2.n4" "X25293947testis.J2.n5" "X25293947testis.J3.n1" "X25293947testis.J3.n2" "X25293947testis.J3.n3" "X25293947testis.J3.n4" "X25293947testis.J3.n5" "X25293947testis.J4.n1" "X25293947testis.J4.n2" "X25293947testis.J4.n3" "X25293947testis.J4.n4" "X25293947testis.J4.n5" "X25293947testis.J7.n1" "X25293947testis.J7.n2" "X25293947testis.J7.n3" "X25293947testis.J7.n4" "X25293947testis.J7.n5" "X27404005oocytes.AB.n1" "X27404005oocytes.AB.n2" "X27404005oocytes.AB.n3" "X27404005oocytes.AB.n4" "X27404005oocytes.AB.n5" "X27404005oocytes.C1.n1" "X27404005oocytes.C1.n2" "X27404005oocytes.C1.n3" "X27404005oocytes.C1.n4" "X27404005oocytes.C1.n5" "X27404005oocytes.C2.n1" "X27404005oocytes.C2.n2" "X27404005oocytes.C2.n3" "X27404005oocytes.C2.n4" "X27404005oocytes.C2.n5" "X27404005oocytes.D1.n1" "X27404005oocytes.D1.n2" "X27404005oocytes.D1.n3" "X27404005oocytes.D1.n4" "X27404005oocytes.D1.n5" "X27404005oocytes.D2.n1" "X27404005oocytes.D2.n2" "X27404005oocytes.D2.n3" "X27404005oocytes.D2.n4" "X27404005oocytes.D2.n5" "X27404005embryo.S1.n1" "X27404005embryo.S1.n2" "X27404005embryo.S1.n3" "X27404005embryo.S1.n4" "X27404005embryo.S1.n5" "X27404005embryo.S2.n1" "X27404005embryo.S2.n2" "X27404005embryo.S2.n3" "X27404005embryo.S2.n4" "X27404005embryo.S2.n5" "X27404005embryo.S3.n1" "X27404005embryo.S3.n2" "X27404005embryo.S3.n3" "X27404005embryo.S3.n4" "X27404005embryo.S3.n5" "X27404005embryo.S4.n1" "X27404005embryo.S4.n2" "X27404005embryo.S4.n3" "X27404005embryo.S4.n4" "X27404005embryo.S4.n5" "X27404005embryo.S5.n1" "X27404005embryo.S5.n2" "X27404005embryo.S5.n3" "X27404005embryo.S5.n4" "X27404005embryo.S5.n5"

"10004_fr1" 0 1 0 1 0 0 0 0 0 0 0 1 0 0 0 0 0 0 1 0 0 0 0 2 0 0 0 0 3 3 1 2 0 0 2 0 0 0 0 0 0 0 0 0 0 0 0 0 0 0 0 0 0 0 0 0 0 0 0 0 0 0 0 0 0 0 0 0 0 0 0 0 0 0 0 0 0 0 0 0 0 0 0 0 0

"100102_fr1" 0 0 0 0 0 0 0 0 0 0 0 0 0 0 0 0 0 0 0 0 0 0 0 0 0 0 0 0 2 0 0 0 0 0 0 0 0 0 0 0 0 0 0 0 0 0 0 0 0 0 0 0 0 0 0 0 0 0 0 0 0 0 0 0 0 0 0 0 0 0 0 0 0 0 0 0 0 0 0 0 0 0 0 0 0

"100307_fr3" 0 0 0 0 0 0 0 0 0 0 0 0 0 0 0 0 0 0 0 0 0 0 0 0 0 0 0 0 0 0 0 0 0 0 0 0 0 0 0 0 0 0 0 0 0 0 0 0 0 0 0 0 0 0 0 0 0 0 0 0 0 0 0 0 0 1 0 1 0 0 0 0 0 0 0 0 0 0 0 0 0 0 0 0 0

"100349_fr3" 11 20 10 6 12 6 24 18 13 10 6 10 23 20 16 23 12 19 19 12 16 13 16 14 14 24 10 10 15 10 22 15 10 15 6 8 3 7 4 7 1 0 1 6 2 1 3 3 4 0 1 4 1 0 1 3 3 3 0 0 5 3 2 2 7 14 16 15 16 10 19 18 19 19 18 22 21 20 27 15 18 14 18 17 16

"100405_fr3" 0 0 1 0 0 0 0 0 0 0 0 0 0 0 0 3 0 0 2 2 3 1 1 1 0 0 2 0 2 1 2 0 1 0 0 0 0 0 0 0 0 0 0 0 0 0 0 0 0 0 0 0 0 0 0 0 0 0 0 0 0 0 0 0 0 0 0 0 0 0 0 0 0 0 0 0 0 0 0 0 0 0 0 0 0

"10101_fr5" 0 0 4 0 2 0 0 0 0 0 0 0 0 0 0 0 1 0 2 0 0 0 0 0 0 2 3 1 0 0 0 0 0 0 0 0 0 0 0 0 0 0 0 0 0 0 0 0 0 0 0 0 0 0 0 0 0 0 0 0 0 0 0 0 0 0 0 0 0 0 0 0 0 0 0 0 0 0 0 0 0 0 0 0 0

"101079_fr6" 1 1 0 1 0 2 0 0 0 0 0 0 0 0 0 0 2 0 0 1 1 2 4 0 0 0 1 0 0 0 1 0 2 0 0 0 0 0 0 0 0 0 0 0 0 0 0 0 0 0 0 0 0 0 0 0 0 0 0 0 0 0 0 0 0 0 0 0 0 0 0 0 0 0 0 0 0 0 0 0 0 0 0 0 0

"10109_fr4" 0 1 1 0 0 0 1 1 0 0 0 0 4 0 1 0 0 2 0 0 1 0 0 0 2 0 0 0 0 0 0 0 0 0 0 0 0 0 0 0 0 0 0 0 0 0 0 0 0 0 0 0 0 0 0 0 0 0 0 0 0 0 0 0 0 0 0 0 0 0 0 0 0 0 0 0 0 0 0 0 0 0 0 0 0

"10149_fr3" 0 2 1 0 0 0 2 1 0 6 0 0 4 0 0 0 0 4 0 0 0 0 0 0 4 0 0 0 0 0 0 0 0 0 0 0 0 0 0 0 0 0 0 0 0 0 0 0 0 0 0 0 0 0 0 0 0 0 0 0 0 0 0 0 0 1 0 0 0 0 0 2 0 3 4 0 1 4 3 4 1 1 4 0 1

"10171_fr5" 0 0 19 25 15 30 0 29 7 0 18 7 1 15 0 1 14 2 1 14 15 1 17 13 1 13 27 14 26 14 1 22 0 0 5 0 0 0 1 1 2 0 0 0 0 4 0 2 1 3 6 0 2 2 0 1 0 12 0 3 2 4 0 0 1 0 0 3 1 0 0 0 0 1 0 1 1 2 5 7 0 0 0 0 0

"10189_fr3" 0 0 1 2 0 0 2 0 0 0 0 0 0 0 3 0 1 0 0 0 0 0 0 0 3 0 0 0 0 0 2 0 0 0 3 0 0 0 0 0 0 0 0 0 0 0 0 0 0 0 0 0 0 0 0 0 0 0 0 0 0 0 0 0 0 0 0 0 0 0 0 0 0 0 0 0 0 0 0 0 0 0 0 0 0

"1019_fr4" 0 0 0 0 0 0 0 0 0 0 0 0 0 0 0 0 0 0 0 0 0 0 0 0 0 0 0 0 3 0 0 0 0 0 0 0 0 0 0 0 0 0 0 0 0 0 0 0 0 0 0 0 0 0 0 0 0 0 0 0 0 0 0 0 0 0 0 0 0 0 0 0 0 0 0 0 0 0 0 0 0 0 0 0 0

"101957_fr1" 0 0 0 0 0 0 0 0 0 0 0 0 0 0 0 0 0 0 0 0 0 0 0 0 0 0 0 0 0 0 0 0 0 0 0 0 0 0 0 0 0 0 0 0 0 0 0 0 0 0 0 0 0 0 0 0 0 0 0 0 0 0 0 0 0 2 0 1 0 0 0 0 0 0 0 0 0 0 0 0 0 0 3 0 1

"101957_fr2" 0 1 0 0 0 0 1 2 0 5 0 2 5 0 0 0 0 2 0 0 4 0 0 0 4 0 0 0 2 0 0 0 0 0 0 0 0 0 0 0 0 0 0 0 0 0 0 0 0 0 0 0 0 0 0 0 0 0 0 0 0 0 0 0 0 0 0 0 0 0 0 0 0 0 0 0 0 0 0 0 0 0 0 0 0

"102_fr5" 0 0 0 0 0 0 0 0 0 0 0 0 0 0 0 0 0 0 0 0 0 0 0 0 0 0 0 0 0 0 0 0 0 0 0 0 0 2 0 0 0 0 0 3 2 0 2 0 1 1 0 0 0 0 1 0 1 0 1 0 0 0 0 0 0 0 0 0 0 0 0 0 0 0 0 0 0 0 0 0 0 0 0 0 0

"102024_fr2" 0 0 0 0 0 0 0 0 0 0 0 0 0 0 0 0 0 0 0 0 0 0 4 0 0 2 0 0 0 0 0 0 3 0 0 0 0 0 0 0 0 0 0 0 0 0 0 0 0 0 0 0 0 0 0 0 0 0 0 0 0 0 0 0 0 0 0 0 0 0 0 0 0 0 0 0 0 0 0 0 0 0 0 0 0

"1024_fr3" 0 0 0 0 0 0 0 0 0 0 0 0 0 2 0 0 0 0 0 0 0 0 0 0 0 0 0 0 0 1 0 0 0 0 0 0 0 0 0 0 0 0 0 0 0 0 0 0 0 0 0 0 0 0 0 0 0 0 0 0 0 0 0 0 0 0 0 0 0 0 0 0 0 0 0 0 0 0 0 0 0 0 0 0 0

"10262_fr3" 2 1 0 0 2 2 0 0 0 0 2 0 0 2 0 0 1 0 2 2 1 2 2 0 0 2 2 1 0 2 0 0 2 2 0 0 0 0 0 0 0 0 0 0 0 0 0 0 0 0 0 0 0 0 0 0 0 0 0 0 0 0 0 0 0 0 0 0 0 0 0 0 0 0 0 0 0 0 0 0 0 0 0 0 0

"103291_fr4" 0 0 0 0 0 0 0 0 0 0 0 0 0 5 0 0 0 0 0 0 0 0 0 0 0 1 0 0 0 0 0 0 0 0 0 0 0 0 0 0 0 0 0 0 0 0 0 0 0 0 0 0 0 0 0 0 0 0 0 0 0 0 0 0 0 0 0 0 0 0 0 0 0 0 0 0 0 0 0 0 0 0 0 0 0

"103394_fr3" 1 0 2 2 2 0 0 0 1 1 0 0 1 0 0 2 0 0 0 0 0 0 0 0 0 0 0 0 1 0 0 0 0 0 0 0 0 0 0 0 0 0 0 0 0 0 0 0 0 0 0 0 0 0 0 0 0 0 0 0 0 0 0 0 0 0 0 0 0 0 0 0 0 0 0 0 0 0 0 0 0 0 0 0 0

"10389_fr3" 0 0 1 0 0 0 0 0 0 0 0 0 0 0 0 0 0 0 2 0 0 0 3 0 0 0 0 0 0 0 0 0 0 0 0 0 0 0 0 0 0 0 0 0 0 0 0 0 0 0 0 0 0 0 0 0 0 0 0 0 0 0 0 0 0 2 2 0 0 0 2 2 2 3 0 2 3 2 0 4 0 0 0 0 1

"10397_fr1" 6 2 0 0 4 0 0 2 1 0 1 3 0 4 0 0 2 0 2 4 2 3 2 2 0 4 2 4 1 4 2 2 2 2 0 0 1 0 0 4 0 0 0 0 0 0 0 0 0 0 0 0 0 0 0 0 0 0 0 0 0 0 0 0 0 0 0 0 0 0 0 0 0 0 1 0 0 1 0 0 3 0 0 0 0

"105002_fr6" 0 2 0 1 0 2 3 1 0 0 0 0 0 0 0 0 0 0 0 0 0 0 1 0 0 1 1 2 1 1 1 1 0 2 2 0 0 0 0 0 0 0 0 0 0 0 0 0 0 0 0 0 0 0 0 0 0 0 0 0 0 0 0 0 0 0 0 0 0 0 0 0 0 0 0 0 0 0 0 0 0 0 0 0 0

"105020_fr5" 2 1 4 8 1 2 4 4 7 11 1 2 4 0 4 4 4 11 0 4 12 6 2 6 9 2 0 2 6 6 9 4 4 11 10 1 0 5 0 7 0 0 0 0 0 0 0 0 0 0 0 0 0 0 0 0 0 0 0 0 0 0 0 0 0 0 0 0 0 0 0 0 2 0 0 0 0 1 0 0 2 2 1 0 2

"105024_fr6" 0 0 0 0 0 0 0 0 0 0 0 0 0 0 0 0 0 0 0 0 0 0 0 0 0 0 0 0 0 0 0 0 0 0 0 0 0 0 0 0 0 0 0 0 0 0 0 0 0 0 0 0 0 0 0 0 0 0 0 0 0 1 0 0 0 0 0 0 0 2 0 0 0 0 3 0 0 0 0 0 0 0 0 0 0

"105032_fr3" 0 0 0 0 0 0 0 2 0 0 0 0 0 0 0 0 0 0 0 0 0 0 3 0 0 0 0 0 0 0 0 0 1 0 0 0 0 0 0 0 0 0 0 0 0 0 0 0 0 0 0 0 0 0 0 0 0 0 0 0 0 0 0 0 0 0 0 0 0 0 0 0 0 0 0 0 0 0 0 0 0 0 0 0 0

"105044_fr3" 0 0 0 0 2 0 0 0 0 0 0 1 0 3 0 0 0 0 0 3 0 1 0 2 0 0 0 0 0 0 0 0 0 0 0 0 0 0 0 0 0 0 0 0 0 0 0 0 0 0 0 0 0 0 0 0 0 0 0 0 0 0 0 0 0 0 0 0 0 0 2 0 0 0 0 0 0 0 0 1 0 0 0 2 0

"105132_fr2" 0 0 0 0 0 0 0 0 0 0 0 0 0 0 0 2 0 4 0 0 0 3 0 0 0 0 0 0 0 0 0 0 0 0 0 0 0 0 0 0 0 0 0 0 0 0 0 0 0 0 0 0 0 0 0 0 0 0 0 0 0 0 1 0 0 0 0 0 0 0 0 0 3 1 1 0 0 0 0 0 0 0 0 0 0

"105143_fr3" 0 0 0 0 0 0 0 0 0 0 0 0 0 0 0 0 0 0 0 0 0 0 0 0 0 0 3 0 0 2 0 2 0 0 0 0 0 0 0 0 0 0 0 0 0 0 0 0 0 0 0 0 0 0 0 0 0 0 0 0 0 0 0 0 0 0 0 1 0 0 0 0 0 0 0 0 0 0 0 1 0 0 0 0 0

"105148_fr3" 0 0 0 0 0 0 0 0 0 0 0 0 0 0 0 0 0 0 0 0 0 0 0 0 0 0 0 0 0 0 0 0 0 0 0 0 0 0 0 0 0 0 0 0 0 0 0 0 0 0 0 0 0 0 0 0 0 0 0 0 0 0 0 0 1 3 0 0 3 1 0 0 0 0 0 0 0 0 0 0 0 0 0 0 0

"10515_fr1" 0 1 0 0 0 2 0 0 1 0 1 2 2 2 0 0 4 0 2 2 1 4 0 0 0 1 0 2 0 4 0 0 0 2 0 0 0 0 0 0 0 0 0 0 0 0 0 0 0 0 0 0 0 0 0 0 0 0 0 0 0 0 0 0 0 0 0 0 0 0 0 0 0 0 0 0 0 0 0 0 0 0 0 0 0

"105194_fr6" 3 5 0 1 0 0 5 5 0 0 0 5 4 0 4 4 6 0 0 0 0 0 0 0 0 0 0 0 0 0 0 0 0 0 4 1 0 2 1 2 3 0 1 2 2 2 2 2 2 2 2 2 1 2 2 1 2 2 2 2 0 1 0 0 1 2 1 2 1 1 1 2 2 1 2 2 0 2 2 0 2 2 2 1 2

"105224_fr3" 0 0 0 0 0 0 0 0 0 0 0 0 0 0 0 0 0 0 0 0 0 0 0 0 1 0 0 0 0 0 0 0 0 0 0 0 0 0 0 0 0 0 0 0 0 0 0 0 0 0 0 0 0 0 0 0 0 0 0 0 0 0 0 0 0 0 0 0 0 0 0 0 0 0 0 0 0 0 0 0 0 0 0 0 0

"105272_fr4" 0 0 2 2 2 2 2 0 2 0 2 2 2 2 2 0 2 0 2 2 0 0 0 2 0 0 0 2 0 0 0 0 2 0 2 0 0 0 0 0 0 0 0 0 0 0 0 0 0 0 0 0 0 0 0 0 0 0 0 0 0 0 0 0 0 0 0 0 0 0 0 0 0 0 0 0 0 0 0 0 0 0 0 0 0

"105311_fr3" 0 0 0 0 0 0 0 0 0 0 0 0 0 0 0 0 0 0 0 0 0 0 0 1 0 0 0 0 0 0 0 0 0 0 0 0 0 0 0 0 0 0 0 0 0 0 0 0 0 0 0 0 0 0 0 0 0 0 0 0 0 0 0 0 0 0 0 0 0 0 0 0 0 0 0 0 0 0 0 0 0 0 0 0 0

"105371_fr5" 4 0 0 0 0 0 0 0 0 0 0 0 1 0 0 0 3 0 2 0 2 0 2 0 0 1 0 0 0 2 0 1 1 1 0 0 0 0 0 0 0 0 0 0 0 0 0 0 0 0 0 0 0 0 0 0 0 0 0 0 0 0 0 0 0 0 0 0 0 0 0 0 0 0 0 0 0 0 0 0 0 0 0 0 0

"105389_fr4" 0 0 0 0 4 0 0 0 0 0 0 0 0 5 0 0 1 0 3 5 2 1 4 2 0 2 1 0 1 4 0 3 4 3 2 0 0 0 0 0 0 0 0 0 0 0 0 0 0 0 0 0 0 0 0 0 0 0 0 0 0 0 0 0 0 0 0 0 0 0 0 0 0 0 0 0 0 0 0 0 0 0 0 0 0

"105399_fr4" 0 0 0 0 0 0 0 0 0 0 0 0 0 0 0 0 0 0 0 0 0 0 0 0 0 0 0 0 0 0 0 0 0 0 0 1 0 0 0 0 0 0 0 2 0 0 0 0 2 0 0 0 0 0 0 0 0 0 0 0 0 0 0 0 0 0 0 0 0 0 0 0 0 0 0 0 0 0 0 0 0 0 0 0 0

"105406_fr4" 0 0 0 0 0 0 0 0 0 0 0 0 0 0 0 0 0 0 0 0 0 0 0 0 0 0 0 0 0 0 0 0 0 0 0 0 0 0 0 0 0 0 0 0 0 0 0 0 0 0 0 0 0 0 0 0 0 0 0 0 0 0 0 0 0 0 0 0 0 0 0 1 0 0 0 0 0 1 1 2 0 0 0 0 0

"105465_fr4" 0 0 0 0 0 0 0 0 0 0 0 0 0 0 0 0 0 0 0 0 0 0 2 0 0 0 0 0 0 0 0 0 0 0 0 0 0 0 0 0 0 0 0 0 0 0 0 0 0 0 0 0 0 0 0 0 0 0 0 0 0 0 0 0 0 0 0 0 0 0 0 0 0 0 0 0 0 0 0 0 0 0 0 0 0

"105472_fr5" 2 6 6 1 5 3 5 4 1 4 6 2 1 0 1 5 4 5 6 5 7 2 5 4 4 3 5 1 0 3 1 5 1 2 0 0 0 0 0 0 0 0 0 0 0 0 0 0 0 0 0 0 0 0 0 0 0 0 0 0 0 0 0 0 0 0 0 0 0 0 0 0 0 0 0 0 0 0 0 0 0 0 0 0 0

"105505_fr5" 0 0 0 0 0 0 0 1 0 0 0 0 0 0 0 0 0 0 0 0 0 0 0 0 0 0 0 0 0 0 0 0 0 0 0 0 0 0 0 0 0 0 0 0 0 0 0 0 0 0 0 0 0 0 0 0 0 0 0 0 0 0 0 0 0 0 0 0 0 0 0 0 0 0 0 0 0 0 0 0 0 0 0 0 0

"105625_fr2" 0 0 0 0 0 0 0 0 0 0 0 0 0 0 0 0 0 0 0 0 0 0 0 0 0 0 0 0 0 0 0 0 0 0 0 0 0 0 0 0 0 0 0 0 0 0 0 0 0 0 0 0 0 0 0 0 0 0 0 0 0 0 0 0 0 0 0 0 0 0 0 2 0 0 1 0 4 2 2 3 3 2 0 1 0

"105628_fr5" 0 0 0 0 0 0 0 2 0 0 0 0 0 0 0 3 0 0 0 0 0 0 0 0 1 0 0 0 0 0 0 0 0 0 1 0 0 0 0 0 0 0 0 0 0 0 0 0 0 0 0 0 0 0 0 0 0 0 0 0 0 0 0 0 0 0 0 0 0 0 0 0 0 0 0 0 0 0 0 0 0 0 0 0 0

"105692_fr1" 0 0 0 2 0 0 0 0 0 0 0 0 0 0 0 0 0 0 0 0 0 0 0 0 0 0 0 0 0 0 0 0 0 0 0 0 0 0 0 0 0 0 0 0 0 0 0 0 0 0 0 0 0 0 0 0 0 0 0 0 0 0 0 0 0 0 0 0 0 0 0 0 0 0 0 0 0 0 0 0 0 0 0 0 0

"105712_fr5" 2 0 4 0 1 0 0 1 0 0 0 0 0 1 0 1 0 0 0 0 2 0 2 0 0 2 1 0 4 0 1 2 0 1 0 0 0 0 0 0 0 0 0 0 0 0 0 0 0 0 0 0 0 0 0 0 0 0 0 0 0 0 0 0 0 0 0 0 0 0 0 0 1 2 1 0 2 2 1 0 0 0 2 2 2

"105765_fr4" 0 2 0 0 0 2 0 0 0 0 2 0 0 1 0 0 0 0 0 0 2 0 0 0 0 0 2 0 0 1 0 2 0 2 0 0 0 0 0 0 0 0 0 0 0 0 0 0 0 0 0 0 0 0 0 0 0 0 0 0 0 0 0 0 0 0 0 0 0 0 0 0 0 0 0 0 0 0 0 0 0 0 0 0 0

"10580_fr5" 0 0 0 0 0 0 0 0 0 0 0 0 0 1 0 0 0 0 0 0 0 0 0 0 1 4 0 0 1 0 0 0 0 0 0 0 0 0 0 0 0 0 0 0 0 0 0 0 0 0 0 0 0 0 0 0 0 0 4 0 0 0 0 0 0 0 0 0 0 0 0 0 0 0 0 0 0 0 0 0 0 2 0 0 0

"10587_fr4" 0 0 0 0 0 0 0 0 0 0 0 0 0 0 0 0 0 0 1 0 0 0 0 0 0 1 0 0 0 0 0 0 0 0 0 0 0 0 0 0 0 0 0 0 0 0 0 0 0 0 0 0 0 0 0 0 0 0 0 0 0 0 0 0 0 0 0 0 0 0 0 0 0 0 0 0 0 0 0 0 0 0 0 0 0

"10596_fr1" 0 0 0 0 0 0 0 0 0 0 0 0 0 0 0 0 0 0 0 0 0 0 0 1 0 0 0 0 0 0 0 0 0 0 0 0 0 0 0 0 0 0 0 0 0 0 0 0 0 0 0 0 0 0 0 0 0 0 0 0 0 0 0 0 0 0 0 0 0 0 0 0 0 0 0 0 0 0 0 0 0 0 0 0 0

"106001_fr4" 0 0 0 0 0 0 2 0 0 2 0 0 0 1 0 1 1 0 0 1 0 0 2 0 0 0 2 0 0 0 3 0 1 0 0 0 0 0 0 0 0 0 0 0 0 0 0 0 0 0 0 0 0 0 0 0 0 0 0 0 0 0 0 0 0 0 0 0 0 0 0 0 0 0 0 0 0 0 0 0 0 0 0 0 0

"10601_fr5" 0 0 0 0 0 0 0 0 0 0 0 0 0 0 0 0 0 0 0 0 0 0 0 0 0 0 0 0 0 0 0 0 0 0 0 0 0 0 0 0 0 0 0 0 0 0 0 0 0 0 0 0 0 0 0 0 0 0 0 0 0 0 0 0 0 3 1 0 1 0 2 2 1 2 2 1 1 1 0 1 0 0 0 0 0

"106155_fr4" 0 0 0 0 0 0 0 0 0 4 0 0 0 0 0 0 0 0 0 0 0 0 0 0 2 2 0 0 2 0 2 0 0 0 0 0 0 0 0 0 0 0 0 0 0 0 0 0 0 0 0 0 0 0 0 0 0 0 0 0 0 0 0 0 0 0 0 0 0 0 0 0 0 0 0 0 0 0 0 0 0 0 0 0 0

"106201_fr2" 0 0 0 0 0 0 0 0 0 0 0 0 0 0 0 0 0 0 1 0 0 0 0 1 0 0 0 0 0 2 0 0 0 0 0 0 0 0 0 0 0 0 0 0 0 0 0 0 0 0 0 0 0 0 0 0 0 0 0 0 0 0 0 0 0 0 0 0 0 0 0 0 0 0 0 0 0 0 0 0 0 0 0 0 0

"10630_fr3" 0 0 0 0 0 0 0 0 0 0 0 0 0 3 0 0 0 0 0 0 0 0 0 0 0 0 0 0 0 0 0 0 0 0 0 0 0 0 0 0 0 0 0 0 0 0 0 0 0 0 0 0 0 0 0 0 0 0 0 0 0 0 0 0 0 0 0 0 1 0 0 0 0 2 0 4 5 5 4 4 6 6 5 3 4

"10635_fr3" 0 0 2 3 0 4 2 3 1 0 2 2 0 0 0 0 2 0 1 1 2 1 0 1 0 0 4 1 4 3 1 1 1 1 2 0 0 0 0 0 0 0 0 0 0 0 0 0 0 0 0 0 0 0 0 0 0 0 0 0 0 0 0 0 0 0 0 0 0 0 1 0 2 2 0 3 2 2 2 2 1 1 1 2 2

"106378_fr4" 0 2 0 0 0 0 0 0 0 0 0 0 0 0 0 0 0 0 1 0 2 0 0 0 0 0 0 0 0 0 2 3 0 1 0 0 0 0 0 0 0 0 0 0 0 0 0 0 0 0 0 0 0 0 0 0 0 0 0 0 0 0 0 0 0 1 0 1 0 0 0 2 2 2 3 3 4 2 4 2 1 0 3 0 0

"1065_fr4" 6 3 4 4 4 5 5 4 4 10 4 5 4 4 3 4 4 8 3 3 3 4 5 4 3 4 4 4 4 4 4 4 5 3 4 4 6 8 2 8 0 0 4 4 0 3 0 1 0 3 0 0 0 4 0 1 2 2 0 0 2 0 0 2 0 0 0 2 2 0 2 4 5 2 1 2 6 8 4 8 5 6 9 8 6

"106531_fr4" 0 0 1 2 0 0 0 1 0 0 0 0 0 0 0 0 0 0 0 0 0 0 0 0 0 0 0 0 0 0 0 0 0 0 0 0 0 0 0 0 0 0 0 0 0 0 0 0 0 0 0 0 0 0 0 0 0 0 0 0 0 0 0 0 0 0 0 0 0 0 0 0 0 0 0 0 0 0 0 0 0 0 0 0 0

"106629_fr1" 0 1 0 0 1 0 0 0 0 0 0 0 0 2 0 2 1 0 2 2 2 0 1 1 0 1 2 0 0 0 0 1 0 0 0 0 0 0 0 0 0 0 0 0 0 0 0 0 0 0 0 0 0 0 0 0 0 0 0 0 0 0 0 0 0 0 0 0 0 0 0 0 0 0 0 0 0 0 0 0 0 0 0 0 0

"106723_fr6" 1 1 0 0 2 0 1 0 0 0 0 0 0 0 0 3 2 0 0 0 0 0 1 0 1 0 0 0 0 0 0 0 0 1 1 0 0 0 0 0 0 0 0 0 0 0 0 0 0 0 0 0 0 0 0 0 0 0 0 0 0 0 0 0 0 0 0 0 0 0 0 0 0 0 0 0 0 0 0 0 0 0 0 0 0

"106795_fr3" 0 0 0 0 0 0 0 0 0 0 0 0 0 0 0 0 0 0 0 0 0 0 0 0 0 0 0 0 0 0 2 3 0 0 0 0 0 0 0 0 0 0 0 0 0 0 0 0 0 0 0 0 0 0 0 0 0 0 0 0 0 0 0 0 0 0 0 0 0 0 0 0 0 0 0 0 0 0 0 0 0 0 0 0 0

"107301_fr2" 0 0 1 0 0 1 0 0 0 0 0 0 0 0 0 0 0 0 0 0 0 0 0 0 0 0 0 0 0 0 0 0 0 0 0 0 0 0 0 0 0 0 0 0 0 0 0 0 0 0 0 0 0 0 0 0 0 0 0 0 0 0 0 0 0 0 0 0 0 0 0 0 0 0 0 0 0 0 0 0 0 0 0 0 0

"107314_fr5" 0 0 1 0 0 0 0 0 0 0 1 0 0 1 0 0 0 0 0 0 0 0 2 0 0 2 1 0 2 0 3 0 1 0 0 0 0 0 0 0 0 0 0 0 0 0 0 0 0 0 0 0 0 0 0 0 0 0 0 0 0 0 0 0 0 0 0 0 0 0 0 0 0 0 0 0 0 0 0 0 0 0 0 0 0

"10740_fr1" 0 2 2 0 4 3 0 4 0 1 0 1 0 2 0 0 0 0 2 3 4 1 4 4 2 2 3 0 3 4 0 3 1 4 1 0 0 0 0 0 0 0 0 0 0 0 0 0 0 0 0 0 0 0 0 0 0 0 0 0 0 0 0 0 0 0 0 0 0 0 0 0 0 1 0 0 3 3 3 0 0 0 3 0 1

"10761_fr5" 0 0 0 0 0 0 0 0 0 0 0 0 0 0 0 0 0 0 0 0 0 0 0 0 0 0 0 0 0 0 0 0 0 0 0 0 1 1 0 3 1 0 0 2 0 0 1 0 0 0 0 0 1 0 0 0 0 0 0 1 0 0 0 0 0 0 0 0 0 0 0 0 0 0 0 0 0 0 0 0 0 0 0 0 0

"10775_fr2" 0 0 0 0 0 0 0 0 0 3 0 0 2 0 0 0 0 1 0 0 0 0 0 0 2 0 0 0 0 0 0 0 0 0 0 0 0 0 0 0 0 0 0 0 0 0 0 0 0 0 0 0 0 0 0 0 0 0 0 0 0 0 0 0 0 0 0 0 0 0 0 0 0 0 0 0 0 0 0 0 0 0 0 0 0

"108_fr5" 0 0 0 0 0 0 0 0 0 0 0 0 0 2 7 0 0 0 10 0 0 0 2 12 0 2 0 0 0 0 0 0 0 2 0 0 0 0 0 0 0 0 0 0 0 0 0 0 0 0 0 0 0 0 0 0 0 0 0 0 0 0 0 0 0 0 0 0 0 0 0 0 0 0 0 0 0 0 0 0 0 0 0 0 0

"108041_fr1" 0 0 2 0 0 0 0 0 0 0 0 0 0 0 0 0 0 0 0 2 0 0 0 0 0 0 0 0 0 1 0 0 0 0 0 0 0 0 0 0 0 0 0 0 0 0 0 0 0 0 0 0 0 0 0 0 0 0 0 0 0 0 0 0 0 0 0 0 0 0 0 0 0 0 0 0 0 0 0 0 0 0 0 0 0

"108701_fr3" 0 0 0 0 2 1 0 0 0 0 0 0 0 0 3 0 3 0 2 0 3 1 4 2 0 4 0 0 0 0 0 4 3 2 3 0 0 0 0 0 0 0 0 0 0 0 0 0 0 0 0 0 0 0 0 0 0 0 0 0 0 0 0 0 0 0 0 0 0 0 0 0 0 0 0 0 0 0 0 0 0 0 0 0 0

"109189_fr5" 0 3 0 0 0 0 0 3 0 0 0 0 0 3 0 0 3 0 0 0 0 0 0 0 0 5 0 0 0 0 0 2 0 0 0 0 0 0 0 0 0 0 0 0 0 0 0 0 0 0 0 0 0 0 0 0 0 0 0 0 0 0 0 0 0 0 0 0 0 0 0 0 0 0 0 0 0 0 0 0 0 0 0 0 0

"1094_fr3" 0 1 1 0 2 0 0 0 0 0 0 0 0 0 0 0 0 0 0 0 0 0 0 4 0 0 0 0 0 0 0 0 0 0 0 0 0 0 0 0 0 0 0 0 0 0 0 0 0 0 0 0 0 0 0 0 0 0 0 0 0 0 0 0 0 0 0 0 0 0 0 0 0 0 0 0 0 0 0 0 0 0 0 0 0

"109413_fr2" 0 0 0 0 1 1 0 0 0 0 0 0 0 4 0 1 3 0 1 3 2 2 3 2 0 2 2 0 3 2 2 2 2 2 2 0 0 0 0 0 0 0 0 0 0 0 0 0 0 0 0 0 0 0 0 0 0 0 0 0 0 0 0 0 0 0 0 0 0 0 0 0 0 0 0 0 0 0 0 0 0 0 0 0 0

"10958_fr4" 0 0 0 0 0 0 0 0 0 0 0 0 0 0 0 0 0 0 0 0 0 0 0 0 0 0 0 0 0 0 0 2 0 0 0 0 0 0 0 0 0 0 0 0 0 0 0 0 0 0 0 0 0 0 0 0 0 0 0 0 0 0 0 0 0 0 0 0 0 0 0 0 0 0 0 0 0 0 0 0 0 0 0 0 0

"110907_fr2" 1 2 15 16 0 3 3 15 2 6 0 3 0 2 3 4 2 3 2 1 2 0 2 1 6 17 14 1 12 0 2 6 1 0 3 2 1 2 1 2 0 0 2 0 0 0 0 0 0 0 0 0 0 0 1 1 0 6 1 0 0 0 0 0 0 0 0 0 0 0 0 2 2 0 0 0 0 2 3 2 2 4 5 3 6

"110912_fr3" 0 0 0 0 0 0 0 0 0 0 0 0 0 0 0 0 0 0 0 0 0 0 0 0 0 2 0 0 0 0 7 2 0 0 0 0 0 0 0 0 0 0 0 0 0 0 0 0 0 0 0 0 0 0 0 0 0 0 0 0 0 0 0 0 0 0 0 0 0 0 0 0 0 0 0 0 0 0 0 0 0 0 0 0 0

"110918_fr2" 3 4 3 3 3 2 5 4 3 3 2 2 5 2 4 3 2 2 2 2 3 3 5 3 2 2 2 1 2 4 2 2 2 3 5 0 0 0 0 0 0 0 0 0 0 0 0 0 0 0 0 0 0 0 0 0 0 0 0 0 0 1 0 2 0 2 2 2 1 0 2 2 1 2 2 2 1 2 2 2 2 3 2 2 2

"110946_fr1" 0 0 0 0 0 1 0 0 0 2 0 0 0 0 0 0 0 0 0 0 0 0 0 0 0 0 0 0 0 0 0 0 0 0 0 0 0 0 0 0 0 0 0 0 0 0 0 0 0 0 0 0 0 0 0 0 0 0 0 0 0 0 0 0 0 0 0 0 0 0 0 0 0 0 0 0 0 0 0 0 0 0 0 0 0

"110964_fr1" 0 1 1 0 0 0 0 0 0 0 0 1 0 0 0 0 0 1 0 0 0 0 0 0 0 0 0 0 0 0 0 0 0 0 0 0 0 0 0 0 0 0 0 0 0 0 0 0 0 0 0 0 0 0 0 0 0 0 0 0 0 0 0 0 0 0 0 0 0 0 0 0 0 0 0 0 0 0 0 0 0 0 0 0 0

"110976_fr3" 0 0 0 1 0 0 0 0 0 0 0 0 0 0 0 0 0 0 0 0 0 0 0 0 0 0 0 0 0 0 0 0 0 0 0 0 0 0 0 0 0 0 0 0 0 0 0 0 0 0 0 0 0 0 0 0 0 0 0 0 0 0 0 0 0 0 0 0 0 0 0 0 0 0 0 0 0 0 0 0 0 0 0 0 0

"110978_fr6" 0 0 0 0 0 0 0 0 0 0 0 0 0 0 2 2 0 0 0 0 0 0 0 0 0 0 0 4 0 0 0 0 0 0 5 0 0 0 0 0 0 0 0 4 0 4 4 4 4 0 3 0 3 4 0 0 4 3 1 0 0 0 0 0 3 0 0 0 0 0 0 0 0 0 0 0 0 0 0 0 0 0 0 0 0

"111094_fr4" 0 0 0 0 0 0 0 0 0 0 0 0 0 0 0 0 0 0 0 0 0 0 0 0 0 0 0 0 0 0 0 0 0 0 0 0 0 0 1 0 0 0 0 0 0 3 0 2 3 3 0 2 0 1 6 2 2 0 0 3 0 0 0 0 0 0 0 0 0 0 0 0 0 0 0 0 0 0 0 0 0 0 0 0 0

"111201_fr2" 0 0 0 0 0 0 0 0 0 0 0 0 0 0 0 0 0 0 0 0 0 0 0 0 0 0 0 0 0 0 0 0 0 0 0 0 0 0 0 0 0 2 0 0 0 2 0 0 0 0 3 2 2 0 0 0 2 0 2 0 0 0 0 0 0 0 0 0 0 0 0 0 0 0 0 0 0 0 0 0 0 0 0 0 0

"111225_fr2" 1 0 0 0 6 0 0 0 0 0 0 0 0 7 0 0 0 0 6 0 4 0 0 0 0 0 0 0 0 5 1 8 0 2 0 0 0 0 0 0 0 0 0 0 0 0 0 0 0 0 0 0 0 0 0 0 0 0 0 0 0 0 0 0 0 0 0 0 0 0 0 0 0 0 0 0 0 0 0 0 0 0 0 0 0

"111453_fr5" 0 0 0 0 0 0 0 0 0 6 0 0 0 0 0 0 0 0 0 0 0 0 0 0 0 1 0 0 2 0 0 0 0 0 0 0 0 0 0 0 0 0 0 0 0 0 0 0 0 0 0 0 0 0 0 0 0 0 0 0 0 0 0 0 0 0 0 0 0 0 0 0 0 0 0 0 0 0 0 0 0 0 0 0 0

"111526_fr5" 1 7 0 0 2 0 0 0 0 0 0 3 0 0 0 0 0 0 0 0 0 0 0 0 0 0 0 0 1 0 0 0 0 0 0 0 0 0 0 0 0 0 0 0 0 0 0 0 0 0 0 0 0 0 0 0 0 0 0 0 0 0 0 0 0 0 0 0 0 0 0 0 0 0 0 0 0 0 0 0 0 0 0 0 0

"111529_fr5" 0 0 0 0 1 0 0 1 0 0 0 0 0 0 0 0 0 0 2 0 0 0 2 2 0 0 0 0 0 1 0 0 2 1 0 0 0 0 0 0 0 0 0 0 0 0 0 0 0 0 0 0 0 0 0 0 0 0 0 0 0 0 0 0 0 0 0 0 0 0 0 0 0 0 0 0 0 0 0 0 0 0 0 0 0

"111573_fr3" 0 0 0 0 0 0 0 0 0 0 0 0 0 0 0 0 0 0 0 0 0 0 0 0 0 0 0 0 0 1 0 0 0 0 0 0 0 0 0 0 0 0 0 0 0 0 0 0 0 0 0 0 0 0 0 0 0 0 0 0 0 0 0 0 0 0 0 0 0 0 0 0 0 0 0 0 0 0 0 0 0 0 0 0 0

"111697_fr2" 0 0 0 0 0 0 0 0 0 0 0 0 0 0 0 0 0 0 0 0 0 0 0 0 0 0 0 0 0 0 0 0 0 0 0 0 0 0 0 0 0 0 0 0 0 0 0 0 0 0 0 0 0 0 0 0 0 0 0 0 0 0 0 0 0 0 0 0 0 0 0 0 0 0 0 0 2 0 0 1 0 0 0 0 0

"111710_fr5" 0 0 0 0 0 0 0 0 0 0 0 0 0 0 0 0 0 0 0 2 0 0 0 0 0 0 0 3 0 0 0 0 0 0 0 0 0 0 0 0 0 0 0 0 0 0 0 0 0 0 0 0 0 0 0 0 0 0 0 0 0 0 0 0 0 0 0 0 0 0 0 0 0 0 0 0 0 0 0 0 0 0 0 0 0

"111711_fr6" 0 1 0 0 0 0 0 0 0 0 0 0 0 0 0 0 0 0 1 0 1 0 3 0 0 0 0 0 0 0 0 0 1 0 0 0 0 0 0 0 0 0 0 0 0 0 0 0 0 0 0 0 0 0 0 0 0 0 0 0 0 0 0 0 0 0 0 0 0 0 0 0 0 0 0 0 0 0 0 0 0 0 0 0 0

"111749_fr2" 0 0 0 4 4 0 0 0 0 0 0 0 0 4 0 0 0 0 0 0 0 0 4 2 0 0 3 0 2 8 0 4 0 0 0 0 0 0 0 0 0 0 0 0 0 0 0 0 0 0 0 0 0 0 0 0 0 0 0 0 0 0 0 0 0 0 0 0 0 0 0 0 0 0 0 0 0 5 5 0 0 0 4 0 5

"11178_fr5" 0 0 0 0 3 0 0 0 0 0 0 0 0 0 0 0 0 0 0 0 0 0 0 0 0 1 1 0 0 0 0 0 0 0 0 0 0 0 0 0 0 0 0 0 0 0 0 0 0 0 0 0 0 0 0 0 0 0 0 0 0 0 0 0 0 0 0 0 0 0 0 0 0 0 0 0 0 0 0 0 0 0 0 0 0

"111876_fr3" 5 0 0 0 3 0 0 0 0 0 0 0 0 0 0 0 0 0 0 0 0 0 0 2 0 2 0 0 0 0 0 2 0 2 0 0 0 0 0 0 0 0 0 0 0 0 0 0 0 0 0 0 0 0 0 0 0 0 0 0 0 0 0 0 0 0 0 0 0 0 0 0 0 0 0 0 0 0 0 0 0 0 0 0 0

"111922_fr5" 0 2 0 0 0 0 0 0 0 0 0 0 3 0 0 0 0 0 0 0 0 0 0 0 0 2 0 0 0 0 0 0 0 0 0 0 0 0 0 0 0 0 0 0 0 0 0 0 0 0 0 0 0 0 0 0 0 0 0 0 0 0 0 0 0 0 0 0 0 0 0 0 0 1 2 2 0 4 4 0 1 0 0 3 0

"11199_fr3" 0 0 0 0 0 0 0 0 0 0 0 0 0 0 0 0 0 0 0 4 0 0 0 0 0 0 0 0 0 0 0 0 0 0 0 0 0 0 0 0 0 0 0 0 0 0 0 0 0 0 0 0 0 0 0 0 0 0 0 0 0 0 0 0 0 0 0 0 0 0 0 0 0 0 0 0 0 0 0 0 0 0 0 0 0

"112216_fr4" 0 0 0 0 0 0 0 0 0 0 0 0 0 4 0 2 0 0 0 0 0 0 0 0 0 3 0 0 0 0 0 0 0 0 0 0 0 0 0 0 0 0 0 0 0 0 0 0 0 0 0 0 0 0 0 0 0 0 0 0 0 0 0 0 0 0 0 0 0 0 0 0 0 0 0 0 0 0 0 0 0 0 0 0 0

"112239_fr2" 0 0 0 0 0 0 0 0 0 0 0 0 0 0 0 0 0 0 0 0 0 0 0 0 0 0 0 0 0 0 0 0 0 0 0 0 0 0 0 0 0 0 0 0 0 0 0 0 0 0 0 0 0 0 0 0 0 0 0 0 0 0 0 0 0 0 0 3 0 0 0 0 0 0 0 0 0 0 0 0 0 0 0 0 0

"112257_fr4" 0 0 0 0 0 0 0 0 0 0 0 0 0 0 0 0 0 0 0 0 0 0 0 0 0 0 0 0 0 0 0 1 0 0 0 0 0 0 0 0 0 0 0 0 0 0 0 0 0 0 0 0 0 0 0 0 0 0 0 0 0 0 0 0 0 0 0 0 0 0 0 0 0 0 0 0 0 0 0 0 0 0 0 0 0

"112455_fr3" 0 0 1 0 0 0 0 0 0 0 0 0 0 0 0 0 0 0 0 0 0 0 0 0 0 0 0 0 0 0 0 0 0 0 0 0 0 0 0 0 0 0 0 0 0 0 0 0 0 0 0 0 0 0 0 0 0 0 0 0 0 0 0 0 0 0 0 0 0 0 0 0 0 0 0 0 0 0 0 0 0 0 0 0 0

"112559_fr6" 0 0 0 0 0 2 0 0 0 0 0 0 0 2 0 0 0 0 0 0 0 0 0 0 0 0 0 0 0 4 0 0 0 0 0 0 0 0 0 0 0 0 0 0 0 0 0 0 0 0 0 0 0 0 0 0 0 0 0 0 0 0 0 0 0 0 0 0 0 0 0 0 0 0 0 0 0 0 0 0 0 0 0 0 0

"11310_fr4" 1 0 0 0 0 0 0 0 0 0 0 0 0 0 0 0 0 0 0 0 0 0 2 2 0 0 0 0 4 0 0 0 0 0 0 0 0 1 0 1 0 0 0 0 0 0 0 0 0 0 0 0 0 0 0 0 0 0 0 0 0 0 0 0 0 4 3 2 3 2 3 3 0 1 2 2 0 1 0 2 0 0 0 0 1

"1134_fr1" 0 0 4 0 9 0 0 4 3 0 2 1 0 2 4 3 0 1 1 0 0 6 3 8 1 6 8 4 0 10 2 1 7 9 3 0 0 0 0 0 0 0 0 0 0 0 0 0 0 0 0 0 0 0 0 0 0 0 0 0 0 0 0 0 0 0 0 3 0 0 0 1 0 0 0 0 1 1 0 0 0 0 1 0 0

"114115_fr5" 0 0 0 0 4 0 0 0 0 0 0 0 0 0 0 0 0 0 0 0 0 0 0 0 0 0 0 0 0 0 0 0 0 0 0 0 0 0 0 0 0 0 0 0 0 0 0 0 0 0 0 0 0 0 0 0 0 0 0 0 0 0 0 0 0 0 0 0 0 0 0 0 0 0 0 0 0 0 0 0 0 0 0 0 0

"11425_fr6" 0 0 0 0 0 0 0 0 0 0 0 0 0 0 0 0 0 0 0 0 0 0 0 0 0 0 0 0 0 0 0 0 0 0 0 0 0 0 0 0 0 0 0 0 0 0 0 0 0 0 0 0 0 0 0 0 0 0 0 0 0 0 0 0 0 3 2 3 2 1 1 1 0 4 0 0 0 2 2 0 1 0 1 1 0

"11505_fr1" 0 0 0 0 0 0 0 0 0 0 0 0 0 0 0 0 0 0 0 0 0 0 0 0 0 0 0 0 1 0 0 0 0 0 0 0 0 0 0 0 0 0 0 0 0 0 0 0 0 0 0 0 0 0 0 0 0 0 0 0 0 0 0 0 0 0 0 0 0 0 0 0 0 0 0 0 0 0 0 0 0 0 0 0 0

"1157_fr3" 0 0 0 0 0 0 0 0 0 0 0 0 0 0 0 0 0 0 0 0 0 0 0 0 0 0 0 0 0 0 0 0 0 0 0 0 0 0 3 0 0 0 0 0 0 0 0 0 0 0 0 0 0 0 0 0 0 0 0 0 0 3 0 0 4 0 0 0 0 0 0 0 0 0 0 0 0 0 0 0 0 0 0 0 0

"1162_fr1" 0 0 0 0 0 0 1 1 0 1 0 0 0 0 0 0 0 0 0 0 0 0 0 0 0 0 0 0 0 0 0 0 0 0 0 0 0 0 0 0 0 0 0 0 0 0 0 0 0 0 0 0 0 0 0 0 0 0 0 0 0 0 0 0 0 0 0 0 0 0 0 0 0 0 0 0 0 0 0 0 0 0 0 0 0

"116225_fr4" 0 0 0 0 0 0 0 0 0 0 0 0 0 0 2 0 0 0 0 0 0 0 0 0 0 0 0 0 0 0 0 0 1 0 0 0 0 0 0 0 0 0 0 0 0 0 0 0 0 0 0 0 0 0 0 0 0 0 0 0 0 0 0 0 0 0 0 0 0 0 0 0 0 0 0 0 0 0 0 0 0 0 0 0 0

"116243_fr4" 0 0 0 0 0 0 0 0 0 0 0 0 1 0 0 0 0 0 4 0 0 0 1 0 0 1 0 0 0 0 2 0 0 0 0 0 0 0 0 0 0 0 0 0 0 0 0 0 0 0 0 0 0 0 0 0 0 0 0 0 0 0 0 0 0 0 0 0 0 0 0 0 0 0 0 0 0 0 0 0 0 0 0 0 0

"116254_fr2" 0 0 0 0 0 0 0 0 0 0 0 0 0 0 0 0 0 0 0 0 0 0 0 0 0 0 0 0 0 0 0 0 0 0 0 0 0 2 0 0 0 0 0 0 0 0 0 0 0 0 0 0 0 0 0 0 0 0 0 0 0 0 0 0 0 0 0 0 0 0 0 0 0 0 0 0 0 0 0 0 0 0 0 0 0

"116336_fr1" 2 0 0 0 1 1 0 0 0 0 0 0 0 0 0 0 0 0 0 1 1 0 0 2 0 1 0 0 0 0 1 2 0 0 0 0 0 0 0 0 0 0 0 0 0 0 0 0 0 0 0 0 0 0 0 0 0 0 0 0 0 0 0 0 0 0 0 0 0 0 0 0 0 0 0 0 0 0 0 0 0 0 0 0 0

"116357_fr4" 0 0 0 0 3 0 0 0 0 0 0 0 0 0 0 0 0 0 0 0 0 0 0 0 0 0 0 0 4 2 0 0 0 4 2 0 0 0 0 0 0 0 0 0 0 0 0 0 0 0 0 0 0 0 0 0 0 0 0 0 0 0 0 0 0 0 0 0 0 0 0 0 0 0 0 0 0 0 0 0 0 0 0 0 0

"116383_fr1" 0 0 0 0 0 0 0 0 0 0 0 0 0 0 0 0 0 1 0 0 0 0 0 4 1 0 0 0 0 0 3 0 0 0 2 0 0 0 2 0 1 0 2 2 0 2 0 0 0 0 0 3 2 0 0 2 4 0 3 2 0 0 0 0 0 0 0 0 0 0 0 0 0 0 0 0 0 0 0 0 0 0 0 0 0

"116386_fr6" 0 0 0 0 0 0 0 0 0 0 0 0 0 0 0 0 0 0 0 0 0 0 0 0 4 0 0 0 0 0 0 0 0 0 0 0 0 0 0 0 0 0 0 0 0 0 0 0 0 0 0 0 0 0 0 0 0 0 0 0 0 0 0 0 0 0 0 0 0 0 0 0 0 0 0 0 0 0 0 0 0 0 0 0 0

"116392_fr2" 0 1 2 0 1 1 1 1 0 0 0 1 0 2 0 0 2 1 1 0 2 1 2 2 0 2 2 0 2 0 0 0 1 0 0 0 0 0 0 0 0 0 0 0 0 0 0 0 0 0 0 0 0 0 0 0 0 0 0 0 0 0 0 0 0 0 0 0 0 0 0 0 0 0 0 0 0 0 0 0 0 0 0 0 0

"116403_fr1" 0 0 1 0 0 2 3 4 2 0 1 0 1 0 0 3 3 0 0 0 0 4 0 0 0 0 0 0 3 0 3 0 0 0 0 0 0 0 0 0 0 0 0 0 0 0 0 0 0 0 0 0 0 0 0 0 0 0 0 0 0 0 0 0 0 0 0 0 0 0 0 0 0 0 0 0 0 0 0 0 0 0 0 0 0

"116507_fr5" 0 0 0 0 0 0 0 0 0 0 0 0 0 0 0 6 0 0 0 0 0 0 0 0 0 0 0 0 0 0 0 0 0 0 0 0 0 0 0 0 0 0 0 0 0 0 0 0 0 0 0 0 0 0 0 0 0 0 0 0 0 0 0 0 1 0 1 2 2 0 0 0 0 0 0 0 0 0 0 0 0 0 0 0 0

"116538_fr6" 0 0 0 0 0 0 0 0 0 0 0 0 0 0 0 0 0 0 0 0 0 0 0 0 0 0 0 0 0 0 0 0 0 0 0 0 0 0 0 0 0 0 0 0 0 0 0 0 0 0 0 0 0 0 0 0 0 0 0 0 0 0 0 0 0 0 0 0 0 0 0 0 0 0 0 0 1 0 1 1 0 0 0 0 0

"116584_fr3" 0 1 0 0 2 1 0 0 1 0 0 0 0 0 0 0 0 0 0 0 0 0 0 1 0 0 0 0 0 1 0 0 0 1 0 0 0 0 0 0 0 0 0 0 0 0 0 0 0 0 0 0 0 0 0 0 0 0 0 0 0 0 0 0 0 0 0 0 0 0 0 0 0 0 0 0 0 0 0 0 0 0 0 0 0

"116790_fr1" 1 0 0 0 0 0 0 0 0 0 0 0 0 1 0 0 0 0 2 0 1 1 0 0 0 0 0 0 0 0 0 2 0 0 0 0 0 0 0 0 0 0 0 0 0 0 0 0 0 0 0 0 0 0 0 0 0 0 0 0 0 0 0 0 0 0 0 0 0 0 0 0 0 0 0 0 0 0 0 0 0 0 0 0 0

"116889_fr5" 2 3 2 2 4 2 2 3 2 2 2 2 2 2 1 2 2 2 3 2 2 2 3 4 2 2 2 2 2 2 0 3 0 0 1 0 0 0 0 0 0 0 0 0 0 0 0 0 0 0 0 0 0 0 0 0 0 0 0 0 0 0 0 0 0 0 0 0 0 0 0 0 0 0 0 0 0 0 0 0 0 0 0 0 0

"1169_fr5" 0 0 0 0 0 0 0 0 0 0 0 1 0 0 0 0 0 0 0 0 0 0 0 0 0 0 0 0 0 0 0 0 0 0 0 0 0 0 0 0 0 0 0 0 0 0 0 0 0 0 0 0 0 0 0 0 0 0 0 0 0 0 0 0 0 0 0 0 0 0 0 0 0 0 0 0 0 0 0 0 0 0 0 0 0

"11694_fr6" 1 2 0 2 0 0 0 0 0 0 0 0 0 2 1 0 0 0 4 4 1 0 4 0 0 2 0 2 0 3 0 0 4 3 2 0 0 0 0 0 0 0 0 0 0 0 0 0 0 0 0 0 0 0 0 0 0 0 0 0 0 0 0 0 0 0 0 0 0 0 0 0 0 0 0 0 0 0 0 0 0 0 0 0 0

"116996_fr2" 0 2 1 0 0 0 0 2 0 0 0 0 2 2 0 1 1 1 0 1 2 0 1 0 0 1 0 0 1 0 1 0 1 0 0 0 0 0 0 0 0 0 0 0 0 0 0 0 0 0 0 0 0 0 0 0 0 0 0 0 0 0 0 0 0 0 0 0 0 0 0 0 0 0 0 0 0 0 0 0 0 0 0 0 0

"11700_fr6" 0 0 0 0 0 0 0 0 0 0 0 0 0 0 0 0 0 0 0 0 0 0 3 0 0 4 1 0 1 0 0 0 0 0 0 0 0 0 0 0 0 0 0 0 0 0 0 0 0 0 0 0 0 0 0 0 0 0 0 0 0 0 0 0 0 0 0 0 0 0 0 0 0 0 0 0 0 0 0 0 0 0 0 0 0

"117014_fr2" 0 0 0 0 0 0 0 0 0 0 2 0 0 0 0 0 0 0 2 0 0 0 0 0 0 0 0 0 0 0 0 0 0 0 0 0 0 0 0 0 0 0 0 0 0 0 0 0 0 0 0 0 0 0 0 0 0 0 0 0 0 0 0 0 0 0 0 0 0 0 0 0 0 0 0 0 0 0 0 0 0 0 0 0 0

"117083_fr4" 0 0 0 0 0 0 0 0 0 0 0 0 0 0 0 4 1 0 0 0 3 0 0 0 0 0 2 0 0 0 0 2 0 0 0 0 0 0 0 0 0 0 0 0 0 0 0 0 0 0 0 0 0 0 0 0 0 0 0 0 0 0 0 0 0 0 0 0 0 0 0 0 0 0 0 0 0 0 0 0 0 0 0 0 0

"11710_fr5" 0 0 0 0 0 0 1 0 0 0 0 0 7 0 0 0 0 1 0 0 0 0 0 0 6 0 0 0 0 0 0 0 0 0 0 0 0 0 0 0 0 0 0 0 0 0 0 0 0 0 0 0 0 0 0 0 0 0 0 0 0 0 0 0 0 0 0 0 0 0 0 0 0 0 0 0 0 0 0 0 0 0 0 0 0

"117102_fr1" 1 5 8 0 7 0 2 0 0 0 0 0 2 4 5 7 5 7 5 6 0 0 2 5 3 7 6 1 8 0 2 7 6 5 8 0 0 0 0 0 0 0 0 0 0 0 0 0 0 0 0 0 0 0 0 0 0 3 0 0 0 0 0 0 0 0 0 0 0 0 0 0 0 0 0 0 4 1 4 0 2 2 1 0 1

"117114_fr5" 2 1 0 0 0 1 0 0 1 0 0 2 0 4 0 1 0 0 0 1 0 1 0 0 0 3 2 0 1 0 2 5 0 0 1 0 0 0 0 0 0 0 0 0 0 0 0 0 0 0 0 0 0 0 0 0 0 0 0 0 0 0 0 0 0 0 0 0 0 0 0 0 1 0 0 0 0 1 0 0 0 0 1 0 0

"11717_fr4" 9 4 4 5 2 5 2 4 2 0 0 0 2 2 0 0 0 1 1 2 1 14 0 2 5 1 4 0 3 0 0 5 2 1 0 0 0 4 0 0 0 0 2 0 0 0 4 0 5 0 0 0 0 0 0 0 2 2 0 0 0 0 0 0 0 0 0 0 0 2 0 0 0 1 1 0 2 1 2 1 0 0 0 0 0

"1172_fr4" 0 1 0 0 0 0 0 0 0 0 0 0 0 1 0 0 0 0 0 0 1 0 0 0 0 2 0 0 0 0 0 1 0 0 0 0 0 0 0 0 0 0 0 0 0 0 0 0 0 0 0 0 0 0 0 0 0 0 0 0 0 0 0 0 0 0 0 0 0 0 0 0 0 1 0 0 1 0 0 1 0 0 0 0 0

"11723_fr3" 0 0 0 0 0 0 0 0 0 0 0 0 0 0 0 0 0 0 2 0 0 0 0 0 0 0 0 0 0 4 0 0 0 0 0 0 0 0 0 0 0 0 0 0 0 0 0 0 0 0 0 0 0 0 0 0 0 0 0 0 0 0 0 0 0 0 0 1 0 0 3 4 4 7 7 15 2 2 2 1 0 0 0 0 0

"117630_fr3" 0 0 1 3 0 0 0 0 0 0 0 0 0 0 0 4 0 0 0 0 0 2 0 0 0 0 2 0 4 1 0 0 0 0 0 0 0 0 0 0 0 0 0 0 0 0 0 0 0 0 0 0 0 0 0 0 0 0 0 0 0 0 0 0 0 0 0 0 0 0 0 0 0 0 0 0 0 0 0 0 0 0 0 0 0

"11772_fr1" 0 0 0 0 0 0 0 0 0 0 0 0 0 0 0 0 0 0 0 0 0 0 2 0 0 0 0 0 0 0 0 0 3 0 0 0 0 0 0 0 0 0 0 0 0 0 0 0 0 0 0 0 0 0 0 0 0 0 0 0 0 0 0 0 0 0 0 0 0 0 0 0 0 0 0 0 0 0 0 0 0 0 0 0 0

"117813_fr6" 0 0 0 0 0 0 0 0 0 0 0 0 0 0 0 0 0 0 0 0 0 0 0 0 0 0 0 0 0 0 0 0 0 0 0 0 0 0 0 0 0 0 0 0 0 0 0 0 0 0 0 0 0 0 0 0 0 0 0 0 0 0 0 0 0 0 0 0 0 0 0 0 0 0 0 0 1 0 2 1 2 0 0 0 0

"117983_fr5" 0 0 0 0 0 0 0 0 1 0 0 0 0 0 0 0 0 0 0 0 0 0 0 0 0 0 0 0 0 2 0 0 0 0 0 0 0 0 0 0 0 0 0 0 0 0 0 0 0 0 0 0 0 0 0 0 0 0 0 0 0 0 0 0 0 0 0 0 0 0 0 0 0 0 0 0 0 0 0 0 0 0 0 0 0

"11839_fr4" 0 0 0 0 0 0 0 0 0 0 0 0 0 0 0 0 0 0 0 0 0 0 0 0 0 0 0 0 0 0 0 0 0 0 0 0 0 0 0 0 0 0 0 0 0 0 0 0 0 0 0 0 0 0 0 0 0 0 0 0 0 0 1 0 0 0 0 0 0 0 0 0 0 1 0 0 0 0 0 0 0 0 0 0 0

"11839_fr6" 0 0 0 0 0 0 0 0 0 0 0 0 0 0 0 0 0 0 0 0 0 0 0 0 0 0 0 0 0 0 0 2 0 0 0 0 0 0 0 0 0 0 0 0 0 0 0 0 0 0 0 3 0 0 0 0 0 0 0 0 0 0 0 0 0 0 0 0 0 0 0 0 0 0 0 0 0 0 0 0 0 0 0 0 0

"1186_fr5" 0 0 0 0 0 0 0 0 0 0 0 0 0 0 0 0 0 0 0 0 0 3 0 0 0 0 0 0 0 0 0 0 0 0 0 0 0 0 0 0 0 0 0 0 0 0 0 0 0 0 0 0 0 0 0 0 0 0 0 0 0 0 0 0 0 0 0 0 0 0 0 0 0 0 0 0 0 0 0 0 0 0 0 0 0

"11975_fr6" 0 1 0 0 0 0 0 1 0 0 1 0 0 0 0 0 1 0 0 0 1 0 0 0 0 1 1 1 0 0 3 1 1 1 0 0 0 0 0 0 0 0 0 0 0 0 0 0 0 0 0 0 0 0 0 0 0 0 0 0 0 0 0 0 0 0 0 0 0 0 0 0 0 0 0 0 0 0 0 0 0 0 0 0 0

"12002_fr2" 0 0 0 0 0 0 0 0 0 0 0 0 0 0 0 0 0 0 0 0 0 0 0 0 0 0 0 0 0 0 0 0 0 0 0 0 0 2 0 2 2 0 2 1 0 0 3 0 0 2 0 0 0 3 2 0 0 2 0 2 0 0 0 0 0 2 2 2 2 2 0 2 2 2 2 2 2 0 1 0 0 0 0 0 0

"12042_fr4" 0 0 2 0 0 0 0 3 0 0 0 0 0 0 0 0 0 0 0 1 3 0 0 0 0 1 3 0 1 0 1 4 0 0 0 0 0 0 0 0 0 0 0 0 0 0 0 0 0 0 0 0 0 0 0 0 0 0 0 0 0 0 0 0 0 0 0 0 0 0 0 0 0 0 0 0 0 0 0 0 0 0 0 0 0

"121034_fr3" 0 0 0 0 0 0 0 0 0 0 0 0 0 0 0 0 0 0 0 0 0 0 0 0 0 0 0 0 0 0 0 0 0 0 0 0 0 0 0 0 0 0 0 0 0 0 0 0 0 0 0 1 0 0 0 0 0 0 2 0 0 0 0 0 0 0 0 0 0 0 0 0 0 0 0 0 0 0 0 0 0 0 0 0 0

"121081_fr5" 0 0 0 0 0 0 0 0 0 0 0 2 0 0 0 0 4 0 0 0 0 0 0 0 0 2 0 0 0 0 0 0 0 0 0 0 0 0 0 0 0 0 0 0 0 0 0 0 0 0 0 0 0 0 0 0 0 0 0 0 0 0 0 0 0 0 0 3 2 0 1 0 0 2 1 0 0 0 0 0 2 2 2 1 2

"121109_fr1" 0 0 0 0 0 0 0 0 0 0 0 0 0 0 0 0 0 0 0 0 0 0 0 0 0 0 0 0 0 0 0 0 0 0 0 0 0 1 0 0 0 0 0 0 0 3 0 1 0 0 0 1 2 0 2 0 2 10 1 1 0 0 0 0 0 0 0 0 0 0 0 0 0 0 0 0 0 0 0 0 0 0 0 0 0

"121166_fr4" 0 0 0 0 0 0 0 0 0 0 0 0 0 0 0 0 0 0 0 0 0 0 0 0 0 0 0 2 0 0 0 0 0 0 0 0 0 0 0 0 0 0 0 0 0 0 0 0 0 0 0 0 0 0 0 0 0 0 0 0 0 0 0 0 0 0 0 0 0 0 0 0 0 0 0 0 0 0 0 0 0 0 0 0 0

"121195_fr5" 0 0 0 0 0 0 0 0 0 0 0 0 0 0 0 0 0 0 0 0 0 0 0 0 0 0 0 0 0 0 0 0 0 0 0 0 0 0 0 0 0 0 0 0 0 0 0 0 0 0 0 0 0 0 0 0 0 0 0 0 0 0 0 1 0 0 0 0 0 0 0 0 0 0 0 0 0 0 0 0 0 2 0 1 0

"121218_fr5" 12 0 8 9 5 11 3 10 2 0 5 11 0 13 3 12 12 2 2 13 5 8 4 10 8 9 4 0 3 6 9 5 3 12 6 4 2 7 0 9 1 0 0 6 0 2 3 2 2 2 1 0 0 0 0 0 3 2 0 0 0 0 0 3 7 15 12 17 15 9 3 9 10 10 9 9 11 2 25 12 5 17 21 18 17

"121249_fr2" 0 0 0 0 0 0 0 0 0 0 0 0 0 0 0 0 0 0 0 0 0 0 0 0 0 0 0 0 0 0 0 0 0 0 0 0 0 0 0 0 0 0 0 0 0 0 0 0 0 0 0 0 0 0 0 0 0 0 0 0 0 0 0 0 0 0 0 0 0 0 2 2 2 2 1 3 4 3 4 1 0 1 0 0 0

"121267_fr4" 0 0 0 0 0 0 0 0 0 0 0 0 0 0 0 0 0 2 0 0 0 0 0 0 0 0 0 0 0 0 0 0 0 0 0 0 0 0 0 0 0 0 0 0 0 0 0 0 0 0 0 0 0 0 0 0 0 0 0 0 0 0 0 0 0 0 0 0 0 0 0 0 0 0 0 0 0 0 0 0 0 0 0 0 0

"121276_fr2" 0 0 2 0 0 0 2 1 0 0 2 0 0 0 0 0 2 2 0 0 0 0 0 0 0 0 2 0 0 0 0 0 0 0 0 0 0 0 0 0 0 0 0 0 0 0 0 0 0 0 0 0 0 0 0 0 0 0 0 0 0 0 0 0 0 0 0 0 0 0 0 0 0 0 0 0 0 0 0 0 0 0 0 0 0

"121300_fr5" 3 4 4 3 5 5 2 4 3 2 2 4 2 2 4 2 4 2 5 4 6 4 2 4 5 7 6 4 6 4 5 2 5 5 4 0 0 0 0 0 0 0 0 0 0 0 0 0 0 0 0 0 0 0 0 0 0 0 0 0 0 0 0 0 0 0 0 0 0 0 0 0 0 0 0 2 4 4 4 3 3 2 3 1 3

"121324_fr1" 0 0 0 0 0 0 1 0 0 0 0 0 0 0 0 0 0 0 0 0 0 0 0 0 0 0 0 0 0 2 0 0 0 0 0 0 0 0 0 0 0 0 0 0 0 0 0 0 0 0 0 0 0 0 0 0 0 0 0 0 0 0 0 0 0 0 0 0 0 0 0 0 0 0 0 0 0 0 0 0 0 0 0 0 0

"121351_fr5" 1 2 0 0 2 3 0 0 0 0 0 2 0 4 0 0 2 0 4 3 2 3 0 0 0 2 3 0 0 2 0 4 4 0 0 0 0 0 0 0 0 0 0 0 0 0 0 0 0 0 0 0 0 0 0 0 0 0 0 0 0 0 0 0 0 0 0 0 0 0 0 0 0 0 0 3 4 4 4 2 2 0 2 1 2

"121407_fr1" 5 2 2 1 2 2 2 2 2 5 2 4 0 6 2 2 2 2 2 3 2 2 2 3 2 2 2 3 2 1 3 2 2 2 2 0 0 0 0 0 0 0 0 2 0 0 0 0 0 0 0 1 0 0 0 0 0 0 0 0 0 0 0 0 0 0 1 0 0 0 2 0 0 0 0 5 2 2 2 5 2 2 0 0 2

"12147_fr6" 0 0 0 0 0 0 0 0 0 0 0 0 0 0 0 0 0 0 0 0 0 0 0 0 0 0 0 0 0 0 0 0 0 0 0 0 0 0 0 0 0 0 0 0 0 0 0 0 0 0 0 0 0 0 0 0 0 0 0 0 0 0 0 0 0 0 2 0 0 0 0 1 0 0 1 0 0 2 4 0 0 0 0 0 0

"1216_fr3" 0 0 0 0 0 0 0 0 0 0 0 0 0 0 0 0 0 0 0 0 0 0 0 0 0 0 0 0 0 0 0 0 0 0 0 4 6 4 0 7 0 0 0 4 4 0 4 4 2 3 0 0 3 4 0 0 0 0 2 0 0 0 0 0 0 0 0 0 0 0 0 0 0 0 0 0 0 0 0 0 0 0 0 0 0

"121767_fr6" 0 0 0 0 0 0 0 0 0 0 0 0 0 0 0 0 0 0 0 0 0 0 0 0 0 0 0 0 0 0 0 0 0 0 0 0 0 0 0 0 0 0 0 0 0 0 0 0 0 0 0 0 0 4 0 0 0 4 0 0 0 0 0 0 0 0 0 0 0 0 0 0 0 0 0 0 0 0 0 0 0 0 0 0 0

"121829_fr6" 0 0 0 1 0 2 0 0 0 0 0 0 0 1 0 0 0 0 0 2 0 0 1 0 0 0 0 0 3 0 1 0 3 0 0 0 0 0 0 0 0 0 0 0 0 0 0 0 0 0 0 0 0 0 0 0 0 0 0 0 0 0 0 0 0 0 0 0 0 0 0 0 0 0 0 0 0 0 0 0 0 0 0 0 0

"121870_fr2" 0 1 1 2 1 1 0 1 1 0 2 0 0 1 2 1 1 0 1 2 0 2 2 6 2 1 2 1 4 5 0 0 2 1 2 0 0 0 0 0 0 0 0 0 0 0 0 0 0 0 0 0 0 0 0 0 0 0 0 0 0 0 0 0 0 0 0 0 0 0 0 0 0 0 0 0 0 0 0 0 0 0 0 0 0

"121871_fr3" 0 0 0 0 0 0 0 0 0 0 0 0 0 0 0 0 0 0 0 0 0 0 0 0 0 0 0 0 0 0 0 1 0 0 0 0 0 0 0 0 0 0 0 0 0 0 0 0 0 0 0 0 0 0 0 0 0 0 0 0 0 0 0 0 0 0 0 0 0 0 0 0 0 0 0 0 0 0 0 0 0 0 0 0 0

"121888_fr5" 0 0 0 0 0 0 0 0 0 0 0 0 0 2 0 13 0 0 0 0 0 0 0 0 0 0 0 0 0 0 0 1 0 0 0 0 0 0 0 0 0 0 0 0 0 0 0 0 0 0 0 0 0 0 0 0 0 0 0 0 0 0 0 0 0 0 0 0 0 0 0 0 0 0 0 0 0 0 0 0 0 0 0 0 0

"121924_fr5" 0 0 3 0 1 2 0 0 0 0 0 0 0 1 2 0 0 0 1 0 2 0 2 0 0 0 0 0 0 0 0 1 2 2 0 0 0 0 0 0 0 0 0 0 0 0 0 0 0 0 0 0 0 0 0 0 0 0 0 0 0 0 0 0 0 0 0 0 0 0 0 0 0 0 0 0 0 0 0 0 0 0 0 0 0

"121996_fr2" 0 0 0 0 1 0 0 0 0 0 0 0 0 0 0 0 0 0 0 0 0 0 0 0 0 0 0 0 0 0 0 0 0 0 0 0 0 0 0 0 0 0 0 0 0 0 0 0 0 0 0 0 0 0 0 0 0 0 0 0 0 0 0 0 0 0 0 0 0 0 0 0 0 0 0 0 0 0 0 0 0 0 0 0 0

"122312_fr4" 0 0 0 0 0 0 0 0 0 0 0 0 0 0 0 0 0 0 0 0 0 0 0 0 0 0 0 0 0 0 0 0 0 0 0 0 0 0 0 0 0 0 0 0 0 0 0 0 0 0 0 0 0 0 0 0 0 0 0 0 0 0 0 0 2 1 3 1 2 3 1 4 4 4 4 1 4 2 4 3 2 2 2 2 0

"122435_fr1" 0 0 0 0 0 0 0 0 0 0 0 0 0 0 3 1 0 0 0 2 0 0 0 0 0 0 0 0 0 0 0 0 0 0 0 0 0 0 0 0 0 0 0 0 0 0 0 0 0 0 0 0 0 0 0 0 0 0 0 0 0 0 0 0 0 0 0 0 0 0 0 0 0 0 0 0 0 0 0 0 0 0 0 0 0

"1226_fr5" 0 0 0 0 0 0 0 0 0 0 0 0 0 0 0 0 0 0 0 0 0 0 0 0 0 0 0 0 0 0 0 2 0 0 0 0 0 0 0 0 0 0 0 0 0 0 0 0 0 0 0 0 0 0 0 0 0 0 0 0 0 0 0 0 0 0 0 0 0 0 0 0 0 0 0 0 0 0 0 0 0 0 0 0 0

"12316_fr3" 3 2 0 2 3 2 2 2 0 1 2 0 0 2 2 0 0 0 0 0 0 1 0 0 0 0 0 2 0 0 0 0 0 0 0 0 0 0 0 0 0 0 0 0 0 0 0 0 0 0 0 0 0 0 0 0 0 0 0 0 0 0 0 0 0 0 0 0 0 0 0 0 0 0 0 0 0 0 0 0 0 0 0 0 0

"123314_fr2" 0 0 0 0 0 0 0 0 0 0 0 0 3 0 0 0 0 0 0 0 0 0 0 0 0 0 0 0 0 0 0 0 0 0 0 0 0 0 0 0 0 0 0 0 0 0 0 0 0 0 0 0 0 0 0 0 0 0 0 0 0 0 0 0 0 0 0 0 0 0 0 0 0 0 0 0 0 0 0 0 0 0 0 0 0

"124_fr5" 0 0 3 0 1 0 0 1 0 0 0 3 0 0 0 0 0 0 0 0 0 0 1 0 0 0 0 0 0 0 0 0 0 0 0 0 0 0 0 0 0 0 0 0 0 0 0 0 0 0 0 0 0 0 0 0 0 0 0 0 0 0 0 0 0 0 0 1 1 0 0 0 0 0 0 0 0 0 0 1 0 0 0 0 0

"12402_fr1" 0 1 0 0 5 0 0 0 0 0 3 6 0 6 4 8 13 0 2 9 4 8 7 0 0 2 10 5 9 12 0 3 8 6 0 0 0 0 0 4 0 0 0 0 6 0 3 2 2 4 3 0 0 6 4 0 0 2 0 0 0 0 0 0 4 0 2 3 2 1 0 0 0 0 0 0 0 2 1 3 0 0 0 0 0

"124382_fr4" 0 0 0 0 0 0 6 0 0 0 0 0 0 0 0 0 0 0 0 0 0 0 0 0 0 0 0 0 0 0 0 0 0 0 0 0 0 0 0 0 0 0 0 0 0 0 0 0 0 0 0 0 0 0 0 0 0 0 0 0 0 0 0 0 0 0 0 0 0 0 0 0 0 0 0 0 0 0 0 0 0 0 0 0 0

"12473_fr6" 0 0 0 0 0 0 0 0 0 0 0 0 0 0 0 0 0 0 0 0 0 0 0 0 0 0 0 0 0 0 0 0 0 0 0 2 3 3 5 5 3 5 1 23 19 10 11 24 11 20 7 10 13 8 7 6 11 7 7 11 5 3 3 6 5 6 2 3 3 2 5 3 2 3 4 4 3 3 2 2 2 2 2 2 2

"125545_fr3" 0 0 3 0 0 0 1 0 4 0 2 0 4 0 2 3 0 0 0 0 0 0 0 0 0 0 0 0 0 0 0 0 0 0 1 0 3 0 0 0 0 0 0 0 0 0 0 0 0 0 0 0 0 0 0 0 0 0 0 0 0 0 0 0 0 0 0 0 0 0 0 0 0 0 0 3 1 0 0 0 0 0 0 0 0

"12555_fr1" 0 0 0 0 0 2 2 0 0 0 0 0 0 0 4 1 0 0 0 0 0 0 0 0 0 0 4 0 0 0 0 0 0 0 0 4 2 2 0 0 0 0 0 3 0 0 0 0 0 0 0 0 0 0 1 0 0 0 0 0 0 0 0 0 0 0 0 0 0 0 0 0 0 0 0 0 0 0 0 0 0 0 0 0 0

"1256_fr1" 0 0 2 0 0 0 1 0 0 1 0 0 0 0 0 0 0 0 0 0 0 0 0 0 1 0 0 0 0 0 1 0 0 0 1 0 0 0 0 0 0 0 0 0 0 0 0 0 0 0 0 0 0 0 0 0 0 0 0 0 0 0 0 0 0 0 0 0 0 1 0 0 0 0 0 0 0 0 0 0 2 0 0 0 0

"125607_fr6" 0 0 0 0 0 0 0 0 0 0 0 0 0 0 0 0 0 0 0 0 0 0 0 0 0 0 0 0 0 0 0 0 0 0 0 0 0 0 0 0 0 0 0 0 0 0 0 0 0 0 0 0 0 0 0 0 0 0 0 0 0 0 0 1 0 0 0 0 0 0 0 0 0 0 0 0 1 0 0 0 0 0 0 0 0

"125644_fr6" 0 0 0 0 0 0 0 0 0 0 2 0 0 3 0 0 0 0 3 2 0 0 0 0 0 1 0 0 0 5 0 0 0 1 0 0 0 0 0 0 0 0 0 0 0 0 0 0 0 0 0 0 0 0 0 0 0 0 0 0 0 0 0 0 0 0 0 0 0 0 0 0 0 0 0 0 0 0 0 0 0 0 0 0 0

"125653_fr4" 0 0 0 0 0 0 0 0 0 0 0 0 0 0 0 0 0 0 0 0 0 0 0 0 0 0 0 0 0 0 0 0 0 0 0 0 0 0 0 0 1 0 1 0 0 0 2 0 0 1 1 0 0 0 1 0 0 0 0 0 0 0 0 0 0 0 0 0 0 0 0 0 0 0 0 0 0 0 0 0 0 0 0 0 0

"125702_fr2" 0 0 0 0 0 0 0 0 0 0 0 0 0 0 0 0 0 0 0 0 0 0 0 0 0 0 0 0 0 0 0 2 0 0 0 0 0 0 0 0 0 0 0 0 0 0 0 0 0 0 0 0 0 0 0 0 0 0 0 0 0 0 0 0 0 0 0 0 0 0 0 0 0 0 0 0 0 0 0 0 0 0 0 0 0

"12571_fr5" 0 0 0 0 0 0 0 0 0 2 0 0 0 0 0 0 0 0 0 0 0 0 0 0 4 0 0 0 0 0 0 3 0 0 0 0 0 0 0 0 0 0 0 0 0 0 0 0 0 0 0 0 0 0 0 0 0 0 0 0 0 0 0 0 0 0 0 0 0 0 0 0 0 0 0 0 0 0 0 0 0 0 0 0 0

"125730_fr1" 2 2 0 0 0 0 0 0 0 0 0 0 0 0 0 3 0 1 0 0 3 2 4 0 0 3 3 0 2 3 1 4 0 2 0 0 0 0 0 0 0 0 0 0 0 0 0 0 0 0 0 0 0 0 0 0 0 0 0 0 0 0 0 0 0 0 0 0 0 0 0 0 0 0 0 0 0 0 0 0 0 0 0 0 0

"125764_fr5" 2 0 0 2 0 3 3 2 2 2 3 0 4 2 2 2 4 5 3 5 3 2 0 2 2 0 0 2 2 0 0 0 6 3 2 0 0 0 0 0 0 0 0 0 0 0 0 0 0 0 0 0 0 0 0 0 0 0 0 0 0 0 0 0 0 0 0 0 0 0 0 0 2 1 2 1 1 2 3 2 1 1 2 2 2

"125789_fr1" 0 0 0 0 0 0 0 0 0 0 2 0 0 1 1 0 2 0 1 1 1 0 0 2 0 1 0 0 3 2 0 2 2 4 0 0 0 0 0 0 0 0 0 0 0 0 0 0 0 0 0 0 0 0 0 0 0 0 0 0 0 0 0 0 0 0 0 0 1 0 3 2 2 8 2 2 3 2 3 3 0 1 4 3 1

"125938_fr2" 0 0 0 0 0 0 0 0 0 0 0 0 0 7 0 0 0 0 0 0 0 0 0 0 0 0 0 0 0 0 0 0 0 0 0 0 0 0 0 0 0 0 0 0 0 0 0 0 0 0 0 0 0 0 0 0 0 0 0 0 0 0 0 0 0 0 0 0 0 0 0 0 0 0 0 0 0 0 0 0 0 0 0 0 0

"12596_fr4" 0 0 0 0 0 0 0 0 0 3 0 0 0 0 0 0 0 0 0 0 0 0 0 0 0 0 0 0 0 0 0 0 0 0 0 0 0 0 0 0 0 0 0 0 0 0 0 0 0 0 0 0 0 0 0 0 0 0 0 0 0 0 0 0 0 0 0 0 0 0 0 0 0 0 0 0 0 0 0 0 0 0 0 0 0

"126060_fr4" 0 0 2 6 0 1 0 0 0 0 0 0 0 2 0 0 0 0 0 1 0 0 2 0 0 2 2 0 0 1 0 0 0 0 0 0 0 0 0 0 0 0 0 0 0 0 0 0 0 0 0 0 0 0 0 0 0 0 0 0 0 0 0 0 0 0 0 0 0 0 0 0 0 0 0 0 0 0 0 0 0 0 0 0 0

"126175_fr5" 1 0 0 2 0 0 0 0 1 0 1 0 0 0 2 0 0 1 0 0 0 0 0 0 1 0 0 0 0 0 0 0 0 0 0 0 0 0 0 0 0 0 0 0 0 0 0 0 0 0 0 0 0 0 0 0 0 0 0 0 0 0 0 0 0 0 0 0 0 0 0 0 0 0 0 0 0 0 0 0 0 0 0 0 0

"12649_fr1" 0 0 0 0 0 0 0 0 0 0 0 0 0 0 0 0 0 0 0 0 0 0 0 0 0 0 6 0 0 0 0 0 0 0 0 0 0 0 0 0 0 0 0 0 0 0 0 0 0 0 0 0 0 0 0 0 0 0 0 0 0 0 0 0 0 0 0 0 0 0 0 0 0 0 0 0 0 0 0 0 0 0 0 0 0

"12681_fr4" 0 0 0 0 0 0 0 0 0 0 0 0 0 0 0 0 0 0 0 0 0 0 0 0 7 0 0 0 0 0 5 2 0 0 1 0 0 0 0 0 0 0 0 0 0 0 0 0 0 0 0 0 0 0 0 0 0 0 0 0 0 0 0 0 0 0 0 0 0 0 0 0 0 0 0 0 0 0 0 0 0 0 0 0 0

"1277_fr6" 0 0 0 2 0 0 0 0 0 0 0 0 0 0 0 0 2 0 0 0 0 0 0 0 0 0 0 0 0 0 0 0 0 0 0 0 0 0 0 0 0 0 0 0 0 0 0 0 0 0 0 0 0 0 0 0 0 0 0 0 0 0 0 0 0 0 0 0 0 0 0 0 0 0 0 0 0 0 0 0 0 0 0 0 0

"12830_fr2" 0 0 0 0 0 0 0 0 0 0 0 0 0 0 0 0 0 0 2 0 0 0 2 0 0 0 0 0 0 0 0 0 0 0 0 0 0 0 0 0 0 0 0 0 0 0 0 0 0 0 0 0 0 0 0 0 0 0 0 0 0 0 0 0 0 0 0 0 0 0 0 0 0 0 0 0 0 0 0 0 0 0 0 0 0

"12849_fr2" 0 0 0 0 0 0 0 0 0 0 0 0 0 0 0 0 0 0 0 0 0 0 0 0 0 0 0 0 0 0 0 0 0 0 0 0 1 2 13 2 4 0 11 5 10 33 11 8 37 13 7 15 11 10 13 9 11 10 14 14 8 11 12 10 8 0 0 0 7 5 0 0 0 0 0 0 0 0 0 0 0 0 0 0 2

"128499_fr4" 0 1 2 0 1 1 0 0 1 2 0 0 0 0 0 0 0 0 0 0 2 0 2 0 2 2 7 1 2 0 2 2 2 1 0 0 0 0 0 0 0 0 0 0 0 0 0 0 0 0 0 0 0 0 0 0 0 0 0 0 0 0 0 0 0 0 0 0 0 0 0 0 0 0 0 0 0 0 0 0 0 0 0 0 0

"128511_fr6" 0 0 8 6 0 0 0 11 0 12 6 0 0 0 0 0 0 0 0 0 10 0 10 11 12 11 8 0 12 0 10 12 0 0 0 0 0 0 0 0 0 0 0 0 0 0 0 0 0 0 0 0 0 0 0 0 0 0 0 0 3 2 2 3 2 0 8 8 0 5 0 0 0 0 0 0 0 0 0 0 0 0 0 0 0

"12885_fr4" 0 0 0 0 0 0 0 0 0 0 0 0 0 0 0 0 0 0 0 0 0 0 0 0 0 0 0 0 0 0 0 0 0 0 0 0 0 0 15 2 2 5 7 2 2 2 4 2 2 2 3 2 2 3 2 5 2 4 5 11 4 4 4 4 19 3 0 5 0 6 0 0 0 0 0 0 0 0 0 0 0 0 0 0 0

"129_fr5" 0 0 0 0 0 0 0 0 0 0 0 0 0 1 0 0 0 0 0 0 0 0 0 0 0 0 0 0 0 0 0 0 0 0 0 0 0 0 0 0 0 0 0 0 0 0 0 0 0 0 0 0 0 0 0 0 0 0 0 0 0 0 0 0 0 0 0 0 0 0 0 0 0 0 0 0 0 0 0 0 0 0 0 0 0

"12901_fr5" 0 0 0 0 0 0 0 0 0 2 0 0 0 0 0 0 0 0 0 0 0 0 0 0 0 0 0 0 0 0 0 0 0 0 0 0 0 0 0 0 0 0 0 0 0 0 0 0 0 0 0 0 0 0 0 0 0 0 0 0 0 0 0 0 0 0 0 0 0 0 0 0 0 0 0 0 0 0 0 0 0 0 0 0 0

"129051_fr2" 1 2 3 3 2 0 1 2 0 2 2 2 0 2 3 0 2 0 3 2 1 2 1 2 0 1 2 0 2 0 1 0 0 4 2 0 0 0 0 0 0 0 0 0 0 0 0 0 0 0 0 0 0 0 0 0 0 0 0 0 0 0 0 0 0 0 0 0 0 0 0 0 0 0 0 0 0 0 0 0 0 0 0 0 0

"12912_fr4" 0 0 0 0 0 0 0 0 0 0 0 1 0 0 0 0 0 0 0 0 0 0 0 0 0 0 0 0 1 0 0 0 0 0 0 0 0 0 0 0 0 0 0 0 0 0 0 0 0 0 0 0 0 0 0 0 0 0 0 0 0 0 0 0 0 0 0 0 0 0 0 0 0 0 0 0 0 0 0 0 0 0 0 0 0

"12917_fr4" 0 0 0 0 0 0 0 0 0 0 0 0 0 0 0 0 0 0 0 0 0 0 0 0 0 0 0 0 0 0 0 0 0 0 0 0 0 0 0 0 0 0 0 10 0 0 0 7 0 0 0 0 2 0 4 0 0 0 0 0 0 0 0 0 0 0 0 0 0 0 0 0 0 0 0 0 0 0 0 0 0 0 0 0 0

"12941_fr2" 1 0 2 2 2 2 0 2 1 0 2 2 0 2 2 0 1 0 2 2 2 0 3 4 0 2 4 1 2 0 1 3 2 0 1 0 0 0 0 0 0 0 0 0 0 0 0 0 0 0 0 0 0 0 0 0 0 0 0 0 0 0 0 0 0 0 0 0 0 0 0 0 0 1 1 2 2 0 0 2 3 0 2 1 2

"1295_fr5" 0 0 0 0 2 2 0 0 0 0 2 0 0 3 0 0 0 0 0 0 0 0 1 0 1 0 0 0 0 2 0 0 0 0 0 0 0 0 0 0 0 0 0 0 0 0 0 0 0 0 0 0 0 0 0 0 0 0 0 0 0 0 0 0 0 0 0 0 0 0 0 0 0 0 0 0 0 0 0 0 0 0 0 0 0

"129687_fr3" 0 0 2 1 0 1 2 2 0 1 1 0 0 6 0 0 2 0 0 1 1 1 0 2 2 0 2 0 0 0 1 0 1 1 0 0 0 0 0 0 0 0 0 0 0 0 0 0 0 0 0 0 0 0 0 0 0 0 0 0 0 0 0 0 0 0 0 0 0 0 0 0 0 0 0 0 0 0 0 0 0 0 0 0 0

"129814_fr2" 0 0 2 3 0 0 0 2 0 0 0 0 0 0 0 0 0 0 0 0 2 0 0 0 0 0 2 0 2 0 0 0 0 3 0 0 0 0 0 0 0 0 0 0 0 0 0 0 0 0 0 0 0 0 0 0 0 0 0 0 0 0 0 0 0 0 0 0 0 0 0 0 0 0 0 0 0 0 0 0 0 0 0 0 0

"129831_fr3" 0 0 0 0 2 0 1 0 0 1 0 0 0 2 0 1 3 0 1 0 0 0 1 0 0 4 1 0 4 0 1 2 1 0 0 0 0 0 0 0 0 0 0 0 0 0 0 0 0 0 0 0 0 0 0 0 0 0 0 0 0 0 0 0 0 0 1 3 4 0 5 6 4 5 5 6 4 6 6 0 4 3 3 1 4

"129865_fr5" 0 0 0 0 0 0 0 0 0 0 0 0 0 0 0 0 0 0 0 0 0 0 0 0 0 0 0 0 0 0 0 0 0 0 0 1 2 2 0 2 0 0 0 1 0 0 0 0 3 0 0 0 0 0 0 0 0 2 0 2 0 0 0 0 0 0 0 0 0 0 0 0 0 0 0 0 0 0 0 0 0 0 0 0 0

"129908_fr3" 0 0 0 0 0 0 0 0 0 0 0 0 0 0 0 0 0 0 0 0 0 0 0 0 0 0 0 0 0 0 0 0 0 0 0 0 0 0 1 3 0 0 1 4 2 0 2 0 1 2 2 0 1 2 2 2 0 0 0 1 0 0 0 0 0 0 0 0 0 0 0 0 0 0 0 0 0 0 0 0 0 0 0 0 0

"129916_fr5" 0 0 0 0 0 0 0 0 0 0 0 0 0 0 0 0 0 0 0 0 0 0 0 0 0 0 0 0 0 0 0 0 0 0 0 0 0 0 0 0 0 0 0 0 0 0 0 0 0 0 0 0 0 0 0 0 0 0 0 0 0 0 0 0 0 0 3 0 2 0 0 0 0 3 3 0 0 0 0 0 0 0 0 0 0

"129929_fr3" 0 0 0 0 0 0 0 0 0 0 0 0 0 0 0 0 0 0 0 0 0 0 1 0 0 0 0 0 0 0 0 0 0 0 0 0 0 0 0 0 0 0 0 0 0 0 0 0 0 0 0 0 0 0 0 0 0 0 0 0 0 0 0 0 0 0 0 0 0 0 0 0 0 0 0 0 0 0 0 0 0 0 0 0 0

"129960_fr4" 0 0 0 0 0 0 0 0 0 4 0 0 0 0 0 0 0 0 0 0 0 0 0 0 0 0 0 0 0 0 0 0 0 0 0 0 0 0 0 0 0 0 0 0 0 0 0 0 0 0 0 0 0 0 0 0 0 0 0 0 0 0 0 0 0 0 0 0 0 0 0 0 0 0 0 0 0 0 0 0 0 0 0 0 0

"130021_fr5" 0 0 0 0 0 0 0 0 0 0 0 0 0 0 0 0 0 0 0 0 0 0 0 0 0 0 0 0 0 0 0 0 0 0 0 0 0 0 0 0 0 0 0 0 0 0 0 0 0 0 0 0 0 0 0 0 0 0 0 0 0 0 0 0 0 0 0 0 0 0 2 0 0 0 0 0 2 0 0 0 0 0 0 0 0

"130034_fr5" 0 2 4 2 4 4 0 2 2 0 3 4 0 2 2 2 2 0 4 4 2 2 4 4 2 4 4 2 4 17 2 4 4 4 2 0 0 0 0 0 0 0 0 0 1 0 1 0 0 0 3 0 0 0 1 0 0 0 0 0 0 0 0 0 0 0 0 0 0 0 0 0 0 0 0 0 0 0 0 0 0 0 0 0 0

"130036_fr1" 0 0 0 0 3 0 0 0 0 0 0 0 0 2 0 0 1 0 1 2 0 2 4 0 0 3 0 0 0 2 0 2 0 1 0 0 0 0 0 0 0 0 0 0 0 0 0 0 0 0 0 0 0 0 0 0 0 0 0 0 0 0 0 0 0 1 0 3 0 0 2 0 4 3 2 3 5 2 4 3 0 0 1 0 1

"130062_fr5" 0 0 0 0 0 0 0 0 0 0 0 0 0 1 0 0 0 0 1 0 0 0 0 0 0 0 0 0 0 0 0 1 0 0 0 0 0 0 0 0 0 0 0 0 0 0 0 0 0 0 0 0 0 0 0 0 0 0 0 0 0 0 0 0 0 0 0 0 0 0 0 0 0 0 0 0 0 0 0 0 0 0 0 0 0

"130066_fr3" 0 0 0 0 0 0 0 0 0 0 0 0 0 0 0 0 0 0 0 0 0 0 0 0 0 0 0 0 2 0 0 1 0 0 0 0 0 0 0 0 0 0 0 0 0 0 0 0 0 0 0 0 0 0 0 0 0 0 0 0 0 0 0 0 0 0 0 0 0 0 0 0 0 0 0 0 0 0 0 0 0 0 0 0 0

"130094_fr1" 0 0 0 0 0 0 0 0 0 0 0 0 0 0 0 0 0 0 0 0 0 0 0 0 0 0 0 0 0 0 0 0 0 0 0 0 0 0 0 0 0 0 0 0 0 0 0 0 0 0 0 0 0 0 2 0 0 0 0 0 0 0 0 0 0 0 0 0 0 0 0 0 0 0 0 0 0 0 0 0 0 0 0 0 0

"130139_fr1" 0 2 6 6 6 0 3 2 4 6 6 2 8 2 2 4 4 5 2 4 2 2 4 2 0 2 2 6 4 3 4 7 5 2 3 0 0 0 0 0 0 0 0 0 0 0 0 0 0 0 0 0 0 0 0 0 0 0 0 0 0 0 0 0 0 0 0 0 0 0 0 0 0 0 0 0 0 3 0 0 0 2 2 1 2

"130148_fr4" 0 1 7 0 0 0 0 4 0 0 0 1 0 0 0 0 0 0 0 2 2 0 6 0 0 3 6 0 2 0 4 3 3 1 0 0 0 0 0 0 0 0 0 0 0 0 0 0 0 0 0 0 0 0 0 0 0 0 0 0 0 0 0 0 0 0 0 0 0 0 0 0 0 0 0 0 0 0 0 0 0 0 0 0 0

"130214_fr4" 2 5 1 0 3 1 4 5 2 0 0 2 0 2 0 5 2 0 0 2 0 2 0 2 0 4 2 1 0 4 3 4 2 2 2 0 0 0 0 0 0 0 0 0 0 0 0 0 0 0 0 0 0 0 0 0 0 0 0 0 0 0 0 0 0 0 2 0 2 2 3 0 0 0 0 0 3 3 6 0 0 0 0 0 0

"130227_fr1" 0 0 0 0 0 0 0 0 0 0 0 0 0 0 0 0 0 0 0 0 0 0 0 0 0 0 0 0 0 0 0 0 0 0 0 1 0 0 0 1 0 0 0 0 0 0 0 0 0 0 0 0 0 0 0 0 0 0 0 0 0 0 0 0 0 0 0 0 0 0 0 0 0 0 0 0 0 0 0 0 0 0 0 0 0

"130236_fr5" 0 0 0 0 0 0 0 0 1 0 0 0 0 0 1 0 0 0 0 0 0 0 0 0 0 0 0 2 0 0 0 0 0 0 0 0 0 0 0 0 0 0 0 0 0 0 0 0 0 0 0 0 0 0 0 0 0 0 0 0 0 0 0 0 0 0 0 0 0 0 0 0 0 0 0 0 0 0 0 0 0 0 0 0 0

"13026_fr5" 0 0 0 0 0 0 0 0 0 0 0 0 0 0 0 0 0 0 0 0 0 0 1 0 0 0 0 0 1 0 0 0 0 0 0 0 0 0 0 0 0 0 0 0 0 0 0 0 0 0 0 0 0 0 0 0 0 0 0 0 0 0 0 0 0 0 0 0 0 0 0 0 0 0 0 0 0 0 0 0 0 0 0 0 0

"130339_fr1" 0 0 0 0 0 0 0 0 0 0 0 0 0 0 0 0 0 0 0 0 0 0 0 0 0 0 0 0 0 0 0 0 0 0 0 0 0 0 0 0 0 0 0 0 0 0 0 0 0 0 0 0 0 0 0 3 0 0 0 0 0 0 0 0 0 0 0 0 0 0 0 0 0 0 0 0 0 0 0 0 0 0 0 0 0

"13039_fr2" 0 0 0 0 0 0 0 0 0 0 0 0 0 0 0 0 3 0 0 0 0 0 0 1 0 2 0 0 0 0 0 0 0 0 0 0 0 0 0 0 0 0 0 0 0 0 0 0 0 0 0 0 0 0 0 0 0 0 0 0 0 0 0 0 0 0 0 0 0 0 0 0 0 0 0 0 0 0 0 0 0 0 0 0 0

"130435_fr3" 0 0 0 0 0 0 1 0 0 0 0 0 0 0 0 0 0 0 0 0 0 0 0 0 0 0 0 0 0 0 0 0 0 0 0 0 0 0 0 0 0 0 0 0 0 0 0 0 0 0 0 0 0 0 0 0 0 0 0 0 0 0 0 0 0 0 0 0 0 0 0 0 0 0 0 0 0 0 0 0 0 0 0 0 0

"130876_fr2" 0 0 0 0 0 0 0 0 0 0 2 0 0 0 0 0 0 0 0 0 0 0 0 0 0 0 0 0 0 0 0 0 0 0 0 0 0 0 0 0 0 0 0 0 0 0 0 0 0 0 0 0 0 0 0 0 0 0 0 0 0 0 0 0 0 0 0 0 0 0 0 0 0 0 0 0 0 0 0 0 0 0 0 0 0

"13125_fr5" 5 2 2 0 1 0 0 1 0 0 1 0 0 0 0 0 0 0 0 0 0 1 0 2 0 1 0 0 0 0 0 1 0 0 0 0 0 0 0 0 0 0 0 0 0 0 0 0 0 0 0 0 0 0 0 0 0 0 0 0 0 0 0 0 0 0 0 0 0 0 0 0 0 0 0 0 0 0 0 0 0 0 0 0 0

"13141_fr2" 0 0 0 2 0 0 0 0 0 0 0 0 0 0 0 0 0 0 0 0 0 0 1 2 0 0 2 0 3 1 0 0 0 0 0 0 0 0 0 0 0 0 0 0 0 0 0 0 0 0 0 0 0 0 0 0 0 0 0 0 0 0 0 0 0 0 0 0 0 0 0 0 0 0 0 0 0 0 0 0 0 0 0 0 0

"13142_fr5" 6 1 2 0 10 0 6 0 3 0 0 7 4 9 0 5 6 0 7 2 2 6 4 5 0 4 6 6 2 4 0 2 2 8 0 0 0 0 0 4 0 0 0 0 3 0 0 0 0 0 0 0 0 0 0 0 0 0 0 0 0 0 0 0 0 3 0 5 0 0 0 0 0 0 1 0 1 0 0 1 0 0 0 0 0

"13187_fr6" 3 5 3 0 4 3 4 0 0 0 0 0 0 0 0 0 3 0 0 0 0 3 0 10 3 2 2 0 0 3 0 3 0 3 0 0 0 0 0 0 0 0 0 0 0 0 0 0 0 0 0 0 0 0 0 0 0 0 0 0 0 0 0 0 0 0 0 0 0 0 0 0 0 0 0 0 0 0 0 0 0 0 0 0 0

"132388_fr1" 0 0 0 0 0 0 0 0 0 0 0 0 0 0 0 0 0 0 3 0 0 0 0 0 0 0 0 0 0 0 0 0 0 0 0 0 0 0 0 0 0 0 0 0 0 0 0 0 0 0 0 0 0 0 0 0 0 0 0 0 0 0 0 0 0 0 0 0 0 0 0 0 0 0 0 0 0 0 0 0 0 0 0 0 0

"13250_fr6" 20 22 16 16 23 19 24 21 21 14 18 22 29 26 20 27 25 27 29 25 23 23 26 27 25 27 25 23 26 33 23 31 23 24 23 3 3 9 4 12 8 0 8 11 12 7 9 8 7 8 11 9 4 8 11 12 11 10 9 8 7 5 8 4 8 17 21 20 17 11 18 20 20 21 20 18 23 23 25 18 16 16 21 19 22

"133468_fr5" 0 0 2 0 1 0 0 0 0 0 0 0 0 0 2 0 0 0 0 0 0 0 0 0 0 2 3 0 0 0 0 3 0 1 0 0 0 0 0 0 0 0 0 0 0 0 0 0 0 0 0 0 0 0 0 0 0 0 0 0 0 0 0 0 0 0 0 0 0 0 0 0 0 0 0 0 0 0 0 0 0 0 0 0 0

"133650_fr5" 0 0 0 0 0 6 0 1 0 0 0 0 0 0 0 0 0 0 0 0 0 0 0 0 0 0 0 0 0 0 0 0 0 0 0 0 2 1 0 2 0 0 0 0 0 0 0 0 0 0 0 0 0 0 0 0 0 0 0 0 0 0 0 0 0 0 0 0 0 0 0 0 0 0 0 0 0 0 0 0 0 0 0 0 0

"133670_fr2" 0 0 0 0 0 1 0 2 0 0 0 0 0 1 0 0 0 0 3 2 2 1 3 0 2 0 0 0 0 3 0 2 3 0 0 0 2 0 0 1 0 0 0 0 0 0 0 0 0 1 0 0 0 0 1 0 0 0 0 0 0 0 0 0 0 0 0 0 0 0 0 0 0 0 0 0 0 0 0 0 0 0 0 0 0

"133685_fr4" 2 3 3 2 1 0 0 0 2 0 0 0 0 1 2 0 0 0 1 1 0 0 1 4 0 0 1 0 0 0 0 0 0 0 0 0 0 0 0 0 0 0 0 0 0 0 0 0 0 0 0 0 0 0 0 0 0 0 0 0 0 0 0 0 0 0 0 0 0 0 0 0 0 0 0 0 0 0 0 0 0 0 0 0 0

"133838_fr6" 0 0 0 0 0 0 0 0 0 0 0 0 0 0 0 0 0 0 1 0 0 0 0 0 0 0 0 0 0 1 0 0 0 0 0 0 0 0 0 0 0 0 0 0 0 0 0 0 0 0 0 0 0 0 0 0 0 0 0 0 0 0 0 0 0 0 0 0 0 0 0 0 0 0 0 0 0 0 0 0 0 0 0 0 0

"133881_fr1" 0 0 0 0 0 0 0 0 0 0 0 0 0 0 0 0 0 0 0 0 0 0 0 0 0 0 1 0 1 0 0 0 0 0 0 0 0 0 0 0 0 0 0 0 0 0 0 0 0 0 0 0 0 0 0 0 0 0 0 0 0 0 0 0 0 0 0 0 0 0 0 0 0 0 0 0 0 0 0 0 0 0 0 0 0

"133946_fr2" 0 0 0 0 0 0 0 0 0 0 0 0 0 0 0 0 0 0 0 0 0 0 0 0 0 0 0 0 0 0 0 0 0 0 0 0 0 0 0 0 0 0 0 2 0 0 0 0 0 0 0 2 0 2 2 0 0 4 1 0 0 0 0 0 0 0 0 0 0 0 0 0 0 0 0 0 0 0 0 0 0 0 0 0 0

"133946_fr3" 4 2 0 0 0 3 0 0 0 0 0 3 0 0 0 0 0 0 4 0 1 1 0 0 0 0 2 0 0 0 2 0 1 2 2 0 0 0 0 0 0 0 0 0 0 0 0 0 0 0 0 0 0 0 0 0 0 0 0 0 0 2 0 0 0 0 2 0 0 1 0 2 0 0 0 0 4 0 0 5 3 6 0 3 4

"134128_fr2" 0 0 0 0 0 0 0 0 0 0 0 0 0 0 0 0 0 0 0 0 0 0 0 0 0 0 0 0 0 0 0 0 0 0 0 4 0 0 0 5 0 0 0 0 0 0 0 0 0 0 0 0 0 0 0 0 0 0 0 0 0 0 0 0 0 0 0 0 0 0 0 0 0 0 0 0 0 0 0 0 0 0 0 0 0

"134170_fr1" 0 0 2 0 0 0 0 0 0 0 0 0 0 0 0 0 0 0 0 0 0 0 0 0 0 0 0 0 0 3 0 0 0 0 0 0 0 0 0 0 0 0 0 0 0 0 0 0 0 0 0 0 0 0 0 0 0 0 0 0 0 0 0 0 0 0 0 0 0 0 0 0 0 0 0 0 0 0 0 0 0 0 0 0 0

"134287_fr6" 0 0 0 0 0 0 0 0 0 0 0 0 0 0 0 0 0 0 0 0 2 0 0 0 0 2 0 0 0 0 0 0 0 0 0 0 0 0 0 0 0 0 0 0 0 0 0 0 0 0 0 0 0 0 0 0 0 0 0 0 0 0 0 0 0 0 0 0 0 0 0 0 0 0 0 0 0 0 0 0 0 0 0 0 0

"1344_fr4" 1 2 2 0 1 1 0 1 2 0 4 4 1 6 2 4 1 0 2 1 3 0 3 4 0 4 6 1 4 4 4 5 5 1 1 0 0 0 0 0 0 0 0 0 0 0 0 0 0 0 0 0 0 0 0 0 0 0 0 0 0 0 0 0 0 0 0 0 0 0 0 0 0 0 0 0 0 0 0 0 0 0 0 0 0

"134418_fr1" 0 0 0 0 0 0 0 0 0 0 0 0 0 0 0 0 0 0 0 0 0 0 0 2 0 0 0 0 0 0 0 0 0 0 0 0 0 0 0 0 0 0 0 0 0 0 0 0 0 0 0 0 0 0 0 0 0 0 0 0 0 0 0 0 0 0 0 0 0 0 0 0 0 0 0 0 0 0 0 0 0 0 0 0 0

"135140_fr6" 0 0 2 0 0 0 0 0 0 0 0 0 0 0 3 0 0 0 0 0 0 0 0 0 0 0 0 0 0 0 0 0 0 0 0 0 0 0 0 0 0 0 0 0 0 0 0 0 0 0 0 0 0 0 0 0 0 0 0 0 0 0 0 0 0 0 0 0 0 0 0 0 0 0 0 0 0 0 0 0 0 0 0 0 0

"135282_fr4" 0 0 0 0 0 0 0 0 0 0 0 0 0 0 0 0 0 0 0 0 0 0 0 0 0 0 0 0 0 0 0 0 0 0 0 0 0 0 0 0 0 0 0 0 0 0 0 0 0 0 0 1 0 0 3 0 0 0 0 0 0 0 0 0 0 0 0 0 0 0 0 0 0 0 0 0 0 0 0 0 0 0 0 0 0

"135405_fr3" 0 0 0 1 0 0 0 0 0 0 0 0 0 0 0 0 0 0 0 0 0 1 0 0 1 0 0 0 0 0 0 0 0 0 3 0 0 0 0 0 0 0 0 0 0 0 0 0 0 0 0 0 0 0 0 0 0 0 0 0 0 0 0 0 0 0 0 0 0 0 0 0 0 0 0 0 0 0 0 0 0 0 0 0 0

"135487_fr5" 10 6 7 8 6 7 3 6 0 6 8 9 0 7 8 6 7 5 6 9 11 6 9 8 6 7 2 6 7 8 9 7 7 8 9 3 3 4 0 2 0 0 0 0 0 0 0 0 0 0 0 0 0 0 0 0 0 0 0 0 0 0 0 0 0 0 0 0 0 0 0 0 0 0 0 0 0 0 0 0 0 0 0 0 0

"1366_fr1" 0 0 0 0 0 0 0 0 0 0 0 0 0 0 0 0 0 0 0 0 0 0 0 0 0 0 0 0 2 0 0 0 0 0 2 0 0 0 0 0 0 0 0 0 0 0 0 0 0 0 0 0 0 0 0 0 0 0 0 0 0 0 0 0 0 0 0 0 0 0 0 0 0 0 0 0 0 0 0 0 0 0 0 0 0

"136881_fr5" 1 0 0 0 0 0 0 0 0 0 0 0 0 0 0 0 0 0 0 0 0 0 0 0 0 0 0 0 0 0 0 0 0 0 0 0 0 0 0 0 0 0 0 0 0 0 0 0 0 0 1 0 0 0 0 0 0 2 0 1 0 0 0 0 0 0 0 0 0 0 0 0 0 0 0 0 0 0 0 0 0 0 0 0 0

"137335_fr1" 0 0 0 0 0 0 0 0 0 0 0 2 0 0 0 0 0 0 0 0 0 0 0 0 4 0 0 0 0 0 4 0 0 0 2 0 0 0 0 0 0 0 0 0 0 0 0 0 0 0 0 0 0 0 0 0 0 0 0 0 0 0 0 0 0 0 0 0 0 0 0 0 0 0 0 0 0 0 0 0 0 0 0 0 0

"137345_fr3" 0 0 0 0 0 0 0 0 0 0 0 0 0 0 0 0 0 0 0 0 0 0 0 0 0 0 0 0 0 0 0 0 0 0 0 1 8 0 0 6 3 0 1 0 0 1 0 1 2 0 8 0 4 1 0 0 3 0 2 1 4 0 4 1 0 1 2 5 0 0 0 0 0 0 0 0 0 0 0 0 0 1 0 0 0

"137367_fr3" 0 0 0 0 0 0 0 0 0 0 0 0 0 0 0 0 0 0 0 0 0 0 0 0 0 0 1 0 3 0 0 0 0 0 0 0 0 0 0 0 0 0 0 0 0 0 0 0 0 0 0 0 0 0 0 0 0 0 0 0 0 0 0 0 0 0 0 0 0 0 0 0 0 0 0 0 0 0 0 0 0 0 0 0 0

"137406_fr3" 0 0 0 0 0 0 0 0 0 1 0 0 0 0 0 0 0 2 0 0 0 0 0 0 0 0 0 0 0 0 0 0 0 0 0 0 0 0 0 0 0 0 0 0 0 0 0 0 0 0 0 0 0 0 0 0 0 0 0 0 0 0 0 0 0 0 0 0 0 0 0 0 0 0 0 0 0 0 0 0 0 0 0 0 0

"137567_fr1" 0 0 0 0 0 0 0 0 0 0 0 0 0 0 0 0 0 0 0 0 0 0 0 0 0 0 0 0 2 0 0 0 0 0 0 0 0 0 0 0 0 0 0 0 0 0 0 0 0 0 0 0 0 0 0 0 0 0 0 0 0 0 0 0 0 0 0 0 0 0 0 0 0 0 0 0 0 0 0 0 0 0 0 0 0

"137592_fr1" 0 0 3 2 0 4 0 1 0 0 0 3 0 0 0 0 0 0 0 0 2 2 0 0 0 5 0 0 0 0 0 0 0 0 3 0 0 0 0 0 0 0 0 0 0 0 0 0 0 0 0 0 0 0 0 0 0 0 0 0 0 0 0 0 0 0 0 0 0 0 0 0 0 0 0 0 0 0 0 0 0 0 0 0 0

"137660_fr4" 0 1 0 0 3 0 0 0 0 0 1 1 1 2 1 0 2 0 2 2 2 0 2 0 0 1 0 1 0 5 1 2 1 2 0 0 0 0 0 0 0 0 0 0 0 0 0 0 0 0 0 0 0 0 0 0 0 0 0 0 0 0 0 0 0 0 0 0 0 0 0 0 0 0 0 0 0 0 0 0 0 0 0 0 0

"13775_fr4" 0 0 0 0 0 0 0 0 0 0 0 0 0 0 0 0 0 0 0 0 0 0 0 1 0 0 0 0 0 0 0 0 0 0 0 0 0 0 0 0 0 0 0 0 0 0 0 0 0 0 0 0 0 0 0 0 0 0 0 0 0 0 0 0 0 0 0 0 0 0 0 0 0 0 0 0 0 0 0 0 0 0 0 0 0

"13781_fr3" 0 0 0 0 0 0 0 0 2 0 0 1 3 4 5 1 5 0 4 4 2 0 2 2 0 2 2 0 1 4 2 1 4 2 0 0 0 0 0 0 0 0 0 0 0 0 0 0 0 0 0 0 0 0 0 0 0 0 0 0 0 0 0 0 0 0 0 0 0 0 0 0 0 0 1 0 1 2 1 1 0 0 0 1 0

"137871_fr4" 0 0 0 0 0 0 0 0 0 0 0 0 0 0 0 0 0 0 0 0 0 0 0 3 0 0 0 0 0 0 0 0 0 0 0 0 0 0 0 0 0 0 0 0 0 0 0 0 0 0 0 0 0 0 0 0 0 0 0 0 0 0 0 0 0 0 0 0 0 0 0 0 0 0 0 0 0 0 0 0 0 0 0 0 0

"138096_fr2" 0 0 0 0 0 0 0 0 0 0 1 0 0 0 0 0 0 0 0 0 0 0 4 0 0 0 0 0 0 0 0 0 2 0 0 0 0 0 0 0 0 0 0 0 0 0 0 0 0 0 0 0 0 0 0 0 0 0 0 0 0 0 0 0 0 0 0 0 0 0 0 0 0 0 0 0 0 0 0 0 0 0 0 0 0

"13825_fr3" 0 0 0 0 0 0 0 0 0 0 0 0 0 0 0 0 0 0 0 0 0 0 0 0 0 0 0 0 0 0 0 0 0 0 0 0 0 0 0 2 0 0 0 0 4 0 1 0 0 4 1 0 0 4 0 0 0 0 1 3 0 0 0 0 0 0 0 0 0 0 0 0 0 0 0 0 0 0 0 0 0 0 0 0 0

"13849_fr2" 0 0 0 0 0 1 0 0 0 0 0 0 0 0 0 2 0 0 0 0 0 0 0 0 0 0 0 0 0 2 0 1 3 0 0 0 0 0 0 0 0 0 0 0 0 0 0 0 0 0 0 0 0 0 0 0 0 0 0 0 0 0 0 0 0 0 0 0 0 0 0 0 0 0 0 0 0 0 0 0 0 0 0 0 0

"138719_fr2" 0 0 0 0 0 0 0 0 0 0 0 0 0 0 0 0 0 0 0 0 0 0 0 0 0 0 0 0 0 0 0 0 0 0 3 0 0 0 0 0 0 0 0 0 0 0 0 0 0 0 0 0 0 0 0 0 0 0 0 0 0 0 0 0 0 0 0 0 0 0 0 0 0 0 0 0 0 0 0 0 0 0 0 0 0

"138924_fr2" 0 0 0 3 0 0 0 1 0 0 0 0 0 0 0 0 0 0 0 0 0 0 0 0 0 0 0 0 0 0 0 0 0 0 0 0 0 0 0 0 0 0 0 0 0 0 0 0 0 0 0 0 0 0 0 0 0 0 0 0 0 0 0 0 0 0 0 0 0 0 0 0 0 0 0 0 0 0 0 0 0 0 0 0 0

"139091_fr5" 14 8 7 0 7 7 2 0 0 0 5 0 6 0 0 8 6 0 6 2 1 5 1 14 0 11 7 4 2 6 1 6 0 9 0 0 0 0 0 0 0 0 0 0 0 0 0 0 0 0 0 0 0 0 0 0 0 0 0 0 0 0 0 0 0 0 0 0 0 0 0 0 0 0 0 0 0 0 0 0 0 0 0 0 0

"13974_fr6" 0 0 0 0 0 0 0 0 0 0 0 0 0 2 0 0 0 0 0 1 0 0 0 0 0 1 0 0 0 3 0 0 0 0 0 0 0 0 0 0 0 0 0 0 0 0 0 0 0 0 0 0 0 0 0 0 0 0 0 0 0 0 0 0 0 0 0 0 0 0 1 0 1 2 0 2 3 3 4 3 1 0 0 2 0

"14_fr3" 13 11 14 18 16 12 18 16 19 30 11 18 16 9 18 19 17 39 5 14 5 16 2 6 23 7 3 8 29 17 40 2 11 7 20 4 0 8 0 5 0 0 8 0 0 0 0 0 0 0 0 0 0 0 0 0 2 0 0 0 0 0 0 0 0 0 0 0 0 0 0 0 1 0 0 8 9 14 12 6 16 18 28 18 21

"140868_fr2" 4 0 0 0 0 0 0 0 0 0 0 0 2 0 0 0 0 3 0 0 0 0 0 0 0 0 0 0 0 0 0 0 0 0 0 0 0 0 0 0 0 0 0 0 0 0 0 0 0 0 0 0 0 0 0 0 0 0 0 0 0 0 0 0 0 0 0 0 0 0 0 1 0 0 0 1 0 0 0 0 0 0 2 0 0

"140931_fr3" 0 0 0 0 0 0 0 0 0 0 0 0 0 0 0 0 0 0 0 0 1 0 0 0 0 0 2 0 0 0 0 0 0 0 0 0 0 0 0 0 0 0 0 0 0 0 0 0 0 0 0 0 0 0 0 0 0 0 0 0 0 0 0 0 0 0 0 0 0 0 0 0 0 0 0 0 0 0 0 0 0 0 0 0 0

"141086_fr5" 2 2 0 4 0 4 0 8 2 2 0 2 0 2 2 3 2 0 2 0 0 2 0 0 0 0 0 1 0 0 0 0 0 4 2 0 0 0 0 0 0 0 0 0 0 0 0 0 0 0 0 0 0 0 0 0 0 0 0 0 0 0 0 0 0 0 0 0 0 0 6 4 2 5 6 7 12 4 13 8 5 3 4 8 9

"14118_fr1" 0 0 0 0 0 0 0 0 0 0 0 1 0 1 0 0 0 0 0 3 0 0 4 1 0 0 0 0 0 2 0 3 0 0 0 0 0 0 0 0 0 0 0 0 0 0 0 0 0 0 0 0 0 0 0 0 0 0 0 0 0 0 0 0 0 0 0 0 0 0 0 0 0 0 0 0 3 1 0 0 0 0 0 0 0

"141191_fr5" 0 0 0 0 0 0 0 1 0 0 3 0 0 2 0 3 0 4 0 0 1 0 0 0 0 2 0 1 0 0 3 0 2 1 0 0 0 0 0 0 0 0 0 0 0 0 0 0 0 0 0 0 0 0 0 0 0 0 0 0 0 0 0 0 0 0 0 0 0 0 0 0 0 0 0 0 0 0 0 0 0 0 0 0 0

"141220_fr4" 0 0 0 0 0 0 0 0 0 0 0 0 0 0 0 0 0 0 0 0 0 0 0 0 3 0 0 0 0 0 0 0 0 0 2 0 0 0 0 0 0 0 0 0 0 0 0 0 0 0 0 0 0 0 0 0 0 0 0 0 0 0 0 0 0 0 0 0 0 0 0 0 0 0 0 0 0 0 0 0 0 0 0 0 0

"141228_fr2" 0 0 0 0 0 0 0 0 0 0 0 0 0 0 0 0 0 0 0 0 0 0 0 1 0 0 0 0 0 0 0 0 0 0 0 0 0 0 0 0 0 0 0 0 0 0 0 0 0 0 0 0 0 0 0 0 0 0 0 0 0 0 0 0 0 0 0 0 0 0 0 0 0 0 0 0 0 0 0 0 0 0 0 0 0

"141294_fr5" 0 0 0 0 0 0 0 0 0 0 0 0 0 0 0 0 1 0 0 0 0 0 0 0 0 0 0 0 0 0 0 0 0 0 0 0 0 0 0 0 0 0 0 0 0 0 0 0 0 0 0 0 0 0 0 0 0 0 0 0 0 0 0 0 0 0 0 0 0 0 0 0 0 0 0 0 0 0 0 0 0 0 0 0 0

"141565_fr2" 0 0 0 0 0 0 0 0 0 0 0 0 0 0 0 0 0 0 0 0 0 0 0 0 0 0 0 0 0 0 0 0 0 0 0 0 0 0 0 0 0 0 0 0 0 0 0 0 0 0 0 0 0 0 0 0 0 0 0 0 0 0 0 0 0 0 0 0 0 0 0 0 0 0 0 0 0 2 3 0 0 0 0 0 0

"141610_fr1" 0 0 0 0 0 0 0 0 0 0 0 0 0 0 0 0 0 0 0 0 0 0 0 0 0 0 0 0 0 3 0 0 0 0 0 0 0 0 0 0 0 0 0 0 0 0 0 0 0 0 0 0 0 0 0 0 0 0 0 0 0 0 0 0 0 0 0 0 0 0 0 0 0 0 0 0 0 0 0 0 0 0 0 0 0

"141642_fr4" 0 0 0 7 0 0 0 4 7 0 0 0 0 4 6 6 2 6 0 2 0 5 0 2 2 0 0 0 1 1 3 0 2 0 6 0 0 0 0 0 0 0 0 0 0 0 0 0 0 0 0 0 0 0 0 0 0 0 0 0 0 0 0 0 0 0 0 0 0 0 0 0 0 0 0 0 0 0 0 0 0 0 0 0 0

"141775_fr4" 8 13 15 14 16 15 11 16 9 14 11 10 12 8 12 11 12 13 13 15 13 2 14 13 13 13 15 10 13 2 11 11 15 12 10 0 0 0 0 0 2 0 0 4 0 0 0 0 0 0 0 0 0 0 0 0 0 0 0 0 0 0 0 0 0 0 0 0 0 0 0 0 0 0 0 0 1 0 0 0 2 0 0 0 0

"14232_fr3" 2 0 3 3 0 0 4 3 3 4 4 0 2 0 3 2 1 5 1 3 1 2 0 2 2 1 1 0 3 0 2 2 0 2 3 0 0 0 0 0 0 0 0 0 0 0 0 0 0 0 0 0 0 0 0 0 0 0 0 0 0 0 0 0 0 0 0 0 0 0 0 0 0 0 0 0 0 0 0 0 1 0 5 2 2

"14235_fr6" 0 0 0 0 0 0 0 1 0 0 0 0 0 0 0 0 0 0 0 2 0 2 0 0 0 0 4 2 2 0 3 0 0 2 0 0 0 0 0 0 0 0 0 0 0 0 0 0 0 0 0 0 0 0 0 0 0 0 0 0 0 0 0 0 0 0 0 0 0 0 0 0 0 0 0 0 0 0 0 0 0 0 0 0 0

"1425_fr6" 0 0 0 0 0 0 0 0 0 0 0 0 0 0 0 0 0 0 0 0 1 0 0 0 0 0 0 0 0 0 0 0 0 0 0 0 0 0 0 0 0 0 0 0 0 0 0 0 0 0 0 0 0 0 0 0 0 0 0 0 0 0 0 0 0 0 0 0 0 0 0 0 0 0 0 0 0 0 0 0 0 0 0 0 0

"142711_fr4" 0 0 0 0 4 0 0 0 0 0 0 0 0 0 0 0 0 0 0 0 0 0 0 2 0 0 3 0 0 4 0 0 0 3 0 0 0 0 0 0 0 0 0 0 0 0 0 0 0 0 0 0 0 0 0 0 0 0 0 0 0 0 0 0 0 0 0 0 0 0 0 0 0 0 0 0 0 0 0 0 0 0 0 0 0

"143_fr6" 2 3 0 0 2 0 2 0 0 0 0 2 0 0 0 2 0 0 1 0 1 3 0 2 0 2 0 0 0 0 0 0 0 3 0 0 0 0 0 0 0 0 0 0 0 0 0 0 0 0 0 0 0 0 0 0 0 0 0 0 0 0 0 0 0 0 0 0 0 0 0 0 0 0 0 0 0 0 0 0 0 0 0 0 0

"143364_fr4" 0 0 0 0 0 0 0 0 0 6 0 0 0 0 7 0 6 0 0 0 0 7 0 6 3 3 0 0 0 0 0 0 0 0 0 0 0 4 0 0 0 0 0 0 0 0 0 0 0 0 0 0 0 0 0 0 0 0 0 0 0 0 0 0 0 0 0 2 0 0 0 0 0 2 0 0 0 0 0 0 0 0 0 6 0

"14348_fr5" 0 0 0 0 0 0 0 0 0 0 0 0 0 0 0 0 0 0 0 1 0 0 0 0 0 0 0 0 0 0 0 0 0 0 0 0 0 0 0 0 0 0 0 0 0 0 0 0 0 0 0 0 0 0 0 0 0 0 0 0 0 0 0 0 0 0 0 0 0 0 0 0 0 0 0 0 0 0 0 0 0 0 0 0 0

"143811_fr2" 5 0 0 2 0 0 0 4 2 0 0 5 0 0 0 0 0 0 0 0 6 2 0 0 0 0 0 0 0 4 0 0 0 0 0 0 0 0 0 0 0 0 0 0 0 0 0 0 0 0 0 0 0 0 0 0 0 0 0 0 0 0 0 0 0 0 0 0 0 0 0 0 0 0 0 0 0 0 0 0 0 0 0 0 0

"14388_fr6" 2 5 2 2 2 0 0 5 0 2 1 0 2 0 3 1 1 0 4 2 4 4 2 7 7 5 0 2 2 2 0 2 2 6 1 0 0 0 0 0 0 0 0 0 0 0 0 0 0 0 0 0 0 0 0 0 0 0 0 0 0 0 0 0 0 0 0 0 0 0 0 0 0 0 0 0 0 0 0 0 0 0 0 0 0

"14404_fr1" 20 36 33 32 37 30 37 36 32 33 34 23 32 29 36 28 31 34 26 30 33 29 37 38 34 33 32 39 33 27 36 44 36 30 37 9 9 12 1 17 2 3 8 6 4 3 3 3 5 0 3 1 2 0 5 2 4 3 1 2 0 0 0 0 0 4 3 3 1 0 2 1 4 5 3 13 25 23 20 16 15 18 26 13 25

"144144_fr2" 0 0 0 0 0 0 0 0 0 0 0 0 2 0 0 0 0 0 0 0 0 0 0 0 0 0 0 0 0 0 0 0 0 0 0 0 0 0 0 0 0 0 0 0 0 0 0 0 0 0 0 0 0 0 0 0 0 0 0 0 0 0 0 0 0 0 0 0 0 0 0 0 0 0 0 0 0 0 0 0 0 0 0 0 0

"144152_fr3" 1 3 2 1 3 1 2 4 2 0 2 3 4 2 2 2 1 2 2 3 2 4 3 0 2 0 4 4 2 2 2 3 4 2 1 0 0 0 0 0 0 0 0 0 0 0 0 0 0 0 0 0 0 0 0 0 0 0 0 0 0 0 0 0 0 0 0 0 0 0 0 0 0 0 0 0 0 0 0 0 0 0 0 0 0

"144157_fr4" 0 0 0 0 0 0 0 0 0 2 0 0 0 0 0 0 0 0 0 0 0 0 0 0 0 0 0 0 0 0 0 0 0 0 0 0 0 0 0 0 0 0 0 0 0 0 0 0 0 0 0 0 0 0 0 0 0 0 0 0 0 0 0 0 0 0 0 0 0 0 0 0 0 0 0 0 0 0 0 0 0 0 0 0 0

"144171_fr6" 13 2 14 20 11 10 17 14 23 14 12 13 19 12 17 13 13 4 10 10 9 16 9 15 14 7 8 10 11 12 20 14 13 13 21 0 0 1 0 7 0 0 0 0 0 0 0 0 0 0 0 0 0 0 0 0 0 0 0 0 0 0 0 0 0 0 0 0 0 0 0 0 0 0 0 0 0 2 1 0 8 2 9 0 0

"144184_fr5" 0 0 0 0 0 0 0 0 0 0 0 0 0 0 0 0 0 0 0 0 0 0 0 0 0 0 0 0 0 0 0 0 0 0 0 0 0 0 1 0 0 0 0 0 0 0 0 0 1 2 2 3 0 0 0 1 0 0 0 0 0 0 0 0 0 0 0 0 0 0 0 0 0 0 0 0 0 0 0 0 0 0 0 0 0

"144320_fr1" 0 0 2 2 0 2 1 2 2 0 2 1 2 0 2 0 0 2 0 2 2 0 2 0 2 2 2 2 2 0 2 2 2 2 2 0 0 0 0 0 0 0 0 0 0 0 0 0 0 0 0 0 0 0 0 0 0 0 0 0 0 0 0 0 0 0 0 0 0 0 0 0 0 0 0 0 0 0 0 0 0 0 0 0 0

"144332_fr2" 0 0 0 0 0 0 0 0 0 0 0 0 1 0 0 0 0 0 0 0 0 0 0 0 0 0 0 0 0 0 0 0 0 0 0 0 0 0 0 0 0 0 0 0 0 0 0 0 0 0 0 0 0 0 0 0 0 0 0 0 0 0 0 0 0 0 0 0 0 0 0 0 0 0 0 0 0 0 0 0 0 0 0 0 0

"144378_fr3" 0 0 0 0 0 0 0 0 0 0 0 0 0 0 0 0 0 0 0 0 0 0 0 0 0 0 0 0 0 0 0 0 0 0 0 0 0 0 0 0 0 0 0 0 0 0 0 0 0 0 0 0 0 0 0 0 0 0 0 0 0 0 0 0 0 0 0 0 0 0 0 0 0 0 0 0 0 2 0 0 1 0 10 2 10

"144790_fr4" 0 0 0 0 0 0 0 0 0 4 0 0 0 0 0 0 0 0 0 0 0 0 0 0 0 0 0 0 0 0 0 0 0 0 0 0 0 0 0 0 0 0 0 0 0 0 0 0 0 0 0 0 0 0 0 0 0 0 0 0 0 0 0 0 0 0 0 0 0 0 0 0 0 0 0 0 0 0 0 0 0 0 0 0 0

"145070_fr4" 0 0 2 0 0 2 2 0 0 1 0 1 0 0 4 2 2 0 2 4 3 9 0 0 4 2 0 0 0 0 2 2 5 2 2 0 0 0 0 6 0 0 2 0 0 0 0 0 0 0 0 0 0 0 0 0 0 0 0 0 0 0 0 0 0 0 0 0 0 0 0 0 0 0 0 0 5 2 0 2 0 3 0 3 0

"145138_fr3" 2 0 2 2 0 2 0 1 0 0 1 0 0 0 0 0 0 0 0 0 0 2 2 0 0 0 3 2 0 0 0 0 0 1 0 0 0 0 0 0 0 0 0 0 0 0 0 0 0 0 0 0 0 0 0 0 0 0 0 0 0 0 0 0 0 0 0 0 0 0 0 0 0 0 0 0 0 0 0 0 0 0 0 0 0

"14545_fr1" 0 0 4 0 0 3 0 0 0 0 2 0 0 0 0 0 4 0 3 0 0 0 2 10 0 4 5 0 0 0 0 6 0 4 0 0 0 0 0 0 0 0 0 0 0 0 0 0 0 0 0 0 0 0 0 0 0 0 0 0 0 0 0 0 0 0 0 0 0 0 0 0 0 0 0 0 0 0 0 0 0 0 0 0 0

"14580_fr6" 2 2 5 5 4 8 0 0 2 1 3 2 2 6 3 1 1 0 0 0 0 0 0 0 0 4 5 3 9 1 4 0 0 0 4 1 0 2 0 3 0 0 3 3 3 3 1 2 0 0 0 0 3 4 2 0 0 2 2 3 0 0 0 1 0 3 2 2 2 2 2 2 2 1 2 1 3 3 2 2 0 0 1 0 1

"147007_fr3" 0 0 0 0 0 0 0 0 0 0 0 3 0 0 0 2 1 0 0 0 0 0 0 0 0 4 0 0 2 0 0 0 4 0 0 0 0 0 0 0 0 0 0 0 0 0 0 0 0 0 0 0 0 0 0 0 0 0 0 0 0 0 0 0 0 0 0 0 0 0 0 0 0 0 0 0 0 0 0 0 0 0 0 0 0

"14720_fr4" 0 0 0 0 0 0 0 0 0 0 0 0 0 0 0 0 0 0 0 0 0 0 0 0 0 0 0 0 0 0 0 0 0 0 0 0 0 0 0 0 0 0 0 0 0 0 0 0 0 0 0 0 0 0 0 0 0 0 0 0 0 4 0 0 2 0 0 0 0 0 0 0 0 0 0 0 0 0 0 0 0 0 0 0 0

"14720_fr5" 0 0 0 0 0 0 0 0 0 0 0 0 0 0 0 0 0 0 0 0 0 0 0 0 0 0 0 0 0 0 0 0 0 0 0 0 0 0 2 0 0 0 0 0 2 0 3 1 0 3 0 0 0 0 0 0 0 0 0 0 0 0 0 0 0 0 0 0 0 0 0 0 0 0 0 0 0 0 0 0 0 0 0 0 0

"147298_fr2" 0 0 0 0 0 0 0 0 0 0 0 0 0 0 0 0 0 0 0 0 0 0 0 0 0 0 0 0 0 0 0 0 0 0 0 0 0 0 4 0 0 0 0 2 0 1 0 0 1 0 0 0 0 0 4 0 0 0 0 0 0 0 0 0 0 0 0 0 0 0 0 0 0 0 0 0 0 0 0 0 0 0 0 0 0

"147363_fr5" 15 2 2 7 4 1 5 2 6 7 2 0 2 5 2 16 9 12 2 1 4 5 0 2 7 0 3 1 6 1 2 4 6 2 8 0 0 1 0 0 0 0 1 0 0 0 0 0 0 0 0 0 0 0 0 0 0 0 0 0 0 0 0 0 0 0 0 0 0 0 0 0 0 0 0 2 0 4 4 1 2 2 2 2 6

"147423_fr4" 0 0 0 0 0 0 0 0 0 0 0 0 0 0 0 0 0 0 0 0 0 1 0 0 0 0 0 2 0 0 0 0 0 0 0 0 0 0 0 0 0 0 0 0 0 0 0 0 0 0 0 0 0 0 0 0 0 0 0 0 0 0 0 0 0 0 0 0 0 0 0 0 0 0 0 0 0 0 0 0 0 0 0 0 0

"147876_fr3" 0 0 0 0 0 0 0 0 0 0 0 0 0 0 0 0 0 0 0 0 0 0 0 0 0 3 0 0 0 0 0 0 0 0 0 0 0 0 0 0 0 0 0 0 0 0 0 0 0 0 0 0 0 0 0 0 0 0 0 0 0 0 0 0 0 0 0 0 0 0 0 0 0 0 0 0 0 0 0 0 0 0 0 0 0

"147887_fr5" 0 0 0 0 0 0 0 0 0 0 0 0 0 0 0 0 0 0 0 0 0 0 0 0 0 0 0 0 0 0 0 0 0 0 0 0 0 0 0 0 0 0 0 0 0 0 0 0 0 0 0 0 0 0 0 0 0 4 0 0 0 0 0 0 0 0 0 0 0 0 0 0 0 0 0 0 0 0 0 0 0 0 0 0 0

"148355_fr4" 0 0 4 2 0 0 0 0 0 0 0 0 0 0 0 0 0 5 0 0 0 0 0 0 0 0 0 0 0 0 0 0 11 0 5 0 0 0 0 0 0 0 0 0 0 0 0 0 0 0 0 0 0 0 0 0 0 0 0 0 0 0 0 0 0 0 0 0 0 0 0 0 0 0 0 0 0 0 0 0 0 0 0 0 0

"148587_fr2" 1 0 2 1 0 2 0 0 0 0 0 0 0 0 0 0 0 0 0 0 0 0 0 0 0 0 0 0 0 0 0 0 0 0 0 0 0 0 0 0 0 0 0 0 0 0 0 0 0 0 0 0 0 0 0 0 0 0 0 0 0 0 0 0 0 0 0 0 0 0 0 0 0 0 0 0 0 0 0 0 0 0 0 0 0

"14923_fr5" 0 0 0 0 0 2 0 0 0 0 0 0 0 0 0 0 0 0 0 0 0 0 0 0 0 0 2 0 0 0 0 0 0 0 0 0 0 0 0 0 0 0 0 0 0 0 0 0 0 0 0 0 0 0 0 0 0 0 0 0 0 0 0 0 0 0 0 0 0 0 0 0 0 0 0 0 0 0 0 0 0 0 0 0 0

"150178_fr1" 0 0 0 0 0 0 0 0 0 0 0 0 3 0 5 0 0 0 0 0 1 0 0 0 0 3 1 0 0 0 2 0 0 0 0 0 0 0 0 0 0 0 0 0 0 0 0 0 0 0 0 0 0 0 0 0 0 0 0 0 0 0 0 0 0 0 0 0 0 0 0 0 0 0 0 0 0 0 0 0 0 0 0 0 0

"150370_fr2" 0 0 0 0 0 0 0 0 0 0 0 0 0 0 0 0 0 0 0 0 0 0 0 0 0 0 0 0 0 0 0 0 0 0 0 0 0 0 0 0 0 0 0 0 0 0 0 0 0 0 0 0 0 0 0 0 0 0 0 0 0 0 0 0 1 4 0 0 0 0 0 0 0 0 0 0 0 0 0 0 2 2 0 0 0

"151_fr6" 0 0 0 0 0 0 0 0 0 2 0 0 0 0 0 0 0 0 0 0 2 0 0 0 0 0 0 0 0 0 0 0 0 0 0 0 0 1 1 0 3 1 0 0 3 1 1 0 1 2 4 1 5 1 0 2 3 1 1 1 44 0 0 1 0 1 0 0 0 0 24 2 0 1 1 0 1 19 19 0 9 14 0 6 14

"151500_fr2" 4 0 0 0 0 0 0 0 0 0 0 0 0 0 0 0 0 0 0 0 0 0 0 4 0 0 0 0 0 0 0 0 0 0 0 0 0 0 0 0 0 0 0 0 0 0 0 0 0 0 0 0 0 0 0 0 0 0 0 0 0 0 0 0 0 0 0 0 0 0 0 0 0 0 0 0 0 0 0 0 0 0 0 0 0

"151942_fr2" 1 0 0 0 0 0 0 4 1 0 0 0 0 0 1 0 0 0 0 4 2 0 0 0 2 0 0 0 5 4 0 0 5 0 0 0 0 0 0 0 0 0 0 0 0 0 0 0 0 0 0 0 0 0 0 0 0 0 0 0 0 0 0 0 0 0 0 0 0 0 0 0 0 0 0 0 0 0 0 0 0 0 0 0 0

"15282_fr2" 0 0 0 0 3 0 0 0 1 0 0 0 0 0 0 0 0 0 0 0 0 0 0 0 0 0 0 0 0 2 0 0 0 0 2 0 0 0 0 0 0 0 0 0 0 0 0 0 0 0 0 0 0 0 0 0 0 0 0 0 0 0 0 0 0 0 0 0 0 0 0 0 0 0 0 0 0 0 0 0 0 0 0 0 0

"153034_fr3" 0 2 3 0 0 0 2 0 0 0 0 0 4 0 0 0 0 0 0 0 3 0 0 0 0 0 0 0 1 0 0 0 0 0 1 0 2 1 0 0 3 0 0 0 0 0 0 0 0 0 0 0 0 0 0 0 0 0 0 0 0 0 0 0 0 0 0 0 0 0 0 0 0 0 0 0 0 0 0 0 0 0 0 0 0

"153040_fr3" 0 0 0 0 0 0 0 0 0 0 0 0 0 0 0 0 0 0 0 0 0 0 0 0 0 0 0 0 0 0 0 0 0 0 0 0 0 0 0 0 0 0 0 0 0 0 0 0 0 0 0 0 0 0 0 0 0 0 0 0 0 0 0 0 0 0 0 0 0 0 1 1 1 2 3 2 4 2 2 3 1 1 1 1 1

"153076_fr1" 0 0 0 0 0 0 0 0 0 0 0 0 0 0 0 0 0 0 0 0 0 0 0 0 0 0 0 0 0 0 0 0 0 0 0 0 0 0 1 4 0 0 0 0 0 0 0 0 0 0 0 2 0 0 0 0 0 2 0 0 0 0 0 0 0 0 0 0 0 0 0 0 0 0 0 0 0 0 0 0 0 0 0 0 0

"153091_fr5" 0 0 0 0 0 0 0 0 0 0 0 0 0 0 0 0 0 0 0 0 0 0 0 0 0 0 0 0 0 0 0 0 0 0 0 0 0 0 0 0 0 0 0 0 0 0 0 0 0 0 0 0 0 0 0 0 0 0 0 0 0 0 0 0 0 0 0 0 0 0 0 0 0 0 0 1 1 0 0 0 0 0 1 0 0

"153121_fr6" 0 9 1 0 0 0 5 6 0 9 12 0 6 7 6 12 0 9 0 6 10 4 9 7 3 9 6 4 0 0 9 9 4 0 0 0 0 0 0 0 0 0 0 0 0 0 0 0 0 0 0 0 0 0 0 0 0 0 0 0 0 0 0 0 0 0 0 0 0 0 0 0 0 0 0 0 0 0 0 0 0 0 0 0 0

"153160_fr6" 0 3 2 0 0 0 0 0 2 0 0 1 0 6 0 2 2 0 4 3 3 1 0 0 0 3 0 0 0 1 3 1 0 1 0 0 0 0 0 0 0 0 0 0 0 0 0 0 0 0 0 0 0 0 0 0 0 0 0 0 0 0 0 0 0 2 3 4 5 0 5 5 5 4 6 4 7 5 7 4 4 5 7 3 4

"153193_fr2" 0 0 0 1 3 0 4 0 0 0 0 0 0 14 0 0 0 0 5 1 0 0 5 0 0 0 0 0 4 1 0 10 0 1 0 0 0 0 0 4 0 0 0 0 0 0 0 0 0 0 0 0 0 0 0 0 0 0 0 0 0 0 0 0 0 0 0 0 2 0 1 0 0 0 0 0 13 0 0 1 0 0 0 0 0

"153496_fr1" 0 0 0 0 0 0 0 0 0 3 0 0 0 0 0 0 0 0 0 0 0 0 0 0 0 0 0 0 0 0 0 0 0 0 0 0 0 0 0 0 0 0 0 0 0 0 0 0 0 0 0 0 0 0 0 0 0 0 0 0 0 0 0 0 0 0 0 0 0 0 0 0 0 0 0 0 0 0 0 0 0 0 0 0 0

"153630_fr6" 0 0 4 4 4 0 0 6 0 0 0 0 3 0 0 0 0 0 3 4 3 0 3 5 0 3 7 4 4 5 0 0 4 7 4 0 0 0 0 0 0 0 0 0 0 0 0 0 0 0 0 0 0 0 0 0 0 0 0 0 0 0 0 0 0 0 0 0 0 0 0 0 0 0 0 0 0 0 0 0 0 0 0 0 0

"1538_fr1" 0 0 0 0 0 0 0 0 0 3 0 0 0 0 0 0 0 0 0 0 0 0 0 0 0 0 0 0 0 0 0 0 0 0 0 0 0 0 0 0 0 0 0 0 0 0 0 0 0 0 0 0 0 0 0 0 0 0 0 0 0 0 0 0 0 0 0 0 0 0 0 0 0 0 0 0 0 0 0 0 0 0 0 0 0

"154258_fr2" 2 0 0 0 0 0 0 0 0 0 10 0 0 0 0 2 0 0 0 0 0 0 0 0 0 0 0 1 0 0 8 14 2 0 0 0 0 0 0 0 0 0 0 0 0 0 0 0 0 0 0 0 0 0 0 0 0 0 0 0 0 0 0 0 0 0 0 0 0 0 0 0 0 0 0 0 0 0 0 0 0 0 0 0 0

"15435_fr3" 0 0 0 0 0 0 0 0 2 2 0 0 0 0 0 0 0 1 0 0 0 0 0 0 2 0 0 0 0 0 0 0 0 0 0 0 0 0 0 0 0 0 0 0 0 0 0 0 0 0 0 0 0 0 0 0 0 0 0 0 0 0 0 0 0 0 0 0 0 0 0 0 0 0 0 0 0 0 0 0 0 0 0 0 0

"155_fr4" 0 0 0 0 0 0 0 0 0 0 0 0 0 0 0 0 0 0 0 0 0 0 0 0 0 0 1 0 0 0 0 0 0 0 0 0 0 0 0 0 0 0 0 0 0 0 0 0 0 0 0 0 0 0 0 0 0 0 0 0 0 0 0 0 0 0 0 0 0 0 0 0 0 0 0 0 0 0 0 0 0 0 0 0 0

"15501_fr2" 0 2 1 0 3 2 0 1 0 0 2 0 0 3 5 2 2 0 2 9 3 3 4 3 0 3 2 2 1 2 1 4 6 5 2 0 0 0 0 0 0 0 0 0 0 0 0 0 0 0 0 0 0 0 0 0 0 0 0 0 0 0 0 0 0 0 0 0 0 0 0 0 0 0 0 0 0 0 0 0 0 0 0 0 0

"155245_fr1" 0 0 0 0 2 0 0 0 0 0 0 0 0 0 0 0 0 0 0 0 0 0 0 0 0 0 0 0 0 4 0 0 0 0 0 0 0 0 0 0 0 0 0 0 0 0 0 0 0 0 0 0 0 0 0 0 0 0 0 0 0 0 0 0 0 0 0 0 0 0 0 0 0 0 0 0 0 0 0 0 0 0 0 0 0

"155311_fr4" 0 8 0 0 0 0 6 5 4 7 2 0 6 12 2 0 0 0 1 1 9 0 13 2 0 0 6 10 4 10 0 0 0 10 0 0 0 0 0 0 0 0 0 0 0 0 0 0 0 0 0 0 0 0 0 0 0 0 0 0 0 0 0 0 0 0 0 0 0 0 0 0 0 0 0 0 0 0 0 0 0 0 0 0 0

"15561_fr1" 0 2 0 0 2 2 0 0 0 0 0 0 0 4 0 0 5 0 2 0 0 0 5 2 0 1 0 1 4 5 2 0 4 6 0 0 0 0 0 0 0 0 0 0 0 0 0 0 0 0 0 0 0 0 0 0 0 0 0 0 0 0 0 0 0 0 0 0 0 0 0 0 0 0 0 0 0 0 0 0 0 0 0 0 0

"155615_fr4" 0 0 0 0 0 0 0 0 0 0 0 0 0 0 0 0 0 0 0 0 0 0 0 0 0 0 0 0 0 0 0 0 0 0 0 0 0 0 0 0 0 0 0 0 0 0 0 0 0 0 0 0 0 0 0 0 0 0 0 0 0 0 0 0 0 0 0 0 0 5 0 0 0 0 0 0 0 0 0 0 0 0 0 0 0

"155620_fr2" 4 0 1 7 0 0 4 0 5 0 0 4 4 0 7 4 4 7 0 4 0 0 2 4 0 0 0 1 2 1 6 9 1 1 7 0 0 0 0 0 0 0 0 0 0 0 0 0 0 0 0 0 0 0 0 0 0 0 0 0 0 0 0 0 0 0 0 0 0 0 0 0 0 0 0 0 0 0 0 0 4 4 4 4 4

"155642_fr5" 0 0 0 3 0 0 0 0 4 2 2 2 2 0 3 2 3 4 0 2 0 2 0 2 3 0 0 0 2 2 3 0 0 0 4 0 0 0 0 0 0 0 0 0 0 0 0 0 0 0 0 0 0 0 0 0 0 0 0 0 0 0 0 0 0 0 0 0 0 0 0 0 0 0 0 0 3 2 4 1 5 1 6 3 6

"155689_fr5" 0 0 0 0 0 0 0 0 0 0 0 0 0 0 0 0 0 0 0 0 0 0 0 0 0 0 0 0 0 0 0 0 0 0 0 0 0 0 0 0 0 0 0 0 0 0 0 0 0 0 0 0 0 0 0 0 0 0 0 0 0 0 0 0 0 0 0 0 0 0 0 0 0 0 0 0 4 4 3 0 0 2 0 0 0

"155762_fr2" 2 1 2 2 2 0 0 2 0 0 2 0 2 3 0 0 0 0 0 3 0 0 2 2 0 0 0 2 0 2 0 2 0 2 2 0 0 0 0 0 0 0 0 0 0 0 0 0 0 0 0 0 0 0 0 0 0 0 0 0 0 0 0 0 0 0 0 0 0 0 0 0 0 0 0 0 0 0 0 0 0 0 0 0 0

"155798_fr4" 0 0 0 0 0 0 0 0 0 0 1 0 0 0 0 0 0 0 2 0 0 0 0 0 0 0 0 0 0 0 0 0 0 0 0 0 0 0 0 0 0 0 0 0 0 0 0 0 0 0 0 0 0 0 0 0 0 0 0 0 0 0 0 0 0 0 0 0 0 0 0 0 0 0 0 0 0 0 0 0 0 0 0 0 0

"155803_fr2" 0 0 0 0 0 0 0 0 0 0 0 0 0 0 0 0 0 0 0 0 0 0 0 0 0 0 0 0 0 0 0 0 0 0 0 0 0 0 0 0 0 0 0 0 0 0 0 0 0 0 0 0 0 0 0 0 0 0 0 0 0 0 0 0 0 0 0 3 0 0 0 0 3 0 0 1 1 0 1 0 0 0 0 0 0

"155837_fr3" 0 0 1 0 2 0 0 0 0 0 7 2 0 0 0 0 0 0 0 1 1 0 9 2 0 2 0 1 2 0 2 11 1 1 0 0 0 0 0 0 0 0 0 0 0 0 0 0 0 0 0 0 0 0 0 0 0 0 0 0 0 0 0 0 0 0 0 0 0 0 0 0 0 0 0 0 0 0 0 0 0 0 0 0 0

"155889_fr2" 0 0 0 0 0 0 0 0 0 0 0 0 0 0 0 0 0 0 0 0 0 0 0 0 0 0 0 0 0 0 0 0 0 0 0 4 5 0 0 6 0 0 0 3 3 0 0 0 3 0 0 0 0 0 0 3 0 0 0 0 0 0 0 0 0 0 0 0 0 0 0 0 0 0 0 0 0 0 0 0 0 0 0 0 0

"155991_fr2" 10 15 19 14 19 7 8 14 12 3 9 5 0 13 8 10 8 3 21 20 17 16 27 15 13 11 21 4 22 0 14 27 20 18 7 0 0 0 0 0 0 0 0 0 0 0 0 0 0 1 0 3 0 0 4 1 0 0 0 0 0 0 0 0 0 0 0 1 0 0 0 1 0 0 6 0 0 0 2 3 0 1 0 0 0

"156_fr4" 0 0 2 1 0 1 0 0 0 0 0 0 0 0 0 0 0 0 0 0 0 0 0 2 0 0 2 0 1 1 0 0 0 0 0 0 0 0 0 0 0 0 0 0 0 0 0 0 0 0 0 0 0 0 0 0 0 0 0 0 0 0 0 0 0 0 0 0 0 0 0 0 0 0 0 0 0 0 0 0 0 0 0 0 0

"156236_fr1" 0 0 0 0 0 0 0 0 0 0 0 0 0 0 0 0 0 0 0 0 0 0 0 0 0 0 0 0 0 0 0 0 0 3 0 0 0 0 0 0 0 0 0 0 0 0 0 0 0 0 0 0 0 0 0 0 0 0 0 0 0 0 0 0 0 0 0 0 0 0 0 0 0 0 0 0 0 0 0 0 0 0 0 0 0

"156252_fr6" 0 0 0 0 0 4 0 0 0 0 3 0 0 0 0 0 0 0 0 0 0 0 0 0 0 0 0 0 0 0 0 0 0 0 0 0 0 0 0 0 0 0 0 0 0 0 0 0 0 0 0 0 0 0 0 0 0 0 0 0 0 0 0 0 0 0 0 0 0 0 0 0 0 0 0 0 0 0 0 0 0 0 0 0 0

"1574_fr4" 0 0 0 0 0 0 0 0 0 0 0 0 0 0 0 0 0 0 0 0 0 0 0 0 0 0 2 0 1 0 0 0 0 0 0 0 0 0 0 0 0 0 0 0 0 0 0 0 0 0 0 0 0 0 0 0 0 0 0 0 0 0 0 0 0 0 0 0 0 0 0 0 0 0 0 0 0 0 0 0 0 0 0 0 0

"1574_fr5" 0 0 0 0 0 0 0 0 0 0 0 0 0 0 0 0 0 0 0 0 0 0 0 0 0 0 0 0 0 0 0 0 0 0 0 0 0 0 0 0 0 0 0 0 0 0 0 0 0 0 0 0 0 0 0 0 0 0 0 0 0 0 0 0 0 0 0 0 0 0 0 0 0 0 0 0 0 0 0 0 0 0 4 2 0

"15750_fr4" 0 0 0 0 0 0 0 0 0 0 0 0 0 0 0 0 0 0 0 0 0 0 0 0 0 0 0 0 0 0 0 0 0 0 0 0 0 0 0 0 0 0 0 0 0 0 0 0 0 0 0 0 0 0 0 0 0 0 0 0 0 0 0 0 0 0 0 0 0 0 0 0 0 0 0 0 0 0 0 0 0 2 0 0 0

"15759_fr3" 0 0 0 0 0 0 0 0 0 0 0 0 0 0 0 0 0 0 0 0 0 0 0 0 0 0 0 0 0 0 0 0 0 0 0 0 0 0 0 0 0 0 0 2 0 0 0 0 0 0 0 0 0 1 0 1 0 0 0 0 0 0 0 0 0 0 0 0 0 0 0 0 0 0 0 0 0 0 0 0 0 0 0 0 0

"157736_fr6" 0 0 0 0 3 0 0 3 0 0 0 0 0 1 0 0 0 0 0 0 0 5 0 3 0 5 4 0 3 1 0 4 0 0 0 0 0 0 0 0 0 0 0 0 0 0 0 0 0 0 0 0 0 0 0 0 0 0 0 0 0 0 0 0 0 0 0 3 0 0 0 0 0 0 0 0 0 0 0 0 0 0 0 0 0

"157947_fr6" 0 0 0 0 0 1 2 0 2 0 0 0 0 0 0 2 0 0 0 0 3 0 0 0 0 0 0 0 0 0 0 0 0 0 0 0 0 0 0 0 0 0 0 0 0 0 0 0 0 0 0 0 0 0 0 0 0 0 0 0 0 0 0 0 0 0 1 0 0 2 0 0 0 0 0 0 1 0 0 0 0 0 0 0 0

"1580_fr3" 0 0 0 0 0 0 0 0 0 0 0 0 0 0 0 0 0 0 0 0 0 0 0 0 0 0 0 0 0 0 0 0 0 0 0 3 2 2 0 2 0 0 0 0 0 0 0 0 0 0 0 0 0 0 0 0 0 0 0 0 0 0 0 0 0 0 0 0 0 0 0 0 0 0 0 0 0 0 0 0 0 0 0 0 0

"158015_fr5" 0 0 0 0 1 0 2 0 0 1 1 3 0 0 0 0 0 1 0 0 1 0 0 0 0 0 2 0 0 0 0 0 0 0 0 0 0 0 0 0 0 0 0 0 0 0 0 0 0 0 0 0 0 0 0 0 0 0 0 0 0 0 0 0 0 0 0 0 0 0 0 0 0 0 0 0 0 0 0 0 0 0 0 0 0

"158027_fr2" 2 0 0 0 3 0 1 0 0 0 1 0 0 0 2 0 0 0 0 1 0 2 1 0 0 0 5 2 1 2 0 0 2 2 0 0 0 0 0 0 0 0 0 0 0 0 0 0 0 0 0 0 0 0 0 0 0 0 0 0 0 0 0 0 0 0 0 0 0 0 0 0 0 0 0 0 0 0 0 0 0 0 0 0 0

"158043_fr6" 0 0 0 1 0 0 0 0 0 0 0 0 0 1 0 0 0 0 0 1 0 0 0 0 0 0 0 0 0 0 0 0 0 0 0 0 0 0 0 0 0 0 0 0 0 0 0 0 0 0 0 0 0 0 3 0 0 0 0 0 0 0 0 0 0 0 0 0 0 0 0 0 0 0 0 0 0 0 0 0 0 0 0 0 0

"158083_fr3" 0 0 0 1 0 0 0 0 3 0 0 0 0 0 0 0 0 1 0 0 0 0 0 0 2 0 0 0 0 0 0 0 0 0 1 0 0 0 0 0 0 0 0 0 0 0 0 0 0 0 0 0 0 0 0 0 0 0 0 0 0 0 0 0 0 0 0 0 0 0 0 0 0 0 0 0 0 0 0 0 0 0 0 0 0

"158419_fr1" 0 0 0 0 0 0 0 0 0 0 0 0 0 0 0 0 0 0 0 0 0 0 0 0 0 0 0 0 0 0 0 0 0 0 0 0 0 2 4 3 4 0 3 0 4 4 4 4 0 4 4 4 3 5 4 4 4 4 2 4 0 1 2 3 4 4 0 2 1 4 4 2 0 0 0 0 3 0 0 0 0 0 0 0 0

"158593_fr3" 0 0 0 0 0 0 0 0 0 0 0 0 0 0 0 0 0 0 0 0 0 0 0 0 0 0 0 0 0 0 0 0 0 0 0 0 0 0 0 0 0 0 0 2 0 0 1 0 0 0 0 0 0 0 0 0 0 0 0 0 0 0 0 0 0 0 0 0 0 0 0 0 0 0 0 0 0 0 0 0 0 0 0 0 0

"1601_fr2" 0 5 4 3 1 4 0 5 2 1 4 1 3 1 4 4 3 3 3 0 4 2 5 3 3 2 5 0 5 0 2 3 4 5 3 0 0 0 0 0 0 0 0 0 0 0 0 0 0 0 0 0 0 0 0 0 0 0 0 0 0 0 0 0 0 0 0 0 0 0 0 0 0 0 0 0 0 0 0 0 0 0 0 0 0

"160359_fr6" 0 0 0 0 0 1 0 0 0 0 0 3 0 0 0 0 0 0 0 0 0 0 0 0 0 0 0 0 0 0 0 0 0 0 0 0 0 0 0 0 0 0 0 0 0 0 0 0 0 0 0 0 0 0 0 0 0 0 0 0 0 0 0 0 0 0 0 0 0 0 0 0 0 0 0 0 0 0 0 0 0 0 0 0 0

"160363_fr1" 0 0 0 0 0 0 0 0 0 0 0 0 0 0 0 0 0 0 0 0 0 0 0 0 0 0 0 0 0 0 0 0 0 0 0 3 0 3 0 0 0 0 0 0 0 0 0 0 0 0 0 0 0 0 0 0 0 0 0 0 0 0 0 0 0 0 0 0 0 0 0 0 0 0 0 0 0 0 0 3 0 0 0 0 0

"160369_fr4" 0 0 0 0 0 0 0 0 0 0 0 0 0 0 0 0 0 0 0 0 0 0 0 0 0 0 0 3 0 0 0 0 0 0 0 0 0 0 0 0 0 0 0 0 0 0 0 0 0 0 0 0 0 0 0 0 0 0 0 0 0 0 0 0 0 0 0 0 0 0 0 0 0 0 0 0 0 0 0 0 0 0 0 0 0

"160381_fr4" 2 0 2 0 0 0 0 2 0 0 2 0 2 0 0 2 2 0 7 0 1 2 0 0 2 7 6 5 0 0 0 7 0 0 0 0 0 0 0 0 0 0 0 0 0 0 0 0 0 0 0 0 0 0 0 0 0 0 0 0 0 0 0 0 0 0 0 0 0 0 0 0 0 0 0 0 0 0 0 0 0 0 0 0 0

"160483_fr5" 0 0 0 0 0 0 0 0 0 0 0 0 0 0 0 0 0 0 0 0 0 0 0 0 0 0 0 0 0 1 0 0 0 0 0 0 0 0 0 0 0 0 0 0 0 0 0 0 0 0 0 0 0 0 0 0 0 0 0 0 0 0 0 0 0 0 0 0 0 0 0 0 0 0 0 0 0 0 0 0 0 0 0 0 0

"160484_fr6" 0 0 0 2 0 0 0 3 0 0 0 0 0 0 0 0 0 0 0 0 0 0 2 0 0 0 2 0 0 0 0 0 0 0 0 0 0 0 0 0 0 0 0 0 0 0 0 0 0 0 0 0 0 0 0 0 0 0 0 0 0 0 0 0 0 0 0 0 0 0 0 0 0 0 0 0 0 0 0 0 0 0 0 0 0

"160555_fr4" 0 1 0 0 0 0 0 0 0 0 0 0 1 0 0 0 0 0 0 0 0 0 1 2 0 0 5 1 0 2 1 2 0 0 0 0 0 0 0 0 0 0 0 0 0 0 0 0 0 0 0 0 0 0 0 0 0 0 0 0 0 0 0 0 0 0 0 0 0 0 0 0 0 0 0 0 0 0 0 0 0 0 0 0 0

"160935_fr3" 0 0 0 0 0 0 0 0 0 0 0 0 0 0 0 0 0 0 0 0 0 0 0 0 0 0 0 0 0 0 0 3 0 0 0 0 0 0 0 0 0 0 0 0 0 0 0 0 0 0 0 0 0 0 0 0 0 0 0 0 0 0 0 0 0 0 0 0 0 0 0 0 0 0 0 0 0 0 0 0 0 0 0 0 0

"16110_fr4" 0 0 0 0 0 0 0 0 0 0 0 0 0 0 0 0 0 0 0 0 0 0 0 0 0 0 0 0 0 0 0 0 0 0 0 0 3 0 2 0 9 2 12 2 3 3 2 2 2 2 4 2 4 4 3 4 5 2 4 2 3 21 2 2 3 3 0 1 2 0 0 0 0 0 0 0 0 0 0 0 0 0 0 0 0

"16143_fr4" 0 0 0 0 2 0 0 0 0 0 0 0 0 0 0 0 0 0 0 0 0 1 0 1 2 0 2 0 1 1 0 2 0 0 0 0 0 0 0 0 0 0 0 0 0 0 0 0 0 0 0 0 0 0 0 0 0 0 0 0 0 0 0 0 0 0 0 0 0 0 0 0 0 0 0 0 0 0 0 0 0 0 0 0 0

"16167_fr2" 7 4 4 7 4 4 3 4 4 0 4 4 2 4 3 4 4 2 4 2 4 4 4 7 1 4 8 8 4 2 7 8 4 4 4 1 0 0 0 2 0 0 0 0 0 0 0 0 0 0 0 0 0 0 0 0 0 0 0 0 0 0 0 0 0 0 0 0 0 0 0 0 0 0 0 0 2 0 0 0 0 0 2 5 0

"16199_fr5" 0 0 0 0 3 0 2 0 1 0 0 3 0 2 0 0 0 0 0 0 0 0 0 0 0 0 0 0 0 4 0 0 0 0 0 0 0 0 0 0 0 0 0 0 0 0 0 0 0 0 0 0 0 0 0 0 0 0 0 0 0 0 0 0 0 0 0 0 0 0 0 0 0 0 0 0 0 0 0 0 0 0 0 0 0

"162106_fr1" 0 0 0 0 0 0 0 0 0 0 0 0 0 0 0 0 0 0 0 0 1 0 0 0 0 0 1 0 0 0 0 6 0 0 0 0 0 0 0 0 0 0 0 0 0 0 0 0 0 0 0 0 0 0 0 0 0 0 0 0 0 0 0 0 0 0 0 0 0 0 0 0 0 0 0 0 0 0 0 0 0 0 0 0 0

"162506_fr3" 0 0 0 0 0 0 0 0 0 0 0 0 0 0 0 0 2 0 0 0 0 0 3 0 0 0 0 0 0 0 0 0 0 0 0 0 0 0 0 0 0 0 0 0 0 0 0 0 0 0 0 0 0 0 0 0 0 0 0 0 0 0 0 0 0 0 0 0 0 0 0 0 0 0 0 0 0 0 0 0 0 0 0 0 0

"162551_fr3" 0 0 0 0 0 0 0 0 0 0 0 0 0 0 0 0 0 0 0 3 0 0 0 0 0 0 0 2 0 0 0 0 0 0 0 0 0 0 0 0 0 0 0 0 0 0 0 0 0 0 0 0 0 0 0 0 0 0 0 0 0 0 0 0 0 0 0 0 0 0 0 0 0 0 0 0 0 0 0 0 0 0 0 0 0

"162564_fr1" 0 0 0 0 0 1 0 0 0 0 0 0 0 0 0 0 0 0 0 0 0 0 1 0 0 3 0 0 0 0 0 0 0 0 0 0 0 0 0 0 0 0 0 0 0 0 0 0 0 0 0 0 0 0 0 0 0 0 0 0 0 0 0 0 0 0 0 0 0 0 0 0 0 0 0 0 0 0 0 0 0 0 0 0 0

"162578_fr5" 1 0 0 0 5 0 0 0 0 0 1 0 0 0 0 0 1 0 2 0 0 0 0 0 0 2 0 2 0 7 0 0 0 0 0 0 0 2 0 9 0 0 0 0 0 0 0 0 0 0 0 0 0 0 0 0 0 0 0 0 0 0 0 0 0 8 0 0 0 6 0 0 0 0 0 0 1 5 4 0 3 0 0 0 0

"162582_fr2" 3 9 3 1 12 2 9 2 9 7 11 2 7 3 9 3 4 8 12 3 10 2 14 2 0 4 12 10 11 13 2 14 11 4 0 0 0 0 0 0 0 0 0 0 5 0 0 0 0 0 0 0 0 0 0 0 0 0 0 0 0 0 0 0 0 2 0 6 0 1 17 15 18 18 20 7 9 15 11 21 11 0 0 0 8

"162616_fr2" 0 0 0 0 0 0 0 0 0 0 0 0 0 0 0 0 0 0 0 0 0 0 0 0 0 0 0 0 0 0 0 0 0 0 0 0 0 0 0 0 0 0 0 0 0 0 0 0 0 0 0 0 0 0 0 0 0 0 0 0 0 0 0 0 0 0 0 0 0 0 0 0 0 3 0 2 4 4 2 2 0 0 0 0 0

"1628_fr1" 0 4 6 5 5 4 1 7 2 0 0 3 1 0 2 1 1 0 0 3 2 2 5 2 0 0 5 1 2 2 1 5 3 2 0 0 0 0 0 0 0 0 0 0 0 0 0 0 0 0 0 0 0 0 0 0 0 0 0 0 0 0 0 0 0 0 0 0 0 0 0 0 0 0 0 0 0 0 0 0 0 0 0 0 0

"163642_fr3" 0 0 0 0 0 0 0 2 0 0 0 0 0 0 4 0 0 0 3 0 4 0 6 0 0 0 0 0 3 0 0 0 0 0 0 0 0 0 0 0 0 0 0 0 0 0 0 0 0 0 0 0 0 0 0 0 0 0 0 0 0 0 0 0 0 0 0 0 0 0 0 1 3 4 0 0 0 0 0 0 0 3 4 0 3

"164353_fr6" 0 0 0 0 0 0 0 0 0 0 0 0 0 0 0 0 0 0 0 0 0 0 0 0 0 0 0 0 0 0 0 0 0 0 0 0 0 0 0 0 0 0 0 0 0 0 0 0 0 0 0 0 0 0 0 0 0 0 0 0 0 0 0 0 0 0 0 0 0 0 0 0 0 0 0 0 0 0 0 2 0 0 0 0 0

"164360_fr5" 7 0 0 0 0 0 0 0 0 0 0 0 0 0 0 0 0 0 0 0 0 0 0 0 0 0 0 0 0 0 0 0 0 0 0 3 0 0 0 0 0 1 0 0 0 0 0 0 0 0 0 0 0 0 0 0 0 0 0 0 0 0 0 0 0 0 0 0 0 0 0 0 0 0 0 0 0 0 0 0 0 0 0 0 0

"164562_fr1" 0 0 0 0 0 0 0 0 0 0 0 0 0 0 0 0 0 0 0 0 0 0 0 0 0 0 0 0 0 0 0 0 0 0 0 0 0 0 0 0 0 0 0 0 0 0 0 0 0 0 0 0 0 0 0 0 0 0 0 0 0 0 0 0 0 0 0 0 2 6 0 0 0 0 0 0 0 0 0 0 0 0 0 0 0

"164713_fr5" 0 1 0 0 2 0 0 0 0 0 0 1 0 0 3 0 0 0 2 0 0 0 0 0 0 0 0 0 2 0 0 0 2 0 0 0 0 0 0 0 0 0 0 0 0 0 0 0 0 0 0 0 0 0 0 0 0 0 0 0 0 0 0 0 0 0 0 0 0 0 0 0 0 0 0 0 0 0 0 0 0 0 0 0 0

"16488_fr1" 0 0 0 0 0 0 0 0 0 0 0 0 0 0 0 0 0 0 0 0 0 0 0 0 0 0 0 0 0 0 0 0 0 0 0 0 0 0 0 0 0 0 0 0 0 0 0 0 0 0 0 0 0 0 0 0 0 0 0 0 0 0 0 0 0 0 2 2 1 0 0 2 1 2 2 2 2 2 1 2 2 2 1 1 0

"166043_fr4" 3 0 0 0 2 0 0 0 0 0 0 0 0 2 0 0 0 0 2 0 1 0 0 0 0 0 0 0 0 2 0 2 0 0 0 0 0 0 0 0 0 0 0 0 0 0 0 0 0 0 0 0 0 0 0 0 0 0 0 0 0 0 0 0 0 0 0 0 0 0 0 0 0 0 0 0 0 0 0 0 0 0 0 0 0

"166183_fr3" 0 0 0 0 0 0 0 0 0 0 0 0 0 3 0 0 0 0 0 0 0 0 0 0 0 0 0 0 0 0 0 0 0 0 0 0 0 0 0 0 0 0 0 0 0 0 0 0 0 0 0 0 0 0 0 0 0 0 0 0 0 0 0 0 0 0 0 0 0 0 0 0 0 0 0 0 0 0 0 0 0 0 0 0 0

"166184_fr2" 0 0 0 0 0 0 0 0 0 0 0 0 0 0 0 2 2 0 0 2 2 3 0 0 0 0 4 0 0 0 0 4 3 0 0 0 0 0 0 0 0 0 0 0 0 0 0 0 0 0 0 0 0 0 0 0 0 0 0 0 0 0 0 0 0 0 0 0 0 0 0 0 0 0 0 0 0 0 0 0 0 0 0 0 0

"166198_fr4" 0 0 0 0 0 0 0 0 0 4 0 0 0 0 0 0 0 0 0 0 0 0 0 0 0 0 0 0 0 0 0 0 0 0 0 0 0 0 0 0 0 0 0 0 0 0 0 0 0 0 0 0 0 0 0 0 0 0 0 0 0 0 0 0 0 0 0 0 0 0 0 0 0 0 0 0 0 0 0 0 0 0 0 0 0

"166361_fr5" 0 0 0 0 0 0 0 0 0 0 0 0 0 0 0 0 0 0 0 0 3 0 0 0 0 0 0 0 0 0 0 0 0 0 0 0 0 0 0 0 0 0 0 0 0 0 0 0 0 0 0 0 0 0 0 0 0 0 0 0 0 0 0 0 0 0 0 0 0 0 0 0 0 0 0 0 0 0 0 0 0 0 0 0 0

"166374_fr5" 0 1 0 0 0 0 0 0 0 0 0 0 0 0 4 0 1 0 0 0 0 0 0 0 0 0 0 0 0 0 2 2 1 0 0 0 0 0 0 0 0 0 0 0 0 0 0 0 0 0 0 0 0 0 0 0 0 0 0 0 0 0 0 0 0 0 0 0 0 0 0 0 0 0 0 0 0 0 0 0 0 0 0 0 0

"166437_fr1" 0 0 0 0 0 0 0 0 0 0 0 0 0 0 0 3 0 0 0 0 4 0 0 0 0 0 0 0 0 0 0 4 0 0 0 0 0 0 0 0 0 0 0 0 0 0 0 0 0 0 0 0 0 0 0 0 0 0 0 0 0 0 0 0 0 0 0 0 0 0 0 0 0 0 0 0 0 0 0 0 0 0 0 0 0

"166827_fr1" 0 0 5 5 9 7 9 6 6 0 8 9 13 7 0 5 0 10 0 7 11 0 8 0 0 0 7 11 5 7 0 8 7 9 0 2 2 0 0 6 0 3 3 0 3 0 3 0 4 3 3 2 0 0 0 0 0 0 1 1 0 2 0 1 0 2 0 0 0 0 0 0 0 0 0 5 0 6 8 0 8 0 8 0 7

"16696_fr1" 0 0 0 2 2 2 0 2 0 0 2 2 0 2 2 0 2 2 2 2 2 0 4 2 1 0 2 2 2 1 4 2 2 2 0 0 0 0 0 0 0 0 0 0 0 0 0 0 0 0 0 0 0 0 0 0 0 0 0 0 0 0 0 0 0 0 0 0 0 0 0 0 0 0 0 0 0 0 0 0 0 0 0 0 0

"167195_fr1" 0 0 0 0 0 0 0 0 0 0 0 0 0 0 0 0 0 0 0 0 0 0 0 0 0 0 0 0 0 0 0 0 0 0 0 0 0 0 0 0 0 0 0 0 0 0 0 0 0 0 0 0 0 0 0 0 0 0 0 0 0 0 0 0 0 0 0 3 2 0 0 0 0 3 0 3 3 3 3 2 2 0 0 0 0

"16739_fr5" 0 0 0 0 0 0 0 0 0 0 0 0 0 0 0 0 0 0 0 0 0 0 0 0 0 0 0 0 3 0 0 0 0 0 0 0 0 0 0 0 0 0 0 0 0 0 0 0 0 0 0 0 0 0 0 0 0 0 0 0 0 0 0 0 0 0 0 0 0 0 0 0 0 0 0 0 0 0 0 0 0 0 0 0 0

"167919_fr6" 0 1 0 0 0 3 0 0 0 0 0 0 0 0 0 0 0 0 0 0 2 0 0 0 0 0 1 0 0 2 0 0 0 2 0 0 0 0 0 0 0 0 0 0 0 0 0 0 0 0 0 0 0 0 0 0 0 0 0 0 0 0 0 0 0 0 0 0 0 0 0 0 0 0 0 0 0 0 0 0 0 0 0 0 0

"167946_fr1" 0 0 0 0 0 0 0 0 0 0 0 0 0 0 0 0 0 0 0 0 0 0 0 0 0 0 0 0 0 0 0 0 0 0 0 0 0 0 0 0 0 0 2 0 0 0 0 0 0 0 0 0 0 0 0 0 0 0 0 0 0 0 0 0 0 0 0 0 0 0 0 0 0 0 0 0 0 0 0 0 0 0 0 0 0

"167958_fr3" 0 0 0 0 2 0 0 0 0 0 0 0 0 1 0 0 0 0 0 0 0 0 0 0 0 0 0 0 0 0 0 0 0 0 0 0 0 0 0 0 0 0 0 0 0 0 0 0 0 0 0 0 0 0 0 0 0 0 0 0 0 0 0 0 0 0 0 0 0 0 0 0 0 0 0 0 0 0 0 0 0 0 0 0 0

"167959_fr3" 0 0 0 0 0 0 0 0 0 0 0 0 0 0 0 0 0 0 0 0 0 0 0 0 0 0 0 0 0 0 0 0 0 0 0 0 0 0 0 0 0 0 0 0 0 0 0 0 0 0 0 0 0 0 0 0 0 0 0 0 0 0 0 0 0 0 0 0 0 0 0 0 4 4 0 0 0 0 0 0 0 0 4 0 0

"168076_fr2" 0 0 0 0 0 0 0 0 0 0 0 0 0 0 0 0 0 0 0 0 0 0 0 0 0 4 0 0 0 0 0 0 0 0 0 0 0 0 0 0 0 0 0 0 0 0 0 0 0 0 0 0 0 0 0 0 0 0 0 0 0 0 0 0 0 0 0 0 0 0 0 0 0 0 0 0 0 0 0 0 0 0 0 0 0

"168131_fr3" 0 0 0 2 0 0 0 0 2 0 0 0 0 1 0 0 0 0 0 4 0 0 1 0 8 4 0 0 1 0 2 1 0 0 0 0 0 0 0 0 0 0 0 0 0 0 0 0 0 0 0 0 0 0 0 0 0 0 0 0 0 0 0 0 0 0 0 0 0 0 0 0 0 0 0 0 1 3 4 0 0 1 0 1 2

"168248_fr3" 0 7 5 0 2 0 2 1 2 2 0 2 2 0 0 7 0 1 1 2 1 2 1 0 1 0 0 1 2 1 4 2 4 0 0 1 2 0 0 3 0 0 0 1 0 0 0 0 0 0 0 0 0 0 0 0 0 0 0 0 0 0 0 0 0 0 0 0 0 0 0 0 0 0 0 0 0 0 0 0 0 0 0 0 0

"168270_fr4" 0 0 0 0 0 2 0 0 0 0 0 0 0 0 0 0 0 0 0 0 0 0 0 0 0 0 6 0 0 0 0 2 0 0 0 0 0 0 0 0 0 0 0 0 0 0 0 0 0 0 0 0 0 0 0 0 0 0 0 0 0 0 0 0 0 0 0 0 0 0 0 0 0 0 0 0 0 0 0 0 0 0 0 0 0

"168505_fr1" 0 0 0 0 0 0 0 0 0 0 0 0 0 2 0 0 0 0 0 0 0 0 0 0 0 0 0 0 0 3 0 0 0 0 0 0 0 0 0 0 0 0 0 0 0 0 0 0 0 0 0 0 0 0 0 0 0 0 0 0 0 0 0 0 0 0 0 0 0 0 0 0 0 0 0 0 0 0 0 0 0 0 0 0 0

"16919_fr2" 0 0 2 2 0 2 0 1 0 0 0 0 0 0 0 1 2 0 0 1 0 0 1 2 0 0 0 0 0 1 0 0 1 0 0 0 0 0 0 0 0 0 0 0 0 0 0 0 0 0 0 0 0 0 0 0 0 0 0 0 0 0 0 0 0 0 0 0 0 0 0 0 0 0 0 0 0 0 0 0 0 0 0 0 0

"169584_fr4" 0 0 0 0 0 0 0 0 0 0 0 0 0 0 0 0 0 0 0 0 0 0 0 0 0 0 0 0 0 0 0 0 0 0 0 2 2 2 0 2 0 0 2 8 2 5 1 0 3 2 0 0 1 2 2 0 0 1 1 2 0 0 0 0 0 0 0 0 0 0 0 0 0 0 0 0 0 0 0 0 0 0 0 0 0

"169586_fr1" 0 0 0 0 0 0 0 0 0 0 0 0 0 0 0 0 0 0 0 0 0 0 0 0 0 0 0 0 0 0 0 0 0 0 0 1 1 1 7 2 6 0 2 0 10 1 3 0 0 12 3 11 1 11 12 8 7 2 3 1 2 3 6 2 1 1 0 2 0 6 0 0 4 4 6 0 0 0 0 6 0 0 0 0 1

"169603_fr2" 0 0 0 0 0 0 0 0 0 0 1 0 0 0 0 0 0 0 0 0 0 0 0 0 0 0 0 0 0 0 1 0 0 0 0 0 0 1 0 2 0 0 0 0 0 0 0 0 0 0 0 0 0 0 0 0 0 0 0 2 0 0 0 0 0 0 0 0 0 0 0 0 0 0 0 0 0 0 0 0 0 0 0 0 0

"169622_fr1" 0 0 0 0 0 0 0 0 0 0 2 0 1 0 0 0 2 0 0 0 0 2 0 0 0 0 0 0 0 0 0 0 0 0 0 0 0 0 0 0 0 0 0 0 0 0 0 0 0 0 0 0 0 0 0 0 0 0 0 0 0 0 0 0 0 0 0 0 0 0 0 0 0 0 0 0 0 0 0 0 0 0 0 0 0

"169648_fr6" 0 0 0 0 0 0 0 0 0 0 0 0 0 0 0 0 0 0 0 0 0 0 0 0 0 0 0 0 1 0 0 0 0 0 0 0 0 0 0 0 0 0 0 0 0 0 0 0 0 0 0 0 0 0 0 0 0 0 0 0 0 0 0 0 0 0 0 0 0 0 0 0 0 0 0 0 0 0 0 0 0 0 0 0 0

"169669_fr4" 0 0 0 0 0 0 0 0 0 0 0 0 0 0 0 0 0 0 0 0 0 0 0 0 0 0 2 0 0 0 0 6 0 0 0 0 0 0 0 0 0 0 0 0 0 0 0 0 0 0 0 0 0 0 0 0 0 0 0 0 0 0 0 0 0 0 0 0 0 0 0 0 0 0 0 0 0 0 0 0 0 0 0 0 0

"169703_fr3" 0 0 2 0 0 0 0 0 0 1 0 0 6 0 0 0 0 4 0 0 0 0 0 0 2 0 0 0 0 0 0 0 0 0 0 0 0 0 0 0 0 0 0 0 0 0 0 0 0 0 0 0 0 0 0 0 0 0 0 0 0 0 0 0 0 0 0 0 0 0 0 0 0 0 0 0 0 0 0 0 0 0 0 0 0

"16972_fr2" 10 9 15 12 14 14 6 4 8 8 13 15 2 9 12 0 12 5 13 12 15 10 18 13 0 12 17 15 16 13 10 14 16 15 11 1 4 2 0 2 0 0 0 2 0 0 0 0 0 0 0 0 0 0 0 0 0 0 0 0 0 0 0 0 0 0 0 0 0 0 0 0 7 1 2 2 4 12 4 0 1 9 10 0 3

"169742_fr1" 0 0 0 0 0 0 0 0 0 4 0 0 0 0 0 0 0 2 0 0 0 0 0 0 3 0 0 0 0 0 0 0 0 0 0 0 0 0 0 0 0 0 0 0 0 0 0 0 0 0 0 0 0 0 0 0 0 0 0 0 0 0 0 0 0 0 0 0 0 0 0 0 0 2 1 0 0 1 0 0 0 0 0 0 0

"169778_fr4" 0 0 0 0 0 0 0 0 0 0 0 0 0 0 0 0 0 0 1 0 0 1 0 0 0 0 0 2 0 1 0 0 0 0 0 0 0 0 0 0 0 0 0 0 0 0 0 0 0 0 0 0 0 0 0 0 0 0 0 0 0 0 0 0 0 0 0 0 0 0 0 0 0 0 0 0 0 0 0 0 0 0 0 0 0

"169890_fr3" 3 0 0 0 2 0 0 2 0 0 0 0 0 0 0 0 0 0 0 0 0 0 0 0 0 0 0 0 2 0 0 0 0 0 0 0 0 0 0 0 0 0 0 0 0 0 0 0 0 0 0 0 0 0 0 0 0 0 0 0 0 0 0 0 0 0 0 0 0 0 0 0 0 0 0 0 0 0 0 0 0 0 0 0 0

"17029_fr2" 0 1 2 0 2 0 3 2 0 0 0 0 1 4 0 3 4 3 3 3 4 5 0 3 0 5 4 0 3 0 3 4 0 0 0 0 0 0 0 0 0 0 0 0 0 0 0 0 0 0 0 0 0 0 0 0 0 0 0 0 0 0 0 0 0 0 0 0 0 0 0 0 0 0 0 0 2 2 2 0 0 0 0 0 0

"170352_fr4" 0 0 0 0 1 0 0 0 0 0 0 0 0 0 0 0 0 0 0 0 0 0 0 0 0 0 0 0 0 0 0 0 0 0 0 0 0 0 0 0 0 0 0 0 0 0 0 0 0 0 0 0 0 0 0 0 0 0 0 0 0 0 0 0 0 0 0 0 0 0 0 0 0 0 0 0 0 0 0 0 0 0 0 0 0

"17046_fr6" 0 0 0 0 0 0 0 0 0 0 0 0 0 0 0 0 0 0 0 0 0 0 0 0 0 0 0 0 0 0 0 0 0 0 0 19 29 60 125 54 43 79 98 115 139 125 123 129 115 169 152 149 139 192 151 143 146 121 167 193 112 111 117 116 139 114 106 108 122 104 98 79 71 74 83 57 54 60 53 47 25 37 7 29 43

"170702_fr2" 2 0 0 4 2 0 2 9 4 10 2 0 4 0 1 0 1 8 0 0 0 0 0 2 5 0 0 2 0 0 0 0 0 1 4 0 0 0 0 0 0 0 0 0 0 0 0 0 0 0 0 0 0 0 0 0 0 0 0 0 0 0 0 0 0 0 0 0 0 0 0 0 0 0 0 0 0 0 0 0 0 0 0 0 0

"17091_fr1" 0 0 0 0 0 0 0 0 0 0 0 0 0 0 0 0 2 0 3 0 0 0 4 0 0 0 0 0 0 5 0 0 0 0 0 0 0 0 0 0 0 0 0 0 0 0 0 0 0 0 0 0 0 0 0 0 0 0 0 0 0 0 0 0 0 0 0 0 0 1 0 1 0 2 2 0 3 0 0 0 0 0 0 0 0

"171058_fr5" 0 0 0 0 0 0 0 0 0 3 0 0 0 0 3 0 0 0 0 0 0 0 0 0 2 0 0 0 0 1 0 0 0 0 0 0 1 0 0 0 2 0 3 0 0 0 0 0 0 2 1 0 0 0 1 0 0 0 0 2 0 0 0 0 0 0 0 0 0 0 0 0 0 0 0 0 0 0 0 0 0 0 0 0 0

"171207_fr2" 7 8 0 0 9 0 4 0 6 0 0 6 5 0 0 0 0 6 0 0 0 0 0 0 0 0 0 0 0 0 0 0 0 0 0 0 0 0 0 0 0 0 0 0 0 0 0 0 0 0 0 0 0 0 0 0 0 0 0 0 0 0 0 0 0 0 0 0 0 0 0 0 0 0 0 0 0 0 0 0 0 0 0 0 0

"171219_fr5" 2 2 2 2 3 2 2 2 2 4 2 0 2 0 2 2 2 2 2 2 2 2 3 2 0 1 3 2 3 2 2 5 2 2 2 0 0 0 0 0 0 0 0 0 0 0 0 0 0 0 0 0 0 0 0 0 0 0 0 0 1 0 0 0 0 0 0 0 0 0 2 2 6 0 0 4 11 4 4 3 4 4 4 4 11

"17127_fr3" 1 0 2 2 0 2 1 2 2 2 3 2 0 2 4 0 0 0 0 2 1 1 4 0 1 2 2 0 2 0 2 0 2 2 3 0 0 0 0 0 0 0 0 0 0 0 0 0 0 0 0 0 0 0 0 0 0 0 0 0 0 0 0 0 0 0 0 0 0 0 0 0 0 0 0 9 2 3 2 2 2 2 2 2 2

"171303_fr3" 0 0 0 0 0 0 0 0 0 3 0 0 0 0 0 0 0 0 0 0 0 0 0 0 0 0 0 0 0 0 0 0 0 0 0 0 0 0 0 0 0 0 0 0 0 0 0 0 0 0 0 0 0 0 0 0 0 0 0 0 0 0 0 0 0 0 0 0 0 0 0 0 0 0 0 0 0 0 0 0 0 0 0 0 0

"171435_fr4" 0 0 0 5 0 5 0 1 0 0 0 0 0 0 0 0 0 0 0 0 0 0 2 0 0 1 0 0 0 0 0 0 0 0 0 0 0 0 0 0 0 0 0 0 0 0 0 0 0 0 0 0 0 0 0 0 0 0 0 0 0 0 0 0 0 0 0 0 0 0 0 0 0 0 0 0 0 3 0 0 0 0 0 0 0

"171530_fr6" 0 0 0 0 0 0 0 0 0 0 0 6 0 0 0 0 2 0 0 0 0 1 0 0 0 0 0 0 0 6 0 0 0 0 0 0 0 0 0 0 0 0 0 0 0 0 0 0 0 0 0 0 0 0 0 0 0 0 0 0 0 0 0 0 0 0 0 0 0 0 1 0 3 1 0 0 0 0 0 3 2 0 2 1 1

"1719_fr3" 2 0 0 0 0 0 0 0 0 0 0 0 0 0 0 0 0 0 0 0 0 0 0 12 0 0 0 0 0 0 0 3 0 0 0 0 0 0 0 0 0 0 0 0 0 0 0 0 0 0 0 0 0 0 0 0 0 0 0 0 0 0 0 0 0 0 0 0 0 0 0 0 0 0 0 0 0 0 0 0 0 0 0 0 0

"17207_fr1" 0 0 0 0 0 0 0 0 0 0 0 0 0 0 0 0 0 0 0 0 0 0 0 0 0 0 0 0 0 0 0 0 0 0 0 0 0 0 0 0 0 0 0 0 0 0 0 0 0 0 0 0 0 0 0 0 0 0 0 0 0 0 0 0 0 0 0 0 0 0 0 0 0 0 0 3 4 0 1 0 0 0 0 0 1

"17207_fr2" 4 3 4 6 1 0 4 4 5 3 0 3 0 4 2 5 2 5 0 0 0 4 0 0 2 0 1 4 0 0 2 0 0 0 0 0 0 0 0 0 0 0 0 0 0 0 0 0 0 0 0 0 0 0 0 0 0 0 0 0 0 0 0 0 0 0 0 0 0 0 0 0 0 0 0 0 0 0 0 0 0 0 0 0 0

"17244_fr3" 0 0 0 0 0 0 0 0 0 0 0 0 0 0 0 0 0 0 0 0 0 0 0 0 0 0 0 0 0 0 0 0 0 0 0 0 0 0 0 0 0 0 0 0 0 0 0 0 0 0 0 0 0 0 0 0 0 0 0 0 0 0 0 0 0 0 2 4 0 0 0 0 0 0 0 0 0 0 0 0 0 0 0 0 0

"172697_fr5" 0 0 0 0 0 0 0 0 0 0 0 0 0 0 0 0 0 0 0 0 0 0 0 0 0 0 0 0 0 0 0 0 0 0 0 0 0 2 6 0 8 0 5 3 9 7 5 2 5 4 6 8 0 6 8 10 5 7 12 12 0 9 0 0 0 0 8 11 0 7 4 5 6 4 0 6 5 2 2 0 0 2 0 0 0

"172699_fr5" 1 0 0 2 2 2 0 0 1 2 0 1 0 0 0 0 2 2 2 0 0 1 2 2 2 1 0 0 0 2 1 2 2 1 0 0 0 0 0 0 0 0 0 0 0 0 0 0 0 0 0 0 0 0 0 0 0 0 0 0 0 0 0 0 0 0 0 0 0 0 0 0 0 0 0 0 0 0 0 0 0 0 0 0 0

"172832_fr5" 0 0 0 0 0 0 0 0 0 0 2 0 0 0 3 0 0 0 0 4 3 3 5 3 0 0 0 0 2 0 2 6 4 0 0 0 0 0 0 0 0 0 0 0 0 0 0 0 0 0 0 0 0 0 0 0 0 0 0 0 0 0 0 0 0 0 0 0 0 0 0 0 0 0 0 0 0 0 0 0 0 0 0 0 0

"1736_fr5" 0 0 0 0 0 0 0 0 0 2 0 0 1 0 0 0 0 0 0 0 0 0 0 0 0 0 0 0 0 0 0 0 0 0 0 0 0 0 0 0 0 0 0 0 0 0 0 0 0 0 0 0 0 0 0 0 0 0 0 0 0 0 0 0 0 0 0 0 0 0 0 0 0 0 0 0 0 0 0 0 0 0 0 0 0

"17385_fr4" 0 0 0 0 0 0 0 0 0 0 0 0 0 0 0 0 0 0 0 0 0 0 0 0 0 0 0 0 0 0 0 0 0 0 0 0 1 0 0 0 2 3 1 2 4 3 5 2 3 0 2 0 0 6 4 0 6 5 3 0 0 2 0 0 0 0 0 0 0 0 0 0 0 0 0 2 0 0 0 3 2 0 0 0 0

"174113_fr3" 0 2 2 0 0 0 0 0 0 0 0 0 2 0 0 0 0 2 0 0 0 0 0 0 0 0 0 0 2 0 0 0 0 0 0 0 0 0 0 0 0 0 0 0 0 0 0 0 0 0 0 0 0 0 0 0 0 0 0 0 0 0 0 0 0 0 0 0 0 0 0 0 0 0 0 0 0 0 0 0 0 0 0 0 0

"174148_fr5" 0 0 0 0 0 0 0 0 0 4 0 0 0 0 0 0 0 0 0 0 0 0 0 0 0 0 1 0 0 0 0 0 0 0 0 0 0 0 0 0 0 0 0 0 0 0 0 0 0 0 0 0 0 0 0 0 0 0 0 0 0 0 0 0 0 0 0 0 0 0 0 0 0 0 0 0 0 0 0 0 0 0 0 0 0

"174162_fr1" 0 0 0 3 0 0 0 0 2 0 0 0 0 5 0 0 1 0 0 0 0 0 0 0 0 1 0 0 0 0 0 0 2 0 0 0 0 0 0 0 0 0 0 0 0 0 0 0 0 0 0 0 0 0 0 0 0 0 0 0 0 0 0 0 0 0 0 0 0 0 0 0 0 0 0 0 0 0 0 0 0 0 0 0 0

"17423_fr2" 0 0 0 0 0 0 0 0 0 0 0 0 0 0 0 0 0 0 0 0 0 0 0 0 0 0 0 0 0 0 0 0 0 0 0 0 0 0 0 0 0 0 0 0 0 0 0 0 0 0 0 0 0 0 0 0 0 0 0 0 0 0 0 0 0 0 0 1 0 0 6 8 5 7 8 10 6 0 0 7 0 0 0 0 0

"174271_fr4" 0 0 0 0 0 1 0 0 0 0 0 1 0 0 0 0 0 0 0 0 0 0 0 0 0 0 0 0 0 0 0 0 0 0 0 0 0 0 0 0 0 0 0 0 0 0 0 0 0 0 0 0 0 0 0 0 0 0 0 0 0 0 0 0 0 0 0 0 0 0 0 0 0 0 0 0 0 0 0 0 0 0 0 0 0

"174278_fr6" 0 6 10 6 0 8 3 9 4 6 9 0 4 8 0 7 7 3 7 5 6 7 7 8 9 6 9 2 6 6 5 8 7 6 0 0 0 0 0 0 0 0 0 0 0 0 0 0 0 0 0 0 0 0 0 0 0 0 0 0 0 0 0 0 0 0 0 0 0 0 0 0 0 0 0 0 0 0 0 0 0 0 0 0 0

"175002_fr1" 3 0 0 0 0 0 0 0 0 0 0 0 0 0 0 0 0 0 0 1 0 0 4 0 0 4 0 0 0 0 0 0 0 0 0 0 0 0 0 0 0 0 0 0 0 0 0 0 0 0 0 0 0 0 0 0 0 0 0 0 0 0 0 0 0 0 0 0 0 0 0 0 0 0 0 0 0 0 0 0 0 0 0 0 0

"17502_fr5" 0 0 0 0 0 0 0 0 0 0 0 0 0 0 0 0 0 0 0 0 0 0 0 0 0 0 0 0 0 0 0 0 0 0 0 0 0 4 0 0 4 0 0 3 5 0 7 8 0 6 8 6 9 8 0 0 10 8 0 0 7 0 0 0 0 6 8 0 1 7 0 0 0 0 0 0 0 0 0 0 0 0 0 0 0

"175507_fr6" 0 0 0 0 0 0 0 0 0 0 0 0 0 3 0 0 0 0 0 3 0 0 2 0 0 4 0 0 4 0 0 0 2 0 0 1 0 0 0 0 2 0 0 0 0 0 0 0 0 0 0 0 0 0 0 0 0 0 0 0 0 0 0 0 0 0 0 0 0 0 0 0 0 0 0 0 1 3 1 0 1 0 2 0 0

"175700_fr1" 0 0 0 0 5 0 0 0 0 0 0 0 0 0 0 0 0 0 0 0 0 0 4 2 0 0 2 0 3 0 0 6 2 0 0 0 0 0 0 0 0 0 0 0 0 0 0 0 0 0 0 0 0 0 0 0 0 0 0 0 0 0 0 0 0 0 0 3 0 0 0 6 5 4 6 5 0 0 8 0 0 0 0 0 0

"175708_fr4" 0 0 0 0 0 0 0 0 0 0 1 0 0 0 0 0 0 0 0 0 0 0 0 0 0 0 0 2 0 0 0 0 0 0 0 0 0 0 0 0 0 0 0 0 0 0 0 0 0 0 0 0 0 0 0 0 0 0 0 0 0 0 0 0 0 0 0 0 0 0 0 0 0 0 0 0 0 0 0 0 0 0 0 0 0

"17585_fr4" 0 0 0 0 0 0 0 0 0 0 0 0 0 0 0 0 0 0 0 0 0 0 0 0 0 0 0 0 0 0 0 0 0 0 0 0 0 0 0 0 0 0 0 0 0 0 0 0 0 0 0 0 0 0 0 0 0 0 0 0 0 2 0 0 0 0 0 0 0 0 0 0 0 0 0 0 0 0 0 0 0 1 0 0 0

"176301_fr2" 0 0 0 3 0 0 0 0 3 0 0 0 0 0 0 0 0 0 0 2 0 2 0 3 0 0 0 0 0 0 0 0 0 0 2 0 0 0 0 0 0 0 0 0 0 0 0 0 0 0 0 0 0 0 0 0 0 0 0 0 0 0 0 0 0 0 0 0 0 0 0 0 0 0 0 0 0 0 0 0 0 0 0 0 0

"176791_fr1" 0 0 0 7 0 10 0 1 0 0 0 0 0 0 0 0 0 4 0 0 0 0 6 0 0 2 0 0 0 0 0 0 6 0 5 0 0 0 0 0 0 0 0 0 0 0 0 0 0 0 0 0 0 0 0 0 0 0 0 0 0 0 0 0 0 0 0 0 0 0 0 0 0 0 0 0 0 0 0 0 0 0 0 0 0

"176997_fr2" 0 0 0 0 0 0 0 0 0 0 0 0 0 0 0 0 0 0 0 0 0 0 0 0 0 0 0 0 0 0 0 0 0 0 0 0 0 0 0 0 0 0 0 0 0 0 2 0 0 0 0 0 0 0 0 0 0 0 0 0 0 0 0 0 0 0 0 0 0 0 0 0 0 0 0 0 0 0 0 0 0 0 0 0 0

"17708_fr3" 0 0 0 0 0 0 0 0 0 0 0 0 0 0 0 0 0 0 0 0 0 0 0 0 0 0 0 0 0 0 0 0 0 3 0 0 0 0 0 0 0 0 0 0 0 0 0 0 0 0 0 0 0 0 0 0 0 0 0 0 0 0 0 0 0 0 0 0 0 0 0 0 0 0 0 0 0 0 0 0 0 0 0 0 0

"177102_fr5" 0 0 0 0 0 0 0 0 0 0 0 0 0 0 0 0 0 0 0 0 0 0 0 0 0 0 0 0 0 0 0 0 0 0 0 0 0 0 0 1 0 0 0 0 3 2 2 3 2 2 0 2 0 1 2 2 0 1 1 3 0 0 0 0 0 0 0 0 0 0 0 0 0 0 0 0 0 0 0 0 0 0 0 0 0

"177368_fr1" 0 0 1 0 1 0 0 0 0 0 0 0 0 0 0 0 1 0 0 0 0 7 0 0 0 0 0 0 0 0 0 0 0 0 0 0 0 0 0 0 0 0 0 0 0 0 0 0 0 0 0 0 0 0 0 0 0 0 0 0 0 0 0 0 0 0 0 0 0 0 0 0 0 0 0 0 0 0 0 0 0 0 0 0 0

"177383_fr6" 0 0 4 0 3 0 0 0 0 0 2 0 2 0 0 0 0 0 0 3 3 0 3 3 5 0 2 0 3 1 2 4 4 0 0 0 3 8 0 6 0 0 0 0 0 0 0 0 0 0 0 0 0 0 0 0 0 0 0 0 0 0 0 0 0 0 0 0 0 0 0 0 0 0 0 0 0 0 0 0 0 0 0 0 0

"177973_fr2" 2 2 2 0 2 2 2 2 2 5 2 2 2 0 2 2 2 2 2 0 2 2 2 2 2 0 1 2 0 2 2 2 2 2 2 0 0 0 0 0 0 0 0 0 0 0 0 0 0 0 0 0 0 0 0 0 0 0 0 0 0 0 0 0 0 1 1 1 2 2 2 2 2 1 2 2 2 0 2 2 1 4 2 1 6

"177978_fr4" 1 0 0 0 0 0 0 0 0 2 0 0 0 0 0 0 0 0 0 0 0 0 0 0 0 0 0 0 0 0 0 0 0 0 0 0 0 0 0 0 0 0 0 0 0 0 0 0 0 0 0 0 0 0 0 0 0 0 0 0 0 0 0 0 0 0 0 0 0 0 0 0 0 0 0 0 0 0 0 0 0 0 0 0 0

"178091_fr2" 0 0 0 0 0 0 0 0 0 0 2 0 0 0 0 0 0 0 0 0 0 1 0 0 0 0 0 0 0 0 0 0 2 4 0 0 0 0 0 0 0 0 0 0 0 0 0 0 0 0 0 0 0 0 0 0 0 0 0 0 0 0 0 0 0 0 0 0 0 0 0 0 0 0 0 0 0 0 0 0 0 0 0 0 0

"178103_fr6" 0 0 0 0 0 0 0 0 0 0 0 0 0 0 0 0 0 0 0 0 0 0 0 0 0 0 2 0 0 0 0 0 0 0 0 0 0 0 0 0 0 0 0 0 0 0 0 0 0 0 0 0 0 0 0 0 0 0 0 0 0 0 0 0 0 0 0 0 0 0 0 0 0 0 0 0 0 0 0 0 0 0 0 0 0

"178132_fr5" 0 0 0 0 0 0 0 0 0 0 0 0 0 0 0 0 0 0 0 0 0 0 0 0 0 0 0 0 0 0 0 0 0 0 0 0 0 0 0 5 0 0 0 0 0 0 0 0 0 0 0 0 0 0 0 0 0 0 0 0 0 0 0 0 0 0 0 0 0 0 0 0 0 0 0 0 0 0 0 0 0 0 0 0 0

"178303_fr6" 0 0 0 0 0 0 0 0 0 0 0 0 0 0 0 0 0 0 0 0 0 0 0 0 0 0 0 0 0 0 0 0 0 0 0 0 0 0 0 0 0 0 0 0 0 0 0 0 0 0 0 0 0 0 0 0 0 0 0 0 0 0 0 0 0 0 0 0 0 0 0 0 0 0 0 1 2 0 0 0 0 0 0 0 1

"178309_fr3" 0 0 0 0 0 0 0 0 0 0 3 0 0 0 0 0 0 0 0 0 0 0 0 0 0 0 0 0 0 0 0 5 0 0 0 0 0 0 0 0 0 0 0 0 0 0 0 0 0 0 0 0 0 0 0 0 0 0 0 0 0 0 0 0 0 0 0 0 0 0 0 0 0 0 0 0 0 0 0 0 0 0 0 0 0

"178360_fr2" 0 0 0 0 0 0 0 0 0 0 0 0 0 0 0 0 0 0 0 0 0 0 0 0 0 0 0 0 0 0 0 0 0 0 0 0 0 0 0 0 0 0 0 0 0 0 0 0 0 0 0 0 0 0 0 0 0 0 0 0 0 0 0 0 0 0 8 0 0 0 0 5 5 0 0 0 0 0 0 0 0 0 0 0 0

"1784_fr6" 0 0 0 0 0 0 0 0 0 0 0 0 0 0 0 0 0 0 0 0 0 0 0 0 0 0 0 0 0 0 0 0 0 0 0 0 0 0 0 0 0 0 0 0 0 0 0 0 0 0 0 0 0 0 0 0 0 0 0 0 0 0 0 0 0 0 0 0 0 0 0 0 0 2 0 0 0 3 3 1 0 0 0 0 0

"17884_fr3" 2 3 0 0 9 4 0 5 0 0 1 0 0 12 7 0 6 2 9 8 8 6 7 10 2 10 4 6 8 13 4 0 5 8 3 0 0 0 0 0 0 0 0 0 0 0 0 0 0 0 0 0 0 0 0 0 0 0 0 0 0 0 0 0 0 0 0 0 0 0 0 0 0 0 0 0 0 1 0 0 0 0 0 2 0

"179252_fr2" 2 0 0 0 0 0 0 0 0 0 0 0 0 0 0 0 0 0 0 0 0 0 0 2 0 0 5 3 0 0 0 0 0 0 0 0 0 0 0 0 0 0 0 0 0 0 0 0 0 0 0 0 0 0 0 0 0 0 0 0 0 0 0 0 0 0 0 0 0 0 0 0 0 0 0 0 0 0 0 0 0 0 0 0 0

"179301_fr4" 3 0 0 4 0 0 3 4 4 0 0 4 0 0 4 4 3 5 0 0 0 4 0 4 5 0 0 0 4 0 0 0 0 0 4 0 0 0 0 0 0 0 0 0 0 0 0 0 0 0 0 0 0 0 0 0 0 0 0 0 0 0 0 0 0 0 0 0 0 0 0 0 0 0 0 0 0 0 0 0 0 0 0 0 0

"179325_fr3" 5 0 0 0 0 0 0 0 0 0 0 0 0 0 0 0 0 0 0 0 0 0 0 7 0 0 0 0 0 0 0 0 0 0 0 0 0 0 0 0 0 0 0 0 0 0 0 0 0 0 0 0 0 0 0 0 0 0 0 0 0 0 0 0 0 0 0 0 0 0 0 0 0 0 0 0 0 0 0 0 0 0 0 0 0

"17967_fr6" 0 0 0 0 0 0 0 0 0 0 0 0 0 0 0 0 0 0 0 0 0 0 0 0 0 3 0 0 0 0 0 0 0 0 0 0 0 0 0 0 0 0 0 0 0 0 0 0 0 0 0 0 0 0 0 0 0 0 0 0 0 0 0 0 0 0 0 0 0 0 0 0 0 0 0 0 0 0 0 0 0 0 0 0 0

"179995_fr3" 0 2 0 0 0 0 2 0 0 0 0 0 2 0 1 0 0 2 0 0 0 0 0 0 0 0 0 0 1 0 0 1 0 0 0 0 0 0 0 0 0 0 0 0 0 0 0 0 0 0 0 0 0 0 0 0 0 0 0 0 0 0 0 0 0 0 0 0 0 0 0 0 0 0 0 0 0 0 0 0 0 0 0 0 0

"180145_fr6" 0 3 0 0 0 0 0 0 0 0 0 0 0 0 0 0 0 0 0 0 0 0 0 0 0 0 0 0 0 0 0 0 0 0 0 0 0 0 0 0 0 0 0 0 0 0 0 0 0 0 0 0 0 0 0 0 0 0 0 0 0 0 0 0 0 0 0 0 0 0 0 0 0 0 0 0 0 0 0 0 0 0 0 0 0

"1803_fr2" 0 0 0 0 0 0 0 0 0 0 0 0 4 0 0 0 0 0 0 0 0 0 0 0 0 0 0 0 0 0 0 0 0 0 0 0 0 0 0 0 0 0 0 0 0 0 0 0 0 0 0 0 0 0 0 0 0 0 0 0 0 0 0 0 0 0 0 0 0 0 0 0 0 0 0 0 0 0 0 0 0 0 0 0 0

"180766_fr1" 0 0 0 0 0 0 0 0 0 0 0 0 0 0 0 0 0 0 0 0 0 0 0 0 0 0 0 0 0 2 0 0 0 0 0 0 0 0 0 0 0 0 0 0 0 0 0 0 0 0 0 0 0 0 0 0 0 0 0 0 0 0 0 0 0 0 0 0 0 0 0 0 0 0 0 0 0 0 0 0 0 0 0 0 0

"181254_fr1" 0 0 0 0 6 0 0 0 0 0 0 0 0 0 0 0 0 0 0 0 0 0 0 0 0 0 0 0 0 0 0 0 0 0 0 0 0 0 0 0 0 0 0 0 0 0 0 0 0 0 0 0 0 0 0 0 0 0 0 0 0 0 0 0 0 0 0 0 0 0 0 0 0 0 0 0 0 0 0 0 0 0 0 0 0

"181255_fr1" 0 0 0 0 0 0 0 0 0 0 0 3 0 0 0 0 0 2 0 0 0 0 0 0 0 0 1 0 1 2 0 0 0 0 0 2 2 2 0 0 0 0 0 0 0 0 0 0 0 0 0 0 0 0 0 0 0 0 0 0 0 0 0 0 0 0 0 0 0 0 0 0 0 0 0 0 0 0 0 0 0 0 0 0 0

"181267_fr5" 0 0 0 0 0 0 0 0 0 0 0 0 0 3 2 1 0 0 2 0 1 0 0 0 0 2 2 0 1 2 2 0 1 1 0 0 0 0 0 0 0 0 0 0 0 0 0 0 0 0 0 0 0 0 0 0 0 0 0 0 0 0 0 0 0 0 0 0 0 0 0 0 0 0 0 0 0 0 0 0 0 0 0 0 0

"181483_fr2" 0 5 0 0 4 0 8 7 0 0 0 0 8 1 3 8 6 7 10 3 5 5 7 0 2 5 0 0 4 4 1 7 7 5 0 0 0 0 0 0 0 0 0 0 0 0 0 0 0 0 0 0 0 0 0 0 0 0 0 0 0 0 0 0 0 0 0 1 0 0 0 3 1 2 2 0 1 1 1 2 0 0 1 1 0

"181526_fr4" 0 1 2 1 1 1 2 2 0 0 2 2 2 1 3 2 1 2 0 1 1 2 2 2 2 0 2 1 1 0 0 0 0 2 1 0 0 0 0 0 0 0 0 0 0 0 0 0 0 0 0 0 0 0 0 0 0 0 0 0 0 0 0 0 0 0 0 0 0 0 0 0 0 0 0 0 0 0 0 0 0 0 0 0 0

"181833_fr2" 0 0 0 0 0 0 0 0 0 4 0 0 3 0 0 0 0 0 0 0 2 0 0 0 2 0 0 0 0 0 0 0 0 0 0 0 0 0 0 0 0 0 0 0 0 0 0 0 0 0 0 0 0 0 0 0 0 0 0 0 0 0 0 0 0 0 0 0 0 0 0 0 0 0 0 0 0 0 0 0 0 0 0 0 0

"182086_fr1" 14 0 26 29 2 0 20 15 36 30 9 17 9 4 36 23 3 40 1 18 1 4 0 28 42 0 0 0 30 0 47 8 13 0 45 0 0 0 0 0 0 0 0 0 0 0 0 0 0 0 0 0 0 0 0 0 0 0 0 0 0 0 0 0 0 0 0 0 0 0 0 0 0 0 0 0 0 1 1 0 27 24 18 14 10

"182089_fr2" 3 0 0 0 1 0 0 0 0 0 0 4 0 0 0 0 0 0 2 0 0 2 1 0 0 3 0 0 0 0 1 0 0 1 0 0 0 0 0 0 0 0 0 0 0 0 0 0 0 0 0 0 0 0 0 0 0 0 0 0 0 0 0 0 0 0 0 0 0 0 0 0 0 0 0 0 0 0 0 0 0 2 0 0 0

"182115_fr3" 0 0 0 0 0 2 0 0 0 0 0 0 0 0 0 0 0 0 0 0 0 0 0 0 0 0 0 0 0 0 0 0 0 2 0 0 0 0 0 0 0 0 0 0 0 0 0 0 0 0 0 0 0 0 0 0 0 0 0 0 0 0 0 0 0 0 0 0 0 0 0 0 0 0 0 0 0 0 0 0 0 0 0 0 0

"182248_fr5" 0 0 0 0 0 3 0 0 0 0 0 0 0 0 0 0 0 0 0 0 0 0 0 0 0 0 0 0 0 3 0 0 0 3 0 0 0 0 0 0 0 0 0 0 0 0 0 0 0 0 0 0 0 0 0 0 0 0 0 0 0 0 0 0 0 0 0 0 0 0 0 0 0 0 0 0 0 0 0 0 0 0 0 0 0

"182450_fr5" 0 0 0 0 0 0 0 0 0 0 0 0 0 0 0 0 0 0 0 0 0 0 2 0 0 0 1 0 0 0 0 3 0 0 0 0 0 0 0 0 0 0 0 0 0 0 0 0 0 0 0 0 0 0 0 0 0 0 0 0 0 0 0 0 0 0 0 0 0 0 0 0 0 0 0 0 0 0 0 0 0 0 0 0 0

"182640_fr2" 0 0 0 0 0 0 0 0 0 3 0 0 0 0 0 0 0 1 0 0 0 0 0 0 1 0 0 0 0 0 0 0 0 0 0 0 0 0 0 0 0 0 0 0 0 0 0 0 0 0 0 0 0 0 0 0 0 0 0 0 0 0 0 0 0 0 0 0 0 0 0 0 0 0 0 0 0 0 0 0 0 0 0 0 0

"182902_fr1" 0 1 0 0 0 0 0 0 0 10 0 0 0 0 0 0 0 0 0 0 0 0 0 0 0 0 0 0 0 0 0 0 0 0 0 0 0 0 0 0 0 0 0 0 0 0 0 0 0 0 0 0 0 0 0 0 0 0 0 0 0 0 0 0 0 0 0 0 0 0 0 0 0 0 0 0 0 0 0 0 0 0 0 0 0

"183076_fr1" 0 0 0 0 1 0 0 0 0 0 0 0 0 0 0 0 0 0 0 0 0 0 3 2 0 0 2 0 0 0 3 0 2 1 0 0 0 0 0 0 0 0 0 0 0 0 0 0 0 0 0 0 0 0 0 0 0 0 0 0 0 0 0 0 0 0 0 0 0 0 0 0 0 0 0 0 0 0 0 0 0 0 0 0 0

"183084_fr4" 0 0 0 0 0 0 0 0 6 0 0 0 0 0 0 4 5 0 0 0 4 3 0 0 0 0 0 0 0 0 0 0 4 3 0 0 0 0 0 0 0 0 0 0 0 0 0 0 0 0 0 0 0 0 0 0 0 0 0 0 0 0 0 0 0 0 0 0 0 0 0 0 0 0 0 0 0 0 0 0 0 0 0 0 0

"183169_fr5" 0 0 0 0 0 0 0 0 0 0 0 0 0 0 0 0 0 0 0 0 0 0 0 0 0 0 0 0 0 0 0 0 0 0 0 0 0 0 0 0 0 0 0 0 0 0 0 0 0 0 0 0 0 0 0 0 0 0 0 0 0 0 0 0 0 0 0 0 0 0 0 0 0 0 0 2 1 0 0 0 0 0 0 0 0

"183846_fr1" 1 0 0 2 0 7 5 0 0 3 0 0 0 0 0 0 0 0 0 0 2 0 2 0 6 1 0 0 0 0 5 0 3 0 0 0 0 0 0 0 0 0 0 0 0 0 0 0 0 0 0 0 0 0 0 0 0 0 0 0 0 0 0 0 0 0 0 0 0 0 0 0 0 5 0 0 0 3 0 2 0 2 0 0 0

"18386_fr3" 0 0 0 0 0 0 0 0 0 0 0 0 0 0 0 0 0 0 0 0 0 0 0 0 0 0 0 0 0 0 0 0 0 0 1 0 0 0 0 0 0 0 0 0 0 0 0 0 0 0 0 0 0 0 0 0 0 0 0 0 0 0 0 0 0 0 0 0 0 0 0 0 0 0 0 0 0 0 0 0 0 0 0 0 0

"183941_fr1" 0 0 0 0 0 0 0 0 0 0 0 0 0 0 4 0 0 0 0 0 0 0 0 0 0 0 0 0 0 0 0 0 0 0 0 0 0 0 0 0 0 0 0 0 0 0 0 0 0 0 0 0 0 0 0 0 0 0 0 0 0 0 0 0 0 0 0 0 0 0 0 0 0 0 0 0 0 0 0 0 0 0 0 0 0

"184449_fr2" 0 0 0 0 0 0 0 0 0 0 0 0 0 0 0 0 0 0 0 0 0 0 0 3 0 0 0 0 0 0 0 0 0 0 0 0 0 0 0 0 0 0 0 0 0 0 0 0 0 0 0 0 0 0 0 0 0 0 0 0 0 0 0 0 0 0 0 0 0 0 0 0 0 0 0 0 0 0 0 0 0 0 0 0 0

"184643_fr2" 0 0 0 0 0 0 0 0 0 0 0 0 0 0 0 0 0 0 0 0 0 0 0 0 0 0 0 0 0 0 0 0 0 0 0 0 0 0 0 2 0 0 0 0 4 0 4 0 0 5 3 0 0 3 0 0 7 0 0 0 0 0 0 0 0 0 0 0 0 0 0 0 0 0 0 0 0 0 0 0 0 0 0 0 0

"18473_fr2" 2 0 1 8 1 5 0 3 2 0 0 0 0 15 13 0 0 0 1 0 3 3 1 1 2 5 0 3 6 3 0 2 3 5 0 0 0 0 0 0 0 0 0 0 0 0 0 0 0 0 0 0 0 0 0 0 0 0 0 0 0 0 0 0 0 0 0 0 0 0 0 0 0 0 0 0 0 0 0 0 0 0 0 0 0

"18473_fr3" 0 0 0 0 0 0 0 0 0 0 0 0 0 0 0 0 0 0 0 0 0 0 0 0 0 0 0 0 0 0 0 0 0 0 0 0 0 0 0 3 0 0 0 0 0 0 0 0 0 0 0 0 0 0 0 0 0 0 0 0 0 0 0 0 0 0 0 0 0 0 0 0 0 0 0 0 0 0 0 0 0 0 0 0 0

"18500_fr3" 0 0 0 0 0 0 0 0 0 0 0 0 0 0 0 0 0 0 0 0 0 0 0 0 0 0 0 0 0 0 0 0 0 0 0 8 7 3 0 0 0 0 0 0 0 0 0 0 0 0 0 0 0 0 0 0 0 0 0 0 0 0 0 0 0 4 4 0 4 0 0 0 0 5 4 2 3 3 0 2 6 0 3 0 0

"18513_fr2" 0 0 0 0 0 0 0 0 0 0 0 0 0 0 0 0 0 0 0 0 0 0 0 0 0 0 0 0 0 0 0 0 0 0 0 2 1 0 0 0 0 0 1 0 0 0 0 0 0 0 0 0 0 0 0 0 0 0 0 0 0 0 0 0 0 0 0 0 0 0 0 0 0 0 0 0 0 0 0 0 0 0 0 0 0

"185442_fr4" 2 2 2 0 3 0 2 0 0 0 2 2 2 3 2 2 2 2 2 0 2 2 3 2 2 4 3 2 3 4 1 3 0 3 0 0 0 0 0 0 0 0 0 0 0 0 0 0 0 0 0 0 0 0 0 0 0 0 0 0 0 0 0 0 0 0 0 0 0 0 0 0 0 0 0 0 0 0 0 0 0 0 0 0 0

"185484_fr5" 0 0 0 0 0 0 0 0 0 0 0 0 0 0 0 0 0 0 0 0 0 0 0 0 0 0 0 0 0 0 0 0 0 0 0 0 0 0 0 0 0 0 3 0 0 0 0 0 0 0 0 0 0 0 0 0 0 0 0 0 0 0 0 0 0 0 0 0 0 0 0 0 0 0 0 0 0 0 0 0 0 0 0 0 0

"185498_fr2" 0 0 6 1 0 5 0 5 0 0 0 0 0 0 1 3 0 0 3 0 0 0 3 0 0 0 2 3 5 3 0 0 1 2 0 0 0 0 0 0 0 0 0 0 0 0 0 0 0 0 0 0 0 0 0 0 0 0 0 0 0 0 0 0 0 0 0 0 0 0 0 0 0 0 0 0 0 0 0 0 0 0 0 0 0

"185506_fr1" 0 1 0 0 0 1 2 0 0 0 2 0 0 2 0 0 2 0 2 1 1 0 3 0 0 3 2 0 2 3 2 2 4 2 0 0 0 0 0 0 0 0 0 0 0 0 0 0 0 0 0 0 0 0 0 0 0 0 0 0 0 0 0 0 0 0 3 2 3 0 0 3 0 0 3 4 0 4 1 5 1 2 0 0 3

"18564_fr4" 0 0 0 0 0 0 0 0 0 0 0 0 0 0 0 0 0 0 0 0 0 0 0 0 0 0 0 0 0 0 0 0 0 0 0 0 0 0 0 0 0 0 0 0 0 0 0 0 0 0 0 0 0 0 0 0 0 0 0 0 0 0 0 0 0 0 0 0 0 0 0 0 0 0 0 0 0 0 0 0 0 0 2 4 1

"185800_fr3" 0 0 0 0 0 0 0 0 0 0 0 0 4 0 0 0 0 0 0 0 0 0 0 0 0 0 0 0 0 0 0 0 0 0 0 0 0 0 0 0 0 0 0 0 0 0 0 0 0 0 0 0 0 0 0 0 0 0 0 0 0 0 0 0 0 0 0 0 0 0 0 0 0 0 0 0 2 0 0 0 1 0 0 0 0

"18609_fr3" 0 0 0 0 5 0 0 0 0 0 0 0 0 0 0 0 0 0 0 0 0 0 0 0 0 0 0 0 0 0 0 0 0 0 0 0 0 0 0 0 0 0 0 0 0 0 0 0 0 0 0 0 0 0 0 0 0 0 0 0 0 0 0 0 0 0 0 0 0 0 0 0 0 0 0 0 0 0 0 0 0 0 0 0 0

"186112_fr4" 0 0 0 0 0 0 0 0 0 0 0 0 0 0 0 0 0 0 0 0 0 0 0 0 0 0 0 0 0 0 0 0 0 0 0 0 0 0 0 2 0 0 0 0 0 0 3 0 0 0 0 0 0 0 1 0 0 0 0 0 0 0 0 0 0 0 0 0 0 0 0 0 0 0 0 0 0 0 0 0 0 0 0 0 0

"186168_fr5" 0 4 0 0 0 0 2 0 0 0 0 0 0 0 0 0 0 0 0 0 0 2 0 0 0 0 0 0 2 0 0 0 0 0 0 0 0 0 0 0 0 0 0 0 0 0 0 0 0 0 0 0 0 0 0 0 0 0 0 0 0 0 0 0 0 0 0 0 0 0 0 0 0 0 0 0 0 0 0 0 0 0 0 0 0

"186195_fr5" 0 1 0 1 1 0 2 1 0 0 0 0 2 0 4 2 0 0 0 2 3 2 3 2 1 2 2 3 1 2 2 2 0 0 1 0 0 0 0 0 0 0 0 0 0 0 0 0 0 0 0 0 0 0 0 0 0 0 0 0 0 0 0 0 0 0 0 0 0 0 0 0 0 0 0 0 0 0 0 0 0 0 0 0 0

"186201_fr3" 1 1 2 3 3 4 5 4 2 2 2 0 5 0 0 0 1 0 3 3 3 0 5 0 0 1 3 8 2 2 2 2 0 4 0 0 0 0 0 2 0 0 1 0 0 0 0 0 0 0 0 0 0 0 0 0 0 0 0 0 0 0 0 0 0 0 0 0 0 0 0 0 0 0 0 1 3 6 4 3 4 1 1 2 0

"186210_fr5" 0 0 0 0 0 0 0 0 0 0 0 0 0 0 0 0 0 0 0 0 0 0 0 0 0 0 0 0 0 0 0 0 0 0 0 0 0 0 0 0 0 0 0 0 0 0 0 0 0 0 0 0 0 0 0 0 0 0 0 0 0 0 0 0 0 0 0 0 0 0 0 0 0 0 0 1 3 0 0 0 0 0 0 0 0

"186237_fr2" 0 0 0 0 0 0 0 0 0 0 0 0 0 0 0 0 0 0 0 0 0 0 0 0 2 0 0 0 0 0 0 0 0 0 0 0 0 0 0 0 0 0 0 0 0 0 0 0 0 0 0 0 0 0 0 0 0 0 0 0 0 0 0 0 0 0 0 0 0 0 0 0 0 0 0 0 0 0 0 0 0 0 0 0 0

"186340_fr4" 0 0 0 0 0 0 0 0 0 0 0 0 0 0 0 0 0 0 0 0 0 0 0 0 0 0 0 0 0 0 0 0 0 0 0 0 0 0 0 0 0 0 0 0 0 0 0 0 1 0 0 0 0 0 0 0 6 2 1 0 0 0 0 0 0 0 0 0 0 0 0 0 0 0 0 0 0 0 0 0 0 0 0 0 0

"186872_fr3" 0 0 0 0 0 0 0 0 0 0 0 0 0 8 0 0 0 0 0 0 0 6 0 0 5 0 0 0 0 0 0 0 0 0 4 0 0 0 0 0 0 0 0 0 0 0 0 0 0 0 0 0 0 0 0 0 0 0 0 0 0 0 0 0 0 0 0 0 0 0 0 0 0 0 0 0 0 0 0 0 0 0 0 0 0

"187499_fr2" 2 4 0 0 0 0 0 0 0 0 5 0 0 0 0 3 0 0 0 0 4 4 0 3 0 0 4 0 5 0 5 4 2 3 0 0 0 0 0 0 0 0 0 0 0 0 0 0 0 0 0 0 0 0 0 0 0 0 0 0 0 0 0 0 0 0 0 0 0 0 0 0 0 0 0 0 2 2 4 0 0 0 0 0 3

"187507_fr1" 0 0 0 0 0 0 0 0 0 0 0 0 0 0 0 0 0 0 0 0 0 0 0 0 0 0 0 0 0 0 0 0 0 0 0 0 1 0 0 0 7 0 0 0 0 0 0 0 0 0 0 0 0 0 0 0 0 0 0 0 0 0 0 0 0 0 0 0 0 0 0 0 0 0 0 0 0 0 0 0 0 0 0 0 0

"187519_fr2" 0 0 0 0 0 0 0 0 0 0 0 0 0 0 0 0 0 0 0 0 0 0 0 0 0 0 0 0 0 0 0 9 0 0 0 0 0 0 0 0 0 0 0 0 0 0 0 0 0 0 0 0 0 0 0 0 0 0 0 0 0 0 0 0 0 0 0 0 0 0 0 0 0 0 0 0 0 0 0 0 0 0 0 0 0

"187564_fr1" 3 3 4 0 0 0 1 0 0 0 0 0 0 0 0 0 4 0 2 0 0 1 0 4 2 2 2 0 0 0 0 0 0 0 0 0 0 0 0 0 0 0 0 0 0 0 0 0 0 0 0 0 0 0 0 0 0 0 0 0 0 0 0 0 0 0 0 0 0 0 0 0 0 0 0 0 0 0 0 0 0 0 0 0 0

"187640_fr4" 0 0 3 2 0 2 0 0 0 0 0 0 0 0 0 0 0 0 0 0 0 0 0 0 0 0 0 0 0 1 1 0 0 0 0 0 0 0 0 0 0 0 0 0 0 0 0 0 0 0 0 0 0 0 0 0 0 0 0 0 0 0 0 0 0 0 0 0 0 0 0 0 0 0 0 0 0 0 0 0 0 0 0 0 0

"188129_fr1" 0 0 0 0 0 0 0 0 0 0 0 0 0 0 0 0 0 0 0 0 1 0 2 1 0 3 0 0 0 0 0 0 0 0 0 0 0 0 0 0 0 0 0 0 0 0 0 0 0 0 0 0 0 0 0 0 0 0 0 0 0 0 0 0 0 0 0 0 0 0 0 0 0 0 0 0 0 0 0 0 0 0 0 0 0

"188130_fr2" 0 0 2 0 2 1 0 0 0 0 2 0 0 0 2 0 2 0 1 4 0 0 4 6 0 1 5 0 4 0 0 5 2 3 1 0 0 0 0 0 0 0 0 0 0 0 0 0 0 0 0 0 0 0 0 0 0 0 0 0 0 0 0 0 0 0 0 0 0 0 0 0 0 0 0 0 0 0 0 0 0 0 0 0 0

"188208_fr1" 0 0 0 0 0 0 0 0 0 0 0 0 0 0 0 0 0 0 0 0 0 0 0 0 0 0 0 0 0 2 0 3 0 0 0 0 0 0 0 0 0 0 0 0 0 0 0 0 0 0 0 0 0 0 0 0 0 0 0 0 0 0 0 0 0 0 0 0 0 0 0 0 0 0 0 0 0 0 0 0 0 0 0 0 0

"188212_fr5" 0 0 0 0 0 0 0 0 0 0 0 0 0 0 0 0 0 0 0 0 0 0 0 0 0 0 7 0 0 0 0 2 0 0 0 0 0 0 0 0 0 0 0 0 0 0 0 0 0 0 0 0 0 0 0 0 0 0 0 0 0 0 0 0 0 0 0 0 0 0 0 0 0 0 0 0 0 0 0 0 0 0 0 0 0

"188305_fr3" 0 7 8 9 7 4 0 8 0 0 3 0 0 7 2 0 0 0 6 4 0 0 5 5 0 4 11 3 10 2 0 9 0 0 0 0 0 0 0 0 0 0 0 0 0 0 0 0 0 0 0 0 0 0 0 0 0 0 0 0 0 0 0 0 0 0 0 0 0 0 0 0 0 0 0 0 0 0 0 0 0 0 0 0 0

"188661_fr3" 0 0 0 0 0 0 0 0 0 0 0 0 0 0 0 0 0 0 0 0 0 0 0 0 0 0 0 0 0 0 0 0 0 0 0 0 0 3 4 0 4 0 4 4 2 4 0 5 0 2 0 0 4 0 2 4 3 2 2 0 0 4 1 4 4 3 3 4 4 4 0 0 0 0 0 0 0 0 0 0 0 0 0 0 0

"188696_fr1" 0 0 0 0 0 0 0 0 0 0 0 0 0 0 0 0 0 0 0 1 0 0 7 0 0 0 0 0 0 0 0 0 0 0 0 0 0 0 0 0 0 0 0 0 0 0 0 0 0 0 0 0 0 0 0 0 0 0 0 0 0 0 0 0 0 0 0 0 0 0 0 0 0 0 0 0 0 0 0 0 0 0 0 0 0

"188731_fr4" 0 0 0 0 0 0 0 0 0 0 0 0 0 0 0 0 0 0 0 0 0 0 0 0 0 0 0 0 0 0 0 0 0 0 0 0 0 0 0 0 0 0 0 0 0 0 0 0 0 0 0 0 0 0 0 0 0 0 0 0 0 0 0 0 0 0 0 0 0 0 0 0 0 0 0 0 4 2 2 2 2 4 4 2 4

"188740_fr6" 0 6 6 3 6 5 0 4 0 0 0 6 0 4 2 0 6 0 7 3 6 6 7 4 0 6 3 0 5 5 0 0 0 2 6 0 0 0 0 0 0 0 0 0 0 0 0 0 0 0 0 0 0 0 0 0 0 0 0 0 0 0 0 0 0 0 0 0 0 0 0 0 0 0 0 0 0 0 0 0 0 0 0 2 0

"189036_fr5" 0 0 0 0 0 0 0 0 0 0 0 0 0 0 0 0 0 0 5 0 0 0 0 0 0 0 0 5 0 0 0 0 0 0 0 0 0 0 0 0 0 0 0 0 0 0 0 0 0 0 0 0 0 0 0 0 0 0 0 0 0 0 0 0 0 0 0 0 0 0 0 0 0 0 0 0 0 0 0 0 0 0 0 0 0

"189299_fr5" 0 0 0 0 0 0 0 0 0 0 0 4 0 0 0 0 0 0 0 0 0 0 0 0 0 0 0 0 0 0 0 0 0 0 0 0 0 0 0 0 0 0 0 0 0 0 0 0 0 0 0 0 0 0 0 0 0 0 0 0 0 0 0 0 0 0 0 0 0 0 0 0 0 0 0 0 0 0 0 0 0 0 0 0 0

"189441_fr6" 0 0 0 0 0 0 0 0 0 1 0 0 0 0 0 0 0 0 0 2 0 0 0 0 1 0 0 0 0 0 0 2 0 0 0 0 0 0 0 0 0 0 0 0 0 0 0 0 0 0 0 0 0 0 0 0 0 0 0 0 0 0 0 0 0 0 0 0 0 0 0 0 0 0 0 0 0 0 0 0 0 0 0 0 0

"189460_fr4" 0 0 1 0 0 0 0 0 0 0 3 1 0 0 8 0 0 0 1 6 2 1 2 0 0 1 8 3 2 0 2 4 8 7 2 0 0 0 0 0 0 0 0 0 0 0 0 0 0 0 0 0 0 0 0 0 0 0 0 0 0 0 0 0 0 0 0 0 0 0 0 0 0 0 0 0 0 0 0 0 0 0 0 0 0

"18968_fr2" 0 0 0 0 0 0 0 0 0 0 0 0 0 0 0 0 0 0 0 0 0 0 0 0 0 0 0 0 0 0 0 0 0 0 0 0 0 2 0 4 0 0 0 0 1 0 2 0 0 0 0 0 0 5 0 0 0 0 0 0 0 0 0 0 0 0 0 0 0 0 0 0 0 0 0 0 0 0 0 0 0 0 0 0 0

"189695_fr2" 2 0 0 0 2 0 0 0 0 0 0 0 0 0 0 0 0 0 1 0 0 0 4 0 0 0 0 1 0 3 0 0 0 0 0 0 0 0 0 0 0 0 0 0 0 0 0 0 0 0 0 0 0 0 0 0 0 0 0 0 0 0 0 0 0 0 0 0 0 0 0 0 0 0 0 0 0 0 0 0 0 0 0 0 0

"189707_fr2" 0 0 0 0 0 0 0 0 0 0 0 0 0 0 0 0 0 0 0 0 0 0 0 0 0 0 0 0 0 0 0 0 0 0 0 0 0 0 2 0 0 0 0 2 0 2 0 0 3 0 3 3 1 2 0 2 0 0 3 0 0 0 0 0 0 0 0 0 0 0 0 0 0 0 0 0 0 0 0 0 0 0 0 0 0

"190020_fr5" 0 0 0 0 0 0 0 0 0 2 0 0 0 0 1 1 0 0 0 0 0 0 0 0 2 0 0 0 0 0 1 0 0 0 2 0 0 0 0 0 0 0 0 0 0 0 0 0 0 0 0 0 0 0 0 0 0 0 0 0 0 0 0 0 0 0 0 0 0 0 0 0 0 0 0 0 0 0 0 0 0 0 0 0 0

"19019_fr6" 0 0 0 0 0 0 0 0 0 0 0 0 0 0 0 0 0 0 0 0 0 0 0 0 0 0 2 0 0 0 0 0 0 0 0 0 0 0 0 0 0 0 0 0 0 0 0 0 0 0 0 0 0 0 0 0 0 0 0 0 0 0 0 0 0 0 0 0 0 0 0 0 0 0 0 0 0 0 0 0 0 0 0 0 0

"190594_fr3" 0 0 0 0 0 1 3 0 0 0 0 0 2 0 0 0 0 0 0 0 0 0 0 0 0 0 2 0 0 0 0 0 0 0 0 0 0 0 0 0 0 0 0 0 0 0 0 0 0 0 0 0 0 0 0 0 0 0 0 0 0 0 0 0 0 0 0 0 0 0 0 0 0 0 0 0 0 0 0 0 0 0 0 0 0

"190624_fr5" 1 0 2 6 2 8 2 8 0 0 2 15 2 2 14 2 5 2 3 1 4 2 1 2 0 6 7 2 6 3 9 2 13 2 9 0 5 1 0 2 0 1 0 0 0 0 0 0 0 0 0 0 0 0 4 0 0 0 4 0 0 0 0 0 0 0 5 0 0 0 4 3 5 0 0 5 8 4 1 2 0 0 2 1 2

"191065_fr3" 6 5 7 5 5 2 7 7 7 3 6 6 6 7 6 5 7 0 6 6 7 6 9 6 6 7 6 6 6 6 6 6 5 6 5 0 0 0 0 0 0 0 0 0 0 0 0 0 0 0 0 0 0 0 0 0 0 0 0 0 0 0 0 0 0 0 0 0 0 0 0 0 0 0 0 0 0 0 0 0 0 0 0 0 0

"19128_fr1" 0 0 0 0 0 0 0 0 0 0 0 0 0 0 0 0 0 0 0 0 0 0 0 0 0 0 0 0 0 0 0 0 0 0 0 0 0 0 3 4 0 0 6 5 0 5 0 6 5 8 8 9 6 5 0 5 7 5 7 0 0 0 0 0 0 0 0 0 0 0 0 0 0 0 0 0 0 0 0 0 0 0 0 0 0

"191476_fr2" 4 4 17 19 14 21 4 19 5 0 11 5 0 4 3 5 4 0 4 11 4 3 4 10 0 12 24 4 19 19 11 18 8 12 4 0 0 5 0 6 0 0 1 0 0 0 0 0 0 0 0 0 0 0 0 0 1 2 0 0 0 0 0 0 0 1 2 8 2 0 2 2 2 4 2 2 11 3 2 9 0 0 0 0 0

"191492_fr6" 1 0 0 0 0 0 0 0 0 0 0 2 0 0 0 0 0 0 0 0 0 0 0 0 0 0 0 0 0 0 0 0 0 0 0 0 0 0 0 0 0 0 0 0 0 0 0 0 0 0 0 0 0 0 0 0 0 0 0 0 0 0 0 0 0 0 0 0 0 0 0 0 0 0 0 0 0 0 0 0 0 0 0 0 0

"191493_fr6" 1 0 1 0 2 1 5 4 0 6 4 0 0 1 1 4 0 2 3 0 2 0 2 0 6 1 0 0 0 6 6 0 2 1 9 0 0 0 0 0 0 0 0 0 0 0 0 0 0 0 0 0 0 0 0 0 0 0 0 0 0 0 0 0 0 1 2 4 0 0 4 4 4 3 2 5 6 0 2 5 0 0 2 4 5

"191505_fr1" 0 0 0 0 0 0 0 0 0 0 0 0 0 0 0 0 0 0 0 0 0 0 0 0 0 0 0 0 0 0 0 0 0 0 0 0 0 0 0 0 0 0 0 1 0 0 0 0 0 4 1 0 0 1 0 0 1 0 3 1 0 0 0 0 0 0 0 0 0 0 0 0 0 0 0 0 0 0 0 0 0 0 0 0 0

"1917_fr2" 0 4 0 0 0 2 0 0 0 0 0 4 0 0 4 0 2 0 5 1 5 1 2 0 0 5 4 0 0 2 1 8 2 7 5 0 0 0 0 0 0 0 0 0 0 0 0 0 0 0 0 0 0 0 0 0 0 0 0 0 0 0 0 0 0 0 0 0 0 0 0 0 0 0 0 0 0 0 0 0 0 0 0 0 0

"191880_fr1" 0 0 0 0 0 0 0 0 0 0 0 0 0 0 0 0 0 0 0 0 0 0 0 0 0 0 0 0 0 0 0 0 0 0 0 0 0 0 0 0 0 0 0 0 0 0 0 0 0 0 0 0 0 0 0 0 0 0 0 0 0 0 0 0 0 0 0 0 0 0 0 0 0 0 0 0 0 2 0 0 0 0 0 0 0

"191891_fr5" 0 0 0 0 0 0 0 0 0 0 0 0 0 0 0 0 0 0 0 0 0 0 0 0 0 0 0 0 0 0 0 0 0 0 0 0 0 0 4 0 0 4 0 0 0 0 0 0 0 0 0 0 0 0 0 0 0 0 0 0 0 0 0 0 4 0 0 0 4 0 4 4 0 0 4 0 1 0 0 0 0 0 0 0 0

"191946_fr3" 0 0 0 0 0 0 0 0 0 0 0 0 0 0 0 0 0 0 0 0 0 0 0 0 0 0 0 0 0 0 0 0 0 0 0 0 0 0 0 4 2 0 0 2 1 0 0 1 1 0 2 0 0 2 0 0 0 1 0 2 0 0 0 0 0 0 0 0 0 0 0 0 0 0 0 0 0 0 0 0 0 0 0 0 0

"19196_fr3" 0 5 7 9 10 8 7 1 9 0 6 4 0 1 0 0 2 7 1 0 0 3 5 3 0 12 10 6 1 0 7 0 7 1 1 0 0 0 0 0 0 0 0 0 0 0 0 0 0 0 0 0 0 0 0 0 0 0 0 0 0 0 0 0 0 0 0 0 0 0 0 0 0 0 0 0 0 0 0 0 0 0 0 0 0

"192148_fr6" 0 2 2 2 1 1 2 2 2 2 2 1 2 0 2 1 0 2 1 2 2 0 0 2 2 0 2 2 0 0 1 2 0 2 1 0 0 0 0 0 0 0 0 0 0 0 0 0 0 0 0 0 0 0 0 0 0 0 0 0 0 0 0 0 0 0 0 0 0 0 0 0 0 0 0 0 0 0 0 0 0 0 0 0 0

"192326_fr6" 0 0 0 0 0 0 0 0 0 0 0 0 0 0 0 0 0 0 0 0 0 0 0 0 0 0 0 0 0 0 0 5 0 0 0 0 0 0 0 0 0 0 0 0 0 0 0 0 0 0 0 0 0 0 0 0 0 0 0 0 0 0 0 0 0 0 0 0 0 0 0 0 0 0 0 0 0 0 0 0 0 3 2 0 2

"19261_fr6" 0 0 0 0 0 0 0 0 0 0 0 0 0 0 0 0 0 0 0 0 0 0 0 0 0 0 0 0 0 0 0 0 0 0 0 0 0 1 21 0 1 0 2 0 14 15 18 9 26 18 18 36 18 28 26 15 20 17 36 36 15 23 27 19 18 0 0 0 0 2 0 0 0 0 0 0 0 0 0 0 0 0 0 0 0

"192652_fr5" 0 0 0 0 0 0 0 0 0 2 0 0 5 0 0 0 0 1 0 0 0 0 0 0 0 0 0 0 0 0 0 0 0 0 0 0 0 0 0 0 0 0 0 0 0 0 0 0 0 0 0 0 0 0 0 0 0 0 0 0 0 0 0 0 0 0 0 0 0 0 0 0 0 0 0 0 0 0 0 0 0 0 0 0 0

"192686_fr4" 0 0 0 0 0 0 0 0 0 0 0 0 0 0 0 0 0 0 0 0 0 0 0 0 0 0 0 0 0 0 0 0 0 0 0 2 0 2 0 0 0 0 0 0 3 0 0 0 0 0 0 0 0 0 0 0 0 0 0 0 0 0 0 0 0 0 0 0 0 0 0 0 0 0 0 0 0 0 0 0 0 0 0 0 0

"192695_fr3" 0 11 17 18 11 21 2 16 11 0 13 2 2 11 2 3 0 1 12 1 15 2 17 9 9 12 18 13 19 12 11 11 14 4 11 0 0 1 2 1 4 0 4 5 0 0 0 3 0 2 0 2 2 0 0 2 0 2 0 0 0 0 0 0 0 0 0 0 0 0 0 0 2 3 0 0 9 1 7 0 4 5 1 2 1

"192788_fr6" 2 0 0 0 2 0 0 0 0 0 0 0 1 0 0 0 0 0 4 0 0 0 0 0 0 0 2 0 0 0 0 2 0 0 0 0 0 0 0 0 0 0 0 0 0 0 0 0 0 0 0 0 0 0 0 0 0 0 0 0 0 0 0 0 0 0 0 0 0 0 0 0 0 0 0 0 0 0 0 0 0 0 0 0 0

"192906_fr4" 0 0 0 0 1 2 0 0 3 0 0 0 0 0 0 0 0 0 0 0 0 0 0 0 0 0 0 0 2 0 0 1 0 0 0 0 0 0 0 0 0 0 0 0 0 0 0 0 0 0 0 0 0 0 0 0 0 0 0 0 0 0 0 0 0 0 0 0 0 0 0 0 0 0 0 0 0 0 0 0 0 0 0 0 0

"193455_fr6" 0 0 0 0 0 0 0 0 0 3 0 0 1 0 0 0 0 0 0 0 0 0 0 0 0 0 0 0 0 0 0 0 0 0 0 0 0 0 0 0 0 0 0 0 0 0 0 0 0 0 0 0 0 0 0 0 0 0 0 0 0 0 0 0 0 0 0 0 0 0 0 0 0 0 0 0 0 0 0 0 0 0 0 0 0

"193782_fr4" 0 0 0 0 0 0 0 0 0 0 0 0 0 0 0 0 0 0 0 0 0 0 0 0 0 0 0 0 0 0 0 0 0 0 0 0 0 0 0 2 0 0 0 0 0 0 0 0 0 0 0 0 0 0 0 0 0 0 0 0 0 0 0 0 0 0 0 0 0 0 0 0 0 0 0 1 0 1 3 1 0 0 0 0 0

"193819_fr5" 0 0 0 0 0 0 0 0 0 0 0 0 0 0 0 0 0 0 0 0 0 0 0 0 0 0 0 0 0 0 0 0 0 0 0 0 0 0 0 0 0 0 0 0 0 0 0 0 0 0 0 0 0 0 0 0 0 0 0 0 0 0 0 0 0 0 0 0 0 0 0 0 0 0 0 0 5 0 6 0 0 0 0 0 0

"194190_fr3" 0 0 0 0 0 0 0 0 0 0 0 0 0 0 0 0 0 0 0 0 0 0 0 0 0 0 3 0 0 0 0 0 0 0 0 0 0 0 0 0 0 0 0 0 0 0 0 0 0 0 0 0 0 0 0 0 0 0 0 0 0 0 0 0 0 0 0 0 0 0 0 0 0 0 0 0 0 0 0 0 0 0 0 0 0

"194448_fr3" 0 0 0 0 0 0 0 0 0 0 0 0 0 0 0 0 0 0 0 0 0 0 0 0 0 0 0 0 0 0 0 0 0 0 0 0 0 0 0 0 0 0 0 0 0 0 0 0 0 0 0 0 0 0 0 0 0 0 0 0 0 0 0 0 0 0 0 0 0 0 0 0 0 0 0 2 1 4 1 0 0 0 0 0 2

"194469_fr4" 0 0 0 0 0 0 0 0 0 0 0 0 0 0 0 0 0 0 0 0 0 0 0 0 0 3 0 0 3 2 0 0 0 0 0 0 0 0 0 0 0 0 0 0 0 0 0 0 0 0 0 0 0 0 0 0 0 0 0 0 0 0 0 0 0 0 0 0 0 0 0 0 0 0 0 0 0 0 0 0 0 0 0 0 0

"194500_fr4" 0 0 0 0 0 0 0 0 0 0 0 0 0 0 0 0 0 0 0 0 0 0 0 0 0 0 0 0 0 0 2 0 1 0 0 0 0 0 0 0 0 0 0 0 0 0 0 0 0 0 0 0 0 0 0 0 0 0 0 0 0 0 0 0 0 0 0 0 0 0 0 0 0 0 0 0 0 0 0 0 0 0 0 0 0

"19453_fr3" 0 0 0 0 1 0 0 0 0 0 0 0 0 0 0 0 0 0 0 0 0 0 0 0 0 0 0 0 0 0 0 0 0 0 0 0 0 0 0 0 0 0 0 0 0 0 0 0 0 0 0 0 0 0 0 0 0 0 0 0 0 0 0 0 0 0 0 0 0 0 0 0 0 0 0 0 0 0 0 0 0 0 0 0 0

"19465_fr3" 0 0 0 0 0 0 0 0 0 0 0 0 0 2 0 0 0 0 3 0 0 0 0 0 0 0 0 0 0 3 0 0 0 0 0 0 0 0 0 0 0 0 0 0 0 0 0 0 0 0 0 0 0 0 0 0 0 0 0 0 0 0 0 0 0 0 0 0 0 0 0 0 0 0 0 0 0 0 0 0 0 0 0 0 0

"194720_fr2" 0 0 0 0 0 0 3 3 0 0 1 0 3 0 0 1 0 1 0 0 0 0 0 1 0 0 0 3 1 0 1 0 1 1 0 0 0 0 0 0 0 0 0 0 0 0 0 0 0 0 0 0 0 0 0 0 0 0 0 0 0 0 0 0 0 0 0 0 0 0 0 0 0 0 0 0 0 0 0 0 0 0 0 0 0

"194758_fr3" 0 0 0 0 0 0 0 0 0 0 0 0 0 0 0 0 0 0 0 0 0 0 0 0 0 0 0 0 0 0 0 0 0 0 0 1 7 17 36 12 29 17 30 24 28 39 37 30 47 30 43 43 56 40 45 50 50 48 67 33 71 36 70 71 50 21 17 22 11 42 0 2 0 0 0 0 0 0 0 0 5 12 0 0 9

"194768_fr2" 0 0 0 0 0 0 0 0 0 0 0 0 0 0 0 0 0 0 0 0 0 0 0 0 0 0 0 0 0 0 0 0 0 0 0 0 0 0 0 0 0 0 0 0 0 0 0 0 0 0 0 0 0 0 0 0 0 0 0 0 0 0 0 0 0 0 0 0 0 0 0 0 0 0 0 0 0 1 1 0 0 2 1 0 1

"194792_fr1" 0 0 0 0 0 0 0 0 0 0 0 0 0 0 0 0 0 0 0 0 0 0 0 0 0 0 0 4 0 0 0 0 0 0 0 0 0 0 0 0 0 0 0 0 0 0 0 0 0 0 0 0 0 0 0 0 0 0 0 0 0 0 0 0 0 0 0 0 0 0 0 0 0 0 0 0 0 0 0 0 0 0 0 0 0

"194796_fr2" 8 4 10 11 1 4 5 7 14 5 3 7 7 4 12 9 8 11 1 6 2 10 8 17 11 3 4 2 6 0 12 8 2 7 14 2 0 2 0 1 0 0 2 0 0 0 0 0 0 0 0 0 0 0 0 0 0 0 0 0 0 0 0 0 0 0 0 0 0 0 0 0 1 0 0 2 2 5 3 0 8 5 6 4 10

"194806_fr6" 0 0 0 0 5 0 0 0 0 0 4 0 0 0 0 0 0 0 0 0 0 0 0 0 0 3 0 3 0 0 0 0 0 0 0 0 0 0 0 0 0 0 0 0 0 0 0 0 0 0 0 0 0 0 0 0 0 0 0 0 0 0 0 0 0 0 0 0 0 0 0 0 0 0 0 0 0 0 0 0 0 0 0 0 0

"195102_fr4" 17 7 12 5 2 9 28 17 6 11 13 18 5 10 6 11 28 7 4 6 0 11 5 4 16 13 12 0 4 14 4 2 6 10 4 5 3 12 0 12 0 0 11 0 0 0 0 0 0 0 0 0 0 0 0 0 0 0 0 0 0 0 0 0 0 0 0 0 0 0 0 0 4 0 0 14 15 18 10 13 21 11 16 14 21

"195111_fr3" 0 0 0 0 0 0 0 0 0 0 0 0 0 0 0 0 0 0 0 0 1 0 0 0 0 1 0 0 0 3 0 0 0 0 0 0 0 0 0 0 0 0 0 0 0 0 0 0 0 0 0 0 0 0 0 0 0 0 0 0 0 0 0 0 0 0 0 0 0 0 0 0 0 0 0 0 0 0 0 0 0 0 0 0 0

"195190_fr6" 4 0 0 0 0 0 0 0 0 0 0 0 0 0 0 0 0 0 0 0 0 0 0 0 0 0 0 0 0 0 0 0 0 0 0 0 0 0 0 0 0 0 0 0 0 0 0 0 0 0 0 0 0 0 0 0 0 0 0 0 0 0 0 0 0 0 0 0 0 0 0 0 0 0 0 0 0 0 0 0 0 0 0 0 0

"195405_fr4" 0 0 0 0 1 2 0 0 0 0 2 2 0 0 0 2 2 0 2 2 4 0 2 0 0 2 2 0 2 0 2 2 2 4 0 0 0 0 0 0 0 0 0 0 0 0 0 0 0 0 0 0 0 0 0 0 0 0 0 0 0 0 0 0 0 0 0 0 0 0 0 0 0 0 0 0 0 0 0 0 0 0 0 0 0

"195483_fr1" 3 4 4 0 4 5 7 5 3 7 4 10 8 4 9 10 8 8 2 8 3 11 3 0 4 4 0 0 4 3 0 0 4 9 3 0 0 3 0 4 0 0 0 0 0 0 0 0 0 0 0 0 0 0 0 0 0 0 0 0 0 0 0 0 0 0 0 0 0 0 0 0 0 0 0 0 3 6 2 0 0 1 8 2 5

"195622_fr2" 6 7 8 7 5 6 6 3 7 9 5 12 9 9 5 9 7 6 7 6 7 6 4 5 7 9 7 8 8 5 1 7 7 8 9 2 1 4 0 4 0 0 0 0 0 0 0 0 1 0 0 0 0 0 0 0 0 0 0 0 0 0 0 0 0 0 0 0 0 0 0 0 1 2 2 0 3 2 5 3 0 0 5 1 0

"195667_fr3" 4 4 4 3 0 4 3 4 4 0 3 2 4 4 0 0 3 4 2 0 2 4 0 4 0 1 2 0 2 3 4 4 2 2 0 0 0 0 0 0 0 0 0 0 0 0 0 0 0 0 0 0 0 0 0 0 0 0 0 0 0 0 0 0 0 3 0 0 0 0 0 3 0 0 0 3 0 0 0 0 3 0 0 5 0

"195874_fr1" 0 0 0 0 0 0 0 0 0 0 0 0 0 0 0 0 0 0 0 0 0 0 0 0 0 0 0 0 0 0 0 0 0 0 0 0 0 0 3 0 0 0 0 0 0 0 1 0 0 0 0 0 0 0 0 0 0 0 0 0 0 0 0 0 0 0 0 0 0 0 0 0 0 0 0 0 0 0 0 0 0 0 0 0 0

"196124_fr3" 0 0 0 0 0 0 0 0 0 0 0 0 0 0 0 0 0 0 0 0 0 0 0 0 0 0 0 0 0 0 0 0 0 0 0 0 0 2 10 0 0 6 11 8 12 7 11 8 8 8 9 10 7 8 10 7 6 7 9 7 6 5 6 7 8 0 0 0 0 5 0 0 0 0 0 0 0 1 0 0 0 0 0 0 0

"196145_fr5" 0 0 0 0 0 0 0 0 0 0 0 0 0 0 0 0 0 0 0 0 0 0 0 0 0 0 0 4 0 0 0 0 0 0 0 0 0 0 0 0 0 0 0 0 0 0 0 0 0 0 0 0 0 0 0 0 0 0 0 0 0 0 0 0 0 0 0 0 0 0 0 0 0 0 0 0 0 0 0 0 0 0 0 0 0

"19615_fr1" 0 0 0 0 0 0 0 0 0 0 0 0 0 0 0 0 0 0 0 0 0 0 0 0 0 0 0 0 0 0 0 0 0 0 0 0 0 0 4 0 0 0 0 3 4 3 4 0 4 4 3 4 0 4 4 4 4 4 3 4 0 0 0 0 0 0 0 0 0 0 0 0 0 0 0 0 0 0 0 0 0 0 0 0 0

"196397_fr2" 0 0 0 2 2 0 0 0 0 0 0 0 0 10 0 0 0 0 0 0 0 0 0 0 2 0 2 0 0 0 0 0 0 0 2 0 0 0 0 0 5 0 6 0 5 0 1 4 1 12 1 5 0 2 4 0 0 1 0 2 0 0 4 4 0 3 2 2 0 3 0 0 0 0 0 0 0 0 0 0 0 0 0 0 0

"196580_fr2" 2 0 1 2 1 0 0 0 1 0 0 0 0 1 0 0 0 1 2 2 0 1 0 0 0 1 0 0 1 0 2 0 1 4 0 0 0 0 0 0 0 0 0 0 0 0 0 0 0 0 0 0 0 0 0 0 0 0 0 0 0 0 0 0 0 0 0 0 0 0 0 0 0 0 0 0 0 0 0 0 0 0 0 0 0

"1966_fr2" 2 0 3 0 2 0 2 0 0 0 2 0 0 0 0 2 0 0 1 2 0 5 2 7 0 2 3 1 2 4 0 4 0 2 0 0 0 0 0 0 0 0 0 0 0 0 0 0 0 0 0 0 0 0 0 0 0 0 0 0 0 0 0 0 0 0 0 0 0 0 0 0 0 0 0 0 0 0 0 0 0 0 0 0 0

"196615_fr5" 3 1 1 0 0 0 4 0 0 0 0 0 0 0 0 2 0 0 2 0 0 2 0 5 2 5 2 0 0 0 0 0 1 1 0 0 0 0 0 0 0 0 0 0 0 0 0 0 0 0 0 0 0 0 0 0 0 0 0 0 0 0 0 0 0 0 0 0 0 0 0 0 0 0 0 0 0 0 0 0 0 0 0 0 0

"19666_fr3" 0 0 0 0 0 0 0 0 0 0 0 0 0 0 0 0 0 0 0 0 0 0 0 0 0 0 0 0 0 0 0 0 0 0 0 0 0 0 0 0 0 0 0 0 0 0 0 0 0 0 0 0 0 0 0 0 0 0 0 0 0 0 0 0 0 0 5 5 0 0 0 6 0 4 0 0 8 2 5 1 0 0 3 0 0

"196745_fr1" 6 5 7 0 0 6 0 0 0 0 0 8 4 0 11 0 8 0 2 7 6 5 8 7 11 0 0 0 5 0 0 5 0 5 11 3 0 4 0 0 0 0 0 0 0 0 0 0 0 0 0 0 0 0 0 0 2 0 0 0 0 0 0 0 0 0 0 0 0 0 0 0 2 0 0 0 0 5 6 5 9 13 8 9 13

"196843_fr3" 0 0 0 0 5 2 8 2 3 2 5 0 2 0 0 2 3 2 10 2 2 2 12 0 0 2 2 20 11 13 0 0 2 2 2 0 0 0 0 0 0 0 0 0 0 0 0 0 0 0 0 0 0 0 0 0 0 0 0 0 0 0 0 0 0 0 0 0 0 0 0 0 0 0 0 5 2 15 19 5 7 9 9 10 2

"196858_fr4" 3 0 0 4 15 0 7 5 0 7 0 0 6 0 0 0 0 6 0 0 0 0 5 6 6 0 0 4 5 12 0 1 5 0 4 0 0 0 0 0 0 0 0 0 0 0 0 0 4 2 0 1 0 0 2 0 2 0 0 0 0 0 0 0 0 3 5 0 0 0 0 0 0 0 0 0 0 0 0 1 0 0 2 3 0

"197005_fr6" 0 1 1 0 0 0 0 2 5 0 1 0 2 0 0 0 0 6 0 0 0 0 0 3 1 0 0 0 0 0 0 0 1 0 1 3 1 0 0 0 0 0 0 0 0 0 0 0 0 0 0 0 0 0 0 0 0 0 0 0 0 0 0 0 0 0 0 0 0 0 0 0 0 0 0 0 0 0 0 3 0 0 0 0 6

"197099_fr1" 9 0 6 11 0 1 0 3 5 0 9 0 0 1 10 2 2 2 2 10 1 2 0 0 10 1 2 0 0 4 3 7 0 1 0 0 1 0 0 4 0 0 0 0 0 0 0 0 0 0 0 0 0 0 0 0 0 2 0 0 0 0 0 0 0 8 2 7 0 0 0 0 0 1 0 0 0 0 2 3 5 1 8 6 7

"197161_fr2" 0 0 0 0 5 2 2 3 0 0 5 4 1 4 0 3 4 0 4 3 2 2 4 0 0 4 4 2 5 0 2 2 4 2 2 0 0 0 0 0 0 0 0 0 0 0 0 0 0 0 0 0 0 0 0 0 0 0 0 0 0 0 0 0 0 0 0 0 0 0 1 2 0 1 1 2 5 2 2 0 2 2 0 1 0

"197192_fr5" 0 0 0 0 0 0 0 0 0 0 0 0 1 0 0 0 0 0 0 0 0 0 2 1 0 0 0 6 0 2 0 0 2 0 0 0 0 0 0 0 0 0 0 0 0 0 0 0 0 0 0 0 0 0 0 0 0 0 0 0 0 0 0 0 0 0 0 0 0 0 0 0 0 0 0 0 0 0 0 0 0 0 0 0 0

"1975_fr6" 0 2 1 0 3 0 3 0 0 0 0 0 0 0 0 1 1 0 0 1 0 0 2 2 1 1 1 0 0 0 0 2 1 3 0 0 0 0 0 0 0 0 0 0 0 0 0 0 0 0 0 0 0 0 0 0 0 0 0 0 0 0 0 0 0 0 0 0 0 0 0 0 0 0 0 0 0 0 0 0 0 0 0 0 0

"197521_fr3" 0 0 0 0 0 0 0 0 0 0 0 0 0 0 0 0 0 0 0 0 0 0 0 0 0 0 0 0 0 0 0 0 0 0 0 0 0 0 0 0 0 0 0 0 0 0 0 0 0 0 0 0 0 0 0 0 0 0 0 0 0 0 0 4 0 0 0 0 0 0 2 0 0 0 0 0 0 0 0 0 0 1 0 0 0

"197578_fr3" 0 0 0 0 0 0 0 0 0 0 0 0 0 0 0 0 0 0 0 0 0 0 0 0 0 0 0 0 0 0 0 0 0 0 0 0 0 0 0 0 0 0 0 0 0 0 0 0 0 0 0 0 0 0 0 0 0 0 0 0 1 0 0 0 0 0 0 0 0 1 0 0 0 0 1 0 0 0 0 0 0 0 0 0 0

"197640_fr5" 0 3 0 0 0 0 0 0 0 2 3 0 0 0 0 0 0 0 0 0 0 0 0 0 0 0 0 0 0 0 0 0 0 0 0 0 0 0 0 0 0 0 0 0 0 0 0 0 0 0 0 0 0 0 0 0 0 0 0 0 0 0 0 0 0 0 0 0 0 0 0 0 0 0 0 0 0 0 0 0 0 2 0 0 0

"19775_fr1" 4 1 3 3 0 2 2 2 0 0 0 0 0 0 1 0 0 0 2 2 0 2 0 0 0 1 0 0 2 0 0 1 0 0 0 0 0 0 0 0 0 0 0 0 0 0 0 0 0 0 0 0 0 0 0 0 0 0 0 0 0 0 0 0 0 0 1 0 0 0 3 1 0 1 1 4 1 2 3 0 0 0 2 0 1

"197797_fr1" 0 0 0 0 0 0 1 0 0 2 0 0 2 0 0 0 0 2 0 0 1 0 0 0 0 0 1 0 1 0 0 0 0 2 0 0 0 0 0 0 0 0 0 0 0 0 0 0 0 0 0 0 0 0 0 0 0 0 0 0 0 1 0 0 0 1 0 2 0 0 0 0 0 0 0 0 0 0 0 3 0 0 0 0 0

"197838_fr2" 0 0 0 0 0 0 0 0 0 0 0 0 2 0 0 0 0 2 0 0 0 0 0 0 0 0 0 0 0 0 0 0 0 0 0 0 0 0 0 0 0 0 0 0 0 0 0 0 0 0 0 0 0 0 0 0 0 0 0 0 0 0 0 0 0 0 0 0 0 0 0 0 0 0 0 0 0 0 0 0 0 0 0 0 0

"198177_fr2" 0 0 0 0 0 0 0 0 0 0 0 0 0 0 0 0 0 0 0 0 0 0 0 0 0 0 0 0 0 0 0 0 0 0 0 0 0 2 2 0 1 0 1 2 0 2 0 2 3 2 0 0 1 3 0 4 0 3 4 4 0 0 0 0 0 0 0 0 0 0 0 0 0 0 0 0 0 0 0 0 0 0 0 0 0

"198233_fr5" 0 0 0 0 2 0 0 2 0 0 1 0 0 0 0 0 0 0 0 0 0 0 0 0 0 0 0 8 0 0 0 0 0 0 0 0 0 0 0 0 0 0 0 0 0 0 0 0 0 0 0 0 0 0 0 0 0 0 0 0 0 0 0 0 0 0 0 0 0 0 0 0 0 0 0 0 0 0 0 0 0 0 0 0 0

"198352_fr6" 0 0 0 0 0 0 0 0 0 0 0 0 0 0 0 0 0 0 0 0 0 0 0 0 0 0 0 0 0 0 0 0 0 0 0 0 0 0 0 0 0 0 0 0 0 0 0 0 0 0 0 0 0 0 0 0 0 0 0 0 0 0 0 0 0 0 0 0 0 0 0 3 2 0 0 0 0 3 3 0 0 0 0 0 0

"198421_fr3" 2 14 11 2 6 7 18 12 8 12 7 0 24 10 8 11 0 15 11 6 12 6 6 2 2 10 6 19 13 0 6 2 13 8 13 0 0 4 0 3 0 0 0 0 5 0 1 0 0 6 0 0 0 1 0 0 0 0 0 0 0 0 0 0 0 0 0 5 0 0 0 0 0 0 0 0 0 0 0 0 7 0 2 1 5

"198446_fr5" 2 2 2 2 6 2 0 2 5 2 2 3 4 5 0 0 5 0 1 5 5 0 2 2 0 6 2 0 2 0 0 2 5 2 0 0 0 0 0 0 0 0 0 0 0 0 0 0 0 0 0 0 0 0 0 0 0 0 0 0 0 0 0 0 0 0 0 0 0 0 0 0 0 0 0 0 2 1 4 0 0 0 0 0 0

"198506_fr4" 0 0 0 0 0 0 0 0 0 0 0 0 0 0 0 0 0 0 0 0 0 0 0 0 0 0 0 0 0 0 0 0 0 0 0 0 0 0 0 0 0 0 0 0 0 0 0 0 0 0 0 0 0 0 0 0 0 0 0 0 0 0 0 0 0 0 0 0 0 0 0 0 0 0 0 1 0 2 3 0 0 0 0 0 0

"198854_fr5" 3 0 3 4 0 4 0 0 0 1 3 0 0 2 0 0 0 0 4 0 4 0 4 0 0 3 8 0 7 2 4 7 2 2 0 0 0 0 0 3 0 0 0 0 0 2 0 3 0 0 0 0 0 0 0 2 3 4 0 0 0 0 0 0 0 0 0 1 3 1 2 1 1 1 4 0 0 2 2 2 0 1 2 0 0

"198862_fr2" 0 0 0 0 0 0 0 0 0 0 0 0 0 0 0 0 0 0 0 0 0 0 0 0 0 0 0 0 0 0 0 0 0 0 0 3 2 0 0 0 0 0 0 0 0 0 0 0 0 0 0 0 0 0 0 0 0 0 0 0 0 0 0 0 0 0 0 0 5 0 0 0 0 0 0 0 0 0 0 0 0 0 0 0 2

"198995_fr4" 2 5 0 0 2 0 6 1 1 3 0 0 6 0 2 0 0 6 2 0 1 0 0 0 0 0 0 0 2 2 0 4 0 1 2 0 0 0 0 0 0 0 0 0 0 0 0 0 0 0 0 0 0 0 0 0 0 0 0 0 0 0 0 0 0 0 0 0 0 0 0 0 0 0 0 0 0 0 0 0 0 0 0 0 0

"199312_fr3" 0 0 0 0 0 0 0 0 0 0 0 0 0 0 0 0 0 0 0 0 0 0 0 0 0 0 0 0 0 0 0 0 0 0 0 0 0 0 0 0 0 0 0 0 0 0 0 0 0 0 0 0 0 0 0 0 0 0 0 0 0 0 0 0 0 0 0 0 0 0 0 0 0 0 2 0 0 0 0 0 0 0 0 0 0

"199470_fr6" 0 0 0 0 0 0 0 6 0 0 0 0 0 0 1 0 0 0 0 0 0 0 0 0 0 0 0 0 0 0 0 0 0 0 0 0 0 0 0 0 0 0 0 0 0 0 0 0 0 0 0 0 0 0 0 0 0 0 0 0 0 0 0 0 0 0 0 0 0 0 0 0 0 0 0 0 0 0 0 0 0 0 0 0 0

"199575_fr4" 0 0 0 0 0 0 0 0 0 0 0 0 0 0 0 0 0 0 0 0 0 0 0 0 0 0 0 0 0 0 0 0 0 0 0 0 0 2 0 0 1 1 5 4 0 0 0 0 0 0 0 0 0 0 0 0 0 0 0 0 0 0 0 0 0 0 0 0 0 0 0 0 0 0 0 0 0 0 0 0 0 0 0 0 0

"199594_fr2" 0 0 2 0 2 0 3 1 0 0 0 0 0 0 0 0 0 0 0 0 0 0 2 0 0 0 0 0 0 0 0 2 2 0 0 0 0 0 0 0 0 0 0 0 0 0 0 0 0 0 0 0 0 0 0 0 0 0 0 0 0 0 0 0 0 0 0 0 0 0 0 0 0 0 0 0 0 0 0 0 0 0 0 0 0

"199608_fr3" 0 0 0 0 0 0 0 0 0 0 0 0 0 0 0 0 0 0 0 0 0 0 0 0 2 0 0 0 0 0 0 0 0 0 0 0 0 0 0 0 0 0 0 0 0 0 0 0 0 0 0 0 0 0 0 0 0 0 0 0 0 0 0 0 0 0 0 0 0 0 0 0 0 0 0 0 0 0 0 0 0 0 0 0 0

"199807_fr1" 3 2 6 3 5 1 1 2 2 9 2 1 3 1 6 4 6 4 2 5 1 6 4 2 3 1 0 0 2 0 4 4 0 2 2 0 0 0 0 0 0 0 0 0 0 0 0 0 0 0 0 0 0 0 0 0 0 0 0 0 0 0 0 0 0 0 0 0 0 0 0 0 0 0 0 0 0 0 0 0 0 0 0 0 0

"199971_fr3" 0 0 0 0 0 0 0 0 0 0 0 0 0 0 0 0 0 0 0 0 0 0 0 0 0 0 0 0 0 0 0 0 0 0 0 1 7 21 52 18 40 34 42 62 58 67 60 63 45 62 52 60 58 74 66 61 60 61 52 67 55 63 51 47 57 33 43 31 28 45 0 2 0 0 0 0 0 0 0 0 0 0 0 0 0

"200124_fr5" 0 0 0 0 0 0 0 0 0 0 0 0 0 0 0 0 0 0 0 0 0 0 0 0 0 0 0 0 0 0 0 0 0 0 0 0 0 0 0 0 0 0 0 0 0 0 0 0 0 0 0 0 0 0 0 0 3 0 0 0 0 0 0 0 0 0 0 0 0 0 0 0 0 0 0 0 0 0 0 0 0 0 0 0 0

"200269_fr2" 2 2 1 0 2 0 2 0 2 0 1 0 0 2 10 1 2 0 1 2 2 1 2 1 0 1 0 0 0 0 0 2 0 0 0 0 0 0 0 0 0 0 0 0 0 0 0 0 0 0 0 0 0 0 0 0 0 0 0 0 0 0 0 0 0 7 6 2 8 0 0 0 7 3 8 0 0 0 0 0 0 0 0 0 0

"200301_fr2" 3 2 2 2 10 2 3 2 2 4 2 4 4 2 2 4 3 5 2 2 0 2 9 9 4 0 0 8 3 0 5 0 2 2 2 0 0 0 0 0 0 0 8 0 0 0 0 0 0 0 0 0 0 0 4 0 0 0 0 0 0 0 0 0 0 0 0 0 0 0 0 0 0 0 0 0 0 0 0 0 0 0 0 0 0

"20034_fr2" 0 0 0 0 0 0 0 0 0 0 0 0 0 0 0 0 0 0 0 0 0 0 0 0 0 0 0 0 0 0 0 0 0 0 0 1 5 7 9 7 1 8 8 10 11 8 11 8 9 9 8 7 9 12 10 8 1 9 9 6 6 10 7 5 8 8 5 6 4 8 2 1 1 0 1 1 2 0 1 1 0 0 0 0 3

"200426_fr2" 0 0 0 0 0 0 0 0 0 0 0 0 0 0 0 0 0 0 0 0 0 0 0 0 0 0 0 0 0 0 0 0 0 0 0 3 0 16 37 15 15 12 22 22 27 10 30 25 11 25 32 38 27 34 37 23 31 32 38 36 25 35 34 25 26 0 3 2 1 11 0 0 0 0 0 0 0 0 0 0 0 0 0 0 0

"200559_fr3" 0 0 0 0 0 0 0 0 0 0 0 0 0 0 0 0 0 0 0 0 0 0 0 0 0 0 0 0 0 0 0 0 0 0 0 0 0 0 3 0 0 1 1 0 4 0 1 0 5 2 5 0 0 0 3 2 2 2 2 0 0 0 0 0 0 0 0 0 0 0 0 0 0 0 0 0 0 0 0 0 0 0 0 0 0

"200648_fr2" 0 0 0 0 0 0 0 0 0 0 0 0 0 0 0 0 0 0 0 0 0 0 0 0 0 0 0 0 0 0 0 0 0 0 0 0 0 0 0 0 0 0 0 0 0 0 0 0 0 0 0 0 0 0 0 0 0 0 0 0 0 0 0 0 0 5 7 0 0 0 0 0 0 0 0 0 0 0 0 0 0 0 0 0 0

"200701_fr2" 0 2 4 14 0 5 0 3 2 0 0 0 0 0 0 0 0 0 0 0 0 0 0 0 0 0 7 0 5 0 0 0 0 0 0 0 0 0 0 0 0 0 2 0 0 0 0 0 0 0 0 0 0 0 0 0 0 4 0 0 0 0 0 0 0 0 0 0 0 0 0 0 0 0 0 0 0 0 0 0 0 0 0 0 0

"200782_fr1" 2 2 2 2 2 2 2 3 2 3 2 0 2 3 0 2 2 4 7 2 0 3 7 2 3 12 0 1 3 2 4 0 2 2 0 6 1 1 0 0 0 0 0 0 0 0 0 0 0 0 0 0 0 0 0 0 0 0 0 0 0 0 0 0 0 0 0 0 0 0 0 0 0 0 0 0 7 4 0 4 3 3 3 2 3

"201238_fr2" 0 0 0 0 0 0 0 0 0 0 0 1 0 0 0 0 2 0 0 0 0 0 0 0 0 0 0 3 0 0 0 0 0 0 0 0 0 0 0 0 0 0 0 0 0 0 0 0 0 0 0 0 0 0 0 0 0 0 0 0 0 0 0 0 0 0 0 0 0 0 0 0 0 0 0 0 0 0 0 0 0 0 0 0 0

"201362_fr4" 0 0 0 0 0 0 0 0 0 0 0 0 0 0 0 0 0 0 0 0 0 0 0 0 0 0 0 0 0 0 0 0 0 0 0 0 3 2 4 2 2 4 1 5 3 2 4 2 4 4 2 2 3 4 2 4 2 2 7 4 0 0 1 0 0 0 0 0 2 0 0 0 0 0 0 0 0 0 0 0 0 0 0 0 0

"201379_fr2" 0 0 0 0 0 0 0 0 0 0 0 6 0 0 2 0 0 0 0 0 0 0 0 0 0 0 0 0 0 0 0 0 0 0 3 0 0 0 0 0 0 0 0 0 0 0 0 0 0 0 0 0 0 0 0 0 0 0 0 0 0 0 0 0 0 0 0 0 0 0 0 0 0 0 0 6 2 0 4 2 0 0 0 0 0

"201400_fr3" 0 0 0 0 0 0 0 0 0 5 0 0 2 0 0 0 0 0 0 0 0 0 0 0 0 0 0 0 0 0 0 0 0 0 0 0 0 0 0 0 0 0 0 0 0 0 0 0 0 0 0 0 0 0 0 0 0 0 0 0 0 0 0 0 0 0 0 0 0 0 0 0 0 0 0 0 0 0 0 0 0 0 0 0 0

"201522_fr3" 0 0 0 0 0 0 0 0 0 0 0 0 0 0 0 0 0 0 0 0 0 0 0 0 0 0 0 0 0 0 0 0 0 0 0 0 0 8 8 0 0 11 8 6 8 11 10 9 8 8 2 12 10 10 10 12 9 12 9 12 13 9 8 8 8 0 0 0 3 4 0 0 0 0 0 0 0 0 0 0 0 0 0 0 0

"201527_fr6" 0 0 0 0 0 0 0 0 0 0 0 0 0 0 0 0 0 0 0 0 0 0 0 0 0 0 0 0 0 0 0 0 0 0 0 0 0 0 0 0 0 0 0 0 0 0 0 0 0 0 0 0 0 0 0 0 0 0 0 0 0 0 0 0 0 0 0 0 0 3 0 0 0 0 0 0 0 0 0 0 0 0 0 0 0

"201587_fr3" 0 0 0 0 0 0 0 0 0 0 0 0 0 0 0 0 0 0 0 0 0 0 0 0 0 0 0 0 0 0 0 0 0 0 0 0 0 3 1 0 5 0 0 5 0 0 5 5 16 0 0 0 0 0 0 0 0 0 0 0 0 0 2 2 3 2 2 0 0 0 0 0 0 0 0 0 0 0 0 0 0 0 0 0 0

"201667_fr3" 0 0 0 0 0 0 0 0 0 0 0 0 0 0 0 0 0 0 0 0 0 0 0 0 0 0 0 0 0 0 0 0 0 0 0 1 1 0 8 8 0 7 7 0 0 10 0 0 10 0 0 0 0 0 12 8 0 0 9 8 0 8 10 8 9 1 3 8 0 0 0 1 0 0 0 0 0 0 0 0 0 0 0 0 0

"201744_fr2" 0 0 0 0 0 0 0 0 0 0 0 0 0 0 0 0 0 0 0 0 0 0 0 0 0 0 0 0 0 0 0 0 0 0 0 0 0 0 2 0 4 2 4 5 2 3 3 4 5 5 5 8 6 7 3 7 9 8 10 6 7 4 5 5 3 6 4 3 2 4 2 2 0 0 1 1 2 0 1 0 0 2 0 0 0

"201834_fr4" 0 0 0 0 0 0 0 0 0 0 0 0 0 0 0 0 0 0 0 0 0 0 0 0 0 0 0 0 0 0 0 0 0 0 0 0 0 0 0 0 0 0 0 0 0 0 0 0 0 0 0 0 0 0 0 0 0 0 0 0 0 0 0 0 0 2 2 2 9 0 4 3 2 4 3 5 6 1 6 5 0 0 0 1 2

"201844_fr3" 0 0 0 0 0 0 0 0 0 0 0 0 0 0 0 0 0 0 0 0 1 0 3 0 0 0 1 0 0 2 0 0 0 0 0 0 0 0 0 0 0 0 0 0 0 0 0 0 0 0 0 0 0 0 0 0 0 0 0 0 0 0 0 0 0 0 0 0 0 0 1 0 0 0 0 0 1 0 0 0 0 0 0 0 0

"201865_fr3" 0 0 0 0 0 0 0 0 0 0 0 0 0 0 0 0 0 0 0 0 0 0 0 0 0 0 0 0 0 0 0 0 0 0 0 0 0 0 0 0 0 0 0 0 1 0 0 1 0 0 2 2 4 0 0 0 4 3 0 0 3 0 0 0 0 0 0 0 0 3 0 0 0 0 0 0 0 0 0 0 0 0 0 0 0

"20187_fr5" 4 0 5 2 3 0 0 0 2 0 0 0 0 3 6 0 5 0 4 5 4 3 0 4 0 5 4 3 4 5 0 6 7 2 4 0 0 0 0 0 0 0 0 0 0 0 0 0 0 0 0 0 0 0 0 0 0 0 0 0 0 0 0 0 0 0 0 0 0 0 0 0 0 0 0 0 0 2 2 0 0 0 3 2 0

"201978_fr3" 0 0 0 0 0 0 0 0 0 0 0 0 0 0 0 0 0 0 0 0 0 0 0 0 0 0 0 0 0 0 0 0 0 0 0 0 0 0 0 0 0 0 0 0 0 0 0 0 0 0 0 0 0 0 0 0 0 0 0 0 0 0 0 0 0 0 0 0 0 0 0 0 0 0 0 0 0 0 0 0 0 5 0 0 1

"202_fr5" 0 0 3 0 0 0 0 0 0 0 0 0 0 0 0 0 0 0 0 0 0 0 0 4 0 0 4 0 1 3 0 0 0 0 0 0 0 0 0 0 0 0 0 0 0 0 0 0 0 0 0 0 0 0 0 0 0 0 0 0 0 0 0 0 0 0 0 0 0 0 0 0 0 0 0 0 0 0 0 0 0 0 0 0 0

"202109_fr3" 0 0 1 0 1 0 0 0 0 0 0 0 0 0 0 0 1 0 0 0 0 0 1 2 2 1 0 0 1 2 2 1 0 0 0 0 0 0 0 0 0 0 0 0 0 0 0 0 0 0 0 0 0 0 0 0 0 0 0 0 0 0 0 0 0 0 0 0 0 0 0 0 0 0 0 0 0 0 0 0 0 0 0 0 0

"202279_fr1" 2 4 3 4 4 3 4 3 4 4 2 2 4 3 4 3 3 4 3 3 4 2 0 4 2 3 1 4 4 0 3 13 3 4 4 0 0 7 0 1 0 5 0 0 5 0 0 0 0 1 0 0 0 0 0 0 0 1 0 0 0 4 2 5 0 3 11 4 2 0 2 7 2 5 7 15 11 3 13 4 3 3 4 4 4

"202300_fr6" 0 0 0 0 0 0 0 0 0 0 0 0 0 0 0 0 0 0 0 0 0 0 0 0 0 0 0 0 0 0 0 0 0 0 0 0 0 0 0 0 0 0 0 0 0 0 0 0 0 3 0 0 0 0 0 0 0 0 0 0 0 0 0 0 0 0 0 0 0 0 0 0 0 0 0 0 0 0 0 0 0 0 0 0 0

"202609_fr5" 0 0 0 0 0 0 0 0 0 0 0 0 1 0 0 0 0 3 0 0 0 0 0 0 0 0 0 0 0 0 0 0 0 0 0 0 0 0 0 0 0 0 0 0 0 0 0 0 0 0 0 0 0 0 0 0 0 0 0 0 0 0 0 0 0 0 0 0 0 0 0 0 0 0 0 0 0 0 0 0 0 0 0 0 0

"202637_fr1" 8 7 7 6 12 6 11 9 11 11 10 7 11 0 10 9 7 10 6 7 8 8 9 9 6 7 7 18 15 17 5 7 9 8 8 0 0 0 0 0 0 0 0 0 0 0 0 0 0 0 0 0 0 0 0 0 0 0 0 0 0 0 0 0 0 0 0 0 0 0 0 0 0 0 0 0 0 0 0 0 0 0 0 0 0

"202637_fr3" 0 0 0 0 0 0 0 0 0 0 0 0 0 0 0 0 0 0 0 0 0 0 0 0 0 0 0 0 0 0 0 0 0 0 0 0 3 0 0 5 1 0 2 0 0 0 0 0 0 0 0 0 0 0 0 0 1 0 0 2 0 2 0 0 0 5 0 0 0 0 0 0 0 0 0 3 0 0 0 2 5 2 2 0 2

"202705_fr3" 0 0 0 0 0 0 0 0 0 0 0 0 10 0 0 0 0 0 0 0 0 0 0 0 1 0 0 0 0 0 0 0 0 0 0 0 0 0 0 0 0 0 0 0 0 0 0 0 0 0 0 0 0 0 0 0 0 0 0 0 0 0 0 0 0 0 2 0 0 0 0 0 0 0 1 0 0 0 0 0 0 0 0 0 0

"2029_fr2" 0 0 0 1 0 0 0 0 4 0 0 0 0 0 3 0 0 4 0 0 0 0 0 0 11 0 0 0 1 0 2 0 0 0 4 0 0 0 0 0 0 0 0 0 0 0 0 0 0 0 0 0 0 0 0 0 0 0 0 0 0 0 0 0 0 0 0 0 0 0 0 0 0 0 0 0 0 0 0 0 0 2 1 1 1

"203291_fr2" 0 0 0 0 0 0 0 0 0 0 0 0 0 0 0 0 0 0 0 0 0 0 0 0 0 0 0 0 0 0 0 0 0 0 0 0 0 0 0 0 0 0 0 0 0 0 0 0 0 0 0 0 2 0 0 0 0 0 0 0 0 0 0 0 0 0 0 0 0 0 0 0 0 0 0 0 0 0 0 0 0 0 0 0 0

"203298_fr1" 0 0 0 0 0 0 0 0 0 0 0 0 0 0 0 0 0 0 0 0 0 0 0 0 0 0 0 0 0 0 0 0 0 0 0 0 0 0 0 0 0 0 0 0 0 0 0 0 0 9 3 0 0 0 0 0 0 0 0 0 0 0 0 0 0 0 0 0 0 0 0 0 0 0 0 0 0 0 0 0 0 0 0 0 0

"20335_fr3" 4 8 4 3 5 2 9 4 4 0 5 3 13 2 0 5 0 0 4 4 6 5 7 0 5 0 0 10 0 0 5 0 0 3 4 0 0 0 0 0 0 0 0 0 0 0 0 0 0 0 0 0 0 0 0 0 0 0 0 0 0 0 0 0 0 0 0 0 0 0 0 0 0 0 0 0 0 0 0 0 0 0 0 0 0

"203444_fr6" 0 0 0 0 0 0 0 0 0 0 0 1 0 4 0 0 4 0 4 0 2 0 0 0 0 0 0 0 0 0 0 0 0 0 0 0 0 0 0 0 0 0 0 0 0 0 0 0 0 0 0 0 0 0 0 0 0 0 0 0 0 0 0 0 0 0 0 0 0 0 0 0 0 0 0 0 0 0 0 0 0 0 0 0 0

"203606_fr2" 0 0 0 0 0 0 0 0 0 0 0 0 0 0 0 0 0 0 0 0 0 0 0 0 0 0 0 0 0 0 0 2 0 0 0 0 0 0 0 0 0 0 0 0 0 0 0 0 0 0 0 0 0 0 0 0 0 0 0 0 0 0 0 0 0 0 0 0 0 0 0 0 0 0 0 0 0 0 0 0 0 0 0 0 0

"203833_fr2" 3 0 1 1 8 0 0 0 0 0 2 1 0 3 0 3 5 0 3 2 1 3 5 3 0 3 3 0 3 5 1 7 2 0 1 3 3 2 0 4 0 0 0 0 0 0 0 0 0 0 0 0 0 0 0 0 0 0 0 0 0 0 0 0 0 2 0 0 6 0 10 8 9 9 8 10 10 10 7 10 2 1 2 3 2

"203938_fr3" 0 0 0 0 0 0 0 0 0 0 0 0 0 0 0 0 0 0 0 0 0 0 0 0 0 0 0 0 0 0 0 0 0 0 0 0 0 0 3 0 0 0 0 0 0 0 0 0 3 0 2 1 0 2 0 0 0 0 0 3 0 0 0 0 2 0 0 0 0 0 0 0 0 0 0 0 0 0 0 0 0 0 0 0 0

"204366_fr5" 0 0 0 0 0 0 0 0 0 0 0 0 0 0 0 0 0 0 0 0 0 0 0 0 0 0 0 0 0 0 0 0 0 0 0 0 0 0 0 0 0 0 0 0 0 0 0 0 0 0 0 3 0 0 0 0 0 0 0 0 0 0 0 0 0 0 0 0 0 0 0 0 0 0 0 0 0 0 0 0 0 0 0 0 0

"204380_fr6" 0 0 0 1 0 0 2 2 0 0 0 0 0 7 0 0 0 0 3 0 0 0 0 0 0 3 3 0 4 3 0 2 0 0 0 0 0 0 0 0 0 0 0 0 0 0 0 0 0 0 0 0 0 0 0 0 0 0 0 0 0 0 0 0 0 0 0 0 0 0 0 0 0 0 0 0 0 0 0 0 0 0 0 0 0

"204527_fr2" 2 3 0 0 3 3 4 0 0 0 3 0 0 2 0 3 0 0 3 0 4 0 2 0 0 5 0 0 0 0 0 4 2 0 0 0 0 0 0 0 0 0 0 0 0 0 0 0 0 0 0 0 0 0 0 0 0 0 0 0 0 0 0 0 0 0 0 0 0 0 0 0 0 0 0 0 0 0 0 0 0 0 0 0 0

"204630_fr1" 0 0 0 0 0 0 0 0 0 0 0 0 0 0 0 0 0 0 0 0 0 0 4 0 0 0 0 0 0 0 0 0 0 0 0 0 0 0 0 0 0 0 0 0 0 0 0 0 0 0 0 0 0 0 0 0 0 0 0 0 0 0 0 0 0 0 0 0 0 0 0 0 0 0 0 0 0 0 0 0 0 0 0 0 0

"204711_fr1" 0 0 0 0 0 0 0 0 0 0 0 0 0 0 0 0 0 0 0 0 0 0 0 0 0 0 0 0 0 0 0 0 0 0 0 0 0 1 0 0 0 0 2 2 2 0 2 0 0 2 2 2 0 1 0 0 4 0 0 0 0 0 0 0 0 0 0 0 0 0 0 0 0 0 0 0 0 0 0 0 0 0 0 0 0

"204825_fr6" 0 0 0 0 0 0 0 0 0 0 0 0 0 0 0 0 0 0 0 0 0 0 0 0 0 0 0 0 0 0 0 0 0 0 0 0 2 2 3 1 0 2 2 2 4 4 3 3 4 4 2 4 5 2 3 4 2 2 4 3 4 4 4 6 3 7 6 4 4 7 5 19 20 25 21 18 18 2 16 14 2 9 1 11 2

"204886_fr1" 0 1 0 3 0 0 0 1 0 1 0 0 0 0 0 0 0 0 0 0 2 0 0 0 0 0 0 0 0 0 0 0 0 0 0 0 0 0 0 0 0 0 0 0 0 0 0 0 0 0 0 0 0 0 0 0 0 0 0 0 0 0 0 0 0 0 0 0 0 0 0 0 0 0 0 0 0 0 0 0 0 0 0 0 0

"204994_fr3" 0 0 0 0 0 0 0 0 0 0 0 0 0 0 0 0 0 0 0 0 0 0 0 0 0 0 0 0 0 0 0 0 0 0 0 0 0 0 0 0 0 0 0 0 0 0 0 0 0 0 0 0 1 0 0 5 0 0 0 0 0 0 1 0 4 0 0 0 0 0 0 0 0 0 0 0 0 0 0 0 0 0 0 0 0

"205010_fr4" 4 3 0 0 0 5 0 0 3 2 4 3 0 0 0 0 2 0 0 5 4 5 4 0 0 4 5 3 5 0 0 4 0 0 1 0 0 0 0 0 0 0 0 0 0 0 0 0 0 0 0 0 0 0 0 0 0 0 0 0 0 0 0 0 0 5 0 0 3 0 3 0 7 0 0 0 0 0 6 3 0 3 6 0 0

"205033_fr1" 0 0 0 0 0 0 0 0 0 0 0 0 0 0 0 0 0 0 0 0 0 0 0 0 0 0 0 0 0 0 0 0 0 0 0 0 0 0 0 0 0 0 0 0 0 0 0 0 0 0 0 0 0 0 0 0 0 0 0 0 0 6 0 0 1 3 0 6 0 3 2 4 2 2 3 4 2 4 2 2 3 5 2 2 5

"205033_fr3" 0 0 0 0 0 0 0 0 0 0 0 0 0 0 0 0 0 0 0 0 0 0 0 0 0 0 0 0 0 0 0 0 0 0 0 0 3 3 9 12 13 9 9 3 11 0 12 4 10 12 15 24 0 0 9 3 11 6 11 9 0 0 0 0 0 0 0 0 0 0 0 0 0 0 0 0 0 0 0 0 0 0 0 0 0

"205082_fr4" 0 4 10 16 0 7 1 4 12 0 0 0 0 0 0 0 2 0 0 0 4 2 0 0 10 1 10 3 5 2 10 1 0 4 4 0 0 0 0 0 0 0 0 0 0 0 0 0 0 0 0 0 0 0 0 0 0 0 0 0 0 0 0 0 0 0 0 0 0 0 0 0 0 0 0 0 0 0 0 0 0 0 0 0 0

"205133_fr6" 0 0 0 0 0 0 0 0 0 0 0 0 0 0 0 0 0 0 0 0 0 0 0 0 0 0 0 0 0 0 0 0 0 0 0 0 0 0 0 0 0 0 0 0 0 0 0 0 0 0 0 0 0 0 0 0 0 0 0 0 2 2 2 0 0 0 0 0 0 0 0 0 0 6 2 1 7 0 0 4 0 0 0 4 0

"205368_fr4" 0 0 0 0 0 0 0 0 0 0 0 0 0 0 0 0 0 0 0 0 0 0 0 0 0 0 0 0 0 0 0 0 0 0 0 0 0 0 0 0 0 0 0 0 0 0 0 0 0 0 0 0 0 0 0 0 0 0 0 0 0 6 0 0 0 0 0 0 0 0 0 3 0 1 2 0 0 0 0 0 0 1 0 0 0

"205425_fr5" 0 0 0 0 0 0 0 0 0 0 0 0 0 0 0 0 0 0 0 0 0 0 0 0 0 0 0 0 0 0 0 0 0 0 0 0 0 0 0 0 2 0 5 0 0 0 0 0 3 13 0 0 0 0 5 0 0 0 0 0 0 0 0 0 0 0 0 0 0 0 0 0 0 0 0 0 0 0 0 0 0 0 0 0 0

"205588_fr2" 0 0 0 0 0 0 0 2 0 0 0 0 0 0 0 0 0 0 0 2 0 0 0 0 0 0 1 0 1 0 0 0 0 0 0 0 0 0 0 0 0 0 0 0 0 0 0 0 0 0 0 0 0 0 0 0 0 0 0 0 0 0 0 0 0 0 0 0 0 0 0 0 0 0 0 0 0 0 0 0 0 0 0 0 0

"205707_fr3" 0 0 0 0 0 0 0 0 0 0 0 0 0 0 0 0 0 0 0 0 0 0 0 0 0 0 0 0 4 0 0 0 0 0 0 0 0 0 0 0 0 0 0 0 0 0 0 0 0 0 0 0 0 0 0 0 0 0 0 0 0 0 0 0 0 0 0 0 0 0 0 0 0 0 0 0 0 0 0 0 0 0 0 0 0

"205875_fr1" 2 1 1 0 3 0 4 0 0 0 0 0 0 6 2 2 6 0 4 1 0 4 4 2 0 2 3 0 1 7 0 5 6 2 0 0 0 0 0 0 0 0 0 0 0 0 0 0 0 0 0 0 0 0 0 0 0 0 0 0 0 0 0 0 0 0 0 0 0 0 0 0 0 0 0 0 0 0 0 0 0 0 0 0 0

"20596_fr2" 0 0 0 0 0 0 0 0 0 0 0 0 0 0 0 0 0 0 0 0 0 0 0 0 0 0 0 0 0 0 0 0 0 0 0 0 0 0 0 0 0 0 0 0 0 0 0 0 0 0 0 0 0 0 0 0 0 0 0 0 0 0 0 0 0 2 0 0 0 0 0 0 0 0 0 0 0 0 0 1 0 0 0 0 0

"205967_fr4" 0 0 0 0 0 0 0 0 0 0 0 0 0 0 0 0 0 0 0 0 0 0 0 0 0 0 0 0 0 0 0 0 0 0 0 0 0 0 0 0 0 0 0 0 0 0 0 2 2 0 0 0 0 0 5 1 0 2 2 0 0 0 0 0 0 0 0 0 0 0 0 0 0 0 0 0 0 0 0 0 0 0 0 0 0

"205972_fr6" 0 0 0 0 0 0 0 0 0 0 0 0 0 0 0 0 0 0 0 0 0 0 0 0 0 0 0 0 0 0 0 0 0 0 0 0 0 0 0 0 0 0 0 0 1 0 0 0 0 0 0 0 0 7 0 0 0 0 0 0 0 0 0 0 0 0 0 0 0 0 0 0 0 0 0 0 0 0 0 0 0 0 0 0 0

"20628_fr1" 0 0 0 0 0 0 0 0 0 0 0 0 0 0 0 0 0 0 0 0 0 0 0 0 0 0 0 0 0 0 0 0 0 0 0 0 0 0 0 0 0 0 1 0 9 1 10 4 0 6 13 12 6 5 13 6 11 2 2 5 9 0 1 0 2 3 0 2 0 9 0 0 0 0 0 0 0 0 0 0 0 0 0 0 0

"206392_fr5" 0 0 0 0 0 2 0 0 0 0 0 0 0 0 0 0 0 0 0 0 0 0 0 0 0 0 2 0 0 0 0 0 0 0 0 0 0 0 0 0 0 0 0 0 0 0 0 0 0 0 0 0 0 0 0 0 0 0 0 0 0 0 0 0 0 0 0 0 0 0 0 0 0 0 0 0 0 0 0 0 0 0 0 0 0

"206469_fr3" 0 0 0 0 0 0 0 0 0 0 0 0 0 0 0 0 0 0 0 0 0 0 0 0 0 0 0 0 0 0 0 0 0 0 0 7 11 11 48 25 26 9 50 25 54 49 51 22 49 26 52 61 43 62 59 56 53 51 60 63 36 39 48 49 53 53 46 46 48 52 39 43 46 40 46 32 36 33 32 33 16 21 4 22 21

"206496_fr4" 0 0 0 0 0 0 0 0 0 0 0 0 0 0 0 0 0 0 0 0 0 0 0 0 0 0 0 0 0 0 0 0 0 0 0 0 0 0 0 0 0 0 0 0 0 0 0 0 0 0 0 0 0 0 0 0 0 0 0 0 0 8 0 0 0 0 0 6 0 8 0 0 0 0 2 0 0 2 2 0 2 0 2 2 0

"206518_fr4" 0 0 0 0 0 0 0 0 0 0 0 0 0 0 0 0 0 0 0 0 0 0 0 0 0 0 0 0 0 0 0 0 0 0 0 0 0 0 3 0 0 0 1 0 0 1 0 1 0 0 0 0 0 0 0 3 0 0 0 0 0 0 0 0 0 0 0 0 0 0 0 0 0 0 0 0 0 0 0 0 0 0 0 0 0

"206576_fr4" 0 0 0 0 0 0 0 0 0 0 0 0 0 0 0 0 0 0 0 0 0 0 0 0 0 0 0 0 0 0 0 0 0 0 0 0 0 0 0 0 0 0 0 0 0 0 0 0 0 0 0 0 0 0 0 0 0 0 0 0 6 4 4 6 6 9 9 4 5 4 0 4 0 2 0 0 2 0 0 0 0 2 0 0 2

"206684_fr4" 0 0 0 0 0 0 0 0 0 0 0 0 0 0 0 0 0 0 0 0 0 0 0 0 0 0 0 0 0 0 0 0 0 0 0 0 0 0 0 0 0 0 0 0 0 0 0 0 0 0 0 0 0 0 0 0 0 0 0 0 0 0 0 0 0 0 0 0 0 0 1 0 0 0 0 0 0 5 0 0 0 0 2 0 0

"206723_fr1" 0 0 0 0 0 0 0 0 0 0 0 0 0 0 0 0 0 0 0 0 0 0 0 0 0 0 0 0 0 0 0 0 0 0 0 5 4 5 19 7 20 8 18 18 25 20 24 21 24 41 26 25 25 59 27 24 28 21 28 38 23 12 16 20 22 22 16 19 22 20 19 21 13 21 26 14 10 5 11 11 6 6 6 9 9

"206753_fr4" 0 0 0 0 0 0 0 0 0 0 0 0 0 0 0 0 0 0 0 0 0 0 0 0 0 0 0 0 0 0 0 0 0 0 0 0 0 3 14 3 11 5 7 14 19 11 12 6 11 14 16 16 9 15 19 16 11 15 18 22 13 13 13 10 14 13 13 13 18 16 0 7 9 7 9 11 9 6 0 0 0 0 0 1 2

"206816_fr3" 0 0 0 0 0 0 0 0 0 0 0 0 0 0 0 0 0 0 0 0 0 0 0 0 0 0 0 0 0 0 0 0 0 0 0 0 0 0 4 0 0 0 0 3 3 4 4 0 2 2 4 0 1 4 0 4 3 3 0 4 3 0 4 2 0 4 2 4 12 3 12 3 3 14 1 1 2 1 8 6 0 0 0 0 0

"206828_fr3" 0 0 0 0 0 0 0 0 0 0 0 0 0 0 0 0 0 0 0 0 0 0 0 0 0 0 0 0 0 0 0 0 0 0 0 0 0 0 0 0 0 0 0 0 0 0 0 0 0 0 0 0 0 0 0 0 2 0 0 0 0 0 0 0 0 0 0 0 0 0 0 0 0 0 0 0 0 0 0 0 0 0 0 0 0

"206895_fr3" 1 2 2 0 3 0 2 2 1 0 1 3 1 3 0 0 3 0 2 3 2 1 2 2 0 3 0 2 2 2 2 2 3 2 0 0 0 0 0 0 0 0 0 0 0 0 0 0 0 0 0 0 0 0 0 0 0 0 0 0 0 0 0 0 0 0 0 0 0 0 0 0 0 0 0 0 0 0 0 0 0 0 0 0 0

"207048_fr3" 0 0 0 0 0 5 0 0 0 0 0 0 0 0 2 0 0 0 0 0 0 0 0 0 0 0 0 0 0 0 0 0 0 0 0 0 0 0 0 0 2 0 0 1 0 0 0 0 0 2 0 3 0 2 0 2 0 0 1 2 0 0 0 0 0 0 0 0 0 0 0 0 0 0 0 0 0 0 0 0 0 0 0 0 0

"207097_fr1" 0 0 0 0 0 0 0 0 0 0 0 0 0 0 0 0 0 0 0 0 0 0 0 0 0 0 0 0 0 0 0 0 0 0 0 3 0 0 0 0 0 0 0 0 0 0 0 0 0 0 0 0 7 0 0 0 0 0 0 0 0 0 0 0 0 0 0 0 0 0 0 0 0 0 0 0 0 0 0 0 0 0 0 0 0

"207130_fr2" 0 0 0 0 0 0 0 0 0 0 0 0 0 0 0 0 0 0 0 0 0 0 0 0 0 0 0 0 0 0 0 0 0 0 0 1 2 0 0 1 0 0 0 0 14 0 0 2 0 8 0 0 0 6 6 0 0 0 4 1 0 0 0 0 0 0 0 0 0 0 0 0 0 0 0 0 0 0 0 0 0 0 0 0 0

"207425_fr2" 0 0 0 0 0 0 0 0 0 0 0 0 0 0 0 0 0 0 0 0 0 0 0 0 0 0 0 0 0 0 0 0 0 0 0 0 0 1 0 2 0 0 0 0 0 0 0 4 0 0 3 0 0 0 0 0 0 0 0 0 0 0 0 0 0 0 0 0 0 0 0 0 0 0 0 0 0 0 0 0 0 0 0 0 0

"207567_fr4" 0 3 4 0 3 0 0 0 0 0 0 0 0 2 0 0 0 0 0 0 2 0 5 0 0 0 0 0 0 0 0 7 0 0 0 0 0 0 0 0 0 0 0 0 0 0 0 0 0 0 0 0 0 0 0 0 0 0 0 0 0 0 0 0 0 0 0 0 0 0 0 0 0 0 0 0 0 3 0 1 0 0 0 1 0

"207844_fr2" 0 0 0 0 0 0 0 0 0 0 0 0 0 0 0 0 0 0 0 0 0 0 0 0 0 0 0 0 0 0 0 0 0 0 0 0 0 1 0 2 0 0 0 0 0 0 0 0 0 0 0 0 0 0 0 2 0 0 0 0 0 0 0 0 0 0 1 1 0 0 2 1 0 0 0 1 5 0 1 0 0 1 0 3 0

"207844_fr3" 2 0 0 0 5 0 0 5 0 0 1 7 0 1 0 0 3 0 3 2 0 1 6 8 0 2 8 2 1 4 0 1 0 2 0 0 0 0 0 0 0 0 0 0 0 0 0 0 0 0 0 0 0 0 0 0 0 0 0 0 0 0 0 0 0 0 0 0 0 0 0 0 0 0 0 0 0 0 0 0 0 0 0 0 0

"207937_fr5" 0 0 0 0 0 1 0 2 0 0 2 0 0 0 0 0 0 0 0 0 0 0 0 0 0 2 1 0 0 0 0 0 0 2 0 0 0 0 0 0 0 0 0 0 0 0 0 0 0 0 0 0 0 0 0 0 0 0 0 0 0 0 0 0 0 0 0 0 0 0 0 0 0 5 4 0 0 2 2 7 0 1 0 0 4

"20801_fr4" 0 0 0 0 0 0 0 0 0 0 0 2 0 0 0 0 0 0 0 0 3 0 0 0 0 1 0 1 0 0 0 0 0 0 0 0 0 0 0 0 0 0 0 0 0 0 0 0 0 0 0 0 0 0 0 0 0 0 0 0 0 0 0 0 0 0 0 0 0 0 0 0 0 0 0 0 0 0 0 0 0 0 0 0 0

"208021_fr3" 0 1 0 3 2 4 6 1 6 2 2 0 6 2 5 0 0 2 6 1 6 0 1 6 4 6 1 8 6 6 4 6 6 1 0 1 0 6 0 1 2 0 3 3 3 3 4 3 3 4 3 0 0 2 0 0 4 0 0 0 0 0 0 0 0 2 5 5 3 2 1 6 5 2 1 1 2 2 7 4 0 1 1 2 4

"208036_fr5" 10 2 0 0 9 0 0 0 0 0 7 0 0 7 0 6 8 0 2 7 0 5 9 0 0 0 0 0 1 0 0 0 0 7 0 0 4 0 0 0 0 0 0 1 0 0 0 0 0 0 0 0 0 0 0 0 0 0 0 0 0 0 0 0 0 6 0 2 0 0 6 0 0 0 5 8 6 6 0 0 4 7 7 6 0

"208049_fr4" 0 0 0 0 0 0 0 0 0 0 0 0 0 0 0 0 0 0 0 0 0 0 0 0 0 0 0 0 0 0 0 0 0 0 0 0 0 0 0 7 0 0 0 0 0 0 0 0 0 0 0 0 0 0 0 0 0 0 0 0 0 0 0 0 0 0 0 0 0 0 0 0 0 0 0 0 0 0 0 0 0 0 0 0 0

"208354_fr2" 0 0 0 0 0 0 0 0 0 0 0 0 0 0 0 0 0 0 0 0 0 0 0 0 0 0 0 0 0 0 0 0 0 0 0 0 0 0 0 0 0 0 0 0 0 0 0 0 0 0 0 0 0 0 0 0 0 0 0 0 0 0 0 0 0 0 0 0 0 0 0 0 3 0 3 0 3 0 0 0 0 0 0 0 0

"208477_fr1" 0 2 0 0 1 2 0 0 0 2 0 1 1 2 0 2 2 0 2 2 2 2 1 2 0 1 0 0 0 1 2 0 0 1 0 0 0 0 0 0 0 0 0 0 0 0 0 0 0 0 0 0 0 0 0 0 0 0 0 0 0 0 0 0 0 0 0 0 0 0 0 0 0 0 0 0 0 0 0 0 0 0 0 0 0

"208646_fr4" 0 0 0 0 0 0 0 0 0 4 0 0 0 0 0 0 0 2 0 0 0 0 0 0 0 0 0 0 0 0 0 0 0 0 0 0 0 0 0 0 0 0 0 0 0 0 0 0 0 0 0 0 0 0 0 0 0 0 0 0 0 0 0 0 0 0 0 0 0 0 0 0 0 0 0 0 0 0 0 0 0 0 0 0 0

"208953_fr6" 0 0 2 0 0 0 0 0 0 0 0 0 0 0 0 0 0 0 0 0 0 0 0 0 0 0 0 0 0 0 0 0 0 0 0 0 0 0 0 0 0 0 0 0 0 0 0 0 0 0 0 0 0 0 0 0 0 0 0 0 0 0 0 0 0 0 0 0 0 0 0 0 0 0 0 0 0 0 0 0 0 0 0 0 0

"20915_fr6" 0 0 0 0 0 0 0 0 0 0 0 0 0 0 5 0 0 0 0 0 0 0 0 0 0 0 0 0 0 0 0 0 0 0 0 0 0 0 0 0 0 0 0 0 0 0 0 0 0 0 0 0 0 0 0 0 0 0 0 0 0 0 0 0 0 0 0 0 0 0 0 0 0 0 0 0 0 0 0 0 0 0 0 0 0

"20931_fr2" 0 0 0 1 0 0 0 0 0 0 0 0 0 0 0 0 0 0 0 0 0 0 0 0 0 0 0 0 0 1 0 0 0 0 0 0 0 0 0 0 0 0 0 0 0 0 0 0 0 0 0 0 0 0 0 0 0 0 0 0 0 0 0 0 0 0 0 0 0 0 0 0 0 0 0 0 0 0 0 0 0 0 0 0 0

"209438_fr3" 8 10 16 17 0 12 4 13 11 13 0 14 3 5 2 0 1 0 0 3 4 0 14 0 10 14 16 0 14 0 12 7 6 1 5 3 0 2 0 3 0 0 0 0 0 0 0 0 0 0 0 0 0 0 0 0 0 1 0 0 0 0 0 0 0 0 0 0 0 0 0 0 0 0 0 2 11 11 13 12 11 8 10 13 11

"20974_fr4" 0 0 0 0 0 0 0 0 0 0 0 0 0 0 0 0 0 0 0 0 0 0 0 0 0 1 0 0 0 3 0 4 0 0 0 0 0 0 0 0 0 0 0 0 0 0 0 0 0 0 0 0 0 0 0 0 0 0 0 0 0 0 0 0 0 0 0 0 0 0 0 0 0 0 0 0 0 0 0 0 0 0 0 0 0

"20975_fr4" 4 3 5 19 4 3 4 7 16 12 7 4 6 7 13 6 4 16 6 5 0 7 1 4 19 3 1 0 10 1 5 0 10 6 18 0 0 0 0 0 0 0 0 0 0 0 0 0 0 0 0 0 0 0 0 0 0 0 0 0 0 0 0 0 0 0 0 0 0 0 0 0 0 0 0 0 2 1 1 0 3 2 9 2 10

"20978_fr3" 0 1 0 2 3 1 0 1 3 0 1 0 7 6 7 5 7 0 8 8 6 0 4 4 0 4 4 3 5 3 2 2 2 8 6 0 0 0 0 0 0 0 0 0 0 0 0 0 0 0 0 0 0 0 0 0 0 0 0 0 0 0 0 0 0 0 0 0 0 0 0 0 0 0 0 0 0 0 0 0 0 0 0 0 0

"209919_fr3" 0 0 0 0 0 0 0 0 0 3 0 0 0 0 4 0 3 0 0 0 0 0 0 0 0 0 0 0 0 0 0 0 0 0 0 0 0 0 0 0 0 0 0 0 0 0 0 0 0 0 0 0 0 0 0 0 0 0 0 0 0 0 0 0 0 0 0 0 0 0 0 0 0 0 0 0 0 0 0 0 0 0 0 0 0

"209926_fr1" 0 0 0 4 0 0 0 0 4 0 0 0 0 0 0 0 0 4 0 0 0 3 0 0 0 0 0 0 0 0 0 0 0 0 4 0 0 0 0 0 0 0 0 0 0 0 0 0 0 0 0 0 0 0 0 0 0 0 0 0 0 0 0 0 0 0 0 0 0 0 0 0 0 0 0 0 0 0 0 0 0 0 0 0 0

"209971_fr2" 2 0 2 2 0 0 2 2 4 0 3 0 0 0 3 0 0 3 0 0 0 0 0 0 3 0 0 0 2 0 0 2 2 0 4 0 0 0 0 0 0 0 0 0 0 0 0 0 0 0 0 0 0 0 0 0 0 0 0 0 0 0 0 0 0 0 0 0 0 0 0 0 0 0 0 0 0 0 0 0 0 0 0 0 0

"2101_fr3" 3 3 1 2 2 2 3 4 3 1 2 4 6 7 4 7 7 6 6 7 6 5 4 7 6 6 6 4 6 8 4 5 8 6 6 0 0 0 0 0 0 0 0 0 0 0 0 0 0 0 0 0 0 0 0 0 0 0 0 0 0 0 0 0 1 1 1 0 0 1 3 0 0 2 3 0 5 7 6 1 0 0 1 1 2

"210246_fr2" 0 0 0 0 0 0 0 0 0 0 0 0 0 0 0 0 0 0 0 0 0 0 0 0 0 0 0 0 0 0 0 0 0 0 0 0 0 0 0 0 0 0 0 1 1 0 2 0 0 1 0 1 1 0 0 0 0 0 0 1 0 0 0 0 0 0 0 0 0 0 0 0 0 0 0 0 0 0 0 0 0 0 0 0 0

"21030_fr6" 0 0 5 4 0 0 0 0 0 0 0 0 0 0 0 0 0 0 0 0 0 0 0 0 0 0 7 0 3 0 0 0 0 0 0 0 0 0 0 0 0 0 0 0 0 0 0 0 0 0 0 0 0 0 0 0 0 0 0 0 0 0 0 0 0 0 0 0 0 0 0 0 0 0 0 0 0 0 0 0 0 0 0 0 0

"210532_fr6" 0 4 0 0 0 0 0 0 0 0 0 1 0 0 0 0 0 0 0 0 0 0 2 0 0 0 0 0 0 0 4 0 0 0 0 0 0 0 0 0 0 0 0 0 0 0 0 0 0 0 0 0 0 0 0 0 0 0 0 0 0 0 0 0 0 0 0 0 0 0 0 0 0 0 0 0 0 2 0 0 4 0 0 4 0

"210555_fr5" 0 0 0 0 2 6 0 0 0 0 0 0 0 2 0 0 1 0 4 1 4 0 2 0 0 1 4 0 0 1 0 2 2 2 0 0 0 0 0 0 0 0 0 0 0 0 0 0 0 0 0 0 0 0 0 0 0 0 0 0 0 0 0 0 0 0 0 3 0 0 0 0 0 2 0 0 0 0 6 3 0 0 0 0 0

"210630_fr5" 0 0 0 0 0 0 0 0 0 0 0 0 0 0 0 0 0 0 0 0 0 0 0 0 0 0 0 4 0 0 0 0 0 0 0 0 0 0 0 0 0 0 0 0 0 0 0 0 0 0 0 0 0 0 0 0 0 0 0 0 0 0 0 0 0 0 0 0 0 0 0 0 0 0 0 0 0 0 0 0 0 0 0 0 0

"210668_fr5" 0 0 0 0 0 0 0 0 0 0 0 0 0 0 0 0 0 0 0 0 0 0 0 0 0 0 0 0 0 0 0 0 0 0 0 0 0 0 0 0 0 0 0 0 0 0 0 0 0 0 0 0 0 0 0 0 0 0 0 0 0 0 0 0 0 0 0 0 0 0 0 0 0 0 0 3 0 0 0 0 0 0 0 0 0

"210817_fr6" 0 0 0 0 0 0 0 0 0 0 0 0 0 0 0 0 0 0 1 0 0 0 0 0 0 0 0 0 0 0 0 0 0 0 0 0 0 0 0 0 0 0 0 0 0 0 0 0 0 0 0 0 0 0 0 0 0 0 0 0 0 0 0 0 0 0 0 0 0 0 0 0 0 0 0 0 0 0 0 0 0 0 0 0 0

"210873_fr1" 0 0 0 0 0 0 0 0 0 0 0 0 0 2 0 0 0 0 0 2 2 0 2 2 0 2 0 0 0 3 0 0 0 0 0 0 0 0 0 0 0 0 0 0 0 0 0 0 0 0 0 0 0 0 0 0 0 0 0 0 0 0 0 0 0 0 0 0 0 0 0 0 0 0 0 0 0 0 0 0 0 0 0 0 0

"210885_fr3" 0 2 2 4 0 3 0 5 4 0 0 0 0 0 0 0 0 0 0 0 0 0 0 0 0 0 6 0 3 0 0 2 0 0 0 0 0 0 2 0 0 0 0 0 0 0 0 0 0 0 0 0 1 0 0 2 3 4 1 0 0 2 0 0 0 0 0 0 0 0 0 0 0 0 0 0 2 0 2 2 0 0 0 2 0

"211002_fr6" 0 0 2 3 0 2 0 2 2 0 0 2 0 2 1 1 0 0 0 2 0 1 1 0 0 2 2 0 2 2 2 0 1 0 2 0 0 0 0 0 0 0 0 0 0 0 0 0 0 0 0 0 0 0 0 0 0 0 0 0 0 0 0 0 0 0 0 0 0 0 0 0 0 0 0 0 0 0 0 0 0 0 0 0 0

"211426_fr6" 0 0 0 0 0 0 0 0 0 0 0 0 0 0 0 0 0 0 0 0 0 0 0 0 0 0 0 0 0 0 0 0 0 0 0 0 3 0 0 0 0 0 0 0 0 0 0 0 0 0 0 0 0 0 0 0 0 0 0 0 0 0 0 0 0 0 0 0 0 0 0 0 0 0 0 0 0 0 0 0 0 0 0 0 0

"21251_fr1" 0 0 0 0 0 0 0 0 0 1 0 0 9 0 0 0 0 0 0 0 0 0 0 0 2 0 0 9 0 0 0 0 0 0 0 0 0 0 0 0 0 0 0 0 0 0 0 0 0 0 0 0 0 0 0 0 0 0 0 0 0 0 0 0 0 0 4 0 0 0 1 1 3 2 3 4 4 4 5 0 4 0 3 0 0

"21264_fr5" 0 0 0 0 0 0 0 0 0 0 0 0 0 2 0 0 4 0 1 0 0 2 1 4 0 0 2 0 1 0 0 0 0 0 0 0 0 0 0 0 0 0 0 0 0 0 0 0 0 0 0 0 0 0 0 0 0 0 0 0 0 0 0 0 0 0 0 0 0 0 0 0 0 0 0 0 0 0 0 0 0 0 0 0 0

"212929_fr6" 0 0 0 0 0 1 0 0 0 0 0 0 0 4 0 0 0 0 0 0 0 0 0 0 0 0 0 0 0 0 0 0 0 0 0 0 0 0 0 0 0 0 0 0 0 0 0 0 0 0 0 0 0 0 0 0 0 0 0 0 0 0 0 0 0 0 0 0 0 0 0 0 0 0 0 0 0 0 0 0 0 0 0 0 0

"213_fr1" 0 0 0 0 0 0 0 0 0 0 0 0 0 0 0 0 0 0 0 0 0 0 0 0 0 0 2 0 0 0 0 0 0 0 0 0 0 0 0 0 0 0 0 0 0 0 0 0 0 0 0 0 0 0 0 0 0 0 0 0 0 0 0 0 0 0 0 0 0 0 0 0 0 0 0 0 0 0 0 0 0 0 0 0 0

"213317_fr5" 0 0 0 0 0 0 0 0 0 2 0 0 0 0 0 0 0 0 0 0 0 0 0 0 0 0 0 0 0 0 0 0 0 0 0 0 0 0 0 0 0 0 0 0 0 0 0 0 0 0 0 0 0 0 0 0 0 0 0 0 0 0 0 0 0 0 0 0 0 0 0 0 0 0 0 0 0 0 0 0 0 0 0 0 0

"213376_fr3" 0 0 0 0 0 0 0 0 0 5 0 0 5 0 0 0 0 0 0 0 0 0 0 0 0 0 0 0 0 0 0 0 0 0 0 0 0 0 0 0 0 0 0 0 0 0 0 0 0 0 0 0 0 0 0 0 0 0 0 0 0 0 0 0 0 0 0 0 0 0 0 0 0 0 0 0 0 0 0 0 0 0 0 0 0

"21346_fr2" 5 2 7 0 8 6 6 0 0 0 4 0 3 8 3 0 0 3 5 7 5 4 7 8 0 8 7 0 4 7 4 4 7 6 0 0 0 0 0 0 0 0 0 0 0 0 0 0 0 0 0 0 0 0 0 0 0 0 0 0 0 0 0 0 0 0 0 0 0 0 0 0 0 0 0 0 0 0 0 0 0 0 0 0 0

"213827_fr4" 2 0 0 0 3 3 0 0 0 0 2 0 0 0 0 0 0 0 0 0 0 3 4 2 3 1 0 0 1 4 0 1 4 4 0 0 0 0 0 0 0 0 0 0 0 0 0 0 0 0 0 0 0 0 0 0 0 0 0 0 0 0 0 0 0 0 0 0 0 0 0 0 0 0 0 0 0 0 0 0 0 0 0 0 0

"21424_fr5" 9 8 7 7 12 22 0 7 5 0 23 19 2 4 17 2 19 2 12 18 3 11 14 12 1 27 6 3 14 14 9 6 5 17 17 0 0 0 0 0 0 0 0 0 0 0 0 0 0 0 0 0 0 0 0 0 0 0 0 0 0 0 0 0 0 0 0 0 0 0 1 0 1 0 1 0 2 8 2 2 0 3 11 1 1

"2151_fr2" 0 0 1 2 0 4 0 0 0 0 0 0 0 0 0 0 0 0 0 0 2 0 2 0 0 1 0 4 0 1 0 2 0 0 1 0 0 0 0 0 0 0 0 0 0 0 0 0 0 0 0 0 0 0 0 0 0 0 0 0 0 0 0 0 0 0 0 0 0 0 0 0 0 0 0 0 0 0 0 0 0 0 0 0 0

"21529_fr6" 1 2 4 1 4 3 4 3 2 3 3 4 3 3 2 3 3 4 2 3 3 4 4 3 2 3 4 2 4 2 4 4 4 4 4 0 0 3 0 1 0 0 0 1 1 0 0 0 0 0 0 0 0 0 0 0 0 0 0 0 0 0 0 0 0 1 2 0 2 1 0 0 0 0 0 2 21 21 8 6 3 3 4 1 3

"21564_fr6" 0 0 0 0 0 0 0 0 3 0 0 0 0 0 0 0 0 2 0 0 0 0 0 0 0 0 0 0 0 0 0 0 0 0 1 0 0 0 0 0 0 0 0 0 0 0 0 0 0 0 0 0 0 0 0 0 0 0 0 0 0 0 0 0 0 0 0 0 0 0 0 0 0 0 0 0 0 0 0 0 0 0 0 0 0

"21574_fr2" 0 0 1 0 0 0 0 0 0 0 2 0 1 0 0 0 0 0 1 0 0 0 1 3 0 0 4 0 0 0 0 0 0 2 0 0 0 0 0 0 0 0 0 0 0 0 0 0 0 0 0 0 0 0 0 0 0 0 0 0 0 0 0 0 0 0 0 0 0 0 0 0 0 0 0 0 0 0 0 0 0 0 0 0 0

"216470_fr4" 0 0 0 0 0 0 0 0 0 0 0 0 3 0 0 0 0 0 0 0 0 0 0 0 0 0 0 0 0 0 0 0 0 0 0 0 0 0 0 0 0 0 0 0 0 0 0 0 0 0 0 0 0 0 0 0 0 0 0 0 0 0 0 0 0 0 0 0 0 0 0 0 0 0 0 0 0 0 0 0 0 0 0 0 0

"216880_fr6" 2 3 0 1 5 0 0 0 4 8 0 0 1 0 1 0 0 6 0 0 0 2 5 4 8 0 0 3 0 3 0 6 6 0 6 0 0 0 0 0 0 0 0 0 0 0 0 0 0 0 0 0 0 0 0 0 0 0 0 0 0 0 0 0 0 0 0 1 1 0 0 3 0 0 0 0 0 0 0 1 0 3 0 3 3

"216940_fr5" 0 0 0 0 0 4 0 0 0 0 4 0 0 0 0 0 0 0 0 4 0 0 0 0 0 4 2 0 0 1 0 1 0 5 1 0 0 0 0 0 0 0 0 0 0 0 0 0 0 0 0 0 0 0 0 0 0 0 0 0 0 0 0 0 0 0 0 0 0 0 0 0 0 0 0 0 0 0 0 0 0 0 0 0 0

"216982_fr4" 0 0 0 0 0 0 0 0 0 0 0 0 0 0 0 0 0 0 0 0 0 0 0 0 0 0 0 0 0 0 0 0 0 0 0 0 0 0 0 0 0 0 0 0 0 0 0 0 0 0 0 0 0 0 0 0 0 0 0 0 0 0 0 0 0 0 0 0 0 0 0 0 0 0 0 0 2 0 0 3 0 0 0 0 0

"217140_fr3" 0 0 0 0 0 0 0 0 0 0 0 0 0 0 0 0 0 0 0 0 0 0 0 0 0 0 0 0 0 0 0 0 0 0 0 0 0 0 0 0 0 0 0 0 0 0 0 0 0 0 0 0 0 0 0 0 0 0 0 0 0 0 0 0 0 0 0 0 0 0 0 3 0 0 0 0 0 0 0 0 0 0 0 0 0

"217166_fr2" 4 0 4 3 0 4 3 3 3 0 0 0 0 0 4 4 4 0 0 4 0 4 0 4 0 0 0 0 4 0 3 0 3 4 4 0 0 0 0 0 0 0 0 0 0 0 0 0 0 0 0 0 0 0 0 0 0 0 0 0 0 0 0 0 0 0 0 0 0 0 0 0 0 0 0 0 0 0 0 0 0 0 0 0 0

"217581_fr6" 0 0 0 0 0 0 0 0 0 1 0 0 0 0 0 0 0 0 0 0 0 0 0 0 0 0 0 0 0 0 0 0 0 0 0 0 0 0 0 0 0 0 0 0 0 0 0 0 0 0 0 0 0 0 0 0 0 0 0 0 0 0 0 0 0 0 0 0 0 0 0 0 0 0 0 0 0 0 0 0 0 0 0 0 0

"217606_fr6" 0 0 0 0 0 0 0 0 0 0 0 0 0 0 0 0 0 0 0 0 0 0 0 0 0 0 0 0 0 0 0 0 0 0 0 0 0 0 0 0 0 0 0 0 0 0 0 0 0 0 0 0 0 0 0 0 0 0 0 0 0 0 0 0 0 0 0 0 0 0 0 0 0 0 0 0 0 0 0 3 0 0 0 0 0

"217647_fr4" 0 3 0 0 5 0 0 0 0 0 0 0 0 0 0 0 0 0 0 0 0 0 3 0 0 0 0 0 0 0 0 3 0 0 0 0 0 0 0 0 0 0 0 0 0 0 0 0 0 0 0 0 0 0 0 0 0 0 0 0 0 0 0 0 0 0 0 0 0 0 0 0 0 0 0 0 0 0 0 0 0 0 0 0 0

"217725_fr5" 0 0 0 0 0 0 0 0 0 0 0 0 0 0 0 0 0 0 0 0 0 0 0 0 6 0 0 0 0 0 0 0 0 0 0 0 0 0 0 0 0 0 0 0 0 0 0 0 0 0 0 0 0 0 0 0 0 0 0 0 0 0 0 0 0 0 0 0 0 0 0 0 0 0 0 0 0 0 0 0 0 0 0 0 0

"217864_fr5" 0 0 0 0 0 0 0 0 0 0 0 0 0 0 0 0 0 0 0 0 0 0 0 0 0 0 0 0 0 0 0 0 0 0 0 0 0 2 0 0 0 0 0 0 0 2 0 2 0 1 0 0 0 0 3 0 0 0 0 0 0 3 0 1 0 0 0 0 0 0 0 0 0 0 0 0 0 0 0 0 0 0 0 0 0

"21803_fr4" 0 1 3 1 0 1 1 3 0 0 0 0 0 0 0 1 0 1 0 0 0 0 0 1 0 0 2 0 0 0 0 1 2 0 0 0 0 0 0 0 0 0 0 0 0 0 0 0 0 0 0 0 0 0 0 0 0 0 0 0 0 0 0 0 0 0 0 0 0 0 0 0 0 0 0 0 0 0 0 0 0 0 0 0 0

"21834_fr4" 3 3 0 0 5 1 0 0 3 0 0 1 0 4 5 1 0 0 2 7 1 1 4 2 0 2 0 2 0 6 4 2 2 3 1 0 0 0 0 0 0 0 0 0 0 0 0 0 0 0 0 0 0 0 0 0 0 0 0 0 0 0 0 0 0 0 0 0 0 0 0 0 0 0 0 0 0 0 0 0 0 0 0 0 0

"218527_fr2" 0 0 0 0 0 0 0 0 0 0 0 0 0 0 0 0 0 0 0 0 0 0 0 0 0 0 0 0 0 0 0 0 0 0 0 0 1 0 0 2 3 0 3 5 5 0 4 0 0 3 0 0 0 0 0 2 3 3 0 0 1 0 3 0 0 0 0 1 0 1 0 0 0 0 0 0 0 0 0 0 0 0 0 0 0

"21971_fr5" 0 0 0 0 0 0 0 0 0 0 0 0 0 0 0 0 0 0 0 0 0 0 0 0 0 0 0 0 0 0 0 0 0 0 0 0 0 0 0 0 0 0 0 0 0 0 0 0 0 0 0 0 0 0 0 0 0 0 0 0 0 0 0 0 0 0 0 0 0 0 0 0 0 0 0 0 2 0 0 0 0 0 0 0 0

"22001_fr2" 0 0 0 0 0 0 0 0 0 0 0 0 0 0 0 0 0 0 0 0 0 0 0 0 0 0 0 0 0 0 0 0 0 0 0 0 0 0 0 0 0 0 0 0 0 0 0 0 0 0 0 0 0 0 0 0 0 0 0 0 0 0 0 0 0 0 0 0 0 0 0 0 0 0 0 0 0 0 6 0 0 0 0 3 0

"22004_fr1" 0 0 0 2 0 0 0 0 0 0 0 0 0 0 1 0 0 0 0 0 0 0 0 0 0 0 0 0 0 0 0 0 0 0 4 0 0 0 0 0 0 0 0 0 0 4 3 4 0 0 0 0 0 0 0 0 0 0 0 0 0 0 0 0 0 0 0 0 0 0 0 0 0 0 0 0 0 0 0 0 0 0 0 0 0

"22040_fr1" 0 0 0 0 0 0 0 0 0 0 0 0 0 0 0 0 0 0 0 0 0 0 0 0 0 0 0 0 0 0 0 0 0 3 0 0 0 0 0 0 0 0 0 0 0 0 0 0 0 0 0 0 0 0 0 0 0 0 0 0 0 0 0 0 0 0 0 0 0 0 0 0 0 0 0 0 0 0 0 0 0 0 0 0 0

"22232_fr3" 0 0 0 2 3 0 0 0 0 0 0 0 0 0 0 0 1 0 0 0 1 0 3 2 0 1 0 0 4 3 0 3 0 1 0 0 0 0 0 0 0 0 0 0 0 0 0 0 0 0 0 0 0 0 0 0 0 0 0 0 0 0 0 0 0 0 0 0 0 0 0 0 0 0 0 0 0 0 0 0 0 0 0 0 0

"22255_fr2" 24 23 17 44 8 23 21 19 41 9 29 19 14 15 40 23 21 17 12 16 19 37 16 30 36 11 5 15 10 2 17 19 12 25 38 4 8 3 0 4 0 0 7 0 0 0 0 0 0 0 0 0 0 0 0 0 4 0 0 0 0 0 0 0 0 0 0 0 0 0 0 0 3 0 0 5 6 5 6 2 16 29 20 29 33

"22261_fr6" 0 4 0 0 0 0 0 0 0 0 3 0 2 0 2 0 0 4 0 0 0 0 0 0 0 0 0 0 0 0 4 0 0 0 2 0 0 0 0 0 0 0 0 0 0 0 0 0 0 0 0 0 0 0 0 0 0 0 0 0 0 0 0 0 0 0 0 0 0 0 0 0 0 0 0 0 0 0 0 0 0 0 0 0 0

"22266_fr2" 0 0 0 0 1 0 3 0 0 2 1 1 1 0 1 1 0 1 0 1 0 1 0 1 1 0 0 0 0 0 1 0 1 0 2 0 0 0 0 0 0 0 0 0 0 0 0 0 0 0 0 0 0 0 0 0 0 0 0 0 0 0 0 0 0 0 0 0 0 0 0 0 0 0 0 0 0 0 0 0 0 0 0 0 0

"22276_fr1" 0 0 0 0 1 0 0 0 0 0 0 0 0 0 0 0 0 0 1 1 0 0 2 0 0 0 0 0 0 0 0 0 0 1 0 0 0 0 0 0 0 0 0 0 0 0 0 0 0 0 0 0 0 0 0 0 0 0 0 0 0 0 0 0 0 0 0 0 0 0 0 0 0 0 0 0 0 0 0 0 0 0 0 0 0

"2235_fr4" 0 0 0 0 2 0 0 0 0 0 1 1 0 0 0 0 0 0 2 0 0 0 0 2 0 0 0 3 3 3 0 2 0 2 1 0 0 0 0 0 0 0 0 0 0 0 0 0 0 0 0 0 0 0 0 0 0 0 0 0 0 0 0 0 0 0 0 0 0 0 0 0 0 0 0 0 0 0 0 0 0 0 0 0 0

"22376_fr4" 0 0 0 0 0 0 0 0 0 0 0 0 0 0 0 0 0 0 0 0 0 0 0 0 0 0 0 0 0 0 0 0 0 0 0 0 0 0 0 0 0 0 0 0 0 0 0 0 0 0 0 0 0 0 0 0 0 0 0 0 0 0 0 0 0 0 0 0 0 0 0 2 0 0 0 0 0 0 0 0 0 0 0 0 0

"22690_fr6" 5 8 4 0 5 2 0 3 0 0 5 5 4 8 3 6 2 4 5 6 0 5 9 9 0 6 0 6 0 6 7 7 4 5 0 0 0 0 0 0 0 0 0 0 0 0 0 0 0 0 0 0 0 0 0 0 0 0 0 0 0 0 0 0 0 0 0 0 0 0 0 0 0 0 0 0 0 0 0 0 0 0 0 0 0

"22707_fr2" 0 0 0 0 0 1 0 0 0 0 0 0 0 1 0 1 1 0 0 3 0 0 0 0 0 0 0 1 1 0 0 0 0 0 0 0 0 0 0 0 0 0 0 0 0 0 0 0 0 0 0 0 0 0 0 0 0 0 0 0 0 0 0 0 0 0 0 0 0 0 0 0 0 0 0 0 0 0 0 0 0 0 0 0 0

"2278_fr6" 5 8 13 13 12 12 5 14 11 9 11 4 13 8 11 7 6 9 4 9 7 7 10 9 13 7 12 8 9 7 9 11 13 11 8 0 0 0 0 0 0 0 3 0 0 0 0 0 0 0 0 0 0 0 0 0 0 0 0 0 0 0 0 0 0 0 0 0 0 0 0 0 0 0 0 0 2 3 2 2 0 4 3 2 5

"22840_fr6" 0 0 0 0 0 0 0 0 0 0 0 0 0 0 0 0 0 0 0 0 0 0 0 0 0 0 0 0 0 0 0 0 0 0 0 0 0 0 0 0 0 0 0 0 0 0 0 0 6 0 0 0 0 0 0 4 0 0 0 0 0 0 0 0 0 5 0 4 0 2 3 9 5 0 0 0 0 0 7 0 0 0 0 2 0

"2292_fr2" 1 3 0 0 0 0 7 0 0 0 0 0 7 3 2 3 2 3 2 2 2 0 3 0 0 5 2 2 0 0 2 4 2 3 0 0 0 0 0 0 0 0 0 0 0 0 0 0 0 0 0 0 0 0 0 0 0 0 0 0 0 0 0 0 0 0 3 3 3 0 4 3 6 6 3 3 6 4 6 5 2 2 4 3 3

"22990_fr6" 0 0 0 0 0 0 0 0 0 0 0 0 0 0 0 0 0 0 0 0 0 0 0 0 0 0 0 0 0 0 0 0 0 0 0 0 2 0 0 0 0 0 0 0 0 0 0 0 3 0 0 0 0 1 0 2 0 0 0 0 0 0 0 0 0 0 0 0 0 0 0 0 0 0 0 0 0 0 0 0 0 0 0 0 0

"2308_fr6" 0 0 2 0 2 0 0 0 2 2 2 0 0 0 0 1 2 2 3 3 2 0 2 2 2 0 2 0 0 11 2 2 0 0 11 0 0 0 0 0 0 0 0 0 0 0 0 0 0 0 0 0 0 0 0 0 0 0 0 0 0 0 0 0 0 0 0 0 0 0 0 0 0 0 0 0 0 0 0 0 0 0 0 0 0

"23125_fr3" 0 0 0 0 0 0 0 0 2 0 0 0 0 0 0 0 0 1 0 0 0 0 0 2 0 0 0 0 2 0 0 0 0 0 4 0 0 0 0 0 0 0 0 0 0 0 0 0 0 0 0 0 0 0 0 0 0 0 0 0 0 0 0 0 0 0 0 0 0 0 0 0 0 0 0 0 0 0 0 0 0 0 0 0 0

"23132_fr4" 0 0 0 1 2 0 0 0 0 0 0 0 0 0 1 0 0 0 0 0 0 0 0 0 0 0 0 0 0 2 0 2 0 0 0 0 0 0 0 0 0 0 0 0 0 0 0 0 0 0 0 0 0 0 0 0 0 0 0 0 0 0 0 0 0 0 0 0 0 0 0 0 0 0 0 0 0 0 0 0 0 0 0 0 0

"23321_fr2" 2 0 1 3 0 0 2 0 3 0 3 0 1 0 4 4 3 3 0 0 0 1 0 0 4 0 0 0 0 0 4 0 0 0 3 0 0 0 0 0 0 0 0 0 0 0 0 0 0 0 0 0 0 0 0 0 0 0 0 0 0 0 0 0 0 0 0 0 0 0 0 0 0 0 0 0 0 0 0 0 2 2 0 1 3

"23428_fr4" 7 9 6 0 12 0 6 6 4 0 15 12 0 6 0 0 7 0 10 6 10 3 14 10 2 6 0 8 1 11 2 10 6 5 0 0 0 5 0 5 0 2 4 0 6 0 0 0 0 0 0 0 0 0 5 0 0 2 0 0 0 0 0 0 0 3 2 2 3 2 0 0 4 0 3 5 6 11 11 2 5 4 3 6 0

"23487_fr5" 0 0 0 0 0 0 0 0 0 0 0 0 0 0 0 0 0 0 0 0 0 0 0 0 0 0 0 0 0 0 0 0 0 0 0 0 0 0 0 0 0 0 0 0 0 0 0 0 0 0 0 0 0 0 0 0 0 0 0 0 0 0 0 0 0 0 0 0 0 0 0 0 0 3 5 0 0 0 0 4 0 0 0 0 0

"23556_fr2" 0 0 7 0 0 7 0 0 0 0 13 0 0 0 0 0 0 5 0 0 0 0 0 0 0 0 4 0 0 0 0 0 0 10 1 0 0 0 0 0 0 0 0 0 0 0 0 0 0 0 0 0 0 0 0 0 0 0 0 0 0 0 0 0 0 0 0 0 0 0 0 0 0 0 0 0 0 0 0 0 0 0 0 0 0

"23593_fr2" 0 0 0 0 1 0 0 0 0 0 0 0 0 2 0 0 3 0 0 2 0 0 2 4 0 0 1 0 0 3 0 1 2 0 0 0 0 0 0 0 0 0 0 0 0 0 0 0 0 0 0 0 0 0 0 0 0 0 0 0 0 0 0 0 0 0 0 0 1 0 0 0 2 2 3 4 4 1 0 0 0 0 0 0 0

"23850_fr4" 0 0 0 0 0 0 0 0 0 0 0 0 0 0 0 0 0 0 0 0 0 0 0 0 0 0 0 0 0 0 0 0 0 0 0 2 2 5 0 2 0 0 0 0 6 0 4 0 0 0 0 0 0 3 1 0 0 0 0 0 0 0 0 0 0 0 0 0 0 0 0 0 0 0 0 0 0 0 0 0 0 0 0 0 0

"23928_fr5" 0 2 3 0 2 3 4 0 0 0 0 0 2 0 0 2 2 0 0 0 0 1 0 5 2 0 2 1 2 2 0 5 0 5 0 0 0 0 0 0 0 0 0 0 0 0 0 0 0 0 0 0 0 0 0 0 0 0 0 0 0 0 0 0 0 0 0 0 0 0 0 0 0 0 0 0 0 0 0 0 0 0 0 0 0

"24066_fr3" 2 4 2 6 2 2 0 6 2 0 2 2 2 2 4 2 2 4 6 2 2 0 5 6 0 2 5 2 2 0 0 5 2 6 0 0 0 0 0 0 0 0 0 0 0 0 0 0 0 0 0 0 0 0 0 0 0 0 0 0 0 0 0 0 0 0 0 0 0 0 0 0 0 0 1 2 3 5 2 0 4 4 3 0 0

"24240_fr3" 0 0 0 0 0 0 0 0 0 0 0 0 0 0 0 0 0 0 0 0 0 0 0 0 0 0 0 0 0 0 0 0 0 0 0 0 0 0 0 0 0 0 0 0 0 0 0 0 0 0 0 0 0 0 0 0 0 0 0 0 0 0 0 0 0 0 0 0 0 0 0 0 1 3 1 0 0 0 0 0 0 0 4 0 0

"24256_fr1" 0 0 0 0 0 0 0 0 0 0 0 0 0 0 0 0 0 0 0 0 0 0 0 0 0 0 0 0 0 0 0 0 0 0 0 0 0 0 0 0 0 0 0 0 0 0 0 0 0 0 0 0 0 0 0 0 0 0 0 0 0 0 0 0 0 0 0 0 0 0 0 3 2 0 0 0 0 0 0 0 0 0 0 0 0

"24285_fr5" 0 0 0 0 0 0 0 0 0 0 0 0 0 0 0 0 0 0 0 0 0 0 0 0 0 0 0 0 0 0 0 0 0 0 0 0 0 0 0 0 0 0 0 0 0 0 0 0 0 0 0 0 0 0 0 0 0 0 0 0 0 0 0 0 0 0 3 0 0 0 1 0 0 0 0 0 0 0 0 0 0 0 0 0 0

"24391_fr3" 0 0 0 0 0 0 0 0 0 0 0 0 0 0 0 0 0 4 0 0 0 0 0 0 0 0 0 0 0 0 0 0 0 0 0 0 0 0 0 0 0 0 0 0 0 0 0 0 0 0 0 0 0 0 0 0 0 0 0 0 0 0 0 0 0 0 0 0 0 0 0 0 0 0 0 3 0 0 0 0 0 0 0 0 0

"24461_fr4" 10 10 0 0 9 10 12 0 9 0 0 9 15 13 10 16 14 11 10 9 0 10 0 0 0 0 0 0 0 13 0 0 12 11 0 0 0 0 0 0 0 0 0 0 0 0 0 0 0 0 0 0 0 0 0 0 0 0 0 0 0 0 0 0 0 10 0 0 8 0 11 10 6 13 9 7 11 9 11 10 12 12 12 12 14

"2450_fr1" 0 4 4 0 5 0 0 0 0 0 0 5 0 0 0 0 0 1 0 2 0 0 4 2 0 5 3 0 1 2 3 5 1 0 0 0 0 0 0 0 0 0 0 0 0 0 0 0 0 0 0 0 0 0 0 0 0 0 0 0 0 0 0 0 0 0 0 0 0 0 0 0 0 0 0 0 0 0 0 0 0 0 0 0 0

"24570_fr5" 0 0 0 0 0 0 0 0 0 0 0 0 0 0 0 0 0 0 0 0 0 0 0 0 0 0 0 0 0 0 0 0 0 0 0 2 7 10 16 13 36 24 25 18 30 23 21 22 22 34 24 21 24 27 26 20 22 23 21 22 21 16 18 17 17 18 14 18 6 14 12 10 8 13 14 4 8 8 4 4 6 8 4 0 7

"2459_fr1" 0 0 0 0 0 1 0 0 0 0 0 0 0 0 0 0 0 0 0 0 2 0 0 0 0 0 4 0 0 0 0 0 1 0 0 0 0 0 0 0 0 0 0 0 0 0 0 0 0 0 0 0 0 0 0 0 0 0 0 0 0 0 0 0 0 0 0 0 0 0 0 0 0 0 0 0 0 0 0 0 0 0 0 0 0

"2460_fr1" 0 0 0 0 0 0 0 0 0 6 0 0 5 0 0 0 0 0 0 0 0 0 0 0 0 0 0 0 0 0 0 0 0 0 0 0 0 0 0 0 0 0 0 0 0 0 0 0 0 0 0 0 0 0 0 0 0 0 0 0 0 0 0 0 0 0 0 0 0 0 0 0 0 0 0 0 0 0 0 0 0 0 0 0 0

"24682_fr1" 0 0 0 0 1 0 0 0 0 0 0 0 0 1 0 0 0 0 0 0 0 0 0 0 0 0 0 0 0 1 0 2 0 0 0 0 0 0 0 0 0 0 0 0 0 0 0 0 0 0 0 0 0 0 0 0 0 0 0 0 0 0 0 0 0 0 0 0 0 0 0 0 0 0 0 0 0 0 0 0 0 0 0 0 0

"24754_fr2" 0 0 5 1 0 2 0 5 0 0 0 0 0 0 0 0 5 0 2 5 0 0 2 0 0 5 0 6 0 0 0 0 0 0 0 0 0 0 0 0 0 0 0 0 0 0 0 0 0 0 0 0 0 0 0 0 0 0 0 0 0 0 0 0 0 0 0 0 0 0 0 0 0 0 0 0 0 0 0 0 0 0 3 0 0

"24823_fr3" 4 2 2 0 7 0 0 3 2 5 5 6 4 11 0 0 8 5 6 9 2 10 3 2 5 12 0 4 2 6 6 4 4 9 0 0 3 0 0 3 0 0 0 0 2 0 0 0 5 0 0 0 0 0 0 0 0 0 0 0 0 0 0 0 0 0 0 0 0 0 0 0 2 4 0 2 2 2 2 7 2 2 2 2 3

"24835_fr4" 0 0 0 0 0 0 0 0 0 0 0 0 0 0 0 0 0 0 0 0 0 0 0 0 0 0 0 0 0 0 0 0 0 0 0 0 0 0 0 0 0 0 0 0 0 0 0 0 0 0 0 0 0 0 0 0 0 0 0 0 0 0 0 0 0 0 0 11 0 0 0 0 0 0 0 0 0 0 0 5 0 0 0 0 0

"24870_fr5" 4 2 2 3 1 0 2 4 4 0 4 0 1 0 4 2 0 1 0 4 5 4 1 4 4 2 2 0 3 3 0 0 3 3 3 0 0 0 0 0 0 0 0 0 0 0 0 0 0 0 0 0 0 0 0 0 0 0 0 0 0 0 0 0 0 0 0 0 0 0 0 0 0 0 0 0 0 0 0 0 0 0 0 0 0

"24870_fr6" 0 0 0 0 0 0 0 0 0 0 0 0 0 0 0 0 0 0 0 0 0 0 0 0 0 0 0 0 0 0 0 0 0 0 0 0 0 0 0 0 0 0 0 0 0 0 0 0 0 0 0 0 0 0 0 0 0 0 0 0 0 0 0 0 0 4 0 1 0 0 1 1 0 1 1 1 0 3 3 4 1 3 1 1 2

"24884_fr5" 0 0 0 0 0 0 0 0 0 0 0 0 0 0 0 0 0 0 0 0 0 0 0 0 0 0 0 0 0 0 0 0 0 0 0 0 0 0 0 0 0 0 0 0 0 0 0 0 0 0 0 0 0 0 0 0 0 0 0 0 0 0 0 0 0 0 0 0 0 0 0 0 4 0 0 0 0 0 0 0 0 0 0 0 0

"2493_fr4" 2 0 0 0 5 0 0 0 0 0 0 0 0 6 0 0 0 0 0 0 0 0 2 0 0 0 0 1 0 0 0 0 0 0 0 0 0 0 0 0 0 0 0 0 0 0 0 0 0 0 0 0 0 0 0 0 0 0 0 0 0 0 0 0 0 0 0 3 0 0 0 0 0 0 0 0 2 0 0 0 0 0 0 0 0

"25127_fr4" 0 0 0 0 0 0 0 0 0 0 0 0 0 0 0 0 0 0 0 0 0 0 0 0 0 0 0 0 0 0 0 0 0 0 0 3 3 4 6 5 1 5 5 8 15 8 5 12 7 20 17 20 8 41 22 9 8 5 12 41 4 4 3 4 3 1 0 1 0 2 0 0 0 0 0 0 0 0 0 0 0 0 0 0 0

"25141_fr3" 0 0 0 0 0 0 0 0 0 0 0 0 0 0 0 0 0 0 0 0 0 0 0 0 0 0 0 0 0 0 0 0 0 0 0 0 0 1 5 6 8 9 2 4 1 2 7 5 11 5 10 11 6 5 6 5 6 2 10 5 3 4 6 8 3 2 2 1 1 2 0 0 0 0 0 0 0 0 0 0 3 1 0 0 0

"25201_fr1" 0 0 0 0 0 0 0 0 0 0 0 0 0 0 0 0 0 0 0 0 0 0 0 0 0 0 0 0 0 0 0 4 0 0 0 0 0 0 0 0 0 0 0 0 0 0 0 0 0 0 0 0 0 0 0 0 0 0 0 0 0 0 0 0 0 0 0 0 0 0 0 0 0 0 0 0 0 0 0 0 0 0 0 0 0

"25209_fr4" 0 4 0 2 3 0 3 4 3 0 0 2 5 0 1 3 3 6 0 4 0 5 0 5 3 0 2 3 2 0 0 1 0 0 2 0 0 1 1 0 2 0 1 2 1 0 2 0 4 0 1 0 1 3 2 0 1 0 2 1 0 0 0 0 0 0 0 0 0 0 0 0 0 0 0 0 0 0 0 0 0 0 0 0 0

"2562_fr2" 0 0 0 0 0 2 0 0 0 0 0 0 0 13 0 0 8 0 12 0 8 0 6 0 0 4 0 2 2 9 0 2 5 0 0 0 0 0 0 0 0 0 0 0 0 0 0 0 0 0 0 0 0 0 0 0 0 0 0 0 0 0 0 0 0 0 0 0 0 0 0 0 0 0 0 0 0 0 0 0 0 0 0 0 0

"25797_fr3" 0 0 0 0 0 0 0 0 0 0 0 0 0 4 0 0 0 0 0 0 0 0 0 0 0 0 0 0 0 0 0 0 0 0 0 0 0 0 0 0 0 0 0 0 0 0 0 0 0 0 0 0 0 0 0 0 0 0 0 0 0 0 0 0 0 0 0 0 0 0 0 0 0 0 0 0 0 0 0 0 0 0 0 0 0

"25891_fr5" 0 0 0 0 0 1 0 0 0 0 0 0 0 0 0 0 0 0 0 0 0 0 0 0 0 0 0 0 0 0 0 0 0 1 0 0 0 0 0 0 0 0 0 0 0 0 0 0 0 0 0 0 0 0 0 0 0 0 0 0 0 0 0 0 0 0 0 0 0 0 0 0 0 0 0 0 0 0 0 0 0 0 0 0 0

"2598_fr4" 0 1 0 0 3 0 0 0 0 0 0 0 0 3 0 0 1 0 0 0 0 0 1 0 0 0 0 0 0 2 0 1 0 1 0 0 0 0 0 0 0 0 0 0 0 0 0 0 0 0 0 0 0 0 0 0 0 0 0 0 0 0 0 0 0 1 3 4 1 0 2 1 0 1 3 1 4 2 4 2 1 0 0 0 0

"26473_fr4" 0 0 0 0 0 0 0 0 0 0 0 0 0 0 0 0 0 0 0 0 0 0 2 0 0 0 0 0 0 0 0 0 3 0 0 0 0 0 0 0 0 0 0 0 0 0 0 0 0 0 0 0 0 0 0 0 0 0 0 0 0 0 0 0 0 0 0 0 0 0 0 0 0 0 0 0 0 0 0 0 0 0 0 0 0

"26896_fr2" 0 0 0 0 0 0 0 0 0 0 0 0 0 0 0 0 0 0 0 0 0 0 0 0 0 0 0 0 0 0 0 0 0 0 5 0 0 0 0 0 0 0 0 0 0 0 0 0 0 0 0 0 0 0 0 0 0 0 0 0 0 0 0 0 0 0 0 0 0 0 0 0 0 0 0 0 0 0 0 0 0 0 0 0 0

"2737_fr5" 0 0 0 0 0 0 0 0 0 0 0 0 0 0 0 0 0 0 0 0 0 0 0 0 0 0 0 0 0 0 0 0 0 0 0 0 0 0 0 0 0 0 0 0 0 0 0 0 0 0 0 0 0 0 0 0 0 0 0 0 0 0 0 0 0 0 0 0 0 0 0 0 0 0 0 0 0 2 1 0 0 0 0 0 0

"2745_fr2" 0 0 0 0 0 0 0 0 0 0 0 0 0 0 0 0 0 0 0 0 0 0 0 0 0 0 0 0 0 0 0 0 0 0 0 0 0 0 0 0 0 0 0 0 0 0 0 4 7 0 8 8 7 0 0 0 0 1 8 7 0 0 0 0 0 0 0 0 0 0 0 0 0 0 0 0 0 0 0 0 0 0 0 0 0

"276_fr4" 0 0 0 0 0 0 0 0 0 0 0 0 0 0 0 0 0 0 0 0 0 0 0 0 0 0 0 0 0 0 0 0 0 0 0 1 9 22 42 19 4 30 28 31 47 34 41 34 45 47 43 50 27 47 50 39 43 43 46 51 42 40 35 24 34 20 22 25 11 23 0 0 0 0 0 0 0 0 0 0 0 3 0 0 3

"277_fr1" 0 0 0 0 0 0 0 0 0 0 0 0 0 0 0 0 0 0 0 0 0 0 0 0 0 0 0 0 0 0 0 0 0 0 0 3 0 14 39 16 7 19 4 18 34 35 23 24 39 35 49 43 35 45 48 22 41 43 52 53 43 51 60 55 42 1 0 0 0 15 0 0 0 0 0 0 0 0 0 0 0 0 0 0 0

"2775_fr2" 0 1 2 0 0 1 0 1 0 3 1 0 0 1 0 2 0 0 2 1 3 0 2 2 0 5 1 0 0 3 0 2 3 0 0 0 0 0 0 0 0 0 0 0 0 0 0 0 0 0 0 0 0 0 0 0 0 0 0 0 0 0 0 0 0 0 0 0 0 0 0 0 0 0 0 0 0 0 0 0 0 0 0 0 0

"28400_fr1" 0 2 0 0 3 0 0 0 0 0 0 0 0 0 0 0 0 0 0 0 0 0 0 0 0 0 0 0 0 2 0 0 0 2 0 0 0 0 0 0 0 0 0 0 0 0 0 0 0 0 0 0 0 0 0 0 0 0 0 0 0 0 0 0 0 0 0 0 0 0 0 0 0 0 0 0 0 0 0 0 0 0 0 0 0

"28434_fr5" 0 0 0 0 0 0 0 0 0 0 0 0 0 0 0 0 0 0 0 0 0 0 0 0 0 0 0 0 0 0 0 1 0 0 0 0 0 0 0 0 0 0 0 0 0 0 0 0 0 0 0 0 0 0 0 0 0 0 0 0 0 0 0 0 0 0 0 0 0 0 0 0 0 0 0 0 0 0 0 0 0 0 0 0 0

"2853_fr3" 2 3 3 5 12 4 7 4 16 3 5 3 15 2 8 3 3 4 3 3 4 4 2 9 9 2 1 9 4 3 6 5 2 3 7 0 0 1 0 1 0 0 2 0 0 0 0 0 1 0 0 0 0 0 2 0 0 0 0 0 0 0 0 0 0 0 0 0 0 0 0 0 0 0 0 0 0 0 0 0 0 0 0 0 0

"28559_fr5" 0 0 0 0 0 0 0 0 0 0 0 0 0 0 1 0 0 0 0 0 0 0 0 0 0 0 0 0 0 0 0 0 0 0 0 0 0 0 0 0 0 0 0 0 0 0 0 0 0 0 0 0 0 0 0 0 0 0 0 0 0 0 0 0 0 0 0 0 0 0 0 0 0 0 0 0 0 0 0 0 0 0 0 0 0

"28570_fr3" 0 0 0 0 0 0 0 0 0 0 0 0 0 0 0 0 0 0 0 0 0 0 0 0 0 0 0 0 0 0 0 0 0 0 0 0 0 2 5 0 1 1 6 6 6 5 5 3 6 8 8 11 6 7 8 8 6 6 6 4 5 6 6 6 8 8 6 8 6 7 0 0 0 0 0 0 0 0 0 0 0 0 0 0 0

"286_fr2" 0 0 0 0 0 0 0 0 0 0 0 0 0 0 0 0 0 0 0 0 0 3 0 0 0 0 0 0 0 0 0 0 0 0 0 0 0 0 0 0 0 0 0 0 0 0 0 0 0 0 0 0 0 0 0 0 0 0 0 0 0 0 0 0 0 0 0 0 0 0 0 0 0 0 0 0 0 0 0 0 0 0 0 0 0

"28713_fr5" 0 0 0 0 0 0 0 0 0 0 0 0 0 0 0 0 0 0 0 0 0 0 4 0 0 0 0 0 0 0 0 0 0 0 0 0 0 0 0 0 0 0 0 0 0 0 0 0 0 0 0 0 0 0 0 0 0 0 0 0 0 0 0 0 0 0 0 0 0 0 0 0 0 0 0 0 0 0 0 0 0 0 0 0 0

"2906_fr1" 0 0 0 0 2 0 0 0 0 0 0 0 0 0 0 0 0 0 0 2 2 0 0 0 0 0 0 0 0 0 0 0 0 1 0 0 0 0 0 0 0 0 0 0 0 0 0 0 0 0 0 0 0 0 0 0 0 0 0 0 0 0 0 0 0 0 0 0 0 0 0 0 0 0 0 0 0 0 0 0 0 0 0 0 0

"2916_fr1" 0 0 0 0 0 0 0 0 0 0 0 0 0 0 0 0 0 0 0 2 0 0 0 0 0 0 2 0 0 0 0 0 0 3 0 0 0 0 0 0 0 0 0 0 0 0 0 0 0 0 0 0 0 0 0 0 0 0 0 0 0 0 0 0 0 0 0 0 0 0 0 0 0 0 0 0 0 0 0 0 0 0 0 0 0

"29225_fr1" 0 0 0 0 0 0 0 0 0 0 0 0 0 0 0 0 0 0 0 0 0 0 0 1 0 0 0 0 0 0 0 0 1 0 0 0 0 0 0 0 0 0 0 0 0 0 0 0 0 0 0 0 0 0 0 0 0 0 0 0 0 0 0 0 0 0 0 0 0 0 0 0 0 0 0 0 0 0 0 0 0 0 0 0 0

"29320_fr2" 0 0 0 0 0 0 0 0 0 0 0 1 0 0 0 0 1 0 0 1 2 0 0 0 0 1 1 0 1 1 2 2 0 0 0 0 0 0 0 0 0 0 0 0 0 0 0 0 0 0 0 0 0 0 0 0 0 0 0 0 0 0 0 0 0 0 0 0 0 0 0 0 0 0 0 0 0 0 0 0 0 0 0 0 0

"29593_fr4" 0 0 0 0 0 0 0 0 0 4 0 2 0 0 0 0 0 0 0 0 0 0 0 0 0 3 0 0 0 0 0 0 0 0 0 0 0 0 0 0 0 0 0 0 0 0 0 0 0 0 0 0 0 0 0 0 0 0 0 0 0 0 0 0 0 0 0 0 0 0 0 0 0 0 0 0 0 0 0 0 0 0 0 0 0

"29874_fr6" 6 4 4 5 4 6 3 3 3 3 1 1 0 4 5 4 5 3 4 5 3 5 2 5 3 4 5 1 4 4 2 4 5 4 3 0 1 0 0 1 0 0 0 0 0 0 0 0 0 0 0 0 0 0 0 0 0 0 0 0 0 0 0 0 0 0 0 0 0 0 0 0 0 0 0 0 0 0 0 0 0 0 0 0 0

"2997_fr4" 0 0 0 0 0 0 0 0 0 0 0 0 0 0 0 0 0 0 0 0 2 0 2 0 0 1 0 0 0 0 0 0 0 0 0 0 0 0 0 0 0 0 0 0 0 0 0 0 0 0 0 0 0 0 0 0 0 0 0 0 0 0 0 0 0 0 0 0 0 0 0 0 0 0 0 0 0 0 0 0 0 0 0 0 0

"30189_fr3" 0 0 0 0 0 0 0 0 0 6 0 0 3 0 0 0 0 0 0 0 0 0 0 0 2 0 0 0 0 0 0 0 0 0 0 0 0 0 0 0 0 0 0 0 0 0 0 0 0 0 0 0 0 0 0 0 0 0 0 0 0 0 0 0 0 0 0 0 0 0 0 0 0 0 0 0 0 0 0 0 0 0 0 0 0

"30226_fr1" 2 1 0 0 2 2 3 0 0 0 4 0 0 0 0 0 2 0 0 0 0 2 0 0 0 0 2 2 0 0 0 2 2 0 0 0 0 0 0 0 0 0 0 0 0 0 0 0 0 0 0 0 0 0 0 0 0 0 0 0 0 0 0 0 0 0 0 0 0 0 0 0 0 0 0 0 4 0 3 1 0 0 0 0 0

"30229_fr4" 0 0 0 0 0 0 0 0 0 4 0 0 0 0 0 0 0 0 0 0 0 0 0 0 0 0 0 0 0 0 0 0 0 0 0 0 0 0 0 0 0 0 0 0 0 0 0 0 0 0 0 0 0 0 0 0 0 0 0 0 0 0 0 0 0 0 0 0 0 0 0 0 0 0 0 0 0 0 0 0 0 0 0 0 0

"30247_fr5" 5 8 3 6 8 4 11 9 9 7 6 0 8 4 4 4 5 9 2 5 4 3 8 6 5 4 2 9 3 6 6 8 3 2 4 0 1 4 0 5 0 1 4 0 3 0 4 2 4 4 3 0 0 2 3 0 0 0 0 3 0 0 0 1 0 5 5 4 0 0 4 0 3 3 2 5 4 4 5 1 2 5 9 4 3

"30292_fr1" 0 0 0 0 0 0 0 0 0 0 0 0 0 0 0 0 0 0 0 0 0 0 0 0 0 0 0 0 0 0 0 0 0 0 0 0 0 0 0 0 0 0 0 0 0 0 0 0 0 0 0 0 0 0 0 0 0 0 0 0 0 0 0 0 0 0 0 0 0 0 0 0 0 0 0 0 0 0 8 4 0 0 0 0 0

"30342_fr3" 0 2 3 0 0 0 0 0 0 7 0 0 6 0 0 0 0 4 0 0 1 0 0 0 3 0 0 0 0 0 0 0 0 0 0 0 0 0 0 0 0 0 0 0 0 0 0 0 0 0 0 0 0 0 0 0 0 0 0 0 0 0 0 0 0 0 0 0 0 0 0 0 0 0 0 0 0 0 0 0 0 0 0 0 0

"30353_fr4" 0 0 0 0 0 3 0 0 0 0 0 0 0 0 0 0 0 0 0 0 0 0 5 0 0 4 0 0 0 0 0 0 0 0 0 0 0 0 0 0 0 0 0 0 0 0 0 0 0 0 0 0 0 0 0 0 0 0 0 0 0 0 0 0 0 0 0 0 0 0 0 0 0 0 0 0 0 0 0 0 0 0 0 0 0

"30375_fr6" 4 6 8 4 10 6 11 8 8 1 7 8 6 4 7 8 7 12 0 7 8 0 10 13 17 6 8 0 14 4 11 11 7 0 8 0 0 0 0 0 0 0 0 0 0 0 0 0 0 0 0 0 0 0 0 0 0 0 0 0 0 0 0 0 0 0 0 0 0 0 0 0 0 0 0 3 0 5 5 0 5 4 2 6 4

"30378_fr1" 0 0 0 0 1 0 0 0 0 0 0 0 0 0 0 0 0 0 0 0 0 0 1 0 0 0 0 1 0 5 0 0 0 0 0 0 0 0 0 0 0 0 0 0 0 0 0 0 0 0 0 0 0 0 0 0 0 0 0 0 0 0 0 0 0 0 0 0 0 0 0 0 0 0 0 0 0 0 0 0 0 0 0 0 0

"30412_fr5" 0 4 0 7 0 0 0 4 6 4 4 8 0 0 0 7 0 0 0 4 0 0 0 0 0 0 0 0 5 0 10 0 0 0 0 0 0 0 0 0 0 0 0 0 0 0 0 0 0 0 0 0 0 0 0 0 0 0 0 0 0 0 0 0 0 0 0 0 0 0 0 0 0 0 0 1 0 0 0 0 0 0 7 2 0

"30449_fr6" 2 4 5 4 0 0 8 5 3 2 9 5 3 0 6 4 2 7 0 6 0 2 2 4 0 0 0 0 2 2 8 0 1 0 4 0 0 0 0 0 0 0 0 0 0 0 0 0 0 0 0 0 0 0 0 0 0 0 0 0 0 0 0 0 0 0 0 0 0 0 0 0 0 0 0 0 1 0 0 0 6 6 6 5 5

"30461_fr2" 0 0 0 0 1 2 0 0 0 0 0 0 0 2 0 0 0 0 0 0 3 0 1 0 0 0 0 0 0 3 0 0 0 0 0 0 0 0 0 0 0 0 0 0 0 0 0 0 0 0 0 0 0 0 0 0 0 0 0 0 0 0 0 0 0 0 0 0 0 0 0 0 0 0 0 4 0 0 0 0 3 0 0 0 0

"30486_fr4" 0 0 0 0 0 0 0 0 0 0 0 0 0 0 0 0 0 0 0 1 0 0 0 1 2 0 0 0 0 0 1 0 1 0 0 0 0 0 0 0 0 0 0 0 0 0 0 0 0 0 0 0 0 0 0 0 0 0 0 0 0 0 0 0 0 0 0 0 0 0 0 0 0 0 0 0 0 0 0 0 0 0 0 0 0

"30490_fr1" 0 0 0 0 0 0 0 0 0 0 0 0 0 0 0 0 0 0 0 0 0 0 0 0 0 0 0 0 0 0 0 0 0 0 0 0 5 9 11 10 12 21 5 8 7 8 8 9 6 9 5 10 6 14 12 8 6 5 2 11 7 9 7 6 9 12 13 8 6 16 0 1 0 0 0 0 0 1 0 0 0 0 0 0 1

"30505_fr4" 0 0 0 0 3 0 0 0 0 0 0 0 0 2 0 0 0 0 0 0 0 0 0 0 0 0 0 0 0 0 0 0 0 0 0 0 0 0 0 0 0 0 0 0 0 0 0 0 0 0 0 0 0 0 0 0 0 0 0 0 0 0 0 0 0 0 0 0 0 0 0 0 0 0 0 0 0 0 0 0 0 0 0 0 0

"30611_fr4" 0 12 5 10 0 9 20 16 25 8 0 14 15 12 8 0 0 33 8 15 11 7 0 5 10 9 7 5 9 8 13 2 5 3 11 0 0 0 0 0 0 0 0 0 0 0 0 0 0 0 0 0 0 0 0 0 0 0 0 0 0 0 0 0 0 0 0 0 0 0 0 0 0 0 0 0 0 9 0 4 12 5 17 5 4

"30611_fr6" 0 0 0 0 0 0 0 0 0 0 0 0 0 0 0 0 0 0 0 0 0 0 0 0 0 0 0 0 0 0 0 0 0 0 0 2 0 1 0 0 0 0 0 0 0 0 0 0 0 0 0 0 0 0 0 0 0 0 0 0 0 0 0 0 0 0 0 0 0 0 0 0 0 0 0 0 0 0 0 0 0 0 0 0 0

"30649_fr2" 0 0 0 0 0 0 0 0 0 0 0 0 0 0 0 0 0 0 0 0 0 0 0 0 0 0 0 0 0 0 0 0 0 0 0 7 10 14 24 17 2 24 24 27 30 22 27 31 17 30 23 24 23 28 23 23 26 24 23 23 23 21 21 20 23 11 11 12 12 17 0 0 0 0 0 0 0 2 0 0 1 5 0 0 6

"30755_fr3" 4 2 2 0 2 0 0 0 0 0 0 0 0 0 0 4 2 0 2 0 0 2 0 4 0 3 3 0 0 0 0 0 0 4 0 0 0 0 0 0 0 0 0 0 0 0 0 0 0 0 0 0 0 0 0 0 0 0 0 0 0 0 0 0 0 0 0 0 0 0 0 0 0 0 0 0 0 0 0 0 0 0 0 0 0

"30770_fr5" 0 0 0 0 0 0 0 4 0 0 0 0 0 0 0 0 0 0 0 0 0 0 0 0 0 0 0 0 0 0 0 0 0 0 0 0 0 0 0 0 0 0 0 0 0 0 0 0 0 0 0 0 0 0 0 0 0 0 0 0 0 0 0 0 0 0 0 0 0 0 0 0 0 0 0 0 0 0 0 0 0 0 0 0 0

"30813_fr2" 0 0 0 0 0 2 0 2 3 2 0 0 0 0 0 0 0 0 0 6 0 0 0 0 0 0 0 0 0 0 0 0 2 0 0 0 0 0 0 0 0 0 0 0 0 0 0 0 0 0 0 0 0 0 0 0 0 0 0 0 0 0 0 0 0 0 0 0 0 0 0 0 0 0 0 0 0 0 0 0 0 0 0 0 0

"30841_fr6" 0 0 0 0 0 0 0 0 0 0 0 0 0 0 0 0 1 0 0 0 0 0 0 0 0 0 0 0 0 0 0 0 0 0 0 0 0 0 0 0 0 0 0 0 0 0 0 0 0 0 0 0 0 0 0 0 0 0 0 0 0 0 0 0 0 0 0 0 0 0 0 0 0 0 0 0 0 0 0 0 0 0 0 0 0

"30894_fr1" 1 4 6 1 0 1 1 2 8 0 0 0 0 7 0 5 0 0 0 0 0 0 0 0 0 0 2 0 0 1 0 0 0 0 0 0 0 0 0 7 0 0 2 2 5 0 0 0 0 0 0 0 0 0 5 0 0 0 0 0 0 0 0 0 0 2 1 0 1 0 2 0 1 0 1 0 0 1 0 0 8 0 3 8 4

"30921_fr3" 4 4 2 4 1 4 5 7 6 2 4 0 4 2 8 4 4 4 0 4 3 7 1 5 4 0 0 0 4 1 4 4 6 4 5 0 0 0 0 0 0 0 0 0 0 0 0 0 0 0 0 0 0 0 0 0 0 0 0 0 0 0 0 0 0 0 0 0 0 0 0 0 0 0 0 0 0 2 0 0 0 0 3 1 4

"3093_fr3" 0 0 0 0 0 0 0 0 0 0 0 0 0 0 0 1 0 0 0 0 0 2 1 0 0 0 2 0 0 0 0 3 0 0 0 0 0 0 0 0 0 0 0 0 0 0 0 0 0 0 0 0 0 0 0 0 0 0 0 0 0 0 0 0 0 0 0 0 0 0 0 0 0 0 0 0 0 0 0 0 0 0 0 0 0

"30941_fr4" 0 0 0 0 0 0 0 0 0 0 0 0 0 0 0 0 0 0 0 0 0 0 0 0 0 0 0 0 0 0 0 0 0 0 0 0 0 0 0 0 0 0 0 0 0 0 0 0 0 0 0 0 0 0 0 0 0 0 0 0 0 0 0 0 0 4 4 6 4 0 0 6 0 2 8 0 0 0 0 7 0 3 0 0 0

"30942_fr6" 2 0 0 0 0 0 5 0 0 0 0 0 0 0 0 0 0 0 0 0 0 7 0 0 0 0 0 2 0 2 0 1 0 0 0 0 0 0 0 0 0 0 0 0 0 0 0 0 0 0 0 0 0 0 0 0 0 0 0 0 0 0 0 0 0 0 0 0 0 0 0 0 0 0 0 0 0 0 0 0 0 0 0 0 0

"3100_fr1" 0 0 1 0 0 0 0 0 0 0 0 0 0 0 0 0 0 0 0 0 0 0 0 0 0 0 2 0 0 0 0 0 0 0 0 0 0 0 0 0 0 0 0 0 0 0 0 0 0 0 0 0 0 0 0 0 0 0 0 0 0 0 0 0 0 0 0 0 0 0 0 0 0 0 0 0 0 0 0 0 0 0 0 0 0

"3110_fr2" 0 0 0 0 0 0 0 0 0 0 0 0 0 0 0 0 0 0 0 0 0 0 0 0 0 0 0 0 0 0 0 0 0 0 0 0 0 0 0 0 0 0 0 0 0 0 0 0 0 0 0 0 0 0 0 0 0 0 0 0 0 0 0 0 0 0 0 0 0 0 0 0 0 0 0 0 4 0 0 0 0 0 0 0 0

"31248_fr3" 0 0 3 6 5 0 4 4 6 4 0 1 0 0 4 0 1 6 0 0 0 2 6 4 6 0 0 0 4 0 3 2 3 0 4 0 0 0 0 0 0 0 0 0 0 0 0 0 0 0 0 0 0 0 0 0 0 0 0 0 0 0 0 0 0 0 0 0 0 0 0 0 0 0 0 0 0 0 0 0 0 0 0 0 0

"31408_fr2" 0 0 0 0 0 0 0 0 0 3 0 0 0 0 0 0 0 0 0 0 0 0 0 0 0 0 0 0 0 0 0 0 0 0 0 0 0 0 0 0 0 0 0 0 0 0 0 0 0 0 0 0 0 0 0 0 0 0 0 0 0 0 0 0 0 0 0 0 0 0 0 0 0 0 0 0 0 0 0 0 0 0 0 0 0

"31415_fr2" 0 0 1 0 2 0 3 1 0 0 0 0 0 0 0 0 0 0 0 6 2 0 0 1 0 2 0 0 0 0 0 0 0 0 0 0 0 0 0 0 0 0 0 0 0 0 0 0 0 0 0 0 0 0 0 0 0 0 0 0 0 0 0 0 0 0 0 0 0 0 0 0 0 0 0 0 0 0 0 0 0 0 0 0 0

"3149_fr2" 0 0 0 0 0 0 0 0 0 0 0 0 0 0 0 0 0 0 0 0 0 0 0 0 0 0 0 0 0 0 0 0 0 0 0 0 0 0 0 0 0 0 0 0 0 0 0 0 0 0 0 0 0 0 0 0 0 0 0 0 0 0 0 0 0 0 0 0 0 0 0 0 0 0 0 1 1 6 7 0 4 6 6 2 7

"3149_fr3" 3 4 6 16 8 4 12 11 22 13 11 5 4 9 6 16 4 20 3 5 4 9 4 3 4 8 6 6 5 4 15 3 12 5 5 3 0 0 0 4 0 0 5 0 0 0 0 0 0 0 0 0 0 0 0 0 0 0 0 0 0 0 0 0 0 0 0 0 0 0 0 0 0 0 0 0 0 0 0 0 0 0 0 0 0

"3157_fr1" 1 0 1 2 0 0 2 1 4 0 0 0 1 0 2 2 1 2 0 0 0 2 0 0 3 0 0 0 0 0 3 0 0 0 2 0 0 0 0 0 0 0 0 0 0 0 0 0 0 0 0 0 0 0 0 0 0 0 0 0 0 0 0 0 0 0 0 0 0 0 0 0 0 0 0 0 0 0 0 0 0 0 0 0 0

"31580_fr4" 0 0 0 0 0 0 0 0 0 0 0 0 0 0 0 0 0 0 0 0 4 0 0 0 0 0 0 0 0 0 0 0 0 0 0 0 0 0 0 0 0 0 0 0 0 0 0 0 0 0 0 0 0 0 0 0 0 0 0 0 0 0 0 0 0 0 0 0 0 0 0 0 0 0 0 0 0 0 0 0 0 0 0 0 0

"31746_fr5" 0 0 0 0 0 0 0 0 0 0 0 0 0 0 0 0 0 0 0 0 0 0 0 0 0 0 0 0 0 0 0 0 0 0 0 0 0 24 14 10 13 26 16 35 21 18 15 44 17 48 18 23 14 23 21 15 13 14 19 19 13 9 13 14 17 10 7 6 12 9 3 2 5 3 6 2 6 3 0 2 2 4 0 0 2

"31805_fr5" 0 0 0 0 0 0 0 0 0 0 0 0 0 0 0 0 0 0 0 0 0 0 0 0 0 0 0 7 0 0 0 0 0 0 0 0 0 0 0 0 0 0 0 0 0 0 0 0 0 0 0 0 0 0 0 0 0 0 0 0 0 0 0 0 0 0 0 0 0 0 0 0 0 0 0 0 0 0 0 0 0 0 0 0 0

"31902_fr5" 0 8 9 1 1 4 11 8 4 0 5 15 5 4 15 0 12 4 3 8 6 9 2 4 28 3 3 3 13 2 5 6 0 8 8 0 0 0 0 0 0 0 0 0 0 0 0 0 0 0 0 0 0 0 0 0 0 0 0 0 0 0 0 0 0 0 0 0 0 0 0 0 0 0 0 5 0 0 0 6 0 0 2 0 21

"31948_fr3" 0 0 0 5 0 0 1 0 3 0 0 0 0 0 8 0 2 3 0 0 0 1 0 2 0 0 0 0 2 0 0 0 1 0 6 0 0 0 0 0 0 0 0 0 0 0 0 0 0 0 0 0 0 0 0 0 0 0 0 0 0 0 0 0 0 0 0 0 0 0 0 0 0 0 0 0 0 0 0 0 0 0 0 0 0

"31949_fr6" 0 2 0 3 0 0 2 0 6 0 0 0 0 0 1 2 2 3 0 0 0 2 0 2 0 0 0 2 0 0 2 0 0 0 0 0 0 0 0 0 0 0 0 0 0 0 0 0 0 0 0 0 0 0 0 0 0 0 0 0 0 0 0 0 0 0 0 0 0 0 0 0 0 0 0 0 0 0 0 0 0 0 0 0 0

"32195_fr3" 0 0 6 0 0 6 0 6 3 0 0 0 0 0 0 0 0 0 0 0 0 0 0 0 0 0 6 0 4 0 0 0 0 0 0 0 0 0 0 0 0 0 0 0 0 0 0 0 0 0 0 0 0 0 0 0 0 0 0 0 0 0 0 0 0 0 0 0 0 0 0 0 0 0 0 0 0 0 0 0 0 0 0 0 0

"32234_fr1" 2 0 0 0 4 0 0 0 0 1 2 2 2 2 2 0 4 0 4 2 2 0 4 5 1 3 0 5 0 4 2 0 1 5 3 0 0 0 0 0 0 0 0 0 0 0 0 0 0 0 0 0 0 0 0 0 0 0 0 0 0 0 0 0 0 0 0 0 0 0 0 0 0 0 0 1 1 1 2 2 2 2 1 0 0

"32254_fr1" 0 0 0 0 0 0 0 0 0 0 0 0 0 0 0 0 0 0 0 0 0 0 0 0 0 0 0 0 0 0 0 0 0 0 0 0 0 0 0 0 0 0 0 0 0 0 0 0 0 0 0 0 0 0 0 0 0 0 0 0 0 0 0 0 0 0 0 0 0 0 0 0 0 0 0 0 0 0 0 5 0 0 0 0 0

"3241_fr5" 0 0 0 0 0 0 0 0 0 0 0 0 0 0 0 0 0 0 0 0 0 0 0 3 0 0 0 0 0 0 0 0 0 0 0 0 0 0 0 0 0 0 0 0 0 0 0 0 0 0 0 0 0 0 0 0 0 0 0 0 0 0 0 0 0 0 0 0 0 0 0 0 0 0 0 0 3 0 0 0 0 0 0 0 0

"327_fr3" 0 0 0 0 0 0 0 0 0 0 0 0 0 0 0 0 0 0 0 0 0 0 0 0 0 0 0 0 0 0 0 0 0 0 0 0 0 0 0 0 0 0 0 0 0 0 0 0 2 0 0 0 4 3 0 0 0 0 5 0 0 3 1 1 2 2 0 0 0 0 0 0 0 0 0 0 0 0 0 0 0 0 0 0 0

"32837_fr1" 0 0 0 0 0 0 0 0 0 0 0 0 0 0 0 0 0 0 0 0 0 0 0 0 0 0 0 0 0 0 0 0 0 0 0 0 0 0 0 0 0 0 0 0 3 0 0 0 0 0 0 0 0 0 4 0 0 0 0 0 0 0 0 0 0 0 0 0 0 0 0 0 0 0 0 0 0 0 0 0 0 0 0 0 0

"32861_fr4" 4 6 6 2 4 1 8 5 4 2 0 2 7 5 7 8 6 8 6 6 6 4 6 6 1 6 6 3 4 6 6 4 6 5 3 0 0 0 0 0 0 0 0 0 0 0 0 0 0 0 0 0 0 0 0 0 0 0 0 0 0 0 0 0 0 3 2 2 3 1 5 5 7 4 6 8 6 5 6 3 2 4 2 2 3

"33340_fr1" 0 0 0 0 0 0 0 0 0 0 0 0 0 0 0 0 0 0 0 0 0 0 0 0 0 0 0 0 0 0 1 0 0 0 0 0 0 0 0 0 0 0 0 0 0 0 0 0 0 0 0 0 0 0 0 0 0 0 0 0 0 0 0 0 0 0 0 0 0 0 0 0 0 0 0 0 0 0 0 0 0 0 0 0 0

"33679_fr3" 0 0 0 0 0 0 0 0 0 0 0 0 0 0 0 0 0 0 0 0 0 0 0 0 0 0 0 0 0 0 2 0 0 0 0 0 0 0 0 0 0 0 0 0 0 0 0 0 0 0 0 0 0 0 0 0 0 0 0 0 0 0 0 0 0 0 0 0 0 0 0 0 0 0 0 0 0 0 0 0 0 0 0 0 0

"33730_fr4" 0 0 0 0 0 0 0 0 0 0 0 0 0 0 0 0 0 0 0 0 0 0 0 0 0 0 0 0 0 0 0 0 0 0 0 0 0 0 2 0 0 0 0 0 3 0 0 0 0 0 0 0 0 0 0 0 0 0 0 2 0 0 0 0 0 0 0 0 0 0 0 0 0 0 0 0 0 0 0 0 0 0 0 0 0

"34079_fr5" 33 9 11 19 8 12 11 23 17 11 13 32 24 21 16 31 12 14 5 23 6 15 6 9 21 7 5 8 8 6 16 4 20 10 18 14 3 4 0 5 0 0 5 0 0 0 0 0 0 0 0 0 0 0 0 0 2 0 0 0 0 0 0 0 0 0 0 0 0 0 0 0 8 0 0 6 8 9 18 4 9 8 12 14 19

"34157_fr4" 0 0 0 0 0 0 0 0 0 0 0 0 0 0 0 0 0 0 0 0 0 0 0 0 0 0 0 0 0 0 0 0 0 0 0 0 0 0 0 3 2 0 0 0 0 0 0 0 0 5 0 0 0 0 0 0 4 2 0 0 0 0 0 0 0 0 0 0 0 0 0 0 0 0 0 0 0 0 0 0 0 0 0 0 0

"34339_fr5" 2 2 2 2 2 2 2 2 2 0 2 2 2 2 2 2 2 2 0 2 0 2 0 2 2 0 0 2 2 1 2 0 2 0 2 0 0 0 0 0 0 0 0 0 0 0 0 0 0 0 0 0 0 0 0 0 0 0 0 0 0 0 0 0 0 0 0 0 0 0 0 0 0 0 0 0 0 0 0 0 0 0 0 0 0

"34340_fr6" 0 0 0 0 0 0 0 0 0 0 0 0 0 0 0 0 0 0 0 0 0 0 0 0 0 0 0 0 0 0 0 0 0 0 0 0 0 0 13 0 2 8 5 0 9 2 18 0 0 0 10 2 0 11 0 0 0 0 0 0 0 0 0 0 0 0 0 0 0 0 0 0 0 0 0 0 0 0 0 0 0 0 0 0 0

"345_fr2" 0 0 0 0 0 0 0 0 0 0 0 0 0 0 0 0 0 0 0 0 0 0 0 0 0 0 0 0 0 0 0 0 0 0 0 0 0 0 0 0 0 0 0 0 0 0 0 0 0 0 0 0 0 0 0 0 0 0 0 0 0 0 0 0 0 2 2 5 2 0 2 0 3 3 2 0 0 2 0 2 0 0 2 0 2

"34536_fr6" 0 0 0 0 0 0 0 0 0 0 0 0 0 0 0 0 0 0 0 0 0 0 0 0 0 0 0 0 0 0 0 0 0 0 0 0 0 0 0 0 0 0 0 0 0 0 0 0 0 0 0 0 0 0 0 0 0 0 0 0 0 0 0 0 0 0 0 0 0 0 0 0 0 0 0 0 0 0 0 0 0 0 0 0 3

"3461_fr4" 0 0 0 0 0 0 0 0 0 0 0 0 0 0 0 0 0 0 0 0 0 0 0 0 0 0 0 0 0 0 1 0 0 0 0 0 0 0 0 0 0 0 0 0 0 0 0 0 0 0 0 0 0 0 0 0 0 0 0 0 0 0 0 0 0 0 0 0 0 0 0 0 0 0 0 0 0 0 0 0 0 0 0 0 0

"34610_fr6" 0 0 0 0 0 0 0 0 0 0 0 0 0 0 0 0 0 0 0 0 0 0 0 0 0 0 0 0 0 0 0 0 0 0 0 0 0 0 0 0 0 0 0 0 0 0 0 0 0 0 0 0 0 0 0 0 0 0 0 0 0 0 0 0 0 0 0 0 0 0 0 1 3 0 1 3 0 0 0 0 0 0 0 0 0

"34845_fr5" 13 13 16 18 15 15 8 14 13 13 9 15 11 7 5 17 12 15 13 14 13 14 15 13 14 15 15 15 11 4 15 16 17 17 0 0 0 5 0 0 0 0 0 0 0 0 0 0 0 0 0 0 0 0 0 0 0 0 0 0 0 0 0 0 0 0 0 0 0 0 0 0 0 0 0 0 0 0 0 0 0 0 0 0 0

"34912_fr1" 0 0 0 0 0 0 0 0 0 0 0 0 0 0 0 0 0 0 0 0 0 0 5 0 0 0 0 0 0 0 0 0 0 0 0 0 0 0 0 0 0 0 0 0 0 0 0 0 0 0 0 0 0 0 0 0 0 0 0 0 0 0 0 0 0 0 0 0 0 0 0 0 0 0 0 0 0 0 0 0 0 0 0 0 0

"35354_fr1" 0 0 0 0 3 0 0 0 0 0 0 0 0 2 0 0 0 0 3 4 0 0 6 0 0 0 4 0 3 1 0 0 0 4 0 0 0 0 0 0 0 0 0 0 0 0 0 0 0 0 0 0 0 0 0 0 0 0 0 0 0 0 0 0 0 0 0 0 0 0 0 0 0 0 0 0 0 0 0 0 0 0 0 0 0

"35422_fr5" 6 3 0 0 2 2 5 0 0 0 0 4 2 7 0 0 0 0 3 7 1 0 0 0 0 0 0 0 3 1 0 8 1 0 0 0 0 0 0 3 0 0 0 0 0 0 0 0 0 0 0 0 0 0 0 0 0 0 0 0 0 0 0 0 0 3 0 0 3 0 0 0 0 1 5 1 6 6 2 0 2 2 0 1 2

"35613_fr5" 0 0 0 0 0 0 0 0 0 0 0 0 0 0 0 0 0 0 0 0 0 0 0 0 0 0 0 0 0 0 0 0 0 0 0 1 0 0 0 1 0 0 0 0 0 0 0 0 0 0 0 0 0 0 0 0 0 0 0 0 0 0 0 0 0 0 0 0 0 0 0 0 0 0 0 0 0 0 0 0 0 0 0 0 0

"35858_fr6" 1 16 3 3 0 1 4 1 1 4 0 0 0 2 3 3 1 3 1 0 2 4 1 1 1 0 0 0 1 2 2 0 0 0 4 0 0 0 0 0 0 0 0 0 0 0 0 0 0 0 0 0 0 0 0 0 0 0 0 0 0 0 0 0 0 0 0 0 0 0 0 0 0 0 0 0 0 10 9 0 0 2 0 4 0

"3601_fr6" 0 0 0 0 1 0 0 0 0 0 0 0 0 0 0 0 2 0 0 0 0 2 0 0 0 0 0 0 0 2 0 0 0 0 0 0 0 0 0 0 0 0 0 0 0 0 0 0 0 0 0 0 0 0 0 0 0 0 0 0 0 0 0 0 0 0 0 0 0 0 0 0 0 0 0 0 0 0 0 0 0 0 0 0 0

"3624_fr5" 0 0 0 0 0 0 0 0 0 0 0 0 0 0 0 0 0 0 0 0 0 0 0 0 0 0 0 0 0 0 0 0 0 0 0 0 7 3 9 3 4 1 9 10 10 8 10 12 11 9 10 12 10 11 11 11 11 11 10 11 8 6 6 8 8 5 6 7 7 7 4 5 5 4 4 5 4 0 3 4 2 2 0 2 2

"36242_fr3" 5 12 11 11 17 12 20 15 15 2 15 5 6 2 6 10 8 5 1 13 11 6 3 14 9 1 15 8 1 0 14 16 12 9 14 0 0 0 0 0 0 0 0 0 0 0 0 0 0 0 0 0 0 0 0 0 0 0 0 0 0 0 0 0 0 0 0 0 0 0 0 0 0 0 0 0 0 0 0 0 0 0 0 0 0

"36278_fr6" 0 0 0 0 0 0 0 0 0 0 0 0 0 0 0 0 0 0 0 0 0 0 0 0 0 0 0 0 0 0 0 0 0 0 0 0 0 0 0 0 0 0 0 0 0 0 0 0 0 0 0 0 0 0 0 0 0 0 0 0 0 0 0 0 0 0 2 0 1 0 5 0 0 0 0 0 0 0 0 0 0 5 7 4 0

"3678_fr1" 0 5 2 0 8 0 0 8 6 11 0 0 7 3 0 0 0 3 0 0 6 0 6 4 10 0 0 4 1 3 1 2 0 0 5 0 0 0 0 0 0 0 0 0 0 0 0 0 0 0 0 0 0 0 0 0 0 0 0 0 0 0 0 0 0 0 0 0 0 0 0 0 0 0 0 0 0 0 0 0 0 0 0 0 0

"37031_fr3" 0 0 0 0 0 0 0 0 0 4 0 0 0 0 0 0 0 0 0 0 0 0 0 0 1 0 0 0 0 0 0 0 0 0 0 0 0 0 0 0 0 0 0 0 0 0 0 0 0 0 0 0 0 0 0 0 0 0 0 0 0 0 0 0 0 0 0 0 0 0 0 0 0 0 0 0 0 0 0 0 0 0 0 0 0

"3706_fr1" 0 0 0 0 0 0 0 0 0 0 0 0 0 0 0 0 0 0 0 0 0 0 0 0 0 0 0 0 0 0 0 0 0 0 0 0 0 0 0 0 0 0 0 0 0 0 0 0 0 0 0 0 0 0 0 0 0 0 0 0 0 0 0 0 0 0 0 0 6 2 3 0 3 0 3 2 0 1 2 2 0 0 0 4 3

"37243_fr4" 0 0 0 0 0 0 0 0 0 0 0 0 0 0 0 0 0 0 0 0 0 0 0 0 0 0 0 0 0 0 0 0 0 0 0 0 3 0 0 4 0 0 0 0 0 2 5 0 5 0 4 2 0 2 0 2 0 0 0 0 0 2 3 0 0 0 0 0 0 0 0 0 0 0 0 0 0 0 0 0 0 0 0 0 0

"37255_fr2" 12 10 4 2 9 2 12 2 3 0 5 10 3 1 0 12 13 10 12 5 1 0 15 5 7 1 10 13 2 2 5 6 13 2 7 0 0 0 0 0 0 0 0 0 0 0 0 0 0 0 0 0 0 0 0 0 0 0 0 0 0 0 0 0 0 0 0 0 1 0 6 0 2 0 0 0 1 0 0 1 0 0 0 0 0

"37276_fr5" 0 0 0 0 0 0 0 0 0 0 0 0 0 0 0 0 0 0 0 0 0 0 0 0 0 0 0 0 0 0 0 0 0 0 0 4 0 4 0 8 0 0 9 0 0 0 0 0 0 0 0 0 0 0 0 0 0 0 0 0 0 0 0 0 0 0 0 0 0 0 0 0 0 0 0 0 0 0 0 0 0 0 0 0 0

"37276_fr6" 15 12 30 30 23 17 18 25 28 26 13 9 33 19 27 17 19 36 4 17 11 21 9 26 42 9 3 20 32 16 35 29 24 16 33 0 0 0 0 0 0 0 0 0 0 0 0 0 0 0 0 0 0 0 0 0 0 0 0 0 0 0 0 0 0 0 0 0 0 0 0 0 3 0 0 4 1 17 14 1 11 13 16 11 27

"37308_fr2" 0 0 0 0 5 0 0 0 0 0 0 0 0 0 0 0 0 2 2 0 0 0 0 0 0 0 0 0 0 2 0 0 0 0 0 0 0 0 0 0 0 0 0 0 0 0 0 0 0 0 0 0 0 0 0 0 0 0 0 0 0 0 0 0 0 0 0 0 0 0 0 0 0 0 0 0 2 0 9 0 0 0 0 0 0

"37338_fr3" 0 0 0 0 0 0 0 0 0 0 0 13 0 0 0 0 0 0 0 0 0 0 0 0 0 0 0 0 0 0 0 7 0 0 0 0 0 0 0 4 0 0 0 0 1 0 0 0 0 0 0 0 0 0 0 0 0 0 0 0 0 0 0 0 0 0 0 0 0 0 0 0 0 0 0 0 0 0 0 0 0 0 0 0 0

"37524_fr1" 0 0 0 0 0 0 0 0 1 0 0 5 0 0 0 0 0 0 7 0 0 0 0 0 0 0 0 0 0 0 0 0 0 0 0 0 0 0 0 0 0 0 0 0 0 0 0 0 0 0 0 0 0 0 0 0 0 0 0 0 0 0 0 0 0 0 0 0 0 0 0 0 0 0 0 0 0 0 0 0 0 0 0 0 0

"37597_fr3" 0 0 0 0 0 5 0 0 0 0 0 0 0 0 0 0 0 0 0 0 0 0 0 0 0 0 2 0 0 0 0 0 0 0 0 0 0 0 0 0 0 0 0 0 0 0 0 0 0 0 0 0 0 0 0 0 0 0 0 0 0 0 0 0 0 0 0 0 0 0 0 0 0 0 0 0 0 0 0 0 0 0 0 0 0

"37605_fr1" 0 0 0 0 0 0 0 0 0 0 0 0 4 0 0 0 0 0 0 0 0 0 0 0 0 0 0 0 0 0 0 0 0 0 0 0 0 0 0 0 0 0 0 0 0 0 0 0 0 0 0 0 0 0 0 0 0 0 0 0 0 0 0 0 0 0 0 0 0 0 0 0 0 0 3 0 0 0 0 0 0 0 0 0 0

"3764_fr3" 3 0 1 0 0 0 0 0 0 0 0 0 0 0 2 0 0 0 1 0 0 0 0 0 0 1 0 1 0 0 0 1 0 0 0 0 0 0 0 0 0 0 0 0 0 0 0 0 0 0 0 0 0 0 0 0 0 0 0 0 0 0 0 0 0 0 0 0 0 0 0 0 0 0 0 0 0 0 0 0 0 0 0 0 0

"37670_fr4" 3 4 1 4 6 6 4 4 2 2 2 2 3 5 2 3 2 0 2 2 2 3 3 3 2 3 5 4 7 7 3 0 3 8 2 0 0 0 0 0 0 0 0 0 0 0 0 0 0 0 0 0 0 0 0 0 0 0 0 0 0 0 0 0 0 0 0 0 0 0 0 0 0 0 0 0 2 3 3 0 0 0 2 0 2

"37683_fr2" 0 5 0 0 0 0 0 0 0 0 0 0 0 0 0 0 0 0 1 0 0 0 0 0 0 0 0 0 0 0 1 0 0 0 3 0 0 0 0 0 0 0 0 0 0 0 0 0 0 0 0 0 0 0 0 0 0 0 0 0 0 0 0 0 0 0 0 0 0 0 0 0 0 0 0 0 0 0 0 0 0 0 0 0 0

"37698_fr1" 0 0 0 0 0 0 0 0 0 0 0 0 0 0 0 0 0 0 0 0 0 0 0 0 1 0 0 0 0 0 0 0 0 0 0 0 0 0 0 0 0 0 0 0 0 0 0 0 0 0 0 0 0 0 0 0 0 0 0 0 0 0 0 0 0 0 0 0 0 0 0 0 0 0 0 0 0 0 0 0 0 0 0 0 0

"3774_fr3" 0 0 0 0 0 0 0 0 0 0 0 0 0 0 0 0 0 0 0 0 0 0 3 0 0 0 0 0 0 0 0 0 0 0 0 0 0 0 0 0 0 0 0 0 0 0 0 0 0 0 0 0 0 0 0 0 0 0 0 0 0 0 0 0 0 0 0 0 0 0 5 0 8 0 0 5 10 11 10 0 0 0 6 3 4

"37795_fr5" 0 0 0 0 0 0 0 0 0 0 0 0 0 0 0 0 0 0 0 0 0 0 0 0 0 0 0 0 0 0 0 0 0 0 0 0 0 0 29 0 7 0 12 21 84 16 50 29 47 110 53 63 20 100 52 20 30 32 61 114 10 11 9 8 21 0 0 0 0 0 0 0 0 0 0 0 0 0 0 0 0 0 0 0 2

"38110_fr6" 0 0 0 0 0 0 0 0 0 0 0 0 0 0 0 0 0 0 0 0 0 0 5 0 0 0 0 0 0 0 0 0 0 0 0 0 0 0 0 0 0 0 0 3 0 0 0 0 0 0 0 0 0 0 0 0 0 0 0 0 0 0 0 0 0 0 0 3 0 0 5 0 3 0 9 1 0 0 0 3 0 0 0 0 0

"38187_fr5" 0 0 2 0 2 0 0 0 0 2 0 0 0 0 0 0 1 2 0 0 2 2 1 0 1 0 0 0 2 0 2 2 0 2 2 0 0 0 0 0 0 0 0 0 0 0 0 0 0 0 0 0 0 0 0 0 0 0 0 0 0 0 0 0 0 0 0 0 0 0 0 0 0 0 0 0 0 0 0 0 0 0 0 0 0

"38209_fr1" 0 0 0 0 0 0 0 0 0 0 0 0 0 0 0 0 0 0 0 0 0 0 0 0 0 0 0 0 0 0 0 0 0 0 0 0 0 0 0 0 0 0 0 0 0 0 0 0 0 0 0 0 0 0 0 0 0 0 0 0 0 2 0 3 1 0 0 0 0 0 0 0 0 0 0 0 0 0 0 0 0 0 0 0 0

"38283_fr1" 0 0 0 0 0 0 0 0 0 1 0 0 0 0 0 0 0 0 0 0 0 0 0 0 0 0 0 0 0 0 0 0 0 0 0 0 0 0 0 0 0 0 0 0 0 0 0 0 0 0 0 0 0 0 0 0 0 0 0 0 0 0 0 0 0 0 0 0 0 0 0 0 0 0 0 0 0 0 0 0 0 0 0 0 0

"38577_fr5" 0 0 0 0 0 0 0 0 0 0 0 0 0 0 0 0 0 0 0 0 0 0 0 0 0 0 0 0 0 0 0 0 0 0 0 0 0 0 0 0 0 0 0 0 0 0 0 0 0 0 0 0 0 0 0 0 0 0 0 0 0 0 0 0 2 2 4 2 6 2 0 3 4 6 2 5 8 2 1 4 0 0 0 0 1

"38577_fr6" 2 2 5 0 2 5 2 2 1 0 0 2 0 0 0 1 2 1 0 4 1 2 2 1 2 2 4 0 4 2 2 4 4 1 0 3 0 1 0 2 0 0 0 2 1 1 2 1 0 2 2 0 0 1 1 1 1 2 4 1 0 0 0 0 0 0 0 0 0 0 0 0 0 0 0 0 0 0 0 0 0 0 0 0 0

"38609_fr3" 0 0 2 0 0 0 0 0 0 0 0 0 0 0 0 0 0 0 0 0 0 0 0 0 0 0 0 0 0 0 0 0 0 0 0 0 0 0 0 0 0 0 0 0 0 0 0 0 0 0 0 0 0 0 0 0 0 0 0 0 0 0 0 0 0 0 0 0 0 0 0 0 0 0 0 0 0 0 0 0 0 0 0 0 0

"38765_fr2" 0 0 0 0 2 0 0 0 0 0 0 0 0 0 0 0 0 0 0 0 0 0 6 0 0 0 0 0 0 0 0 0 0 0 0 0 0 0 0 0 0 0 0 0 0 0 0 0 0 0 0 0 0 0 0 0 0 0 0 0 0 0 0 0 0 0 0 0 0 0 0 0 0 0 0 0 0 0 0 0 0 0 0 0 0

"38846_fr3" 0 0 0 0 0 0 0 0 0 0 0 0 0 0 0 0 0 0 0 0 0 0 0 0 0 0 0 0 0 0 0 0 0 0 0 0 0 0 0 0 0 0 0 0 0 0 0 0 0 0 0 0 0 0 0 0 0 0 0 0 0 0 0 0 0 0 0 0 0 0 0 0 0 0 0 0 1 0 0 0 0 0 6 0 0

"39038_fr2" 0 0 0 0 0 0 0 0 0 0 0 0 0 0 0 0 0 0 0 0 0 0 0 0 0 0 0 0 0 0 0 2 0 0 0 0 0 0 0 0 0 0 0 0 0 0 0 0 0 0 0 0 0 0 0 0 0 0 0 0 0 0 0 0 0 0 0 0 0 0 0 0 0 0 0 0 2 0 2 0 0 0 0 0 0

"3913_fr1" 0 0 0 0 0 0 0 0 0 0 0 0 0 0 0 0 0 0 0 0 0 0 0 0 0 0 0 0 0 0 0 0 0 0 0 8 6 12 0 16 0 0 0 0 4 0 6 0 1 2 0 0 0 0 2 0 0 0 3 0 0 0 0 0 0 0 0 0 0 0 0 0 0 0 0 0 0 0 0 0 0 0 0 0 0

"39162_fr2" 13 0 0 0 5 0 6 0 0 7 10 0 5 2 7 2 5 5 8 7 0 0 4 7 0 7 0 3 0 0 7 4 3 2 0 0 0 0 0 0 0 0 0 0 0 0 0 0 0 0 0 0 0 0 0 0 0 0 0 0 0 0 0 0 0 7 8 11 10 3 12 11 10 9 12 9 14 11 14 12 0 9 0 0 0

"39262_fr4" 0 0 0 0 0 0 0 0 0 0 0 0 0 0 0 0 0 0 0 0 0 0 0 0 0 0 0 0 0 0 0 0 0 0 0 0 4 4 3 4 0 4 13 3 4 9 4 2 8 5 3 2 5 2 3 8 4 3 7 3 5 0 3 3 6 3 3 3 2 4 0 0 0 0 0 0 0 0 0 0 0 0 0 0 0

"39357_fr1" 0 0 0 0 0 0 0 0 0 0 0 0 0 0 3 0 0 0 0 0 0 0 0 0 0 0 0 0 0 0 0 0 0 0 0 0 0 0 0 0 0 0 0 0 0 0 0 0 0 0 0 0 0 0 0 0 0 0 0 0 0 0 0 0 0 0 0 0 0 0 0 0 0 0 0 0 0 0 0 0 0 0 0 0 0

"3948_fr4" 0 0 0 1 0 0 0 0 0 0 0 0 0 0 0 0 0 0 0 0 0 0 0 0 0 0 1 0 0 0 0 0 0 0 0 0 0 0 0 0 0 0 0 0 0 0 0 0 0 0 0 0 0 0 0 0 0 0 0 0 0 0 0 0 0 0 0 0 0 0 0 0 0 0 0 0 0 0 0 0 0 0 0 0 0

"39518_fr3" 0 0 0 1 0 2 0 1 0 0 0 0 0 0 3 0 0 0 0 0 0 0 0 1 0 0 0 2 0 0 0 0 0 0 0 0 0 0 0 0 0 0 0 0 0 0 0 0 0 0 0 0 0 0 0 0 0 0 0 0 0 0 0 0 0 0 0 0 0 0 0 0 0 0 0 0 0 0 0 0 0 0 0 0 0

"39606_fr3" 0 0 0 0 0 0 0 0 0 0 0 0 0 0 0 0 0 0 0 0 0 0 0 0 0 0 0 0 0 0 0 0 0 0 0 10 25 34 77 37 52 45 60 64 71 74 78 66 73 77 83 85 76 80 71 82 87 73 93 89 69 72 75 72 76 56 61 55 54 62 42 41 35 40 39 29 34 25 26 26 19 28 11 17 31

"39612_fr1" 0 0 0 0 0 0 0 0 0 0 0 0 0 0 0 0 0 0 0 0 0 0 0 0 0 0 0 0 0 0 0 0 0 0 0 0 0 0 0 0 1 4 0 0 8 0 5 0 0 0 0 0 0 0 6 0 0 0 0 9 0 0 0 0 0 0 0 0 0 0 0 0 0 0 0 0 0 0 0 0 0 0 0 0 0

"39721_fr5" 0 0 0 0 0 0 0 0 0 0 0 3 0 4 0 0 0 0 2 2 0 0 0 0 0 0 0 0 0 0 0 0 0 2 0 0 0 0 0 0 0 0 0 0 0 0 0 0 0 0 0 0 0 0 0 0 0 0 0 0 0 0 0 0 0 0 0 0 0 0 0 0 0 0 0 0 0 0 0 0 0 0 0 0 0

"40028_fr4" 4 0 11 10 6 8 0 7 6 0 4 5 3 4 6 0 3 3 6 1 5 4 6 7 0 0 4 4 7 8 4 8 10 6 3 0 0 0 0 0 0 0 0 0 0 0 0 0 0 0 0 0 0 0 0 0 0 0 0 0 0 0 0 0 0 0 0 0 0 0 0 0 0 0 0 0 0 0 0 0 0 0 0 0 0

"40034_fr4" 0 0 0 0 0 0 0 0 0 0 0 0 0 0 0 0 0 0 0 0 0 0 0 0 0 0 0 0 0 0 0 0 0 0 0 0 0 0 0 0 0 0 0 0 2 0 3 0 0 0 0 0 0 0 1 0 0 0 0 0 0 0 0 0 0 0 0 0 0 0 0 0 0 0 0 0 0 0 0 0 0 0 0 0 0

"40034_fr6" 0 0 3 0 3 0 0 2 1 0 0 0 0 10 2 0 0 0 0 17 0 0 0 0 0 0 0 2 1 0 0 2 0 0 0 0 0 0 0 0 0 0 0 0 0 0 0 0 0 0 0 0 0 0 0 0 0 0 0 0 0 0 0 0 0 0 0 0 0 0 0 0 0 0 1 0 0 13 3 0 0 3 0 0 2

"40044_fr3" 0 0 0 0 0 0 0 0 0 0 0 0 0 0 0 0 0 0 0 0 0 0 0 0 0 0 0 0 0 0 0 0 0 0 0 0 0 0 0 0 0 0 0 2 7 0 3 1 4 9 4 6 0 5 5 0 0 0 5 6 0 0 0 0 0 0 0 0 0 0 0 0 0 0 0 0 0 0 0 0 0 0 0 0 0

"40137_fr6" 0 4 0 0 0 0 0 4 2 0 3 0 5 0 0 0 0 4 0 0 0 0 0 0 0 0 0 2 0 0 0 0 0 0 0 0 0 0 0 0 0 0 0 0 0 0 0 0 0 0 0 0 0 0 0 0 0 0 0 0 0 0 0 0 0 0 0 0 0 0 0 0 0 0 0 0 0 0 0 0 0 0 0 0 0

"4020_fr4" 1 0 0 0 0 0 0 0 0 0 0 1 0 0 0 0 1 0 2 1 0 0 3 0 0 3 1 1 0 8 0 0 0 2 0 0 0 0 0 0 0 0 0 0 0 0 0 0 0 0 0 0 0 0 0 0 0 0 0 0 0 0 0 0 0 0 0 0 0 0 0 0 0 0 0 0 0 0 0 0 0 0 0 0 0

"40218_fr2" 3 1 0 0 1 0 0 0 0 0 1 0 0 0 0 0 0 0 0 1 0 0 2 0 0 1 0 0 0 0 2 2 0 0 2 0 0 0 0 0 0 0 0 0 0 0 0 0 0 0 0 0 0 0 0 0 0 0 0 0 0 0 0 0 0 0 0 0 0 0 0 0 0 0 0 0 0 0 0 0 0 0 2 0 0

"40293_fr4" 12 2 13 21 0 3 4 4 13 19 10 3 1 2 24 7 17 6 0 5 1 21 0 15 31 2 1 0 19 0 24 10 13 11 23 0 0 0 0 0 0 0 0 0 0 0 0 0 0 0 0 0 0 0 0 0 0 0 0 0 0 0 0 0 0 0 0 0 0 0 0 0 0 0 0 0 0 4 1 0 3 16 5 19 19

"40709_fr6" 0 0 0 2 0 0 0 0 0 0 0 0 0 0 0 0 0 0 0 0 0 0 0 0 0 0 0 0 1 0 0 0 0 0 0 0 0 0 0 0 0 0 2 3 0 2 0 0 1 0 1 0 0 0 0 1 0 0 0 0 0 0 0 0 0 0 0 0 0 0 0 0 0 0 0 0 0 0 0 0 0 0 0 0 0

"4071_fr2" 2 0 0 0 3 5 3 2 2 0 4 6 0 3 0 0 4 0 2 3 4 7 5 4 0 3 3 0 2 8 2 2 4 3 0 1 0 2 0 4 0 0 0 4 3 0 0 1 0 1 0 0 0 0 0 0 0 0 0 0 0 0 0 1 0 3 3 3 0 4 4 2 2 1 2 7 6 3 5 4 4 0 3 1 1

"41027_fr6" 0 2 2 0 0 0 2 1 0 2 0 0 2 0 0 0 0 2 0 0 0 0 0 0 2 0 0 0 0 0 0 0 0 0 0 0 0 0 0 0 0 0 0 0 0 0 0 0 0 0 0 0 0 0 0 0 0 0 0 0 0 0 0 0 0 0 0 0 0 0 0 0 0 0 0 0 0 0 0 0 0 0 0 0 0

"41135_fr1" 0 0 0 0 0 0 0 0 0 0 0 0 0 0 0 0 0 0 0 0 0 0 0 0 0 0 0 0 0 0 0 0 0 0 0 0 3 1 0 0 0 0 2 0 0 0 0 0 0 0 0 0 0 0 0 0 0 0 0 0 0 0 0 0 0 0 0 0 0 0 0 0 0 0 0 4 3 0 0 0 0 0 0 0 0

"4123_fr3" 0 0 2 0 0 0 0 1 0 0 0 0 0 0 0 0 0 0 0 0 0 0 0 0 0 0 4 0 0 1 0 8 0 0 0 0 0 8 0 0 0 0 10 0 0 0 0 0 0 0 0 0 0 0 0 0 8 6 0 0 0 0 0 0 0 0 0 3 0 0 1 1 1 0 0 0 2 0 0 0 0 0 0 0 0

"41306_fr6" 0 0 0 4 0 0 0 0 1 0 0 0 0 0 0 0 0 0 0 0 0 0 0 0 0 0 0 0 0 0 1 0 0 0 3 0 0 0 0 0 0 0 0 0 0 0 0 0 0 0 0 0 0 0 0 0 0 0 0 0 0 0 0 0 0 0 0 0 0 0 0 0 0 0 0 0 0 0 0 0 0 0 0 0 0

"4152_fr4" 1 1 0 0 8 5 0 0 0 0 1 3 0 5 0 1 7 0 4 2 0 2 11 3 0 7 8 5 0 10 0 7 6 0 0 0 0 0 0 4 0 0 0 2 0 2 0 0 4 4 0 0 0 6 4 0 2 4 2 4 0 0 0 0 4 4 7 9 6 0 0 5 2 4 4 0 1 0 0 3 0 0 0 0 0

"41523_fr3" 0 4 8 5 4 2 5 4 3 6 4 5 5 0 3 4 4 2 7 1 7 15 14 2 0 6 12 3 7 5 12 2 7 0 8 0 0 2 0 10 0 0 1 0 0 0 0 0 0 0 0 0 0 0 0 0 0 0 0 0 0 0 0 0 0 0 0 0 0 0 0 0 0 0 0 0 0 0 0 0 0 0 0 0 0

"41820_fr2" 0 0 0 0 0 0 0 0 0 0 0 0 0 0 0 1 0 0 0 0 0 0 0 0 0 0 0 0 0 0 0 0 0 0 0 0 0 0 0 0 0 0 0 0 0 0 0 0 0 0 0 0 0 0 0 0 0 0 0 0 0 0 0 0 0 0 0 0 0 0 0 0 0 0 0 0 0 0 0 0 0 0 0 0 0

"41923_fr2" 0 0 0 0 0 0 0 0 0 0 0 0 0 0 0 0 0 0 0 0 0 0 3 0 0 0 0 0 0 0 0 0 0 0 0 0 0 0 0 0 0 0 0 0 0 0 0 0 0 0 0 0 0 0 0 0 0 0 0 0 0 0 0 0 0 0 0 0 0 0 0 0 0 0 0 0 0 0 0 0 0 0 0 0 0

"41933_fr1" 0 0 0 0 0 0 0 0 2 9 0 0 2 0 1 0 0 0 5 0 0 7 0 1 4 0 0 5 3 0 4 1 1 10 2 0 0 0 0 0 0 0 0 0 0 0 0 0 0 0 0 0 0 0 0 0 0 0 0 0 0 0 0 0 0 0 0 0 0 0 0 0 0 0 0 0 0 0 0 0 0 0 9 0 0

"41969_fr5" 2 0 2 2 0 1 2 2 2 2 0 2 0 0 2 2 0 2 0 2 0 2 2 2 2 0 1 0 0 0 2 0 0 2 2 0 0 0 0 0 0 0 0 0 0 0 0 0 0 0 0 0 0 0 0 0 0 0 0 0 0 0 0 0 0 0 0 0 0 0 0 0 0 0 0 0 0 0 0 0 0 0 0 0 0

"4212_fr2" 0 0 3 7 0 1 0 4 0 0 0 0 0 0 0 0 0 0 0 0 0 0 0 0 0 0 5 0 3 0 0 0 0 0 0 0 0 0 0 0 0 0 0 0 0 0 0 0 0 0 0 0 0 0 0 0 0 0 0 0 0 0 0 0 0 0 0 0 0 0 0 0 0 0 0 0 0 0 0 0 0 0 0 0 0

"42143_fr3" 0 0 0 0 0 8 0 0 0 0 0 0 0 5 0 0 5 0 2 2 0 2 0 9 0 2 2 0 0 0 0 0 0 9 0 0 0 0 0 0 0 0 0 0 0 0 0 0 0 0 0 0 0 0 0 0 0 0 0 0 0 0 0 0 0 0 0 0 0 0 0 0 0 0 0 0 0 0 0 0 1 7 0 0 0

"422_fr4" 9 11 8 9 3 11 10 8 8 7 7 9 8 5 9 9 22 11 11 9 4 30 9 7 11 4 8 3 6 5 10 0 6 8 10 7 2 2 0 3 0 0 2 0 0 0 0 0 0 0 0 0 0 0 0 0 0 0 0 0 0 0 0 0 0 0 0 0 0 0 0 0 0 0 0 2 4 17 10 8 6 8 7 7 10

"4222_fr1" 0 0 0 2 0 1 0 0 0 0 0 0 0 0 0 0 0 0 0 0 0 0 0 0 0 0 0 0 0 0 0 0 0 0 0 0 0 0 0 0 0 0 0 0 0 0 0 0 0 0 0 0 0 0 0 0 0 0 0 0 0 0 0 0 0 0 0 0 0 0 0 0 0 0 0 0 0 0 0 0 0 0 0 0 0

"4227_fr2" 6 12 9 9 7 3 6 8 14 4 6 12 7 5 20 13 7 9 5 14 11 12 8 5 16 9 4 5 10 5 17 10 13 10 13 0 0 0 0 0 0 0 0 0 0 0 0 0 0 0 0 0 0 0 0 0 0 0 0 0 0 0 0 0 0 0 0 0 0 0 0 0 0 0 0 0 0 6 4 2 7 6 14 6 11

"42448_fr5" 0 0 0 0 0 0 0 0 0 0 0 0 0 1 0 0 0 0 0 0 0 0 0 0 0 0 0 0 0 0 0 0 0 0 0 0 0 0 0 0 0 0 0 0 0 0 0 0 0 0 0 0 0 0 0 0 0 0 0 0 0 0 0 0 0 0 0 0 0 0 0 0 0 0 0 0 0 0 0 0 0 0 0 0 0

"42505_fr2" 0 0 0 2 0 2 0 2 0 0 0 0 0 0 0 0 0 0 0 0 0 0 0 0 0 0 2 0 2 2 0 0 2 2 0 0 0 0 0 0 0 0 0 0 0 0 0 0 0 0 0 0 0 0 0 0 0 0 0 0 0 0 0 0 0 0 0 0 0 0 0 0 0 0 0 0 0 0 0 0 0 0 0 0 0

"42523_fr4" 0 0 0 0 0 0 0 0 0 0 0 0 0 0 0 0 0 0 0 0 0 0 0 0 0 0 0 0 0 0 0 0 0 0 0 0 0 0 0 1 0 0 2 0 0 0 0 0 0 0 0 0 0 0 0 0 0 3 0 0 0 0 0 0 0 0 0 0 0 0 0 0 0 0 0 0 0 0 0 0 0 0 0 0 0

"42757_fr6" 0 0 0 0 0 0 0 0 0 0 0 0 0 4 0 0 0 0 0 0 0 0 0 0 0 0 0 0 0 0 0 0 0 0 0 0 0 0 0 0 0 0 0 0 0 0 0 0 0 0 0 0 0 0 0 0 0 0 0 0 0 0 0 0 0 0 0 0 0 0 0 0 0 0 0 0 0 0 0 0 0 0 0 0 0

"4277_fr6" 0 0 0 0 0 0 0 0 0 0 0 0 0 0 0 0 2 0 0 0 0 0 0 0 0 0 0 0 0 0 0 0 0 0 0 0 0 0 0 0 0 0 0 0 0 0 0 0 0 0 0 0 0 0 0 0 0 0 0 0 0 0 0 0 0 0 0 0 0 0 0 0 0 0 0 0 0 0 0 0 0 0 0 0 0

"42924_fr4" 0 0 0 0 0 0 0 0 0 0 0 0 0 0 0 0 0 0 0 0 3 0 0 0 0 0 0 0 0 0 0 0 0 0 0 0 0 0 0 0 0 0 0 0 0 0 0 0 0 0 0 0 0 0 0 0 0 0 0 0 0 0 0 0 0 0 0 0 0 0 0 0 0 0 0 0 0 0 3 0 0 0 0 0 0

"42986_fr5" 0 0 0 0 0 0 0 0 0 2 0 0 4 0 0 0 0 2 0 0 0 0 0 0 3 0 0 0 0 0 0 0 0 0 0 0 0 0 0 0 0 0 0 0 0 0 0 0 0 0 0 0 0 0 0 0 0 0 0 0 0 0 0 0 0 0 0 0 0 0 0 0 0 0 0 0 0 0 0 0 0 0 0 0 0

"43139_fr5" 0 0 0 0 1 1 4 1 2 0 0 0 0 0 0 0 0 0 2 1 0 0 0 0 0 0 0 3 0 2 0 0 0 2 0 0 0 0 0 0 0 0 0 0 0 0 0 0 0 0 0 0 0 0 0 0 0 0 0 0 0 0 0 0 0 0 0 0 0 0 0 0 0 0 0 0 0 0 0 0 0 0 0 0 0

"4315_fr5" 0 0 0 0 0 0 0 0 0 0 0 0 0 0 0 0 0 0 1 0 0 0 0 0 0 0 0 0 3 4 0 0 0 0 0 0 0 0 0 0 0 0 0 0 0 0 0 0 0 0 0 0 0 0 0 0 0 0 0 0 0 0 0 0 0 0 0 0 0 0 0 0 0 0 0 0 0 0 0 0 0 0 0 0 0

"43172_fr6" 1 2 4 0 0 3 1 1 0 1 2 2 1 2 1 2 3 0 2 1 2 1 2 2 0 2 1 7 0 5 2 2 2 1 0 0 0 0 0 0 0 0 0 0 0 0 0 0 0 0 0 0 0 0 0 0 0 0 0 0 0 0 0 0 0 0 0 0 0 0 0 0 0 0 0 0 0 0 0 0 0 0 0 0 0

"43196_fr6" 0 3 0 0 0 0 0 0 0 0 1 0 0 0 0 0 0 0 0 1 2 1 0 0 0 0 1 0 2 0 1 2 0 0 0 0 0 0 0 0 0 0 0 0 0 0 0 0 0 0 0 0 0 0 0 0 0 0 0 0 0 0 0 0 0 0 0 0 0 0 0 0 0 0 0 0 0 0 0 0 0 0 0 0 0

"43493_fr2" 0 0 0 0 1 0 0 1 0 0 0 0 0 12 2 0 1 0 1 0 0 0 0 0 0 0 0 0 0 1 0 0 0 6 0 0 0 0 0 0 0 0 0 0 0 0 0 0 0 0 0 0 0 0 0 0 0 0 0 0 0 0 0 0 0 0 0 0 0 0 0 0 0 0 0 0 0 0 0 0 0 0 0 0 0

"43527_fr5" 0 0 0 0 0 0 0 0 0 0 0 0 0 0 0 0 0 0 0 0 0 0 0 0 0 0 0 0 0 0 0 0 0 0 0 0 0 0 0 0 0 0 0 0 0 0 0 0 0 0 0 0 0 0 0 0 6 0 0 0 0 0 0 0 0 0 0 0 0 0 0 0 5 0 0 0 0 0 0 0 0 0 0 0 0

"43739_fr3" 0 0 0 0 0 0 0 0 4 0 0 0 0 0 0 0 0 0 0 0 0 0 0 0 0 0 0 0 0 0 0 0 0 0 0 0 0 0 0 0 0 0 0 0 0 0 0 0 0 0 0 0 0 0 0 0 0 0 0 0 0 0 0 0 0 0 0 0 0 0 0 0 0 0 0 0 0 0 0 0 0 0 0 0 0

"4384_fr5" 0 0 0 0 0 0 0 0 0 0 0 0 0 0 0 0 0 0 0 0 0 0 0 0 0 0 0 0 0 0 0 0 0 0 0 0 0 0 0 0 0 0 0 0 0 0 0 0 0 0 0 0 0 0 0 0 0 0 0 0 0 0 0 0 0 0 0 0 0 0 0 0 0 0 0 1 2 0 1 0 0 0 0 0 0

"439_fr3" 13 11 10 0 15 6 15 4 0 0 1 3 4 0 2 7 9 0 2 4 0 10 0 20 8 5 2 2 2 8 0 5 0 13 0 0 0 0 0 0 0 0 0 0 0 0 0 0 0 0 0 0 0 0 0 0 0 0 0 0 0 0 0 0 0 0 0 0 0 0 0 0 0 0 0 0 0 0 0 0 0 0 0 0 0

"43912_fr3" 13 12 5 0 12 2 5 0 0 0 0 0 2 0 0 8 8 0 6 0 0 8 2 13 0 8 6 7 0 0 0 5 0 5 0 0 0 0 0 0 0 0 0 0 0 0 0 0 0 0 0 0 0 0 0 0 0 0 0 0 0 0 0 0 0 0 0 0 0 0 0 0 0 0 0 0 0 0 0 0 0 0 0 0 0

"43914_fr3" 0 0 0 0 0 0 0 0 0 0 0 0 0 0 0 0 0 0 0 0 0 0 0 0 0 0 0 0 0 0 0 0 1 0 0 0 0 0 0 0 0 0 0 0 0 0 0 0 0 0 0 0 0 0 0 0 0 0 0 0 0 0 0 0 0 0 0 0 0 0 0 0 0 0 0 0 0 0 0 0 0 0 0 0 0

"4401_fr4" 0 6 4 0 0 0 0 0 0 0 0 8 0 1 0 1 0 0 0 0 0 0 0 0 0 0 0 0 0 0 0 0 0 0 0 0 0 0 0 0 0 0 0 0 0 0 0 0 0 0 0 0 0 0 0 0 0 0 0 0 0 0 0 0 0 0 0 0 0 0 0 0 0 0 0 0 0 0 0 0 0 0 0 0 0

"44073_fr1" 0 0 0 0 0 0 0 0 0 0 0 0 0 0 0 0 0 0 0 0 0 0 0 0 0 0 0 0 0 0 0 0 0 0 0 0 0 0 0 0 0 0 0 0 0 0 0 0 0 0 0 0 0 0 0 0 0 0 0 0 0 0 0 0 0 0 0 0 0 0 0 0 0 0 0 4 0 0 0 0 0 0 0 0 0

"441_fr2" 3 1 4 1 6 2 0 2 1 0 2 3 0 3 2 1 0 0 3 2 3 4 5 4 0 2 0 3 4 4 0 2 4 4 2 0 0 0 0 1 0 0 0 0 1 0 0 0 0 0 0 0 0 0 2 0 0 0 0 0 0 0 0 0 0 0 0 0 0 0 0 0 0 0 0 1 1 2 1 0 0 0 1 0 0

"44126_fr2" 0 0 0 10 0 0 8 0 13 1 0 0 0 0 0 0 0 13 0 0 0 0 0 3 16 0 0 0 0 0 2 0 0 0 13 0 0 0 0 0 0 0 0 0 0 0 0 0 0 0 0 0 0 0 0 0 0 0 0 0 0 0 0 0 0 0 0 0 0 0 0 0 0 0 0 0 0 0 0 0 0 0 0 0 0

"44177_fr5" 3 2 0 0 3 4 1 4 0 0 3 2 0 3 0 0 5 0 0 1 0 4 0 3 0 2 3 0 0 3 0 0 0 0 0 0 0 0 0 0 0 0 0 0 0 0 0 0 0 0 0 0 0 0 0 0 0 0 0 0 0 0 0 0 1 0 0 3 3 2 0 0 0 0 5 0 0 0 3 0 1 2 0 2 0

"44186_fr4" 0 0 0 0 0 0 0 0 0 0 0 0 0 0 0 0 0 1 0 0 0 0 0 2 0 0 5 0 0 0 0 0 2 2 2 0 0 0 0 0 0 0 0 0 0 0 0 0 0 0 0 0 0 0 0 0 0 0 0 0 0 0 0 0 0 0 0 0 0 0 0 0 0 0 0 0 0 0 0 0 0 0 0 0 0

"44277_fr2" 0 0 0 0 2 0 0 0 0 0 0 0 0 2 0 0 2 0 1 0 0 0 2 0 0 0 1 0 1 2 0 0 0 0 0 0 0 0 0 0 0 0 0 0 0 0 0 0 0 0 0 0 0 0 0 0 0 0 0 0 0 0 0 0 0 0 3 1 2 0 3 2 0 0 2 1 2 1 0 1 0 0 0 0 0

"44466_fr3" 2 0 0 3 0 3 0 0 4 0 0 0 0 2 0 0 2 0 0 0 0 0 0 0 0 0 0 0 0 0 3 0 0 0 0 0 0 0 0 0 0 0 0 0 0 0 0 0 0 0 0 0 0 0 0 0 0 0 0 0 0 0 0 0 0 0 0 0 0 0 0 0 0 0 0 0 0 0 0 0 0 0 0 0 0

"44520_fr6" 0 6 3 0 2 0 5 6 0 6 2 7 6 0 2 11 0 4 2 2 2 3 5 0 3 2 0 8 1 2 0 3 2 0 0 0 0 0 0 0 0 0 0 0 0 0 0 0 0 0 0 0 0 0 0 0 0 0 0 0 0 0 0 0 0 0 0 0 0 0 0 0 0 0 0 0 0 0 0 0 0 0 0 0 0

"4456_fr5" 0 0 2 4 0 1 0 0 0 0 0 0 0 0 0 0 0 0 0 0 0 0 0 0 0 0 3 0 1 0 0 0 0 0 0 0 0 0 0 0 0 0 0 0 0 0 0 0 0 0 0 0 0 0 0 0 0 0 0 0 0 0 0 0 0 0 0 0 0 0 0 0 0 0 0 0 0 0 0 0 0 0 0 0 0

"44616_fr3" 0 0 0 0 0 0 0 0 0 0 0 0 0 0 0 0 0 0 0 0 0 0 0 0 0 0 0 0 0 0 0 0 0 0 0 0 0 0 0 0 0 0 0 0 0 0 0 0 0 0 0 0 0 0 0 0 0 0 0 0 0 0 0 0 0 0 0 0 0 0 0 0 0 0 0 2 0 0 0 0 0 0 0 0 0

"44693_fr2" 0 3 0 0 0 4 0 0 0 0 0 0 6 0 0 0 0 0 0 0 0 0 0 2 0 0 0 0 0 0 0 0 0 1 0 0 0 0 0 0 0 0 0 0 0 0 0 0 0 0 0 0 0 0 0 0 0 0 0 0 0 0 0 0 0 0 0 0 0 0 0 0 0 0 0 0 0 0 0 0 0 0 0 0 0

"44983_fr2" 0 0 0 3 0 2 0 0 0 0 0 2 0 0 0 0 0 0 0 3 0 0 0 0 0 0 0 0 0 0 0 0 0 0 0 0 0 0 0 0 0 0 0 0 0 0 0 0 0 0 0 0 0 0 0 0 0 0 0 0 0 0 0 0 0 0 0 0 0 0 0 0 0 0 0 0 0 0 0 0 0 0 0 0 0

"45051_fr1" 2 0 0 2 2 2 2 2 2 0 3 0 0 0 0 2 0 0 4 2 4 2 0 0 0 0 3 4 0 8 0 1 2 3 0 0 0 0 0 0 0 0 0 0 0 0 0 0 0 0 0 0 0 0 0 0 0 0 0 0 0 0 0 0 0 0 0 0 0 0 0 0 0 0 0 0 0 0 0 0 0 0 0 0 0

"45104_fr3" 0 0 1 2 0 0 0 1 1 0 0 0 1 0 0 0 0 3 0 1 0 1 1 0 1 0 0 0 0 0 0 0 1 0 1 0 0 0 0 0 0 0 0 0 0 0 0 0 0 0 0 0 0 0 0 0 0 0 0 0 0 0 0 0 0 0 0 0 0 0 0 0 0 0 0 0 0 0 0 0 0 0 0 0 0

"45258_fr3" 0 0 2 0 0 0 0 0 2 0 0 2 1 0 0 0 0 0 2 0 0 0 6 5 2 1 0 1 5 1 2 0 0 3 1 0 0 0 0 0 0 0 0 0 0 0 0 0 0 0 0 0 0 0 0 0 0 0 0 0 0 0 0 0 0 0 0 0 0 0 0 0 0 0 0 0 0 0 0 0 0 0 0 0 0

"45296_fr3" 0 0 0 0 0 0 0 0 0 0 0 3 0 0 0 0 0 0 0 0 0 0 0 0 2 2 0 0 0 0 0 0 0 0 2 0 0 0 0 0 0 0 0 0 0 0 0 0 0 0 0 0 0 0 0 0 0 0 0 0 0 0 0 0 0 0 0 0 0 0 0 0 0 0 0 0 0 0 0 0 0 0 0 0 0

"4531_fr5" 0 0 0 0 1 0 2 0 0 0 0 0 0 0 0 0 0 0 0 0 0 1 0 2 0 4 1 0 0 0 0 1 0 3 0 0 0 0 0 0 0 0 0 0 0 0 0 0 0 0 0 0 0 0 0 0 0 0 0 0 0 0 0 0 0 0 0 0 0 0 0 0 0 0 0 0 0 0 0 0 0 0 0 0 0

"45375_fr4" 0 0 0 0 0 0 0 0 0 3 0 0 0 0 0 0 0 0 0 0 0 0 4 0 0 2 0 0 0 0 2 2 0 0 0 0 0 0 0 0 0 0 0 0 0 0 0 0 0 0 0 0 0 0 0 0 0 0 0 0 0 0 0 0 0 0 0 0 0 0 0 0 0 0 0 0 0 0 0 0 0 0 0 0 0

"45519_fr4" 0 0 0 0 1 0 4 0 0 0 0 0 0 0 0 0 0 0 1 0 0 0 0 8 3 3 4 2 0 2 0 2 0 2 0 0 0 0 0 0 0 0 0 0 0 0 0 0 0 0 0 0 0 0 0 0 0 0 0 0 0 0 0 0 0 0 0 0 0 0 0 0 0 0 0 0 0 0 0 0 0 0 0 0 0

"4553_fr3" 0 0 0 0 0 0 0 0 0 0 0 0 0 0 0 0 0 0 0 0 0 0 0 0 0 0 0 0 0 0 0 0 0 0 0 0 0 0 0 0 0 0 0 0 0 0 0 0 0 0 0 0 0 0 0 0 0 0 0 0 0 0 0 0 0 0 0 0 0 0 0 3 0 0 0 0 0 0 0 0 0 0 0 0 0

"4565_fr1" 5 6 4 14 7 3 6 6 18 2 5 6 4 5 4 5 8 12 0 5 0 2 0 14 4 2 0 5 10 4 8 5 8 4 19 0 0 0 0 0 0 0 0 0 0 0 0 0 0 0 0 0 0 0 0 0 0 0 0 0 0 0 0 0 0 0 0 0 0 0 0 0 0 0 0 0 0 0 0 0 0 0 4 0 4

"45660_fr2" 0 6 4 0 0 0 5 0 0 0 0 0 0 0 4 0 0 6 0 0 0 0 0 0 0 0 3 0 0 0 5 0 0 0 4 0 0 0 0 0 0 0 0 0 0 0 0 0 0 0 0 0 0 0 0 0 0 0 0 0 0 0 0 0 0 0 0 0 0 0 0 0 0 0 0 0 0 0 0 0 0 0 0 0 0

"45864_fr1" 0 0 0 0 0 0 0 0 0 0 0 0 0 0 0 0 0 0 0 0 0 0 0 0 0 0 0 0 0 0 0 0 0 0 0 0 0 0 0 3 0 0 0 0 0 0 0 0 0 0 0 0 0 0 0 0 0 0 0 0 0 0 0 0 0 0 7 4 6 4 4 0 2 6 4 0 6 5 5 4 0 3 4 0 4

"46083_fr3" 4 0 5 0 2 3 4 0 0 0 0 0 4 3 6 0 4 9 4 1 5 4 2 8 6 3 0 0 0 3 0 0 4 6 10 0 0 0 0 4 0 0 2 0 0 0 0 0 0 0 0 0 0 0 0 0 0 0 0 0 0 0 0 0 0 0 0 0 0 0 0 0 0 0 0 0 4 2 4 0 2 3 0 0 6

"461_fr1" 9 6 10 0 11 9 12 7 0 0 5 1 5 0 2 3 9 0 6 2 3 8 4 15 5 7 7 2 6 8 0 7 4 13 0 0 0 0 0 0 0 0 0 0 0 0 0 0 0 0 0 0 0 0 0 0 0 0 0 0 0 0 0 0 0 0 0 0 0 0 0 0 0 0 0 0 0 0 0 0 0 0 0 0 0

"46143_fr1" 0 0 0 0 0 0 0 0 0 0 0 0 0 0 0 0 0 0 0 0 0 0 0 0 0 0 0 0 0 0 0 0 0 0 0 0 0 0 0 0 0 0 0 0 0 0 0 0 0 0 0 0 0 0 0 0 0 0 0 0 0 0 0 0 0 0 0 0 0 0 0 0 0 0 0 3 2 2 1 0 0 0 0 0 0

"46175_fr6" 0 0 0 0 0 0 0 0 0 0 0 0 0 0 0 0 0 0 0 0 0 0 0 0 0 0 0 0 3 0 0 0 0 0 0 0 0 0 0 0 0 0 0 0 0 0 0 0 0 0 0 0 0 0 0 0 0 0 0 0 0 0 0 0 0 0 0 0 0 0 0 0 0 0 0 0 0 0 0 0 0 0 0 0 0

"46249_fr5" 0 0 0 0 0 0 0 0 0 2 0 0 0 0 0 0 0 0 0 0 0 0 0 0 0 0 0 0 0 0 0 0 0 0 0 0 0 0 0 0 0 0 0 0 0 0 0 0 0 0 0 0 0 0 0 0 0 0 0 0 0 0 0 0 0 0 0 0 0 0 0 0 0 0 0 0 0 0 0 0 0 0 0 0 0

"46252_fr2" 1 0 4 0 0 1 2 0 0 0 0 0 1 0 0 0 3 0 0 0 1 3 0 2 1 0 0 1 1 0 0 0 0 5 0 0 0 0 0 0 0 0 0 0 0 0 0 0 0 0 0 0 0 0 0 0 0 0 0 0 0 0 0 0 0 0 0 0 0 0 0 0 0 0 0 0 0 0 0 0 0 0 0 0 0

"46326_fr4" 5 2 2 4 1 3 3 1 3 3 0 3 2 1 4 2 1 4 2 5 1 1 0 2 4 0 1 4 2 2 2 2 2 2 4 0 0 0 0 0 0 0 0 0 0 0 0 0 0 0 0 0 0 0 0 0 0 0 0 0 0 0 0 0 0 0 0 0 0 0 0 0 0 0 0 0 0 0 0 0 0 0 0 0 0

"4635_fr5" 12 5 7 6 5 8 4 5 4 4 4 9 2 6 5 7 6 0 5 11 7 4 6 8 2 5 8 8 11 10 4 11 2 2 1 3 4 4 0 2 0 0 0 0 0 0 0 0 0 0 0 0 0 0 0 0 0 0 0 0 0 0 0 0 0 0 0 0 0 0 0 0 0 0 0 0 0 0 0 0 0 0 0 0 0

"4647_fr6" 2 2 7 7 1 6 2 5 3 0 1 4 1 3 1 2 2 2 0 0 2 3 0 1 1 1 5 1 2 0 3 1 0 3 0 0 0 0 0 0 0 0 0 0 0 0 0 0 0 0 0 0 0 0 0 0 0 0 0 0 0 0 0 0 0 0 0 0 0 0 0 0 0 0 0 0 0 0 0 0 0 0 0 0 0

"46501_fr6" 0 0 0 0 0 0 0 0 0 6 0 0 0 0 0 0 0 6 0 0 0 0 0 0 9 0 0 0 0 0 0 0 0 0 0 0 0 0 0 0 0 0 0 0 0 0 0 0 0 0 0 0 0 0 0 0 0 0 0 0 0 0 0 0 0 0 0 0 0 0 0 0 0 0 0 0 0 0 0 0 0 0 0 0 0

"46567_fr4" 0 0 0 0 0 0 4 0 0 0 0 0 0 0 0 0 0 0 0 0 2 0 0 0 0 0 0 2 0 0 0 1 0 0 0 0 0 0 0 0 0 5 0 0 0 0 0 0 3 0 3 2 0 0 0 0 0 0 0 2 4 1 4 0 3 1 0 0 0 2 0 0 0 0 0 0 0 0 0 0 1 0 0 0 0

"4674_fr2" 0 0 0 0 0 0 0 0 0 0 0 0 0 0 0 0 0 0 0 0 0 0 0 0 0 0 0 0 0 0 0 0 0 0 0 0 0 0 0 0 0 0 0 0 0 0 0 0 0 0 0 0 0 0 0 0 0 0 0 0 0 0 0 0 0 0 0 0 0 0 4 0 0 0 0 0 0 0 0 0 3 2 2 2 0

"46762_fr6" 0 0 0 0 0 0 0 0 0 0 0 5 0 0 0 0 0 0 0 0 0 0 0 0 0 0 0 0 0 0 0 0 0 0 0 0 0 0 0 0 0 0 0 0 0 0 0 0 0 0 0 0 0 0 0 0 0 0 0 0 0 0 0 0 0 0 0 0 0 0 0 0 0 0 0 0 0 0 0 0 0 0 0 0 0

"46841_fr4" 0 0 0 0 0 0 0 0 0 0 0 0 3 0 0 0 0 0 0 0 0 0 0 0 0 0 0 0 0 0 0 0 0 0 0 0 0 0 0 0 0 0 0 0 0 0 0 0 0 0 0 0 0 0 0 0 0 0 0 0 0 0 0 0 0 0 0 0 0 0 0 0 0 0 0 0 0 0 0 0 0 0 0 0 0

"4696_fr3" 0 0 0 0 1 2 0 0 0 0 0 0 0 0 0 0 0 0 0 0 0 0 0 0 0 1 0 0 0 0 0 0 0 0 0 0 0 0 0 2 0 0 0 0 0 0 0 0 0 0 0 0 0 0 0 0 0 0 0 0 0 0 0 0 0 0 0 0 0 0 0 0 0 0 0 0 0 0 0 0 0 0 0 0 0

"47_fr4" 0 0 0 0 0 0 0 0 0 0 0 0 0 0 0 0 0 0 0 0 0 0 0 0 0 0 0 0 0 0 0 0 0 0 0 0 0 0 4 0 0 2 4 4 6 0 4 4 0 5 3 6 0 0 4 0 4 4 5 4 0 0 3 3 0 0 0 0 0 0 0 0 0 0 0 0 0 0 0 0 0 0 0 0 0

"470_fr4" 1 3 12 11 2 9 0 4 3 0 2 6 0 1 4 4 2 0 2 2 4 2 6 5 0 4 13 2 8 3 4 3 3 4 1 0 0 0 0 0 0 0 0 0 0 0 0 0 0 0 0 0 0 0 0 0 0 0 0 0 0 0 0 0 0 0 0 0 0 0 0 0 0 0 0 0 0 1 2 0 1 2 0 1 0

"4729_fr4" 0 0 0 0 0 0 0 0 0 0 0 0 0 0 0 0 0 0 0 0 0 0 0 0 0 0 0 0 0 0 0 0 0 0 0 0 0 0 0 0 0 0 0 0 0 0 0 0 0 0 0 0 0 0 0 0 0 0 0 0 0 0 0 0 0 0 0 0 0 0 0 2 0 1 2 1 3 2 2 2 0 0 0 0 0

"47294_fr1" 15 15 16 16 16 17 9 11 13 9 13 13 9 10 16 12 15 15 14 13 25 15 19 12 17 13 16 9 23 13 17 19 18 17 20 1 3 8 0 6 4 0 3 3 0 3 0 0 0 3 0 3 0 0 1 0 3 4 0 0 0 0 0 0 0 0 0 3 0 0 0 0 4 5 7 6 7 9 9 6 2 8 11 2 10

"47342_fr2" 0 0 0 0 0 0 0 0 0 0 0 0 0 0 0 0 0 0 0 0 0 0 0 0 0 0 0 0 0 0 0 0 0 0 0 0 0 0 0 0 0 0 0 0 0 0 0 0 0 0 0 0 0 0 0 0 0 0 0 0 0 0 0 0 0 4 1 1 2 0 0 0 0 1 0 0 1 1 0 0 1 1 0 0 0

"4737_fr3" 0 0 9 8 0 8 0 8 0 0 8 0 0 0 0 6 7 0 6 9 8 0 9 10 0 8 11 11 7 7 9 8 8 8 0 0 0 0 0 0 0 0 0 0 0 0 0 0 0 0 0 0 0 0 0 0 0 0 0 0 0 0 0 0 0 0 0 0 0 0 0 0 0 0 0 0 0 0 0 0 0 0 0 0 0

"47389_fr4" 0 0 0 0 4 0 0 0 0 0 0 0 0 0 0 0 0 0 0 0 0 0 0 0 0 0 0 0 0 0 0 0 0 0 0 0 0 0 0 0 0 0 0 0 0 0 0 0 0 0 0 0 0 0 0 0 0 0 0 0 0 0 0 0 0 0 0 0 0 0 0 0 0 0 0 0 0 0 0 0 0 0 0 0 0

"4741_fr1" 0 0 0 0 0 0 0 0 0 0 0 0 0 2 0 0 0 0 3 0 1 1 0 0 0 0 0 0 0 2 0 0 0 0 0 0 0 0 0 0 0 0 0 0 0 0 0 0 0 0 0 0 0 0 0 0 0 0 0 0 0 0 0 0 0 0 0 0 0 0 0 0 0 0 0 0 0 0 0 0 0 0 0 0 0

"47426_fr4" 0 0 0 0 0 0 0 0 0 4 0 0 0 0 0 0 0 0 0 0 0 0 0 0 0 0 0 0 0 0 0 0 0 0 0 0 0 0 0 0 0 0 0 0 0 0 0 0 0 0 0 0 0 0 0 0 0 0 0 0 0 0 0 0 0 0 0 0 0 0 0 0 0 0 0 0 0 0 0 0 0 0 0 0 0

"47446_fr5" 5 0 0 0 3 0 0 0 0 0 0 0 0 0 0 0 0 0 0 0 0 2 0 0 0 0 0 0 0 0 0 2 0 0 0 0 0 0 0 0 0 0 0 0 0 0 0 0 0 0 0 0 0 0 0 0 0 0 0 0 0 0 0 0 0 0 0 0 0 0 0 0 0 0 0 0 0 0 0 0 0 0 0 0 0

"4746_fr4" 0 0 0 0 0 0 0 0 1 0 0 0 0 0 0 0 0 3 0 0 0 2 0 1 0 0 0 0 0 0 0 0 0 0 2 0 0 0 0 0 0 0 0 0 0 0 0 0 0 0 0 0 0 0 0 0 0 0 0 0 0 0 0 0 0 0 0 0 0 0 0 0 0 0 0 0 0 0 0 0 0 0 0 0 0

"47479_fr5" 1 0 0 1 0 1 4 4 0 0 0 0 0 1 0 2 0 0 3 2 4 1 2 0 1 1 4 0 0 0 2 1 0 4 0 0 1 0 0 0 0 0 3 0 0 0 0 0 1 0 0 0 0 1 1 0 0 2 1 3 0 0 0 0 0 0 0 0 0 0 0 0 0 0 0 0 0 0 0 0 0 0 0 0 0

"4757_fr2" 0 0 0 0 0 0 0 0 0 3 0 0 4 0 0 0 0 4 0 0 0 0 0 0 0 0 0 0 0 0 0 0 0 0 0 0 0 0 0 0 0 0 0 0 0 0 0 0 0 0 0 0 0 0 0 0 0 0 0 0 0 0 0 0 0 0 0 0 0 0 0 0 0 0 0 0 0 0 0 0 0 0 0 0 0

"47577_fr4" 0 0 0 0 0 0 0 0 0 0 0 0 0 0 0 0 0 0 0 0 0 0 0 0 0 0 0 0 0 0 0 0 0 0 0 0 0 0 0 0 0 0 0 0 1 3 0 0 0 0 0 0 0 3 0 0 0 0 0 1 0 0 0 0 0 0 0 0 0 0 0 0 0 0 0 0 0 0 0 0 0 0 0 0 0

"47680_fr4" 1 2 2 0 1 0 2 2 1 3 2 0 2 0 2 0 1 2 1 1 1 1 2 3 1 2 1 3 2 2 1 2 0 1 0 0 0 0 0 0 0 0 0 0 0 0 0 0 0 0 0 0 0 0 0 0 0 0 0 0 0 0 0 0 0 0 0 0 0 0 0 0 0 0 0 0 0 0 0 0 0 0 0 0 0

"47690_fr4" 0 0 0 0 0 0 3 2 3 0 0 0 1 0 0 0 0 3 0 0 0 0 0 2 0 0 0 0 2 0 1 0 2 0 0 0 0 0 0 0 0 0 0 0 0 0 0 0 0 0 0 0 0 0 0 0 0 0 0 0 0 0 0 0 0 0 0 0 0 0 0 0 0 0 0 0 0 0 0 0 0 0 3 0 0

"47943_fr4" 0 2 6 0 4 0 0 0 0 0 3 5 0 4 6 0 3 0 3 3 5 4 5 4 0 4 4 2 4 4 4 6 6 3 3 0 0 0 0 0 0 0 0 0 0 0 0 0 0 0 0 0 0 0 0 0 0 0 0 0 0 0 0 0 0 0 0 0 0 0 0 0 0 0 0 0 0 0 0 0 0 0 0 0 0

"47948_fr6" 10 2 8 0 4 2 3 2 2 10 2 10 15 4 2 2 12 12 6 2 2 11 2 3 4 4 0 17 2 6 2 6 2 4 0 6 6 9 0 9 1 0 2 6 9 7 6 0 6 4 6 0 0 6 8 0 0 8 5 6 0 6 0 0 0 8 6 6 0 0 0 0 2 4 0 0 0 2 2 8 12 5 2 4 0

"47954_fr3" 0 0 0 0 0 0 0 0 0 0 0 0 0 0 0 0 0 0 0 2 0 0 0 0 0 0 0 0 0 5 0 0 0 0 0 0 0 0 0 0 0 0 0 0 0 0 0 0 0 0 0 0 0 0 0 0 0 0 0 0 0 0 0 0 0 0 0 0 0 0 0 0 0 0 0 0 0 0 0 0 0 0 0 0 0

"47963_fr2" 0 0 0 0 0 0 0 0 0 0 0 0 0 0 0 0 0 0 0 0 0 0 0 0 0 0 0 0 0 0 0 0 0 0 0 0 0 0 0 0 0 0 0 0 0 0 0 0 0 0 0 0 0 0 0 0 0 0 0 0 0 0 0 0 0 0 0 0 0 0 0 0 0 0 0 0 0 0 0 0 0 0 3 2 4

"47965_fr6" 2 3 2 2 2 2 3 3 2 3 2 3 4 2 3 2 2 3 2 2 2 2 2 3 3 2 2 4 2 2 2 4 2 4 2 0 0 0 0 0 0 0 0 0 0 0 0 0 0 0 0 0 0 0 0 0 0 0 0 0 0 0 0 0 0 0 0 0 0 0 0 0 0 0 0 0 0 0 0 0 0 0 0 0 0

"48103_fr2" 0 0 0 0 1 0 1 0 0 0 1 0 3 0 0 0 0 0 0 0 0 0 0 0 0 0 0 0 0 0 0 0 0 0 0 0 0 0 0 0 0 0 0 0 0 0 0 0 0 0 0 0 0 0 0 0 0 0 0 0 0 0 0 0 0 0 0 0 0 0 0 0 0 0 0 0 0 0 0 0 0 0 0 0 0

"48119_fr1" 0 3 4 2 2 2 3 4 2 4 2 1 3 0 1 4 2 3 2 3 3 2 4 4 3 0 4 1 4 5 3 4 2 3 3 0 0 0 0 0 0 0 0 0 0 0 0 0 0 0 0 0 0 0 0 0 0 0 0 0 0 0 0 0 0 0 0 0 0 0 0 0 0 0 0 0 0 0 0 0 0 0 0 0 0

"48166_fr4" 0 0 0 0 0 3 0 5 0 0 0 0 0 0 0 0 0 0 0 0 0 0 0 0 0 0 0 0 2 0 0 0 0 0 4 0 0 0 1 0 0 1 0 0 3 0 0 0 0 6 1 0 0 0 3 0 0 0 0 2 0 4 0 0 0 0 0 0 0 2 0 1 0 3 2 0 3 0 3 0 4 3 3 0 4

"48222_fr4" 0 2 2 0 2 0 5 4 3 0 1 0 4 0 3 0 0 4 0 1 4 0 2 0 3 1 0 4 1 0 0 5 1 0 2 0 0 0 0 0 0 0 0 0 0 0 0 0 0 0 0 0 0 0 0 0 0 0 0 0 0 0 0 0 0 0 0 0 0 0 0 0 0 0 0 0 0 0 0 0 0 0 0 0 0

"4825_fr6" 0 0 0 0 0 0 0 0 0 0 0 0 0 0 0 0 0 0 0 0 0 0 0 0 0 0 0 0 0 0 0 0 0 0 3 0 0 0 0 0 0 0 0 0 0 0 0 0 0 0 0 0 0 0 0 0 0 0 0 0 0 0 0 0 0 0 0 0 0 0 0 0 0 0 0 0 0 0 0 0 0 0 0 0 0

"48266_fr3" 0 4 1 3 3 4 4 4 4 2 3 4 3 1 6 1 2 3 2 3 3 3 4 3 2 1 1 5 5 3 2 4 0 4 4 0 0 0 0 3 0 1 0 0 2 0 1 0 0 2 0 0 0 0 1 0 0 2 1 2 0 2 0 0 0 2 0 0 0 0 0 0 2 0 0 1 1 0 0 1 3 2 3 2 3

"48395_fr6" 0 0 0 0 0 0 0 0 0 0 0 0 0 0 0 0 0 0 0 0 0 0 0 0 0 0 0 0 0 0 0 0 0 0 0 0 0 0 0 0 0 0 0 0 0 0 0 0 0 2 1 0 0 0 2 0 0 0 3 1 0 0 0 0 0 0 0 0 0 4 0 0 0 0 0 0 0 0 0 0 0 0 0 0 0

"48484_fr2" 0 7 2 4 5 0 3 1 3 1 2 1 3 0 2 3 4 4 3 2 5 5 2 4 2 2 2 6 0 4 4 2 2 2 1 0 0 0 0 3 2 0 1 0 0 0 3 0 1 0 0 0 0 0 0 0 0 1 0 0 0 0 0 0 0 3 3 3 0 0 0 0 0 0 0 0 0 3 0 0 0 0 0 3 0

"48631_fr4" 0 0 0 0 0 0 0 0 0 0 0 2 1 5 1 0 1 0 0 0 2 0 0 0 0 0 0 0 0 0 1 1 0 0 0 0 0 0 0 0 0 0 0 0 0 0 0 0 0 0 0 0 0 0 0 0 0 0 0 0 0 0 0 0 0 0 0 0 0 0 0 0 0 0 0 0 0 0 0 0 0 0 0 0 0

"48693_fr3" 9 12 12 14 6 9 11 13 11 6 13 8 10 9 14 12 13 13 8 9 8 15 6 11 11 6 2 7 13 5 11 6 8 6 20 0 0 0 0 0 0 0 0 0 0 0 0 0 0 0 0 0 0 0 0 0 0 0 0 0 0 0 0 0 0 0 0 0 0 0 0 0 0 0 0 3 4 5 10 2 1 3 12 6 9

"48717_fr1" 0 0 0 0 0 0 0 0 0 0 0 0 0 0 0 0 0 0 0 0 0 0 0 0 0 0 0 11 0 1 0 0 0 1 0 0 0 0 0 0 0 0 0 0 0 0 0 0 0 0 0 0 0 0 0 0 0 0 0 0 0 0 0 0 0 0 0 0 0 0 0 0 0 0 0 0 0 0 0 0 0 0 0 0 0

"48739_fr4" 0 1 1 1 0 0 0 0 0 2 0 0 0 0 1 0 0 0 0 0 1 0 0 0 2 0 0 0 0 0 0 0 1 0 2 0 0 0 0 0 0 0 0 0 0 0 0 0 0 0 0 0 0 0 0 0 0 0 0 0 0 0 0 0 0 0 0 0 0 0 0 0 0 0 0 0 0 0 0 0 0 2 2 0 1

"48741_fr3" 0 0 0 0 0 0 0 2 0 10 0 1 2 0 0 0 0 0 0 0 1 0 0 0 0 0 0 1 0 1 0 0 0 0 0 0 0 0 0 0 0 0 0 0 0 0 0 0 0 0 0 0 0 0 0 0 0 0 0 0 0 0 0 0 0 0 0 0 0 0 0 0 0 0 0 0 0 0 0 0 0 0 0 0 0

"48760_fr1" 2 19 2 21 20 24 31 28 26 25 27 21 32 0 18 22 6 9 18 0 26 5 6 5 15 4 5 32 19 4 5 1 18 24 9 0 0 0 0 0 0 0 0 0 0 0 0 0 0 0 0 0 0 0 0 0 0 0 0 0 0 0 0 0 0 5 0 0 0 0 0 0 0 0 0 0 0 0 0 15 21 0 21 20 24

"48769_fr1" 0 3 0 0 0 0 2 0 0 0 0 0 3 0 1 0 0 0 0 0 0 0 0 0 0 0 0 0 0 0 0 0 0 0 0 0 0 0 0 0 0 0 0 0 0 0 0 0 0 0 0 0 0 0 0 0 0 0 0 0 0 0 0 0 0 0 0 0 0 0 0 0 0 0 0 0 0 0 0 0 0 0 0 0 0

"48770_fr4" 0 0 0 0 0 0 0 0 0 0 0 0 0 0 0 0 0 0 0 0 0 0 0 0 0 0 0 0 0 0 0 0 0 0 0 0 0 0 0 0 0 3 0 0 0 0 0 0 0 0 0 1 1 0 0 0 0 0 0 0 0 0 0 0 0 0 0 0 0 0 0 0 0 0 0 0 0 0 0 0 0 0 0 0 0

"48779_fr4" 0 2 4 5 4 2 2 1 2 3 0 2 0 1 5 2 2 1 5 1 3 3 3 2 6 3 3 0 1 2 2 1 0 4 6 0 0 0 0 0 0 0 0 0 0 0 0 0 0 0 0 0 0 0 0 0 0 0 0 0 0 0 0 0 0 0 0 0 0 0 0 0 0 0 0 0 0 0 0 0 0 0 0 0 0

"4879_fr4" 2 2 2 0 2 3 0 2 1 2 0 7 2 2 2 2 1 1 2 2 2 1 2 2 0 2 2 2 2 2 2 3 2 2 2 0 0 0 0 0 0 0 0 0 0 0 0 0 0 0 0 0 0 0 0 0 0 0 0 0 0 0 0 0 0 0 0 0 0 0 0 0 0 0 0 0 0 0 0 0 0 0 0 0 0

"48828_fr2" 3 2 2 4 2 2 3 3 0 4 2 2 2 2 3 2 2 4 2 2 2 3 2 2 3 2 2 2 2 2 3 2 2 2 4 3 1 2 0 2 0 0 2 2 0 0 4 0 0 0 4 0 0 0 4 0 2 0 0 0 4 4 4 4 0 0 0 0 0 0 4 0 2 2 1 2 2 2 2 2 2 1 2 2 0

"48864_fr1" 0 0 0 0 0 0 0 0 3 0 1 0 0 0 0 0 0 0 0 0 0 1 0 0 1 0 0 0 0 0 0 0 0 0 0 0 0 0 0 0 0 0 0 0 0 0 0 0 0 0 0 0 0 0 0 0 0 0 0 0 0 0 0 0 0 0 0 0 0 0 0 0 0 0 0 0 0 0 0 0 0 0 0 0 0

"48868_fr5" 4 6 4 5 5 5 4 4 5 4 5 4 4 4 4 4 5 4 5 5 4 5 5 4 3 6 4 7 5 4 2 4 4 6 4 4 4 2 2 4 0 2 3 2 2 2 2 2 2 2 2 2 2 2 3 2 2 2 2 0 2 4 2 2 0 6 2 4 3 2 2 2 2 2 2 2 2 2 2 2 6 2 2 2 2

"48880_fr4" 0 0 0 0 0 0 0 0 0 0 0 0 0 0 0 0 0 0 0 0 0 0 0 0 0 0 0 0 0 0 0 0 0 0 0 0 0 0 0 0 0 0 0 0 0 0 0 0 0 0 0 0 0 0 0 0 0 0 0 0 0 0 0 0 0 0 0 0 0 0 8 0 0 0 0 0 3 0 0 0 0 0 0 0 0

"48901_fr2" 3 0 3 0 7 3 4 0 2 0 0 1 0 0 0 7 0 0 5 0 0 4 0 0 0 1 7 0 4 2 0 4 0 3 2 0 0 2 0 9 0 0 0 0 0 0 0 0 0 1 0 0 0 0 0 0 0 0 0 0 0 0 0 0 4 6 7 7 6 7 5 6 10 8 7 14 11 7 3 10 3 7 0 4 4

"49115_fr1" 1 0 0 1 0 0 0 0 1 0 0 0 1 1 0 0 0 0 2 0 0 1 0 0 0 0 0 0 0 0 0 0 1 2 0 0 0 0 0 0 0 0 0 0 0 0 0 0 0 0 0 0 0 0 0 0 0 0 0 0 0 0 0 0 0 0 0 0 0 0 0 0 0 0 0 0 0 0 0 0 0 0 0 0 0

"49126_fr2" 0 1 1 4 0 0 0 1 10 1 0 0 1 0 0 0 0 1 0 0 0 0 0 0 0 0 0 0 0 0 0 0 0 1 3 0 0 0 0 0 0 0 0 0 0 0 0 0 0 0 0 0 0 0 0 0 0 0 0 0 0 0 0 0 0 0 0 0 0 0 0 0 0 0 0 0 0 1 1 0 0 0 2 0 4

"49153_fr2" 0 0 0 0 0 0 0 0 0 0 0 2 0 0 0 0 0 0 0 0 0 0 0 0 0 0 0 0 0 0 0 0 0 0 0 0 0 0 0 0 0 0 0 0 0 0 0 0 0 0 0 0 0 0 0 0 0 0 0 0 0 0 0 0 0 0 0 0 0 0 0 0 0 0 0 0 0 0 0 0 0 0 0 0 0

"49231_fr6" 0 0 0 0 0 0 0 0 0 0 0 0 0 0 0 0 0 0 0 0 0 0 0 0 0 0 0 0 0 0 0 0 0 0 0 0 0 0 0 0 0 0 0 0 0 0 0 0 0 0 0 0 0 0 0 0 0 0 0 0 0 0 0 0 0 2 1 1 0 0 0 0 0 0 1 2 2 0 0 2 0 0 1 0 0

"49244_fr3" 5 6 6 6 9 9 5 6 6 1 6 7 5 22 11 7 8 7 9 9 7 6 8 8 3 11 6 7 8 11 6 5 8 7 12 0 0 3 0 1 0 0 1 2 0 0 0 0 3 0 2 0 4 5 1 0 0 2 3 0 0 0 0 0 0 0 0 0 0 0 0 0 0 2 2 2 4 5 4 2 3 4 1 3 4

"49275_fr3" 0 0 0 0 0 0 0 0 0 0 0 0 0 0 0 0 0 0 0 0 0 0 0 0 0 0 0 0 0 0 0 0 0 0 0 3 1 4 0 0 0 0 1 3 3 0 4 3 0 2 3 3 0 2 2 0 2 2 4 0 0 0 0 0 0 0 0 0 0 0 0 0 0 0 0 0 0 0 0 0 0 0 0 0 0

"49276_fr4" 13 5 19 5 2 4 7 5 9 3 4 1 8 0 2 7 0 22 3 3 5 19 17 15 3 21 14 7 3 9 17 1 5 7 20 0 3 0 0 15 1 0 0 0 0 0 0 0 0 0 0 0 0 0 0 0 0 0 0 0 0 1 0 0 0 13 3 8 0 0 0 2 0 12 1 4 1 2 1 3 4 17 2 1 3

"49285_fr6" 1 0 1 0 0 0 0 0 0 0 0 0 0 0 6 0 1 1 0 0 0 0 0 0 0 0 0 0 0 0 1 0 0 0 0 0 0 0 0 0 0 0 0 0 0 0 0 0 0 0 0 0 0 0 0 0 0 0 0 0 0 0 0 0 0 0 0 0 0 0 0 0 0 0 0 0 0 0 0 0 0 0 0 0 0

"4935_fr3" 0 0 0 0 1 0 0 0 0 0 0 1 0 0 0 0 1 0 0 0 0 0 1 0 0 0 0 0 0 3 0 0 1 0 0 0 0 0 0 0 0 0 0 0 0 0 0 0 0 0 0 0 0 0 0 0 0 0 0 0 0 0 0 0 0 0 0 0 0 0 0 0 0 0 0 0 0 0 0 0 0 0 0 0 0

"49678_fr2" 0 0 0 0 0 0 0 0 0 16 0 0 4 0 0 0 0 0 0 0 0 0 0 0 8 0 0 0 0 0 0 0 0 0 0 0 0 0 0 0 0 0 0 0 0 0 0 0 0 0 0 0 0 0 0 0 0 0 0 0 0 0 0 0 0 0 0 0 0 0 0 0 0 0 0 0 0 0 0 0 0 0 0 0 0

"49680_fr4" 0 2 1 0 0 0 2 0 0 4 0 0 4 0 0 0 0 6 0 0 0 0 0 0 4 0 0 0 0 0 0 0 0 0 0 0 0 0 0 0 0 0 0 0 0 0 0 0 0 0 0 0 0 0 0 0 0 0 0 0 0 0 0 0 0 0 0 0 0 0 0 0 0 0 0 0 0 0 0 0 0 0 0 0 0

"49809_fr1" 0 0 0 0 0 0 0 0 0 1 0 0 9 0 0 0 0 0 0 0 0 0 0 0 0 0 0 0 0 0 0 0 0 0 0 0 0 0 0 0 0 0 0 0 0 0 0 0 0 0 0 0 0 0 0 0 0 0 0 0 0 0 0 0 0 0 0 0 0 0 0 0 0 0 0 0 0 0 0 0 0 0 0 0 0

"5003_fr4" 0 0 0 0 0 0 0 0 0 0 0 0 0 0 0 0 0 0 0 0 0 0 0 0 0 0 0 0 0 0 0 0 0 0 0 0 0 0 0 0 0 0 0 0 0 0 0 3 0 0 0 5 0 0 4 0 0 0 0 0 0 0 0 0 0 0 0 0 0 0 0 0 0 0 0 0 0 0 0 0 0 0 0 0 0

"50392_fr3" 0 0 0 0 0 0 0 0 0 0 0 0 0 3 0 0 0 1 0 0 4 0 0 0 0 0 0 0 0 0 0 0 0 0 0 0 0 0 0 0 0 0 0 0 0 0 0 0 0 0 0 0 0 0 0 0 0 0 0 0 0 0 0 0 0 0 0 0 0 0 0 0 0 0 0 0 0 0 0 0 2 0 0 0 0

"50412_fr1" 0 0 0 0 0 0 0 0 0 0 0 0 0 0 0 2 0 0 0 0 0 0 0 0 0 0 2 0 2 0 0 0 0 0 0 0 0 0 0 0 0 0 0 0 0 0 0 0 0 0 0 0 0 0 0 0 0 0 0 0 0 0 0 0 0 0 0 0 0 0 0 0 0 0 0 0 0 0 0 0 0 0 0 0 0

"50440_fr1" 3 7 2 8 0 7 7 8 9 1 5 2 3 0 12 7 1 4 0 7 6 1 4 8 0 8 6 2 7 2 7 1 8 14 8 0 0 0 0 0 0 0 0 0 0 0 0 0 0 0 0 0 0 0 0 0 0 0 0 0 0 0 0 0 0 0 0 0 0 0 0 0 0 0 0 0 0 0 0 0 0 0 0 0 0

"50478_fr6" 0 0 0 0 0 0 0 0 0 0 0 0 0 0 0 0 0 0 0 0 0 0 0 0 0 0 0 0 0 0 0 0 0 0 0 0 0 0 0 5 0 0 0 0 0 0 0 0 0 0 0 0 0 0 0 0 0 0 0 0 0 0 0 0 0 0 0 0 0 0 0 0 0 0 0 0 0 0 0 0 0 0 0 0 0

"50488_fr6" 0 0 0 0 0 0 0 0 0 0 0 0 0 0 0 0 0 0 0 0 0 0 0 0 0 0 0 0 0 0 0 0 0 0 0 0 0 10 31 7 17 7 33 18 20 19 17 14 27 22 18 17 21 24 17 21 24 14 14 20 31 12 44 13 18 17 12 9 26 16 0 8 1 0 3 0 0 1 0 0 0 1 0 0 5

"50566_fr5" 0 2 0 0 2 3 0 0 0 0 0 0 0 0 0 0 2 0 5 0 0 0 0 0 0 0 0 0 0 0 0 2 0 5 0 0 0 0 0 0 0 0 0 0 0 0 0 0 0 0 0 0 0 0 0 0 0 0 0 0 0 0 0 0 0 0 0 0 0 0 0 0 0 0 0 0 0 0 0 0 0 0 0 0 0

"50669_fr2" 11 9 0 0 0 0 0 2 0 0 1 9 1 0 0 3 14 0 0 11 7 7 0 0 0 8 0 2 0 9 8 0 5 0 3 0 0 0 0 0 0 0 0 0 0 0 0 0 0 0 0 0 0 0 0 0 0 0 0 0 0 0 0 0 0 0 0 0 0 0 0 0 0 0 0 5 2 11 12 15 0 2 0 7 8

"50707_fr6" 0 0 1 1 1 0 2 0 0 1 2 1 0 0 0 0 0 0 1 0 2 0 2 0 0 5 1 0 1 1 2 3 0 0 1 0 0 0 0 0 0 0 0 0 0 0 0 0 0 0 0 0 0 0 0 0 0 0 0 0 0 0 0 0 0 0 0 0 0 0 0 0 0 0 0 0 0 0 0 0 0 0 0 0 0

"50753_fr5" 0 0 0 0 0 0 0 0 2 0 0 0 0 0 0 0 0 0 0 3 0 0 1 0 0 2 0 0 0 0 0 0 0 0 0 0 0 0 0 0 0 0 0 0 0 0 0 0 0 0 0 0 0 0 0 0 0 0 0 0 0 0 0 0 0 0 0 0 0 0 0 0 0 0 0 0 0 0 0 0 0 0 0 0 0

"5094_fr6" 1 0 2 1 2 0 7 2 0 0 1 1 4 1 2 3 0 0 0 1 1 5 1 1 0 1 1 1 0 0 1 1 4 1 0 0 0 0 0 0 0 0 0 0 0 0 0 0 0 0 0 0 0 0 0 0 0 0 0 0 0 0 0 0 0 0 0 0 0 0 0 0 0 0 0 0 0 0 0 0 0 0 0 0 0

"51071_fr6" 0 0 0 0 0 0 0 0 0 2 0 0 0 0 0 0 0 0 0 0 0 0 0 0 4 0 0 0 0 0 0 0 0 0 0 0 0 0 0 0 0 0 0 0 0 0 0 0 0 0 0 0 0 0 0 0 0 0 0 0 0 0 0 0 0 0 0 0 0 0 0 0 0 0 0 0 0 0 0 0 0 0 0 0 0

"51377_fr2" 0 0 0 0 3 0 0 0 0 0 0 4 0 3 0 0 0 0 1 4 1 0 2 4 0 4 2 3 2 2 4 4 2 0 0 0 0 0 0 0 0 0 0 0 0 0 0 0 0 0 0 0 0 0 0 0 0 0 0 0 0 0 0 0 0 0 0 0 0 0 0 0 0 0 0 0 0 0 0 0 0 0 0 0 0

"51491_fr2" 6 0 0 0 5 6 0 0 0 0 0 6 0 0 0 2 0 0 0 6 0 4 0 0 0 6 0 0 0 0 0 2 0 0 0 0 0 0 0 0 0 0 0 0 0 0 0 0 0 0 0 0 0 0 0 0 0 0 0 0 0 0 0 0 0 0 0 0 0 0 0 0 0 0 0 0 0 0 0 0 0 0 0 0 0

"5150_fr4" 0 0 0 0 0 0 0 0 0 0 0 0 0 0 0 0 0 0 0 0 0 0 0 0 0 0 0 0 0 0 0 2 0 0 0 0 0 0 0 0 0 0 0 0 0 0 0 0 0 0 0 0 0 0 0 0 0 0 0 0 0 0 0 0 0 0 0 0 0 0 0 0 0 0 0 0 0 0 0 0 0 0 0 0 0

"51551_fr2" 0 1 0 0 0 0 0 0 0 0 0 0 0 2 0 1 0 2 2 0 0 0 0 0 0 0 0 0 0 0 0 2 0 0 0 0 0 0 0 0 0 0 0 0 0 0 0 0 0 0 0 0 0 0 0 0 0 0 0 0 0 0 0 0 0 0 0 0 0 0 0 0 0 0 0 0 0 0 0 0 0 0 0 0 0

"521_fr6" 0 0 0 0 0 0 0 0 0 0 0 0 0 0 0 0 0 0 0 0 0 0 0 0 0 0 0 0 0 0 0 7 0 0 0 0 0 0 0 0 0 0 0 0 0 0 0 0 0 0 0 0 0 0 0 0 0 0 0 0 0 0 0 0 0 0 0 0 0 0 0 0 0 0 0 0 0 0 0 0 0 0 0 0 0

"5310_fr1" 0 0 4 0 12 4 0 0 2 6 6 0 0 6 0 0 0 3 6 3 5 6 8 8 6 8 8 6 9 12 6 9 2 9 0 0 0 0 0 0 0 0 0 0 0 0 0 0 0 0 0 0 0 0 0 0 0 0 0 0 0 0 0 0 0 0 0 0 0 0 0 0 0 0 0 0 0 0 0 0 0 0 0 0 0

"5392_fr6" 2 0 0 0 0 0 1 0 0 0 0 0 0 0 0 4 0 0 0 0 1 0 0 2 0 0 2 0 0 3 0 7 0 0 0 0 0 0 0 0 0 0 0 0 0 0 0 0 0 0 0 0 0 0 0 0 0 0 0 0 0 0 0 0 0 0 0 0 0 0 0 0 0 0 0 0 0 0 0 0 0 0 0 0 0

"5407_fr1" 0 0 4 6 5 0 0 3 0 0 0 0 0 8 4 3 0 0 2 0 0 0 0 4 4 0 0 0 0 6 0 0 6 6 0 0 0 0 0 3 0 0 0 0 0 0 0 0 0 0 0 0 0 0 0 0 0 0 0 0 0 0 0 0 0 0 2 4 0 0 0 4 0 6 6 5 6 5 0 0 3 0 0 3 4

"5492_fr2" 0 0 0 0 0 0 0 0 0 0 0 0 0 0 0 0 0 0 0 0 0 0 0 0 0 0 0 0 0 0 0 0 0 0 0 0 0 0 0 4 0 0 0 0 0 0 0 0 0 0 0 0 0 0 0 0 0 0 0 0 0 0 0 0 0 0 0 0 0 0 0 0 0 0 0 0 0 2 0 0 3 7 2 6 10

"5492_fr3" 3 3 3 6 3 3 3 6 13 4 4 3 5 3 7 3 4 20 2 3 1 5 2 4 5 0 0 16 3 3 5 2 3 3 12 0 0 0 0 0 0 0 0 0 0 0 0 0 0 0 0 0 0 0 0 0 0 0 0 0 0 0 0 0 0 0 0 0 0 0 0 0 0 0 0 0 0 0 0 0 0 0 0 0 0

"5557_fr4" 0 0 0 0 0 0 0 0 0 0 0 0 0 0 0 0 0 0 0 0 0 0 0 0 0 0 0 0 0 0 0 0 0 0 0 0 0 0 0 0 0 0 0 0 0 0 0 0 0 0 0 0 0 0 0 0 0 0 0 0 0 0 0 0 0 0 0 2 0 0 0 0 3 0 0 0 0 0 0 0 0 0 0 0 0

"55690_fr6" 0 0 0 0 0 0 0 0 0 0 0 0 0 0 0 0 0 2 0 0 0 0 0 0 0 0 0 0 0 0 0 0 0 0 0 0 0 0 0 0 0 0 0 0 0 0 0 0 0 0 0 0 0 0 0 0 0 0 0 0 0 0 0 0 0 0 0 0 0 0 0 0 0 0 0 0 0 0 0 0 0 0 0 0 0

"5589_fr4" 5 6 0 0 1 0 7 0 0 0 0 0 0 0 0 0 0 0 0 0 0 2 0 0 0 2 0 0 0 0 0 5 0 4 0 0 0 0 0 0 0 0 0 0 0 0 0 0 0 0 0 0 0 0 0 0 0 0 0 0 0 0 0 0 0 0 0 0 0 0 0 0 0 0 0 0 0 0 0 0 0 0 0 0 0

"559_fr2" 0 0 0 0 0 0 0 0 0 0 0 0 0 0 0 0 0 0 0 0 0 0 0 0 0 0 0 0 0 0 0 0 0 0 0 0 0 3 0 6 6 0 4 6 3 0 4 0 0 0 0 0 0 0 0 0 0 0 0 0 0 0 0 0 0 0 0 0 0 0 0 0 0 0 0 0 0 0 0 0 0 0 0 0 0

"57175_fr1" 2 2 2 2 2 2 2 2 2 2 2 2 2 2 2 2 2 2 2 2 2 2 2 2 2 2 2 2 2 2 2 2 2 2 2 0 0 0 0 0 0 0 0 0 0 0 0 0 0 0 0 0 0 0 0 0 0 0 0 0 0 0 0 0 0 0 0 0 0 0 0 0 0 0 0 0 0 0 0 0 0 0 0 0 0

"5777_fr5" 0 0 0 0 0 0 0 0 0 0 0 0 0 0 0 0 2 0 0 0 0 0 0 0 0 0 0 0 0 0 0 0 0 0 0 0 0 0 0 0 0 0 0 0 0 0 0 0 0 0 0 0 0 0 0 0 0 0 0 0 0 0 0 0 0 0 0 0 0 0 0 0 0 0 0 0 0 0 0 0 0 0 0 0 0

"57964_fr5" 0 0 0 0 1 0 0 0 0 0 0 0 0 0 0 0 0 0 0 0 0 0 0 0 0 0 0 0 0 0 0 0 1 0 0 0 0 0 0 0 0 0 0 0 0 0 0 0 0 0 0 0 0 0 0 0 0 0 0 0 0 0 0 0 0 0 0 0 0 0 0 0 0 0 0 0 0 0 0 0 0 0 0 0 0

"5839_fr3" 0 0 0 0 0 0 0 0 0 0 0 0 0 0 1 0 0 0 0 0 0 0 0 0 0 0 0 0 0 0 0 0 0 0 0 0 0 0 0 0 0 0 0 0 0 0 0 0 0 0 0 0 0 0 0 0 0 0 0 0 0 0 0 0 0 0 0 0 0 0 0 0 0 0 0 0 0 0 0 0 1 6 0 0 0

"58988_fr5" 0 2 0 0 0 1 0 0 0 0 0 0 0 1 0 0 0 0 2 0 0 0 0 0 0 0 0 1 0 0 3 0 0 0 0 0 0 0 0 0 0 0 0 0 0 0 0 0 0 0 0 0 0 0 0 0 0 0 0 0 0 0 0 0 0 0 0 0 0 0 0 0 0 0 0 0 0 0 0 0 0 0 0 0 0

"5991_fr4" 0 0 0 0 0 0 0 0 0 0 0 0 0 3 0 0 0 0 0 0 0 0 0 0 0 3 0 0 0 0 0 0 0 0 0 0 0 0 0 0 0 0 0 0 0 0 0 0 0 0 0 0 0 0 0 0 0 0 0 0 0 0 0 0 0 0 0 0 0 0 0 0 0 0 0 0 0 0 0 0 0 0 0 0 0

"6039_fr2" 0 0 0 0 0 0 0 0 0 0 0 0 0 0 0 0 0 0 0 0 0 0 0 0 0 0 0 0 0 0 0 0 0 0 0 0 0 0 0 0 0 0 0 0 0 0 0 0 0 0 0 0 0 0 0 0 0 0 0 0 17 8 17 17 18 21 10 0 0 11 14 12 11 0 15 9 8 8 9 8 3 4 1 6 3

"608_fr6" 0 0 0 0 0 0 0 0 0 0 0 1 0 0 0 0 0 0 0 0 1 0 0 0 0 1 0 0 0 0 0 0 0 0 1 0 0 0 0 0 0 0 0 0 0 0 0 0 0 0 0 0 0 0 0 0 0 0 0 0 0 0 0 0 0 0 0 0 0 0 0 0 0 0 0 0 0 0 0 0 0 0 0 0 0

"626_fr5" 0 4 0 0 5 0 0 0 0 17 3 0 23 0 0 0 0 7 0 0 5 0 0 0 0 0 4 14 0 4 0 6 0 0 0 0 0 0 0 0 0 0 0 0 0 0 0 0 0 0 0 0 0 0 0 0 0 0 0 0 0 2 0 0 0 0 0 2 0 0 6 4 4 4 5 7 10 10 7 6 8 5 6 9 5

"6287_fr4" 0 0 1 1 1 2 2 2 0 0 3 0 0 0 0 0 0 0 1 0 1 0 0 0 0 1 3 1 0 1 0 2 0 0 0 0 0 0 0 0 2 0 0 0 0 0 0 2 0 0 0 0 0 0 0 0 0 0 0 3 0 0 0 0 0 0 0 1 0 0 0 0 1 0 1 0 0 0 0 0 0 0 0 0 0

"6306_fr2" 0 0 0 0 0 0 0 0 0 0 0 0 0 0 0 0 0 0 0 0 3 0 0 0 0 0 0 0 0 0 0 6 0 0 0 0 0 0 0 0 0 0 0 0 0 0 0 0 0 0 0 0 0 0 0 0 0 0 0 0 0 0 0 0 0 0 0 0 0 0 0 0 0 0 0 0 0 0 0 0 0 0 0 0 0

"6366_fr2" 0 0 0 0 8 0 0 0 0 0 0 0 0 0 0 0 0 0 0 0 0 0 0 0 0 0 0 0 0 0 0 0 0 0 0 0 0 0 0 0 0 0 0 0 0 0 0 0 0 0 0 0 0 0 0 0 0 0 0 0 0 0 0 0 0 0 0 0 0 0 0 0 0 0 0 0 0 0 0 0 0 0 0 0 0

"6394_fr3" 0 0 0 0 0 0 0 0 0 0 0 0 0 0 0 0 0 0 0 0 0 0 2 0 0 0 0 0 0 0 0 0 0 0 0 0 0 0 0 0 0 0 0 0 0 0 0 0 0 0 0 0 0 0 0 0 0 0 0 0 0 0 0 0 0 0 0 0 0 0 0 0 0 0 0 0 0 0 0 0 0 0 0 0 0

"64_fr5" 0 0 0 0 0 0 0 0 0 0 0 0 0 0 0 0 0 0 0 0 0 0 0 0 0 0 0 0 0 0 0 0 0 0 0 0 3 0 2 0 15 6 4 27 27 32 26 24 5 29 26 30 25 31 28 32 28 29 28 32 11 28 0 27 32 12 10 16 4 25 6 2 6 0 0 0 0 0 0 0 0 1 0 0 9

"6444_fr2" 0 0 0 0 0 0 0 0 0 0 0 0 0 0 0 0 0 0 0 0 0 0 0 0 0 0 0 0 0 0 0 0 0 0 1 0 0 0 0 0 0 0 0 0 0 0 0 0 0 0 0 0 0 0 0 0 0 0 0 0 0 0 0 0 0 0 0 0 0 0 0 0 0 0 0 0 0 0 0 0 0 0 0 0 0

"6495_fr1" 0 0 0 0 0 0 0 0 0 0 0 0 0 0 0 0 0 0 0 0 0 0 0 0 1 0 0 0 0 0 0 0 0 0 0 0 0 0 0 0 0 0 0 0 0 0 0 0 0 0 0 0 0 0 0 0 0 0 0 0 0 0 0 0 0 0 0 0 0 0 0 0 0 0 0 0 0 0 0 0 0 0 0 0 0

"6538_fr6" 2 5 4 8 3 3 5 5 4 4 1 3 6 4 3 2 3 3 4 4 3 8 8 4 2 6 4 6 3 2 7 0 3 4 4 0 0 0 3 0 2 0 6 2 2 3 3 2 3 2 3 0 5 3 3 5 4 4 3 2 0 0 0 0 0 1 2 3 0 0 0 0 0 0 1 5 2 3 1 2 0 0 4 1 4

"657_fr4" 0 2 0 1 0 0 0 4 1 0 1 0 0 0 0 0 0 0 0 2 3 0 0 2 0 1 1 0 2 0 0 0 2 2 2 0 0 0 0 0 0 0 0 0 0 0 0 0 0 0 0 0 0 0 0 0 0 0 0 0 0 0 0 0 0 0 0 0 0 0 0 0 0 0 0 0 0 0 0 0 0 0 0 0 0

"65834_fr4" 15 18 14 12 15 14 17 19 17 11 7 14 21 18 18 20 19 15 18 15 19 14 21 15 15 23 15 12 15 16 18 17 17 14 12 0 0 2 3 4 2 0 2 3 4 1 5 5 5 3 5 7 4 6 4 5 3 2 3 4 4 0 2 5 2 11 11 10 15 7 16 16 12 16 18 10 19 16 17 12 8 9 17 13 11

"65991_fr5" 0 1 4 5 0 2 0 3 0 0 0 0 0 0 0 0 0 0 1 0 0 1 0 2 0 2 3 0 3 0 0 4 1 0 0 0 0 0 0 0 0 0 0 0 0 0 0 0 0 0 0 0 0 0 0 0 0 0 0 0 0 0 0 0 0 0 0 0 0 0 0 0 0 0 0 0 0 0 0 0 0 0 0 0 0

"6689_fr5" 0 0 0 0 2 0 0 0 0 0 0 0 0 0 0 0 0 0 0 0 1 0 4 5 0 2 2 0 3 0 0 2 1 0 0 0 0 0 0 0 0 0 0 0 0 0 0 0 0 0 0 0 0 0 0 0 0 0 0 0 0 0 0 0 0 0 0 0 0 0 0 0 0 0 0 0 0 0 0 0 0 0 0 0 0

"67263_fr2" 0 0 0 0 0 0 0 0 0 0 0 0 0 0 0 0 0 0 0 0 0 0 0 0 0 0 0 0 0 0 0 0 0 0 0 0 0 2 4 0 2 3 4 4 4 4 4 4 4 4 4 4 4 4 4 4 4 4 4 4 4 4 4 4 4 4 4 4 1 4 0 0 0 0 0 0 0 0 0 0 0 0 0 0 0

"6766_fr3" 0 0 0 0 0 0 0 0 0 0 0 3 0 0 0 0 0 0 0 0 0 0 0 0 0 0 0 0 0 0 0 0 0 0 0 0 0 0 0 0 0 0 0 0 0 0 0 0 0 0 0 0 0 0 0 0 0 0 0 0 0 0 0 0 0 0 0 0 0 0 0 0 0 0 0 0 0 0 0 0 0 0 0 0 0

"679_fr2" 0 0 0 0 0 0 2 0 0 0 0 0 0 0 0 0 0 0 0 0 0 0 0 0 0 0 0 0 0 0 0 0 0 0 0 0 0 0 0 0 0 0 0 0 0 0 0 0 0 0 0 0 0 0 0 0 0 0 0 0 0 0 0 0 0 0 0 0 0 0 0 0 0 0 0 0 0 0 0 0 0 0 0 0 0

"681_fr6" 0 0 0 0 0 0 0 0 0 0 0 0 0 0 0 0 0 0 0 0 0 0 0 0 0 0 0 0 0 0 0 0 0 0 0 0 0 0 0 0 0 0 0 0 0 0 0 0 0 0 0 2 0 0 0 0 0 0 0 0 0 0 0 0 0 0 0 0 0 0 0 0 0 0 0 0 0 0 0 0 0 0 0 0 0

"6933_fr4" 0 0 0 0 0 0 0 0 0 0 0 0 0 0 0 0 0 0 0 0 0 0 0 0 0 0 0 0 0 0 0 0 0 0 0 0 0 0 0 0 0 0 0 0 0 0 0 0 0 0 0 0 0 0 0 0 0 0 0 0 6 8 6 0 4 7 7 8 6 0 0 2 0 0 0 0 0 0 0 0 0 6 0 0 2

"6933_fr5" 0 0 0 0 0 0 0 0 0 0 0 0 0 0 0 0 0 0 0 0 0 0 0 0 0 0 0 0 0 0 0 0 0 0 0 8 1 6 5 12 5 8 5 6 6 8 8 7 7 7 7 0 8 7 8 7 6 6 0 7 0 0 0 0 0 0 0 0 0 0 0 0 0 0 0 0 0 0 0 0 0 0 0 0 0

"6945_fr6" 2 5 2 2 4 5 2 2 2 2 2 2 0 2 1 2 3 2 2 2 3 2 3 3 2 2 2 3 6 4 2 2 2 2 2 0 0 0 0 0 0 0 0 0 0 0 0 0 0 0 0 0 0 0 0 0 0 0 0 0 0 0 0 0 0 0 0 0 0 0 0 0 0 0 0 0 0 0 0 0 0 0 0 0 0

"697_fr6" 0 0 0 0 0 0 0 0 0 0 0 9 0 0 0 0 0 0 0 0 0 0 5 0 0 0 0 0 0 0 7 0 0 0 0 0 0 0 0 0 0 0 0 0 0 0 0 0 0 0 0 0 0 0 0 0 0 0 0 0 0 0 0 0 0 0 0 0 0 0 0 0 0 0 0 0 0 0 0 0 0 0 0 0 0

"69913_fr6" 6 1 7 0 7 2 0 0 0 6 2 7 0 3 4 0 4 0 2 6 7 3 5 4 2 4 5 4 6 2 8 7 7 4 2 0 0 0 0 0 0 0 0 0 0 0 0 0 0 0 0 0 0 0 0 0 0 0 0 0 0 0 0 0 0 0 0 0 0 0 0 0 0 0 0 0 0 0 0 0 0 0 0 0 0

"70483_fr5" 0 0 3 3 0 2 0 3 1 0 0 0 0 0 0 0 0 0 0 0 0 0 0 0 0 0 4 0 1 0 0 0 0 0 0 0 0 0 0 0 0 0 0 0 0 0 0 0 0 0 0 0 0 0 0 0 0 0 0 0 0 0 0 0 0 0 0 0 0 0 0 0 0 0 0 0 0 0 0 0 0 0 0 0 0

"7061_fr2" 4 8 5 4 10 4 9 8 8 11 6 0 10 4 5 9 0 6 3 4 7 4 4 5 11 0 3 5 4 4 4 6 6 4 5 0 2 2 5 2 0 4 7 5 11 7 5 9 7 8 8 6 7 10 9 6 9 6 5 8 4 7 6 5 5 4 4 0 0 2 0 0 0 0 0 0 0 0 0 0 0 0 0 0 0

"71063_fr4" 4 6 4 6 5 7 8 8 8 0 6 6 5 6 5 6 12 4 5 8 8 5 4 4 5 4 6 0 4 0 5 6 5 7 5 8 8 4 0 4 0 0 0 0 0 0 0 0 0 0 0 0 0 0 0 0 0 5 0 0 0 9 0 2 0 6 3 4 4 0 6 4 7 3 4 4 4 5 5 4 8 6 5 5 5

"7169_fr4" 0 2 0 0 4 3 0 0 0 0 0 1 0 15 0 0 2 0 6 0 2 0 6 0 0 1 0 0 8 11 0 0 2 0 0 0 0 0 0 0 0 0 0 0 0 0 0 0 0 0 0 0 0 0 0 0 0 0 0 0 0 0 0 0 0 0 0 0 0 0 0 0 0 0 0 0 0 0 0 0 0 0 0 0 0

"73635_fr4" 0 0 0 0 0 0 0 0 0 2 0 0 0 0 0 3 0 0 0 0 0 0 0 0 0 0 0 0 0 0 1 2 0 0 0 0 0 0 0 0 0 0 0 0 0 0 0 0 0 0 0 0 0 0 0 0 0 0 0 0 0 0 0 0 0 0 0 0 0 0 0 0 0 0 0 0 0 0 0 0 0 0 0 0 0

"737_fr2" 0 4 4 0 0 0 3 3 2 0 0 3 2 4 4 3 2 2 2 2 4 2 2 2 0 2 0 2 0 0 2 0 2 0 0 0 0 0 0 0 0 0 0 0 0 0 0 0 0 0 0 0 0 0 0 0 0 0 0 0 0 0 0 0 0 0 0 0 0 0 0 0 0 0 0 0 0 0 0 0 0 0 0 0 0

"7432_fr3" 6 5 0 0 0 1 2 2 2 6 0 0 4 0 0 0 0 0 0 0 0 5 0 0 0 0 0 0 5 0 0 0 4 0 0 0 0 0 0 0 0 0 0 0 0 0 0 0 0 0 0 0 0 0 0 0 0 0 0 0 0 0 0 0 0 0 0 0 0 0 0 0 0 0 0 0 0 0 0 0 0 0 0 0 0

"7625_fr2" 3 8 1 0 3 3 3 3 1 0 3 9 2 11 1 5 1 1 6 3 4 1 3 7 0 4 1 4 1 4 5 11 4 2 0 0 0 0 0 0 0 0 0 0 0 0 0 0 0 0 0 0 0 0 0 0 0 0 0 0 0 0 0 0 0 0 0 0 0 0 0 0 0 0 0 0 0 0 0 0 0 0 0 0 0

"7880_fr2" 0 0 0 0 0 0 0 0 0 0 0 0 0 0 0 0 0 0 0 0 0 0 0 0 0 0 0 0 0 0 0 0 0 0 0 0 0 0 0 0 0 0 0 0 0 0 0 0 0 0 0 0 0 0 0 0 0 0 0 0 0 0 0 0 0 0 0 0 0 0 0 0 2 0 0 2 0 0 0 2 0 0 0 0 0

"7908_fr4" 5 4 6 0 3 4 7 4 1 0 0 2 0 0 0 0 5 0 0 0 0 5 0 11 2 0 4 0 2 4 0 5 0 9 0 0 0 0 0 0 0 0 0 0 0 0 0 0 0 0 0 0 0 0 0 0 0 0 0 0 0 0 0 0 0 0 0 0 0 0 0 0 0 0 0 0 0 0 0 0 0 0 0 0 0

"7942_fr6" 2 2 0 2 0 3 2 2 2 0 2 0 2 0 0 2 2 0 2 2 2 2 3 4 0 3 2 1 4 5 2 4 3 0 2 0 0 0 0 0 0 0 0 0 0 0 0 0 0 0 0 0 0 0 0 0 0 0 0 0 0 0 0 0 0 0 0 2 1 0 0 0 0 2 2 0 5 3 3 2 0 0 2 2 2

"7953_fr6" 5 7 0 0 4 1 0 0 0 0 5 10 0 11 0 0 0 0 4 8 7 0 6 12 0 2 3 14 0 4 9 11 0 6 0 0 0 0 0 0 0 0 0 0 0 0 0 0 0 0 0 0 0 0 0 0 0 0 0 0 0 0 0 0 0 0 0 0 0 0 0 0 0 0 0 0 0 0 0 0 0 0 0 0 0

"7983_fr6" 0 0 0 0 0 0 0 0 2 0 0 0 0 2 0 0 0 0 0 0 0 0 4 0 0 0 0 0 1 0 0 0 2 0 0 0 0 0 0 0 0 0 0 0 0 0 0 0 0 0 0 0 0 0 0 0 0 0 0 0 0 0 0 0 0 0 0 0 0 0 0 0 0 0 0 0 0 0 0 0 0 0 0 0 0

"8_fr4" 0 0 0 0 0 2 0 0 0 0 0 0 0 0 0 0 0 0 0 0 0 0 0 0 0 0 1 0 0 0 0 0 0 0 0 0 0 0 0 0 0 0 0 0 0 0 0 0 0 0 0 0 0 0 0 0 0 0 0 0 0 0 0 0 0 0 0 0 0 0 0 0 0 0 0 0 0 0 0 0 0 0 0 0 0

"8013_fr1" 0 0 0 0 0 0 0 0 0 0 0 0 0 0 0 0 0 0 0 0 0 0 0 0 0 0 0 0 0 0 0 0 0 0 0 0 0 0 0 0 0 0 0 0 0 0 0 0 0 0 0 0 0 0 0 0 0 0 0 0 0 0 0 0 0 0 0 0 0 0 0 0 1 0 0 1 2 2 3 3 0 1 0 0 0

"8064_fr3" 0 0 5 0 0 0 0 0 0 0 0 0 0 0 0 0 0 3 0 0 0 0 0 0 0 0 0 0 0 0 0 0 0 0 0 0 0 0 0 0 0 0 0 0 0 0 0 0 0 0 0 0 0 0 0 0 0 0 0 0 0 0 0 0 0 0 0 0 0 0 0 0 0 0 0 0 0 0 0 0 0 0 0 0 0

"8186_fr2" 0 0 0 0 0 0 1 0 0 0 0 0 0 0 0 1 0 0 0 0 0 0 0 4 0 0 0 0 0 0 0 3 0 0 0 0 0 0 0 0 0 0 0 0 0 0 0 0 0 0 0 0 0 0 0 0 0 0 0 0 0 0 0 0 0 0 0 0 0 0 0 0 0 0 0 0 0 0 0 0 0 0 0 0 0

"8190_fr1" 15 13 13 15 2 11 15 14 17 12 14 13 14 13 14 14 14 16 10 13 11 15 2 2 12 10 9 2 12 9 19 10 14 13 18 9 8 8 0 8 0 0 2 0 0 0 0 0 0 0 0 0 0 0 0 0 0 0 0 0 0 0 0 0 0 0 0 0 0 0 0 0 0 0 0 8 6 12 11 6 11 12 13 12 12

"8288_fr5" 0 0 6 8 3 0 5 1 8 6 0 0 0 0 0 0 2 8 0 0 0 1 0 0 0 0 0 0 6 0 6 0 2 0 7 0 0 0 0 0 0 0 0 0 0 0 0 0 0 0 0 0 0 0 0 0 0 0 0 0 0 0 0 0 0 0 0 0 0 0 0 0 0 0 0 0 0 0 0 0 0 0 0 0 0

"84749_fr6" 0 0 0 0 0 0 0 0 0 0 0 0 0 0 0 0 0 0 0 0 0 0 0 0 0 0 0 0 0 0 0 0 0 0 0 0 0 0 2 0 0 0 0 3 2 3 1 0 3 2 3 4 2 4 2 4 2 1 0 0 0 2 1 1 1 1 2 2 0 2 0 0 0 0 0 0 0 0 0 0 0 0 0 0 0

"8483_fr6" 0 0 2 2 2 0 0 1 1 0 0 0 0 0 5 2 1 0 0 2 0 0 0 5 0 0 0 0 0 3 1 1 1 2 4 0 0 0 0 0 0 0 0 0 0 0 0 0 0 0 0 0 0 0 0 0 0 0 0 0 0 0 0 0 0 0 0 0 0 0 0 0 0 0 0 0 0 0 0 0 0 0 0 0 0

"85980_fr2" 4 0 1 0 0 1 0 0 0 0 0 4 0 1 3 4 0 0 1 1 2 2 0 1 1 0 1 0 0 0 0 0 1 0 0 0 0 0 0 0 0 0 0 0 0 0 0 0 0 0 0 0 0 0 0 0 0 0 0 0 0 0 0 0 0 0 0 0 0 0 0 0 0 0 0 0 0 0 0 0 0 0 0 0 0

"8708_fr1" 0 0 2 0 0 0 0 0 0 0 0 0 0 0 0 0 0 0 0 0 0 0 1 0 0 0 0 0 0 0 0 1 0 0 0 0 0 0 0 0 0 0 0 0 0 0 0 0 0 0 0 0 0 0 0 0 0 0 0 0 0 0 0 0 0 0 0 0 0 0 0 0 0 0 0 0 0 0 0 0 0 0 0 0 0

"8722_fr3" 0 0 0 0 2 0 0 0 0 1 0 0 0 3 0 0 0 0 0 2 0 0 0 2 0 0 5 0 1 0 0 3 0 0 0 0 0 0 0 0 0 0 0 1 0 0 0 0 0 3 0 0 0 0 0 1 0 0 0 0 0 0 0 0 0 0 0 0 0 0 0 0 0 0 0 0 0 0 0 0 0 0 0 0 0

"87598_fr2" 0 0 0 0 0 0 0 0 0 0 0 0 0 0 0 0 0 0 0 0 0 0 0 0 0 0 0 0 0 0 0 0 0 0 0 6 4 21 36 19 0 34 43 41 42 47 49 38 44 58 53 55 42 57 50 49 47 41 47 55 8 42 43 49 51 49 39 35 43 42 2 25 20 24 27 21 20 0 0 17 0 0 0 0 0

"87677_fr3" 4 0 0 3 0 0 0 0 6 0 0 0 0 0 4 0 3 0 0 0 0 2 0 0 2 0 0 0 0 0 0 0 0 0 0 0 0 0 0 0 0 0 0 0 0 0 0 0 0 0 0 0 0 0 0 0 0 0 0 0 0 0 0 0 0 0 0 0 0 0 0 0 0 0 0 0 0 0 0 0 0 0 0 0 0

"87717_fr4" 0 0 0 0 0 0 0 0 0 0 0 0 0 0 0 0 0 0 0 0 0 0 2 0 0 2 2 0 0 0 0 8 4 0 0 0 0 0 0 0 0 0 0 0 0 0 0 0 0 0 0 0 0 0 0 0 0 0 0 0 0 0 0 0 0 0 0 0 0 0 0 0 0 0 0 0 0 0 0 0 0 0 0 0 0

"87769_fr2" 4 3 4 0 4 0 4 0 0 0 0 0 0 0 0 0 4 0 0 0 0 0 0 7 0 0 0 0 0 1 0 2 0 0 0 0 0 0 0 0 0 0 0 0 0 0 0 0 0 0 0 0 0 0 0 0 0 0 0 0 0 0 0 0 0 0 0 0 0 0 0 0 0 0 0 0 0 0 0 0 0 0 0 0 0

"88066_fr3" 0 0 0 0 0 0 0 0 0 0 0 0 0 0 0 0 0 0 0 0 0 0 0 3 0 0 0 0 0 0 0 0 0 0 0 0 0 0 0 0 0 0 0 0 0 0 0 0 0 0 0 0 0 0 0 0 0 0 0 0 0 0 0 0 0 0 0 0 0 0 0 0 0 0 0 0 0 0 0 0 0 0 0 0 0

"88301_fr2" 0 0 0 0 0 0 0 0 0 0 0 0 0 3 0 0 0 0 0 0 0 0 0 0 0 0 0 0 0 0 0 0 0 0 0 0 0 0 0 0 0 0 0 0 0 0 0 0 0 0 0 0 0 0 0 0 0 0 0 0 0 0 0 0 0 0 0 0 0 0 0 0 0 0 0 0 0 0 0 0 0 0 0 0 0

"8832_fr2" 0 1 7 4 5 3 0 3 3 2 0 0 4 4 2 3 0 0 5 6 3 4 5 3 0 2 5 4 5 6 0 2 5 4 2 0 0 0 0 0 0 0 0 0 0 0 0 0 0 0 0 0 0 0 0 0 0 0 0 0 0 0 0 0 0 0 0 0 0 0 0 0 0 0 0 0 0 0 0 0 0 0 0 0 0

"8873_fr3" 0 0 0 0 0 0 0 0 0 0 0 0 0 0 0 0 0 0 0 0 0 0 0 0 0 0 0 0 0 0 0 2 0 0 0 0 0 0 0 0 0 0 0 0 0 0 0 0 0 0 0 0 0 0 0 0 0 0 0 0 0 0 0 0 0 0 0 0 0 0 0 0 0 0 0 0 0 0 0 0 0 0 0 0 0

"8924_fr2" 0 0 0 0 0 0 0 0 0 0 0 0 0 0 0 0 0 0 0 0 0 0 0 0 0 0 0 0 0 0 0 0 0 0 0 0 3 10 9 8 0 13 14 27 8 15 10 7 10 10 17 18 6 22 21 15 16 5 7 7 17 16 13 11 8 9 9 5 2 5 0 0 1 0 0 0 0 0 0 0 0 4 0 0 3

"9002_fr1" 1 0 6 10 0 10 2 9 15 0 1 1 0 1 0 0 1 0 2 0 11 10 2 0 0 3 1 1 8 6 0 0 0 0 0 0 0 2 0 0 0 0 5 0 0 0 0 0 0 0 0 0 0 0 0 0 0 13 0 0 0 0 0 0 0 0 0 0 0 0 0 6 0 10 0 8 5 13 11 0 6 2 6 8 2

"90711_fr1" 3 0 0 0 2 0 0 0 0 0 0 0 0 0 0 5 0 0 2 0 2 0 0 0 0 0 0 0 0 0 0 0 0 0 0 0 0 0 0 0 0 0 0 0 0 0 0 0 0 0 0 0 0 0 0 0 0 0 0 0 0 0 0 0 0 0 0 0 0 0 0 0 0 0 0 0 0 0 0 0 0 0 0 0 0

"9264_fr5" 0 0 2 6 0 6 0 0 5 0 0 0 0 0 0 0 0 0 0 0 0 0 4 0 0 0 3 0 5 0 0 1 2 0 0 0 0 0 0 0 0 0 0 0 0 0 0 0 0 0 0 0 0 0 0 0 0 0 0 0 0 0 0 0 0 0 0 0 0 0 0 0 0 0 0 0 0 0 0 0 0 0 0 0 0

"9333_fr6" 0 0 0 0 0 0 0 0 0 0 0 0 0 0 0 0 0 0 0 0 0 0 0 0 0 0 0 0 0 0 0 0 0 0 0 0 0 0 0 0 0 0 0 0 0 0 0 0 0 0 0 0 0 0 0 0 0 0 0 0 0 0 0 0 0 0 0 0 0 0 0 0 0 1 4 0 0 0 0 0 3 0 0 0 0

"9416_fr2" 0 0 0 0 0 0 0 0 0 0 0 0 0 0 0 0 0 0 0 0 0 0 0 0 0 0 0 0 0 0 0 0 0 0 0 0 0 7 0 0 0 0 4 0 0 3 0 2 2 0 2 0 4 0 0 0 2 3 2 0 0 0 0 4 4 0 1 2 0 0 0 2 0 0 0 0 0 0 0 0 0 0 0 0 0

"9438_fr2" 6 0 0 17 0 0 2 0 21 9 0 0 0 0 3 4 1 24 0 0 0 2 0 8 7 0 0 0 2 4 21 0 4 0 24 0 0 0 0 0 0 0 0 0 0 0 0 0 0 0 0 0 0 0 0 0 0 0 0 0 0 0 0 0 0 0 0 0 0 0 0 0 0 0 0 0 4 13 10 0 14 11 11 13 5

"94725_fr5" 0 0 0 0 0 1 0 0 0 0 0 0 0 0 0 0 0 0 0 0 0 0 0 0 0 0 4 0 2 0 0 0 0 0 0 0 0 0 0 0 0 0 0 0 0 0 0 0 0 0 0 0 0 0 0 0 0 0 0 0 0 0 0 0 0 0 0 0 0 0 0 0 0 0 0 0 0 0 0 0 0 0 0 0 0

"95_fr1" 0 0 0 0 0 0 0 0 0 6 0 0 0 0 0 0 0 0 0 0 0 0 0 0 0 0 0 0 0 0 0 0 0 0 0 0 0 0 0 0 0 0 0 0 0 0 0 0 0 0 0 0 0 0 0 0 0 0 0 0 0 0 0 0 0 0 0 0 0 0 0 0 0 0 0 0 0 0 0 0 0 0 0 0 0

"9612_fr5" 0 0 0 0 0 0 0 0 0 0 0 0 0 0 0 0 0 0 0 0 0 0 0 0 0 0 0 0 0 0 0 0 0 0 0 0 0 0 0 0 0 0 0 0 0 0 0 0 0 0 0 0 0 0 0 0 0 0 3 0 0 0 0 0 0 0 0 0 0 0 0 0 0 0 0 0 0 0 0 0 0 0 0 0 0

"9667_fr6" 0 5 3 0 0 0 0 0 0 9 0 0 6 0 3 0 0 6 0 0 5 0 0 0 6 0 0 0 0 0 0 0 0 0 3 0 0 0 0 0 0 0 0 0 0 0 0 0 0 0 0 0 0 0 0 0 0 0 0 0 0 0 0 0 0 0 0 0 0 0 0 0 0 0 0 0 0 0 0 0 0 0 0 0 0

"9730_fr4" 0 2 5 3 0 0 0 0 0 2 0 0 4 0 0 4 0 2 0 3 0 0 3 4 7 0 5 2 0 0 7 10 3 0 1 0 0 0 0 0 0 0 0 0 0 0 0 0 0 0 0 0 0 0 0 0 0 0 0 0 0 0 0 0 0 0 0 0 0 0 0 0 0 0 0 0 0 0 0 0 0 0 0 0 0

"9731_fr2" 0 0 7 0 2 1 0 0 0 0 0 0 0 2 0 0 0 0 2 0 2 0 2 0 0 2 0 0 3 0 0 0 0 1 0 0 0 0 0 0 0 0 0 0 0 0 0 0 0 0 0 0 0 0 0 0 0 0 0 0 0 0 0 0 0 0 0 0 0 0 0 0 0 0 0 0 0 0 0 0 0 0 0 0 0

"9788_fr4" 0 0 0 0 0 0 0 0 0 0 0 0 0 0 0 0 0 0 0 0 0 0 0 0 0 0 0 0 0 0 0 0 0 0 0 0 3 4 7 7 0 5 12 10 10 9 10 10 10 10 11 11 7 10 10 10 9 10 10 11 12 0 8 11 7 0 0 0 6 0 0 0 0 0 0 0 0 0 0 0 0 0 0 0 0

"97936_fr6" 0 0 0 0 0 0 0 0 0 0 0 0 0 0 0 0 0 0 0 0 0 0 0 0 0 0 0 0 0 0 0 0 0 0 0 0 0 0 0 0 0 0 0 0 0 0 0 0 0 0 0 0 0 0 0 0 0 0 0 0 0 0 0 0 0 0 0 0 0 0 0 0 0 0 0 0 0 1 1 0 1 1 2 7 2

"98010_fr6" 2 2 2 6 1 2 4 4 3 3 2 0 4 2 3 4 3 3 0 2 0 3 0 2 4 0 0 0 3 0 3 0 2 1 4 0 0 0 0 0 0 0 0 0 0 0 0 0 0 0 0 0 0 0 0 0 0 0 0 0 0 0 0 0 0 0 0 0 0 0 0 0 0 0 0 0 0 0 0 0 0 1 4 2 2

"98012_fr3" 0 2 0 0 2 0 2 2 0 6 1 0 2 0 0 1 0 6 0 0 2 1 2 4 1 0 4 2 5 6 2 6 8 0 0 0 0 0 0 0 0 0 0 0 0 0 0 0 0 0 0 0 0 0 0 0 0 0 0 0 0 0 0 0 0 0 0 0 0 0 0 0 0 0 0 0 0 0 0 0 0 0 0 0 0

"98023_fr1" 1 18 6 9 4 10 14 11 0 3 4 5 12 6 0 15 4 13 13 4 4 3 6 1 0 0 8 4 4 5 0 4 4 16 2 0 0 0 0 4 0 0 0 4 0 0 0 0 0 0 0 0 0 0 0 0 0 0 0 0 0 0 0 0 0 2 0 0 4 0 0 0 0 3 4 0 0 0 0 0 1 2 0 0 0

"98110_fr5" 0 0 0 0 0 0 0 0 1 0 0 0 0 0 0 0 0 0 0 0 0 0 0 0 1 0 0 0 0 0 0 0 0 0 0 0 0 0 0 0 0 0 0 0 0 0 0 0 0 0 0 0 0 0 0 0 0 0 0 0 0 0 0 0 0 0 0 0 0 0 0 0 0 0 0 0 0 0 0 0 0 0 0 0 0

"98134_fr4" 0 0 0 0 0 0 0 0 0 0 0 0 0 0 0 1 0 0 0 0 0 0 0 0 0 0 0 0 0 0 0 0 0 0 0 0 0 0 0 0 0 0 0 0 0 0 0 0 0 0 0 0 0 0 0 0 0 0 0 0 0 0 0 0 0 0 0 0 0 0 0 0 0 0 0 0 0 0 0 0 0 0 0 0 0

"98145_fr6" 0 0 0 0 0 0 0 0 3 0 0 0 0 0 0 0 0 0 0 0 0 0 0 0 0 0 0 0 0 0 0 0 0 0 0 0 0 0 0 0 0 0 0 0 0 0 0 0 0 0 0 0 0 0 0 0 0 0 0 0 0 0 0 0 0 0 0 0 0 0 0 0 0 0 0 0 0 0 0 0 0 0 0 0 0

"98152_fr5" 6 3 3 0 2 5 3 0 3 0 2 2 0 2 0 0 5 0 6 6 4 5 5 4 0 5 4 4 4 2 0 3 4 4 0 0 0 0 0 0 0 0 0 0 0 0 0 0 0 0 0 0 0 0 0 0 0 0 0 0 0 0 0 0 2 2 0 7 0 0 5 0 0 5 9 5 7 8 7 7 3 6 4 5 5

"98188_fr3" 0 0 0 0 0 0 0 0 0 0 0 0 0 0 0 0 0 0 0 0 0 0 8 0 0 11 0 0 0 0 0 0 0 0 0 0 0 0 0 0 0 0 0 0 0 0 0 0 0 0 0 0 0 0 0 0 0 0 0 0 0 0 0 0 0 0 0 0 0 0 0 0 0 0 0 0 0 0 0 0 0 0 0 0 0

"9819_fr1" 0 0 0 0 0 0 0 0 0 0 0 0 0 0 0 0 0 0 0 0 0 0 0 0 0 0 0 0 0 0 0 0 0 0 0 0 0 0 0 0 0 0 0 0 0 0 0 0 0 0 0 0 0 0 0 0 0 0 0 0 0 0 0 0 0 0 0 0 0 0 0 0 0 0 0 0 9 7 0 5 0 0 0 0 0

"98247_fr5" 0 0 0 0 16 0 0 6 0 0 0 1 0 4 4 0 0 0 14 2 1 0 15 6 0 0 0 8 0 3 1 2 12 1 0 0 0 0 0 0 0 0 0 0 0 0 0 0 0 0 0 0 0 0 0 0 0 0 0 0 0 0 0 0 0 0 0 0 0 0 0 0 0 0 0 0 0 0 0 0 0 0 0 0 0

"98260_fr1" 0 0 3 0 0 0 0 0 0 3 0 0 3 0 0 0 0 4 0 0 0 0 0 0 4 0 0 0 0 0 0 0 0 0 0 0 0 0 0 0 0 0 0 0 0 0 0 0 0 0 0 0 0 0 0 0 0 0 0 0 0 0 0 0 0 0 0 0 0 0 0 0 0 0 0 0 0 0 0 0 0 0 0 0 0

"98464_fr4" 0 0 0 0 0 0 3 0 0 2 0 0 7 0 0 2 0 2 0 0 6 0 0 0 5 0 8 15 0 0 0 9 11 0 0 0 1 4 0 3 2 0 0 0 5 0 3 4 2 0 0 0 2 1 0 3 5 4 3 2 0 3 2 0 2 6 4 5 3 3 4 0 2 4 1 4 9 0 11 0 2 0 0 3 12

"99411_fr2" 0 2 0 0 3 0 5 2 0 4 3 0 4 0 0 0 0 0 3 1 2 2 2 1 4 0 0 1 0 0 3 0 3 3 2 0 0 0 0 0 0 0 0 0 0 0 0 0 0 0 0 0 0 0 0 0 0 0 0 0 0 0 0 0 0 0 0 0 0 0 0 0 0 0 0 0 0 0 0 0 0 0 0 0 0
