# Supplementary material for: Co-expression network analysis identifies gonad- and embryo-associated protein modules in the sentinel species Gammarus fossarum
Source: Sci Rep. 2019 May 27;9:7862. doi: 10.1038/s41598-019-44203-5 (PMC6536538; doi:10.1038/s41598-019-44203-5)
Supplement: Supplementary file 4 — Dataset 2 [file 41598_2019_44203_MOESM4_ESM.docx]

Co-expression network analysis identifies gonad- and embryo-associated protein modules in the sentinel species *Gammarus fossarum*.

Davide Degli Esposti^1^, Christine Almunia^2^, Marc-Antoine Guery^1^, Natacha Koenig^1^, Jean Armengaud^2^, Arnaud Chaumot^1^, Olivier Geffard^1^

Sample_names P J0 J1 J2 J3 J4 J7 AB C1 C2 D1 D2 S1 S2 S3 S4 S5 Testes Ovaries Embryos group tissue dev pubmed

X25293947testis.P.n1 1 0 0 0 0 0 0 0 0 0 0 0 0 0 0 0 0 1 0 0 P testis n1 X25293947

X25293947testis.P.n2 1 0 0 0 0 0 0 0 0 0 0 0 0 0 0 0 0 1 0 0 P testis n2 X25293947

X25293947testis.P.n3 1 0 0 0 0 0 0 0 0 0 0 0 0 0 0 0 0 1 0 0 P testis n3 X25293947

X25293947testis.P.n4 1 0 0 0 0 0 0 0 0 0 0 0 0 0 0 0 0 1 0 0 P testis n4 X25293947

X25293947testis.P.n5 1 0 0 0 0 0 0 0 0 0 0 0 0 0 0 0 0 1 0 0 P testis n5 X25293947

X25293947testis.J0.n1 0 1 0 0 0 0 0 0 0 0 0 0 0 0 0 0 0 1 0 0 J0 testis n1 X25293947

X25293947testis.J0.n2 0 1 0 0 0 0 0 0 0 0 0 0 0 0 0 0 0 1 0 0 J0 testis n2 X25293947

X25293947testis.J0.n3 0 1 0 0 0 0 0 0 0 0 0 0 0 0 0 0 0 1 0 0 J0 testis n3 X25293947

X25293947testis.J0.n4 0 1 0 0 0 0 0 0 0 0 0 0 0 0 0 0 0 1 0 0 J0 testis n4 X25293947

X25293947testis.J0.n5 0 1 0 0 0 0 0 0 0 0 0 0 0 0 0 0 0 1 0 0 J0 testis n5 X25293947

X25293947testis.J1.n1 0 0 1 0 0 0 0 0 0 0 0 0 0 0 0 0 0 1 0 0 J1 testis n1 X25293947

X25293947testis.J1.n2 0 0 1 0 0 0 0 0 0 0 0 0 0 0 0 0 0 1 0 0 J1 testis n2 X25293947

X25293947testis.J1.n3 0 0 1 0 0 0 0 0 0 0 0 0 0 0 0 0 0 1 0 0 J1 testis n3 X25293947

X25293947testis.J1.n4 0 0 1 0 0 0 0 0 0 0 0 0 0 0 0 0 0 1 0 0 J1 testis n4 X25293947

X25293947testis.J1.n5 0 0 1 0 0 0 0 0 0 0 0 0 0 0 0 0 0 1 0 0 J1 testis n5 X25293947

X25293947testis.J2.n1 0 0 0 1 0 0 0 0 0 0 0 0 0 0 0 0 0 1 0 0 J2 testis n1 X25293947

X25293947testis.J2.n2 0 0 0 1 0 0 0 0 0 0 0 0 0 0 0 0 0 1 0 0 J2 testis n2 X25293947

X25293947testis.J2.n3 0 0 0 1 0 0 0 0 0 0 0 0 0 0 0 0 0 1 0 0 J2 testis n3 X25293947

X25293947testis.J2.n4 0 0 0 1 0 0 0 0 0 0 0 0 0 0 0 0 0 1 0 0 J2 testis n4 X25293947

X25293947testis.J2.n5 0 0 0 1 0 0 0 0 0 0 0 0 0 0 0 0 0 1 0 0 J2 testis n5 X25293947

X25293947testis.J3.n1 0 0 0 0 1 0 0 0 0 0 0 0 0 0 0 0 0 1 0 0 J3 testis n1 X25293947

X25293947testis.J3.n2 0 0 0 0 1 0 0 0 0 0 0 0 0 0 0 0 0 1 0 0 J3 testis n2 X25293947

X25293947testis.J3.n3 0 0 0 0 1 0 0 0 0 0 0 0 0 0 0 0 0 1 0 0 J3 testis n3 X25293947

X25293947testis.J3.n4 0 0 0 0 1 0 0 0 0 0 0 0 0 0 0 0 0 1 0 0 J3 testis n4 X25293947

X25293947testis.J3.n5 0 0 0 0 1 0 0 0 0 0 0 0 0 0 0 0 0 1 0 0 J3 testis n5 X25293947

X25293947testis.J4.n1 0 0 0 0 0 1 0 0 0 0 0 0 0 0 0 0 0 1 0 0 J4 testis n1 X25293947

X25293947testis.J4.n2 0 0 0 0 0 1 0 0 0 0 0 0 0 0 0 0 0 1 0 0 J4 testis n2 X25293947

X25293947testis.J4.n3 0 0 0 0 0 1 0 0 0 0 0 0 0 0 0 0 0 1 0 0 J4 testis n3 X25293947

X25293947testis.J4.n4 0 0 0 0 0 1 0 0 0 0 0 0 0 0 0 0 0 1 0 0 J4 testis n4 X25293947

X25293947testis.J4.n5 0 0 0 0 0 1 0 0 0 0 0 0 0 0 0 0 0 1 0 0 J4 testis n5 X25293947

X25293947testis.J7.n1 0 0 0 0 0 0 1 0 0 0 0 0 0 0 0 0 0 1 0 0 J7 testis n1 X25293947

X25293947testis.J7.n2 0 0 0 0 0 0 1 0 0 0 0 0 0 0 0 0 0 1 0 0 J7 testis n2 X25293947

X25293947testis.J7.n3 0 0 0 0 0 0 1 0 0 0 0 0 0 0 0 0 0 1 0 0 J7 testis n3 X25293947

X25293947testis.J7.n4 0 0 0 0 0 0 1 0 0 0 0 0 0 0 0 0 0 1 0 0 J7 testis n4 X25293947

X25293947testis.J7.n5 0 0 0 0 0 0 1 0 0 0 0 0 0 0 0 0 0 1 0 0 J7 testis n5 X25293947

X27404005oocytes.AB.n1 0 0 0 0 0 0 0 1 0 0 0 0 0 0 0 0 0 0 1 0 AB oocytes n1 X27404005

X27404005oocytes.AB.n2 0 0 0 0 0 0 0 1 0 0 0 0 0 0 0 0 0 0 1 0 AB oocytes n2 X27404005

X27404005oocytes.AB.n3 0 0 0 0 0 0 0 1 0 0 0 0 0 0 0 0 0 0 1 0 AB oocytes n3 X27404005

X27404005oocytes.AB.n4 0 0 0 0 0 0 0 1 0 0 0 0 0 0 0 0 0 0 1 0 AB oocytes n4 X27404005

X27404005oocytes.AB.n5 0 0 0 0 0 0 0 1 0 0 0 0 0 0 0 0 0 0 1 0 AB oocytes n5 X27404005

X27404005oocytes.C1.n1 0 0 0 0 0 0 0 0 1 0 0 0 0 0 0 0 0 0 1 0 C1 oocytes n1 X27404005

X27404005oocytes.C1.n2 0 0 0 0 0 0 0 0 1 0 0 0 0 0 0 0 0 0 1 0 C1 oocytes n2 X27404005

X27404005oocytes.C1.n3 0 0 0 0 0 0 0 0 1 0 0 0 0 0 0 0 0 0 1 0 C1 oocytes n3 X27404005

X27404005oocytes.C1.n4 0 0 0 0 0 0 0 0 1 0 0 0 0 0 0 0 0 0 1 0 C1 oocytes n4 X27404005

X27404005oocytes.C1.n5 0 0 0 0 0 0 0 0 1 0 0 0 0 0 0 0 0 0 1 0 C1 oocytes n5 X27404005

X27404005oocytes.C2.n1 0 0 0 0 0 0 0 0 0 1 0 0 0 0 0 0 0 0 1 0 C2 oocytes n1 X27404005

X27404005oocytes.C2.n2 0 0 0 0 0 0 0 0 0 1 0 0 0 0 0 0 0 0 1 0 C2 oocytes n2 X27404005

X27404005oocytes.C2.n3 0 0 0 0 0 0 0 0 0 1 0 0 0 0 0 0 0 0 1 0 C2 oocytes n3 X27404005

X27404005oocytes.C2.n4 0 0 0 0 0 0 0 0 0 1 0 0 0 0 0 0 0 0 1 0 C2 oocytes n4 X27404005

X27404005oocytes.C2.n5 0 0 0 0 0 0 0 0 0 1 0 0 0 0 0 0 0 0 1 0 C2 oocytes n5 X27404005

X27404005oocytes.D1.n1 0 0 0 0 0 0 0 0 0 0 1 0 0 0 0 0 0 0 1 0 D1 oocytes n1 X27404005

X27404005oocytes.D1.n2 0 0 0 0 0 0 0 0 0 0 1 0 0 0 0 0 0 0 1 0 D1 oocytes n2 X27404005

X27404005oocytes.D1.n3 0 0 0 0 0 0 0 0 0 0 1 0 0 0 0 0 0 0 1 0 D1 oocytes n3 X27404005

X27404005oocytes.D1.n4 0 0 0 0 0 0 0 0 0 0 1 0 0 0 0 0 0 0 1 0 D1 oocytes n4 X27404005

X27404005oocytes.D1.n5 0 0 0 0 0 0 0 0 0 0 1 0 0 0 0 0 0 0 1 0 D1 oocytes n5 X27404005

X27404005oocytes.D2.n1 0 0 0 0 0 0 0 0 0 0 0 1 0 0 0 0 0 0 1 0 D2 oocytes n1 X27404005

X27404005oocytes.D2.n2 0 0 0 0 0 0 0 0 0 0 0 1 0 0 0 0 0 0 1 0 D2 oocytes n2 X27404005

X27404005oocytes.D2.n3 0 0 0 0 0 0 0 0 0 0 0 1 0 0 0 0 0 0 1 0 D2 oocytes n3 X27404005

X27404005oocytes.D2.n4 0 0 0 0 0 0 0 0 0 0 0 1 0 0 0 0 0 0 1 0 D2 oocytes n4 X27404005

X27404005oocytes.D2.n5 0 0 0 0 0 0 0 0 0 0 0 1 0 0 0 0 0 0 1 0 D2 oocytes n5 X27404005

X27404005embryo.S1.n1 0 0 0 0 0 0 0 0 0 0 0 0 1 0 0 0 0 0 0 1 S1 embryo n1 X27404005

X27404005embryo.S1.n2 0 0 0 0 0 0 0 0 0 0 0 0 1 0 0 0 0 0 0 1 S1 embryo n2 X27404005

X27404005embryo.S1.n3 0 0 0 0 0 0 0 0 0 0 0 0 1 0 0 0 0 0 0 1 S1 embryo n3 X27404005

X27404005embryo.S1.n4 0 0 0 0 0 0 0 0 0 0 0 0 1 0 0 0 0 0 0 1 S1 embryo n4 X27404005

X27404005embryo.S1.n5 0 0 0 0 0 0 0 0 0 0 0 0 1 0 0 0 0 0 0 1 S1 embryo n5 X27404005

X27404005embryo.S2.n1 0 0 0 0 0 0 0 0 0 0 0 0 0 1 0 0 0 0 0 1 S2 embryo n1 X27404005

X27404005embryo.S2.n2 0 0 0 0 0 0 0 0 0 0 0 0 0 1 0 0 0 0 0 1 S2 embryo n2 X27404005

X27404005embryo.S2.n3 0 0 0 0 0 0 0 0 0 0 0 0 0 1 0 0 0 0 0 1 S2 embryo n3 X27404005

X27404005embryo.S2.n4 0 0 0 0 0 0 0 0 0 0 0 0 0 1 0 0 0 0 0 1 S2 embryo n4 X27404005

X27404005embryo.S2.n5 0 0 0 0 0 0 0 0 0 0 0 0 0 1 0 0 0 0 0 1 S2 embryo n5 X27404005

X27404005embryo.S3.n1 0 0 0 0 0 0 0 0 0 0 0 0 0 0 1 0 0 0 0 1 S3 embryo n1 X27404005

X27404005embryo.S3.n2 0 0 0 0 0 0 0 0 0 0 0 0 0 0 1 0 0 0 0 1 S3 embryo n2 X27404005

X27404005embryo.S3.n3 0 0 0 0 0 0 0 0 0 0 0 0 0 0 1 0 0 0 0 1 S3 embryo n3 X27404005

X27404005embryo.S3.n4 0 0 0 0 0 0 0 0 0 0 0 0 0 0 1 0 0 0 0 1 S3 embryo n4 X27404005

X27404005embryo.S3.n5 0 0 0 0 0 0 0 0 0 0 0 0 0 0 1 0 0 0 0 1 S3 embryo n5 X27404005

X27404005embryo.S4.n1 0 0 0 0 0 0 0 0 0 0 0 0 0 0 0 1 0 0 0 1 S4 embryo n1 X27404005

X27404005embryo.S4.n2 0 0 0 0 0 0 0 0 0 0 0 0 0 0 0 1 0 0 0 1 S4 embryo n2 X27404005

X27404005embryo.S4.n3 0 0 0 0 0 0 0 0 0 0 0 0 0 0 0 1 0 0 0 1 S4 embryo n3 X27404005

X27404005embryo.S4.n4 0 0 0 0 0 0 0 0 0 0 0 0 0 0 0 1 0 0 0 1 S4 embryo n4 X27404005

X27404005embryo.S4.n5 0 0 0 0 0 0 0 0 0 0 0 0 0 0 0 1 0 0 0 1 S4 embryo n5 X27404005

X27404005embryo.S5.n1 0 0 0 0 0 0 0 0 0 0 0 0 0 0 0 0 1 0 0 1 S5 embryo n1 X27404005

X27404005embryo.S5.n2 0 0 0 0 0 0 0 0 0 0 0 0 0 0 0 0 1 0 0 1 S5 embryo n2 X27404005

X27404005embryo.S5.n3 0 0 0 0 0 0 0 0 0 0 0 0 0 0 0 0 1 0 0 1 S5 embryo n3 X27404005

X27404005embryo.S5.n4 0 0 0 0 0 0 0 0 0 0 0 0 0 0 0 0 1 0 0 1 S5 embryo n4 X27404005

X27404005embryo.S5.n5 0 0 0 0 0 0 0 0 0 0 0 0 0 0 0 0 1 0 0 1 S5 embryo n5 X27404005
